# Identification of polyunsaturated fatty acids related key modules and genes in metabolic dysfunction-associated fatty liver disease using WGCNA analysis

**DOI:** 10.3389/fgene.2022.951224

**Published:** 2022-11-08

**Authors:** Cheng Xiao, Siliang Chen, Chunru Yang, Jieying Liu, Miao Yu

**Affiliations:** ^1^ Department of Endocrinology, Key Laboratory of Endocrinology, National Health Commission, Peking Union Medical College Hospital, Peking Union Medical College, Chinese Academy of Medical Sciences, Beijing, China; ^2^ Department of Dermatology and Venereology, West China Hospital, Sichuan University, Chengdu, China; ^3^ Laboratory of Dermatology, Clinical Institute of Inflammation and Immunology, Frontiers Science Centre for Disease-related Molecular Network, West China Hospital, Sichuan University, Chengdu, China; ^4^ Department of Medical Research Center, Peking Union Medical College Hospital, Peking Union Medical College, Chinese Academy of Medical Sciences, Beijing, China

**Keywords:** WGCNA, metabolic dysfunction-associated fatty liver disease, polyunsaturated fatty acids, differentially expressed genes, ADAMTS1 gene, TGFβ3 gene

## Abstract

Polyunsaturated fatty acids (PUFAs) play important roles in the aetiology and pathogenesis of metabolic dysfunction-associated fatty liver disease (MAFLD). However, the underlying molecular mechanisms are not understood. We analysed a public GEO dataset, GSE89632, to identify differentially expressed genes (DEGs) in MAFLD. Weighted gene coexpression network analysis (WGCNA) was used to reveal the core gene regulation network and to explore the PUFA-related hub genes in MAFLD. We experimentally verified these genes by quantitative reverse transcription PCR in high-fat diet (HFD)-fed mice. A total of 286 common DEGs (89 upregulated; 197 downregulated), mostly related to inflammatory and immune responses, were identified. Six modules were constructed using WGCNA, and 2 modules showed significant correlations with PUFAs. After combining these 2 modules with DEGs, the top 10 hub genes were identified. We further established a MAFLD mouse model with liver steatosis, as proved by HE and Oil Red O staining. Of the hub genes, ADAM metallopeptidase with thrombospondin type 1 motif 1 (*adamts1*) (*p* = 0.005) and transforming growth factor β3 (*tgfβ3*) (*p* < 0.001) showed significantly lower mRNA expression in MAFLD *in vivo*. *adamts1* and *tgfβ3* bridged PUFAs and MAFLD, which might be potential causative genes and therapeutic targets of MAFLD.

## Introduction

Metabolic dysfunction-associated fatty liver disease (MAFLD), which is also referred to as nonalcoholic fatty liver disease (NAFLD) (Eslam et al., 2020), includes a series of chronic liver diseases, ranging from steatosis (SS) to nonalcoholic steatohepatitis (NASH), cirrhosis, and hepatocellular carcinoma. Previous studies reported that MAFLD was closely associated with an increased risk of developing cardiovascular diseases, type 2 diabetes, and other diseases (Anstee et al., 2013; Park et al., 2013; Adams et al., 2017). Currently, one-quarter of the world’s population suffers from MAFLD (Younossi et al., 2019), which is a major health problem worldwide. However, the molecular aetiology and appropriate pharmacotherapeutic approaches for MALFD have not yet been elucidated (Lim et al., 2021). Therefore, there is an urgent need to elucidate the molecular mechanism underlying the pathogenesis of MALFD.

A “two-hit hypothesis” was proposed to explain the pathogenesis of MAFLD (Day and James, 1998; Dowman et al., 2010). According to this theory, lipid accumulation or SS in the liver in the form of triglyceride (TG) and free fatty acids are considered the first hit, rendering the liver more vulnerable to inflammatory insult, which acts as the second hit. Cytokines and chemokines (Peverill et al., 2014) secreted by hepatocytes or activated neutrophils further enhance injury to hepatic tissue, which leads to MAFLD (Machado and Diehl, 2016; Koyama and Brenner, 2017; Schuster et al., 2018). Multiple studies have suggested that polyunsaturated fatty acids (PUFAs) play important roles in the aetiology of MAFLD. It has been reported that dietary PUFA intake is lower in individuals with MAFLD (Puri et al., 2007; Da Silva et al., 2014; Arendt et al., 2015), and PUFA supplementation may ameliorate the clinical symptoms of NASH(Li et al., 2015). One of the explanations for the relationship between PUFAs and MAFLD is that PUFAs contribute to the improvement in lipid metabolism, increase in insulin sensitivity, and amelioration of inflammation (Di Minno et al., 2012; Imamura et al., 2016; Schulze et al., 2020), which further lead to a lower risk of MAFLD. However, the underlying mechanism needs to be further elucidated.

To identify crucial PUFAs-related genes in MAFLD, we used a public GEO dataset to analyse differentially expressed genes (DEGs) between MAFLD patients and healthy controls (HCs) and to determine their biological functions. Now many bioinformatics methods have been developed to identify potential therapeutic targets (Wang et al., 2022) (LIANG et al., 2022). Weighted gene coexpression network analysis (WGCNA) is a tool used to identify modules as candidate regulators and drivers of disease states (Langfelder and Horvath, 2008), and is widely used in liver diseases including porto-sinusoidal vascular disease, cholangiocarcinoma and hepatocellular carcinoma (Hernández-Gea et al., 2021; Long et al., 2021; XU et al., 2022). Besides, a recent study applied WGCNA to identify two hub genes NDUFA9 and UQCRQ which may be involved in the pathogenesis of MAFLD (Zeng et al., 2021). In this study, we applied WGCNA to construct gene modules and to identify hub genes in the PUFAs-related modules. The bioinformatics results were validated by comparing the expression of these hub genes between mice with MAFLD and control mice using quantitative reverse transcription PCR (RT–qPCR).

## Materials and methods

### Data preprocessing

**FIGURE 1:**
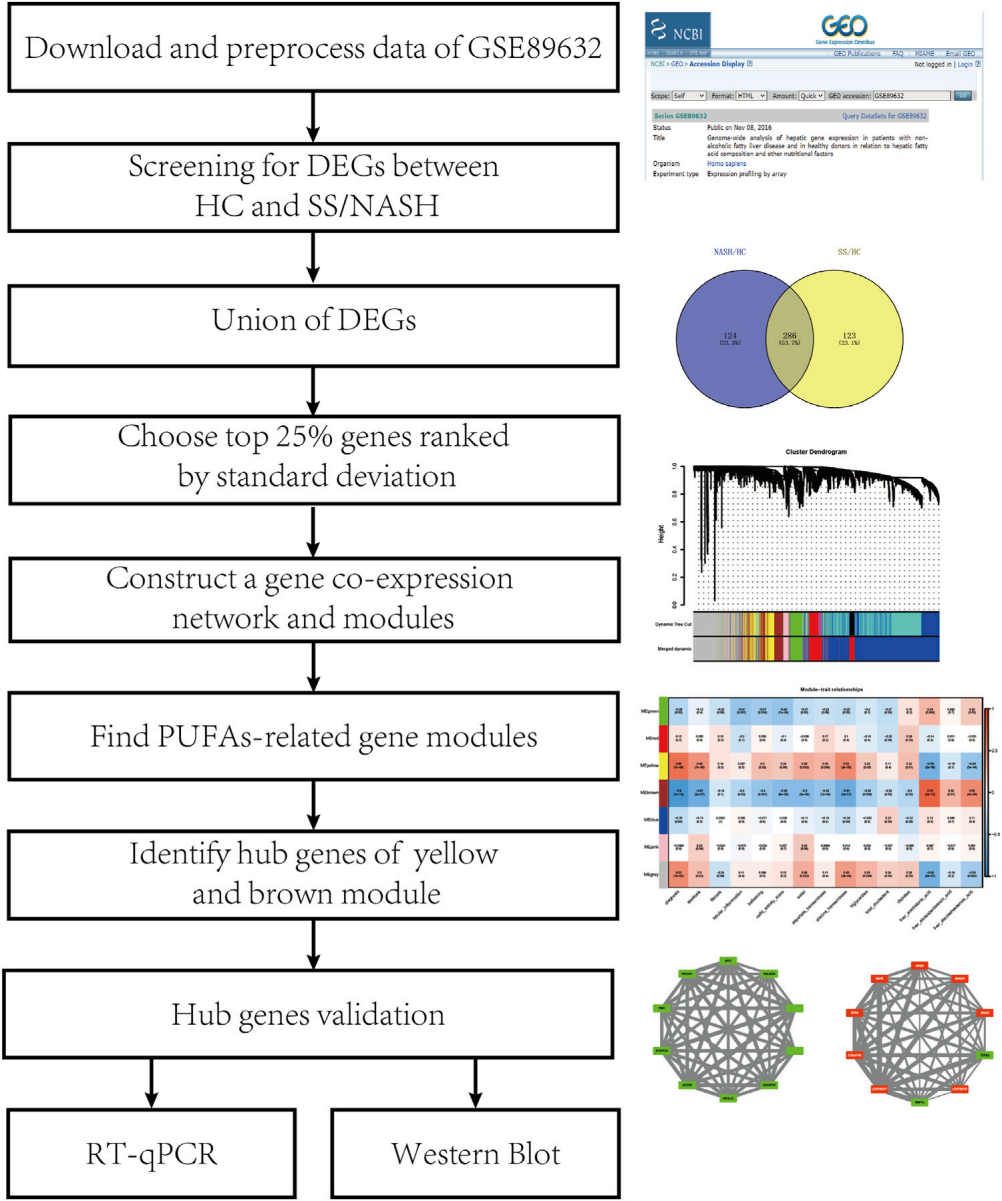
Flowchart of study.

Figure 1 shows the flowchart of the study design. The mRNA microarray dataset GSE89632 from the GEO database was used in our study. We downloaded the series matrix file and GPL14951 platform data table. We sorted the clinical information from the series matrix file, annotated the probe IDs in this matrix and generated an expression matrix with gene symbols using the microarray platform data table. Samples from 24 HCs, 20 patients with simple SS, and 19 patients with NASH were used for further analysis.

### DEG screening

We used the “limma” R package to screen DEGs between HC and patients with SS/NASH. We set the thresholds to |log2 (fold-change)| >1 and adjusted *p* < 0.05 (Lin et al., 2019; Chan et al., 2020; Dobson et al., 2020; Xiu et al., 2020). Then, we combined the DEGs from HC-SS and HC-NASH. We identified the common DEGs of these 2 comparisons. To determine the biological function of these common DEGs, we conducted Gene Ontology (GO) and Kyoto Encyclopedia of Genes Genomes (KEGG) pathway analyses on these DEGs using the “clusterProfiler” R package. We set the cut-off value for the functional annotation analysis to adjusted *p* < 0.05.

### Weighted correlation network construction

We used the “WGCNA” R package for the network analysis. To construct a network with biological significance, we filtered genes with the top 25% expression variance to construct the gene coexpression network. We first built a correlation matrix based on Pearson’s correlation and transformed this correlation matrix into an adjacency matrix with the formula amn = |cmn|β, where amn represents the element in the adjacency matrix, cmn represents the element in the correlation matrix and β represents the soft threshold. To detect gene modules from the coexpression network, we constructed a topological overlap measure (TOM) matrix. We performed average linkage hierarchical clustering and used a dynamic tree cut to detect gene modules using the TOM matrix. We set the minimal module size as 50 and merged similar modules with a threshold of 0.25.

### DEG coexpression network construction and hub gene mining

After gene module detection, we correlated the gene modules with clinical traits using the eigengene of each module. We identified modules with the strongest correlation with the levels of at least one PUFA (i.e., arachidonic acid, eicosapentaenoic acid, and docosahexaenoic acid), and we considered these modules to be PUFAs-related gene modules. We also performed GO analysis on the genes in the PUFAs-related modules to better understand their biological function. To construct DEG coexpression networks for hub gene mining, we mapped common DEGs onto these PUFAs-related modules and constructed DEG coexpression networks. Genes with the top 10 connectivities were considered hub genes in each DEG coexpression network.

### Experimental validation of hub genes

C57BL/6 female mice (HFK Bioscience Co., Ltd., Beijing, China, SCXK-2016-0006) were housed in a specific pathogen-free environment (12-h light/dark cycle) with *ad libitum* access to food and water. After 1 week of adaptation, 6-week-old mice with similar body weights were randomly assigned to 2 groups: the control diet group (CD, *n* = 6), which was fed a standard rodent diet (AIN-93G) (15.8% of the calories as fat); and the high-fat diet group (HFD, *n* = 6), which was fed an HFD (D12492) (60% of the calories as fat). High fat diet formulations were listed in Supplementary Table S5. The oral glucose tolerance test (OGTT, oral administration of 1.0 g/kg of glucose, after 12 h fasting) was performed at the end of the study. All the animal procedures were conducted in compliance with the National Institutes of Health Guide for the Care and Use of Laboratory Animals.

### Pathological staining

Mice were euthanized after 13 weeks to evaluate the pathological changes in their livers. HE staining was conducted according to the protocol of the HE Staining Kit (G1120, Solarbio). Oil Red O staining was performed using Oil Red O kits (G1261, Solarbio). Masson’s staining and Sirius red staining were performed using a Sirius red staining kit (S8060, Solarbio) and Masson’s staining kit (G1340, Solarbio), respectively, according to the manufacturer’s protocol.

### RT–qPCR

Total RNA was extracted from liver tissues using a commercially available kit (Vazyme, RC112). The RNA concentration and quality were determined according to the 260/280 nm ratio, which was measured using a NanoDrop spectrophotometer (ND-100, Thermo Scientific). Total cDNA was synthesized from isolated RNA samples by HiScript III RT SuperMix (Vazyme, R323-01). The mRNA expression of target genes was quantified by RT-qPCR using ChamQ SYBR qPCR Master Mix (Vazyme, Q331-02) on a Light Cycler 480 (Roche, Basel, Switzerland). The GAPDH housekeeping gene (Sangon Biotech, B661304-0001) was used as the internal control. Relative target gene expression was calculated by the 2^−△△CT^ method^.^ The sequences of the primers used in RT-qPCR are listed in Supplementary Table S4.

### Western blot

Proteins from mouse liver samples were extracted using RIPA buffer (APPLYGEN, China) containing protease inhibitors (Solarbio, China) and PMSF (Solarbio, China) Samples containing equal amounts of protein were denatured and subjected to electrophoresis in 10% SDS-PAGE gels followed by transfer to PVDF membrane and probed with specific antibodies, including ADAMTS1 (1:500), TGFβ3 (1:500), and GAPDH (1:1000) (Proteintech, Inc.). Blots bands were visualized using the horseradish peroxidase conjugated secondary antibodies and chemiluminescent substrate.

### Glucose and lipid metabolism parameters measurements

Liver tissues were homogenized in triglyceride assay buffer, and serum was tested directly. TG levels were measured using the Triglyceride Quantification Assay Kit (ab65336, Abcam) according to the manufacturer’s instructions. Fasting blood glucose levels were determined using an automated analyzer (ADVIA1800 Siemens, Germany). Fasting insulin level was measured using the Mouse Insulin ELISA (ALPCO, America) according to the manufacturer’s instructions.

### Statistical analysis

Bioinformatical analyses, such as DEGs screening, WGCNA, and functional annotation, were conducted in R v3.6.2. DEG coexpression network construction and hub gene mining were performed in Cytoscape v3.7.0. The student’s t-test was used to compare data from two groups. A *p* value < 0.05 was considered statistically significant.

## Results

### Filtering of DEGs

**FIGURE 2:**
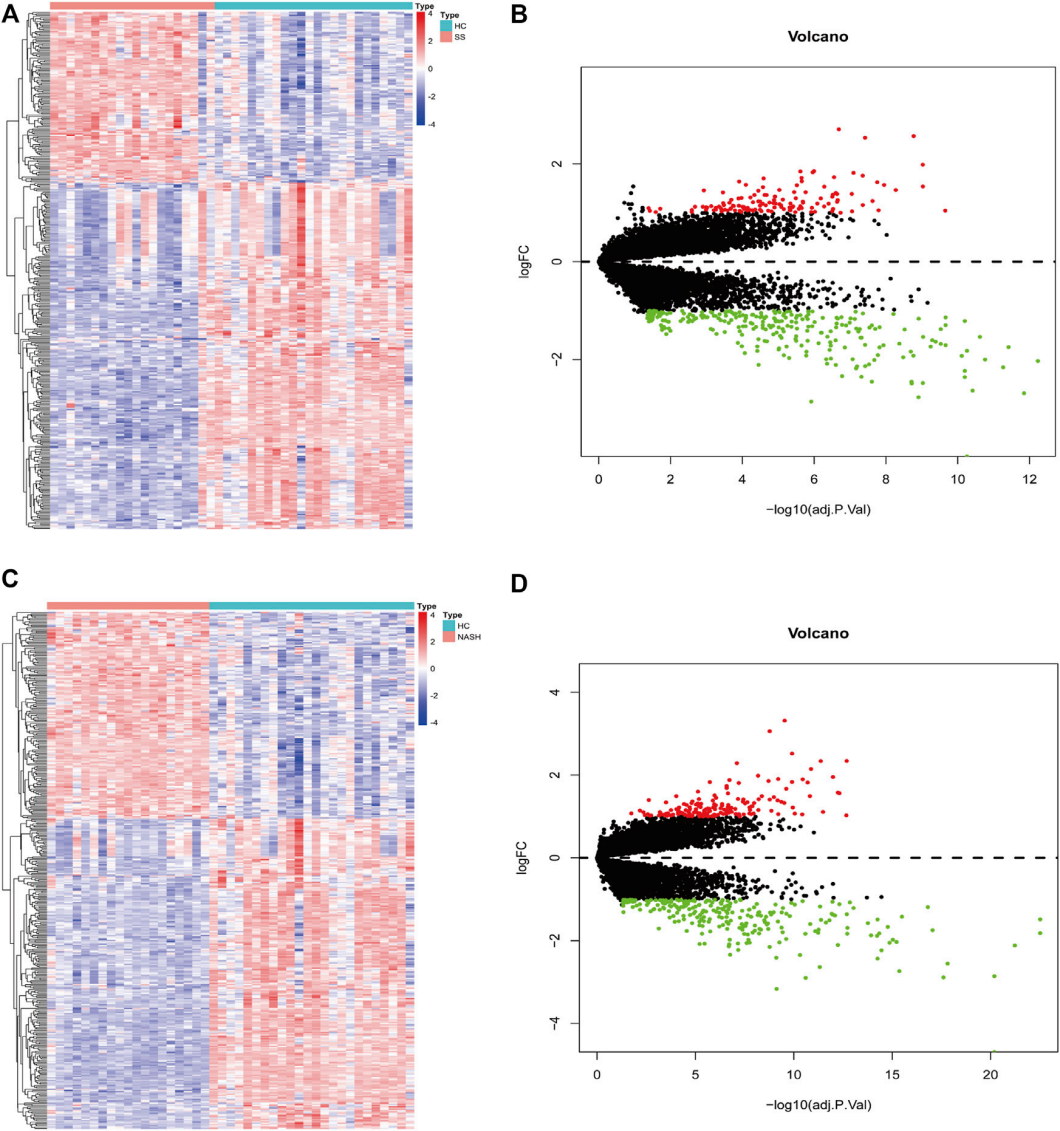
DEGs screening for HC-SS and HC-NASH. In heatmap, red color indicates up-regulation while blue color indicates down-regulation. In volcano plot, red dots represent up-regulated genes while green dots represent down-regulated genes. **(A,B)** Heatmap and volcano plot for HC-SS. **(C,D)** Heatmap and volcano plot for HC-NASH.

**FIGURE 3:**
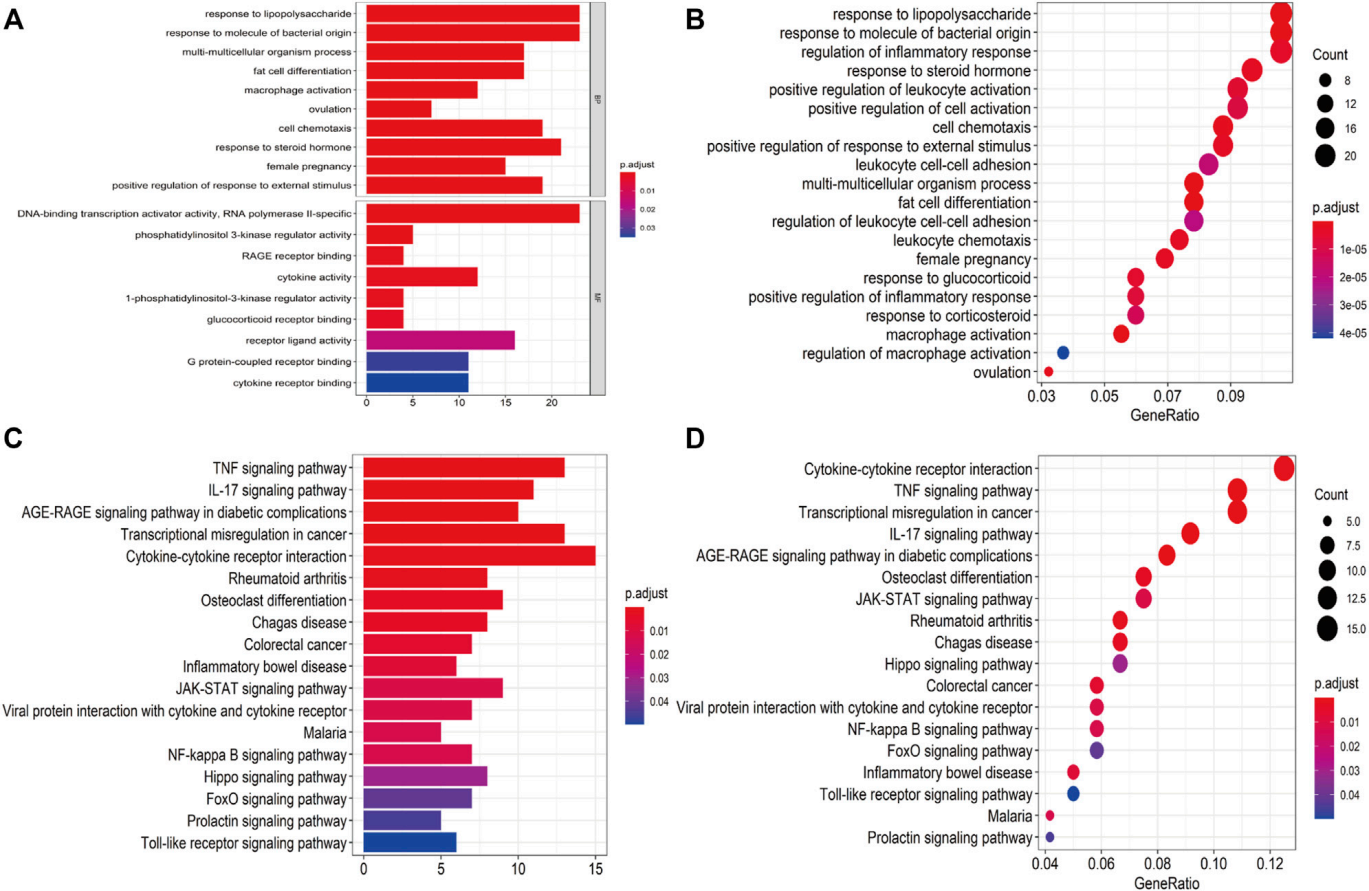
Functional annotation for DEGs. **(A,B)** Bar plot and dot plot of GO analysis for DEGs. **(C,D)** Bar plot and dot plot for KEGG pathway analysis for DEGs.

A total of 409 genes were significantly differentially expressed between the HC and SS groups (Figure 2A), whereas 410 genes were differentially expressed between the HC and the NASH groups (Figure 2C). The heatmap and the volcano plot show the relative expression patterns of these DEGs (Figures 2B,D). A total of 286 DEGs were common in both comparisons, of which 89 were upregulated and 197 were downregulated (Supplementary Figure S1; Supplementary Table S1). The functional annotation analysis of these common DEGs is shown in Figure 3 and Supplementary Table S2. The key GO terms in biological processes were mainly related to macrophage activation (GO: 0042116), positive regulation of inflammatory response (GO: 0050729), and cytokine production involved in immune response (GO: 0002367) (Figures 3A,B). After KEGG analysis, DEGs were mapped to KEGG pathways (Figures 3C,D). The most affected pathways were the tumour necrosis factor (TNF) signalling pathway (hsa04668), interleukin (IL)-17 signalling pathway (hsa04657), and nuclear factor κ-B (NF-κB) signalling pathway (hsa04064). Generally, all of these common DEGs might be primarily involved in inflammatory and immune responses.

### Construction of the gene coexpression network

**FIGURE 4:**
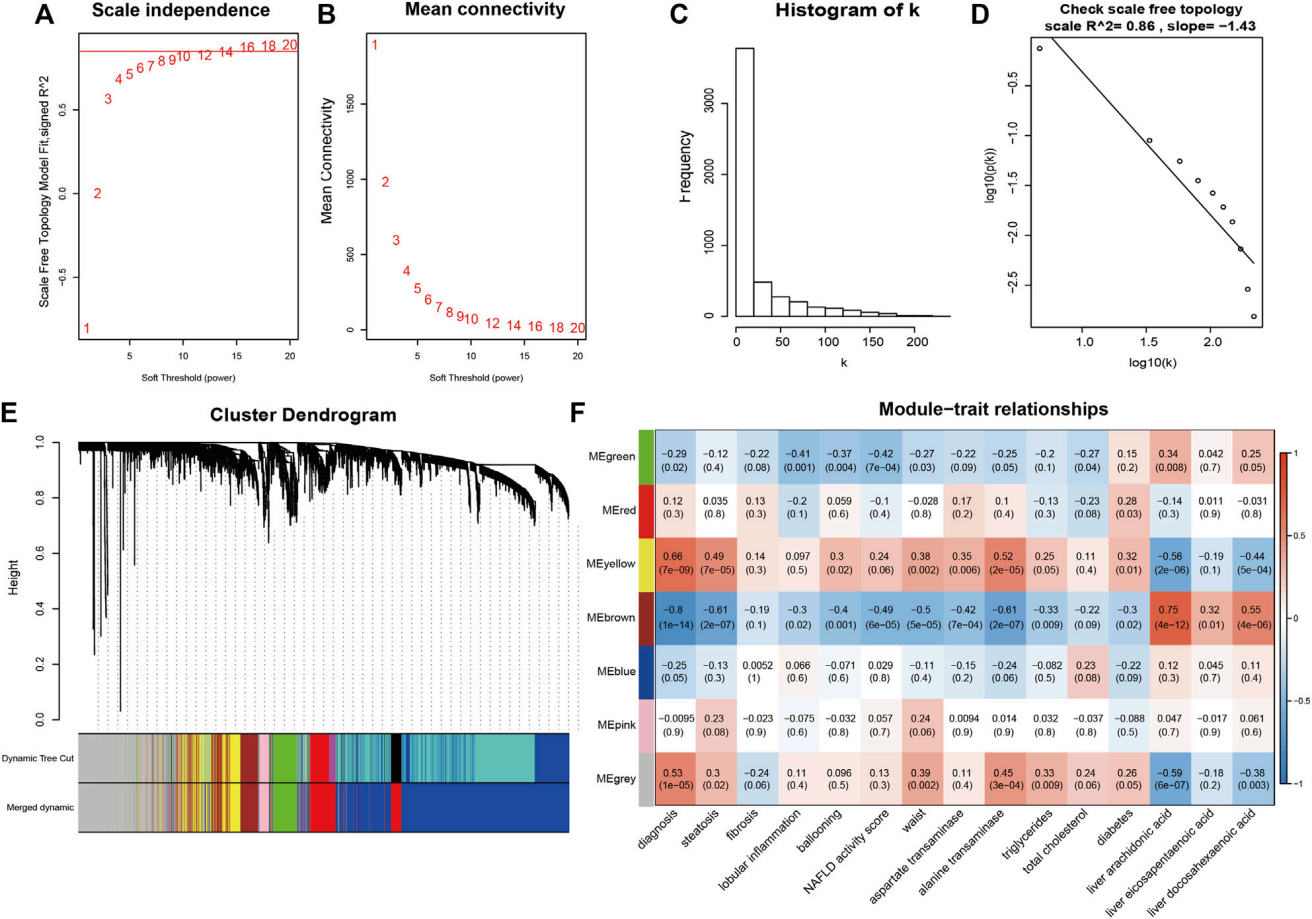
Weighted correlation network construction. **(A,B)** The soft-threshold β was chosen to be 16. **(C,D)** Log-log plot of whole-network connectivity distribution. The x-axis shows the logarithm of the whole network connectivity, and the y-axis is the logarithm of the corresponding frequency distribution. The network met the requirements of scale-free topology. **(E)** A total of 6 module was detected. **(F)** The correlation heatmap for gene modules and traits.

**FIGURE 5:**
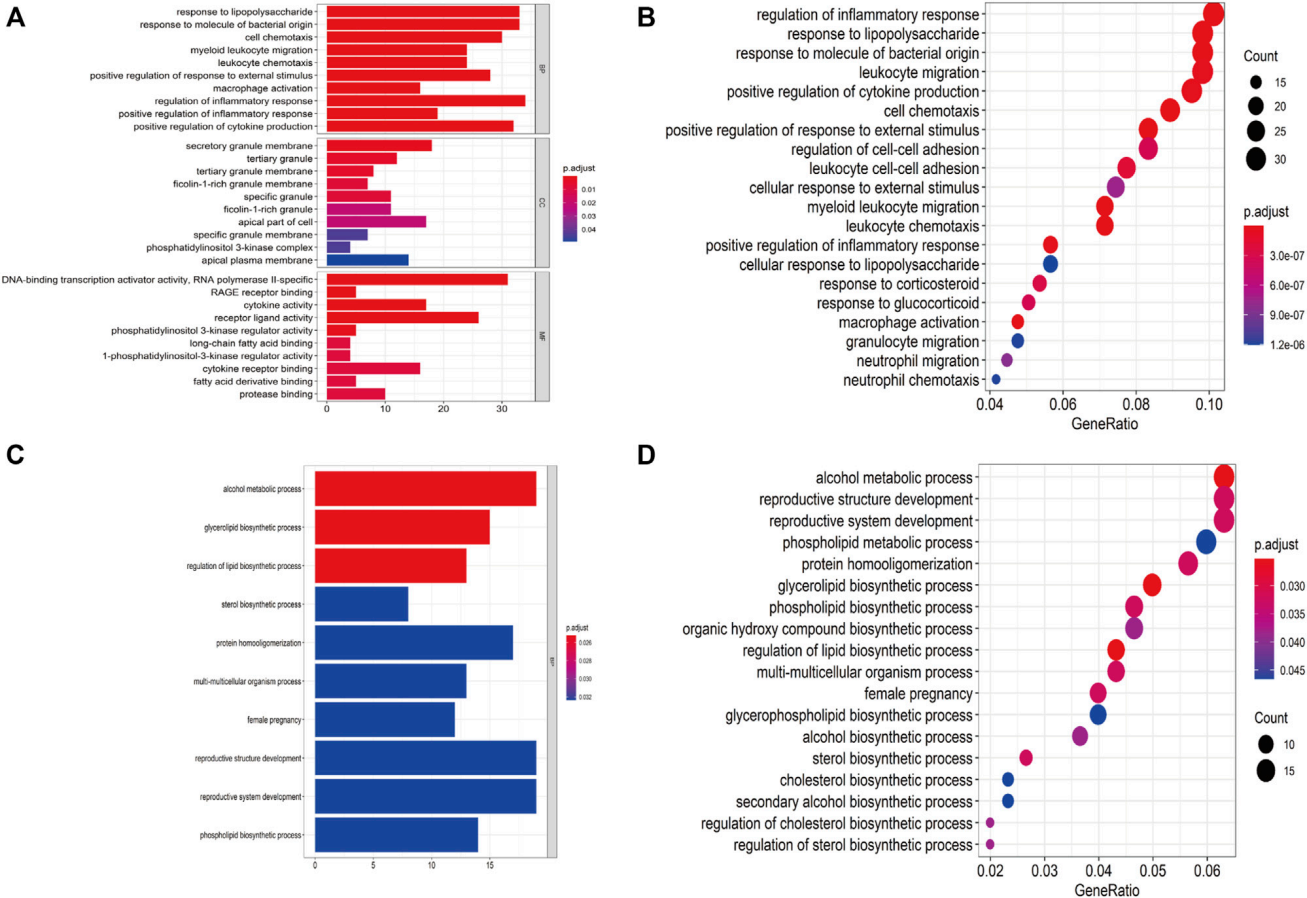
Functional annotation for PUFAs-related modules. **(A,B)** Bar plot and dot plot of GO analysis for brown module. **(C,D)** Bar plot and dot plot for GO analysis for yellow module.

Based on clustering analysis, 2 outliers (GSM2385767 and GSM2385782) were identified and excluded before analysis (Supplementary Figure S2). A cut-off of R^2^ = 0.85 was used to select the soft-threshold β, and β = 16 was selected for network construction (Figures 4A,B). On the log-log plot the distribution approximately follows a straight line (R^2^ = 0.86), which is referred to as approximately scale-free topology. The constructed network met the requirements of scale-free topology. (Figures 4C,D). After module detection, we identified 6 gene modules (Figure 4E), and the correlation of these modules with clinical traits is presented in a heatmap (Figure 4F). Among the 6 gene modules, the brown module had the highest positive correlation with PUFAs levels, while the yellow module had the strongest negative correlation with PUFAs levels (Supplementary Figure S3). Functional annotation analysis showed that the brown module was mainly associated with inflammatory and immune responses (Figures 5A,B; Supplementary Table S3), and the yellow module was mainly associated with lipid metabolism (Figures 5C,D; Supplementary Table S3).

### DEG coexpression network and mining of hub genes

**FIGURE 6:**
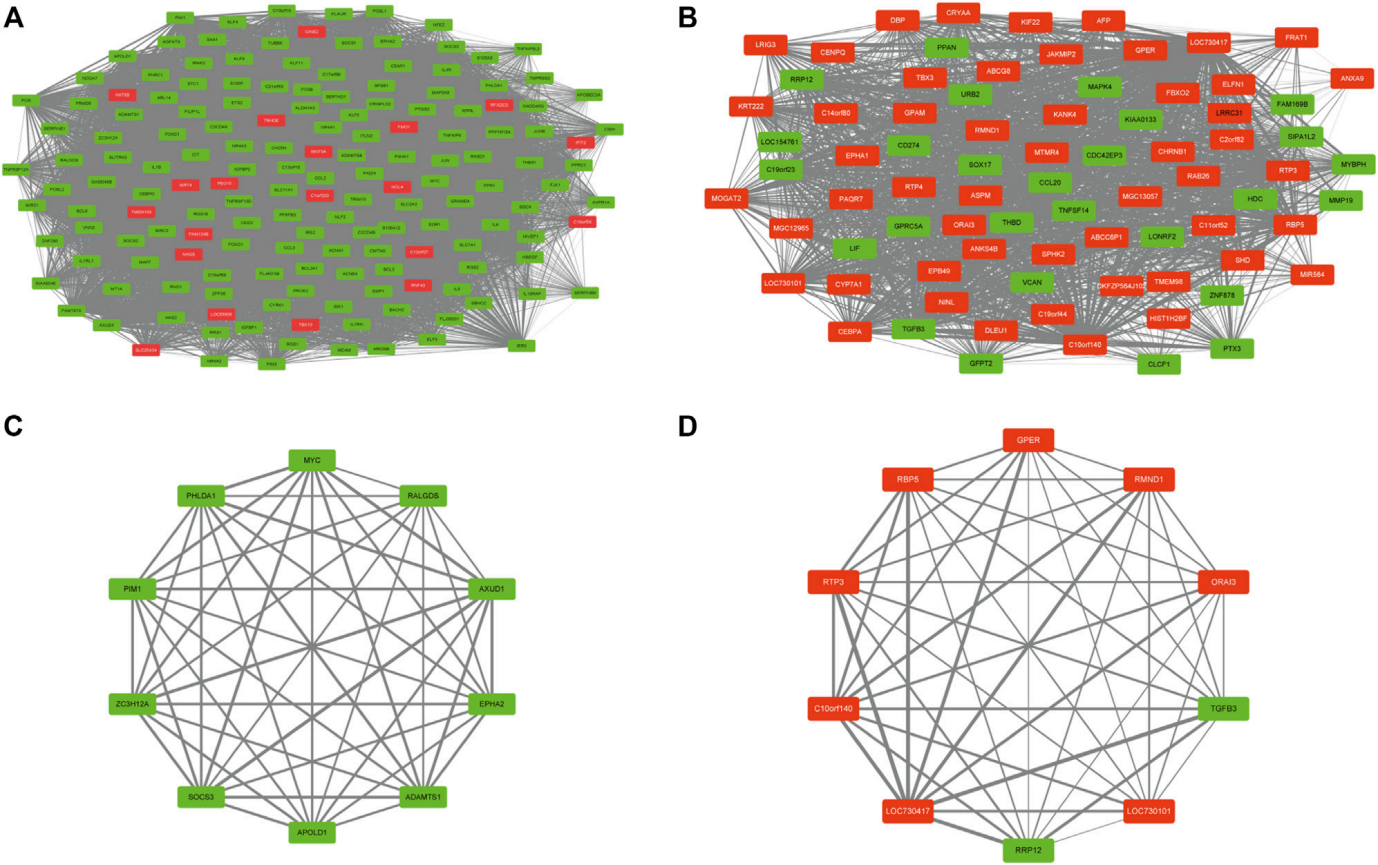
DEG co-expression network and hub genes. **(A,B)** DEG co-expression networks for brown and yellow modules. **(C,D)** hub genes for brown and yellow modules.

To combine DEGs with PUFAs-related modules, we mapped common DEGs onto the brown and yellow gene modules and acquired 2 DEG coexpression networks (Figures 6A,B). In coexpression network, a hub is a node with several links with other nodes that greatly exceed the average. The top 10 connectivity genes were the genes with the greatest number of links and were considered hub genes (Figures 6C,D; Table 1). To verify the hub genes, we established an HFD-fed mouse model to investigate the mRNA expression of these genes.

**TABLE 1:** Crucial genes for the brown and the yellow module.

Module	Crucial gene	Official full name
Brown	PHLDA1	pleckstrin homology like domain family A member 1
	EPHA2	EPH receptor A2
	PIM1	Pim-1 proto-oncogene, serine/threonine kinase
	AXUD1	cysteine and serine rich nuclear protein 1
	SOCS3	suppressor of cytokine signaling 3
	RALGDS	ral guanine nucleotide dissociation stimulator
	MYC	MYC proto-oncogene, bHLH transcription factor
	ADAMTS1	ADAM metallopeptidase with thrombospondin type 1 motif 1
	APOLD1	apolipoprotein L domain containing 1
	ZC3H12A	zinc finger CCCH-type containing 12A
Yellow	GPER	G protein-coupled estrogen receptor 1
	C10orf140	SKI/DACH domain containing 1
	TGFB3	transforming growth factor beta 3
	RBP5	retinol binding protein 5
	ORAI3	ORAI calcium release-activated calcium modulator 3
	LOC730101	—
	LOC730417	—
	RMND1	required for meiotic nuclear division 1 homolog
	RRP12	ribosomal RNA processing 12 homolog
	RTP3	receptor transporter protein 3

### HFD-fed mice developed liver SS and metabolic dysfunction

**FIGURE 7:**
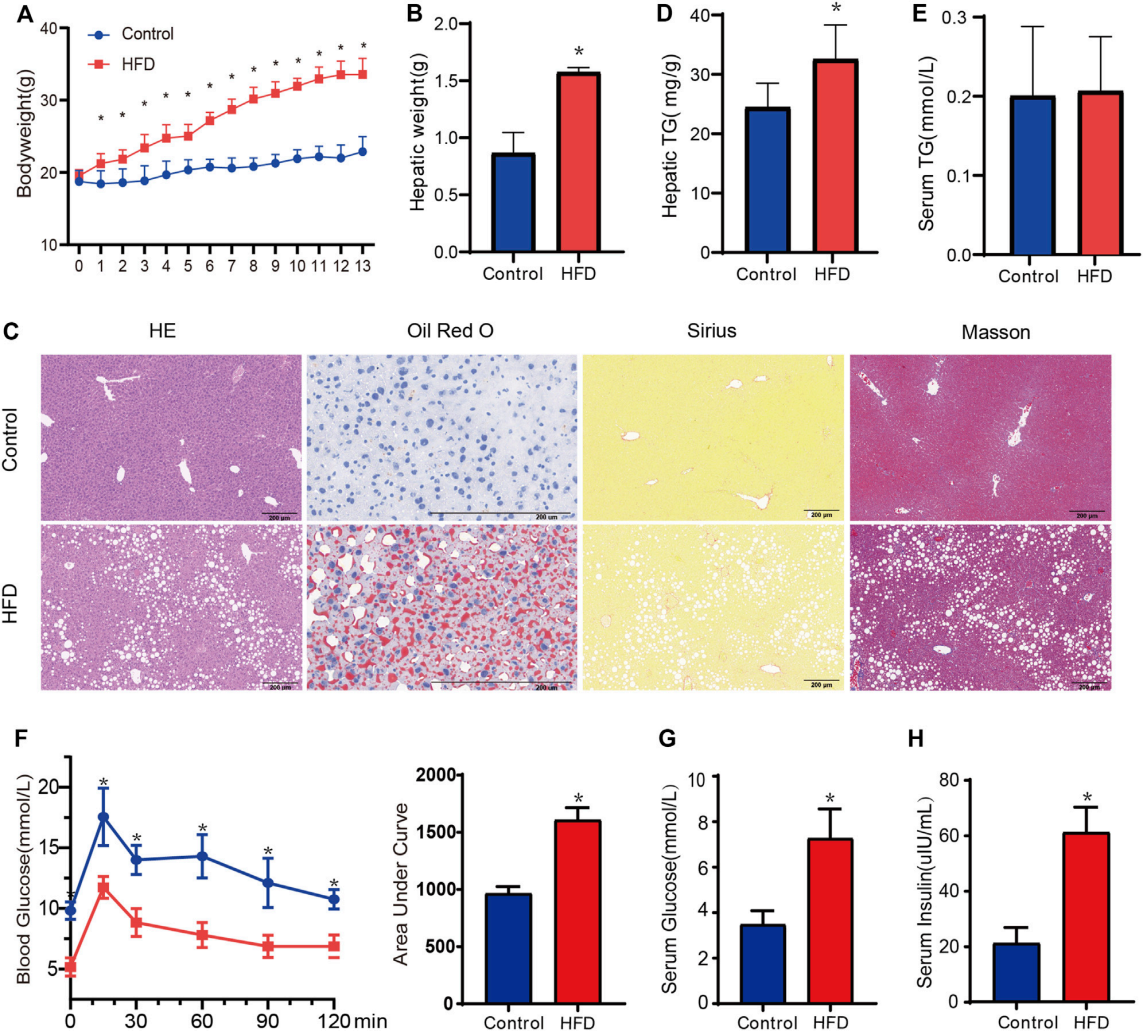
Evaluation of metabolic phenotype in the HFD-fed mice. **(A,B)** The body weight and hepatic weight between HFD and CD groups, respectively. *n* = 6. **(C)**Representative HE staining, Oil-red O staining, Masson staining, and Sirius red staining images of liver from each group. Black scale bar, 200 um. **(D,E)**The hepatic TG and serum TG between HFD and CD group. *n* = 5. **(F)** The oral glucose intolerance test (oral administration of 1.0 g/kg of glucose, after 12 h fasting). **(G,H)** Fasting serum glucose and insulin were measured. *n* = 5. Data are mean ± SD. *p < 0.05 versus CD group.

The body weight and hepatic weight significantly increased in the HFD group compared with the CD group (Figures 7A,B). The liver tissues of mice were stained with HE to observe lipid accumulation in hepatocytes as vacuoles (Figure 7C). Oil Red O staining confirmed the presence of massive lipid deposition in the livers of HFD-fed mice but not in those of control mice (Figure 7C). Masson’s staining and Sirius red staining, which were used to detect fibrosis, revealed no significant differences between the groups (Figure 7C). The HFD group also showed significantly higher hepatic TG contents (*p* = 0.031, Figure 7D), although there was no significance in the serum TG levels (Figure 7E). In addition, OGTT and area under curve of OGTT further showed impaired glucose tolerance in HFD-fed mice (Figure 7F). Both levels of serum glucose and insulin were significantly higher in HFD-fed mice (Figures 7G,H), which meant the HFD-fed mice developed insulin resistance. Overall, all of the above results confirmed the development of MAFLD in HFD-fed mice without obvious fibrosis.

### Validation of hub genes in HFD-fed mice

**FIGURE 8:**
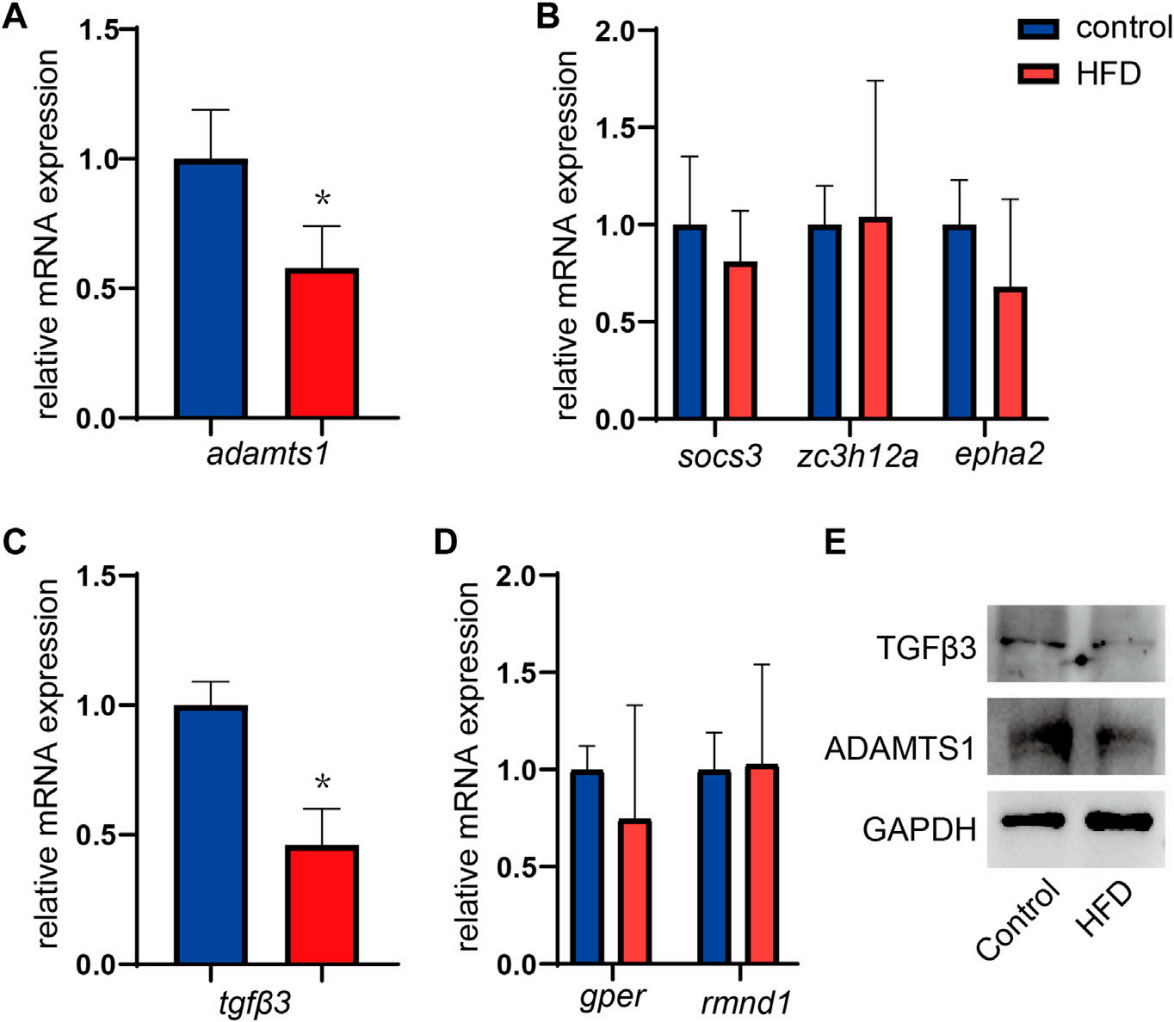
**(A,B)** Expression of the hub genes in the brown modules. *n* = 5. **(C,D)** Expression of the hub genes in the yellow modules. Results represent the mean ± SD of five to six independent experiments. *n* = 5. **(E)** One representative western blot of three independent western blot experiments is shown. Data are mean ± SD.*p < 0.05 versus CD group.

To verify whether the hub genes identified in the two DEG coexpression networks were variably expressed, mRNA was extracted from liver tissues, and gene expression was quantified by RT-qPCR. It has been shown that ADAM metallopeptidase with thrombospondin type 1 motif 1 (*adamts1*), which is a gene in the brown module, was expressed at significantly lower levels in HFD-fed mice, and these results matched expectations well (*p* = 0.005, Figure 8A). However, the expression of the *socs3*, *epha2*, and *zc3h12a* genes showed no significant group differences (all *p* > 0.05, Figure 8B). In the yellow module, significantly lower *tgfβ3* expression was observed in the HFD-fed mice (*p* < 0.001, Figure 8C), although *rmnd1* and *gper* expression did not differ significantly between the groups (all *p* > 0.05) (Figure 8D). The changes of TGFβ3 and ADAMTS1 at the protein levels were further confirmed by WB assays. HFD-fed mice showed lower expression of TGFβ3 and ADAMTS1 (Figure 8E).

## Discussion

In the present study, 286 common DEGs, which were mainly involved in inflammation and immune responses, were identified between HCs and patients with MAFLD. Based on the WGCNA, we constructed 6 modules, of which one inflammation-related and one lipid metabolism-related module were most strongly associated with PUFAs. The genes with the top 10 connectivities within the 2 modules were mined as hub genes. For the first time, we confirmed that two hub genes, namely, *adamts1* and *tgfβ3*, were significantly differentially expressed between healthy mice and HFD-fed mice with MAFLD. These findings suggest that *adamts1* and *tgfβ3* play important roles in the process of MAFLD and may serve as biomarkers for the disease.

In our study, functional annotation analysis showed that pathways related to the inflammatory response, especially the macrophage activation pathway, TNF-α signalling pathway, NF-κB signalling pathway, and IL-17 signalling pathway, might play important roles in MAFLD. Similarly, multiple studies have reported that inflammation is crucial for the process of MAFLD. According to previous studies, the secretion of cytokines and chemokines, including TNF-α, IL-6, and IL-1β, by hepatic macrophages was increased in fatty livers (De Taeye et al., 2007; Stienstra et al., 2010; Chawla et al., 2011). Additionally, NF-κB and TNF-α appear to form positive feedback loops and exacerbate the inflammation and injury of hepatocytes (Rolo et al., 2012). In addition, IL-17, which is produced by pro-inflammatory T helper 17 (Th17) cells, can promote liver inflammation and fibrosis by facilitating the production of IL-6, IL-1, and TNF-α by inflammatory cells and activating hepatic stellate cells to produce collagen type I (Meng et al., 2012; Harley et al., 2014). Conversely, depletion of hepatic macrophages can effectively mitigate the progression of diet-induced SS and hepatic insulin resistance in rodent experiments (Huang et al., 2010; Lanthier et al., 2010; Lanthier et al., 2011). These findings suggested that the inflammatory response was activated in individuals with MAFLD, while macrophages might play a mediator role.

Although the aetiology of MAFLD is far from elucidated, growing evidence suggests that decreased intake of PUFAs is one of the causes of the disease (Puri et al., 2007; Da Silva et al., 2014; Arendt et al., 2015; Li et al., 2015). Based on the WGCNA, our results suggested that PUFAs were associated with MAFLD by regulating the coexpression network of inflammation and lipid metabolism (Figure 3). Consistent with our findings, many studies reported that PUFAs could regulate the TNF-α signalling pathway and NF-κB pathway, which were found to be closely associated with liver injury in MAFLD in our study (Figure 3) and other studies (Zhao et al., 2004; Schmöcker et al., 2007; Tapia et al., 2014). PUFAs, primarily n-3 PUFAs, can inhibit activation of the NF-κB pathway and suppress the inflammatory response (Calder, 2015). Moreover, additional supplementation with n-3 PUFAs can significantly reduce the C-reactive protein, IL-6, and TNF-a levels and further block the progression of western-diet induced MAFLD (Li et al., 2014; Lytle et al., 2017). Regarding lipid metabolism, dietary PUFAs were found to stimulate fatty acid oxidation by binding directly to peroxisome proliferator-activated receptor-α(Pawar et al., 2002). A randomized controlled trial also assessed the association of dietary PUFAs with lipid metabolism and peripheral insulin sensitivity *in vivo* (Hodson et al., 2017). After a 15-18 months intervention, individuals who achieved higher enrichment of erythrocyte docosahexaenoic acid, an PUFA, presented significantly improved hepatic insulin sensitivity, decreased fasting and postprandial plasma triglyceride concentrations, and reduced fasting hepatic *de novo* lipogenesis (Hodson et al., 2017).

Due to the therapeutic role of PUFAs in treating MAFLD, further studies on the core gene regulatory network underlying the PUFAs-MAFLD association would help reveal therapeutic targets for the disease. For the first time, we mined the hub genes of the PUFAs-related gene modules (Table 1) and validated the results in mouse experiments. We found that the expression levels of *adamts1* in the brown inflammation-related module and *tgfβ3* in the yellow lipid metabolism-related module were significantly decreased in mice with MAFLD. These results suggest that *adamts1* and *tgfβ3* may play important roles in the association between PUFAs and MAFLD.

ADAMTS1, the first identified Adamts family member, is characterized by its ability to cleave proteoglycans, aggrecan, collagen, elastin, and other extracellular matrix proteins *via* its metalloprotease-dependent catalytic (Rodriguez-Manzaneque et al., 2009; Esselens et al., 2010) and thrombospondin-dependent regions (Luque et al., 2003); thus, it plays roles in degrading extracellular matrix (ECM) components or inhibiting angiogenesis. A recent study reported that ablation of *adamts1* in adipose tissue led to enlarged adipose tissue mass, reduced insulin sensitivity, and dysregulated lipid metabolism (Chen et al., 2016). In addition, lower mRNA levels of *adamts1* in both subcutaneous and visceral white adipose tissues were associated with higher body mass index in humans (Chen et al., 2016). The reason underlying the above phenomenon might be due to adamts1 enzymatically impairing adipogenesis *via* ECM remodelling (Chen et al., 2016), which indicated the role of ADAMTS1 in maintaining lipids homeostasis. It was also reported that ADAMTS1 served as an extracellular protease that could be activated by prostaglandin F2α, a metabolite of the arachidonic acid (a type of PUFAs) metabolism (Keightley et al., 2010). In addition, ADAMTS1 also identified as an inflammatory associated protein is required for a balanced immune response (Kuno et al., 1997; Rodríguez-Baena et al., 2018). Thus, we speculate that ADAMTS1 decreasing in the liver might indicate abnormal immune response and reflect adipogenesis inhibition *via* ECM remodelling and PUFA regulation. However, further studies are warranted to prove the hypothesis.

TGFβ3, a member of the TGFβ superfamily, plays a multifunctional role in the immune response and ECM formation and regulates cell fate. The mRNA level of *tgfβ3* was shown to be significantly lower in the HFD-fed mice in our study. Consistent with our study, TGFβ3 was reported to maintain lipid homeostasis. For example, overexpression of TGFβ3 was reported to reduce the histopathological damage observed in liver fibrosis (Zhang et al., 2010). Another study suggested that TGFβ3 participates in adipose tissue hypertrophy by regulating adipocyte precursor cell proliferation (Petrus et al., 2018). Moreover, mice with *tgfβ3* haploinsufficiency that are fed an HFD show increased glucose intolerance and weight gain *in vivo* compared to their wild-type littermates. TGFβ3 was also proven to play an anti-inflammatory role and might participate in obesity and insulin resistance. TGFβ3 secretion was also found to be decreased in a genetic model of mice with CD4^+^ T cell-specific KLF10 knockout *in vitro* and *in vivo*, and these mice have a predisposition to obesity, insulin resistance, and fatty liver (Wara et al., 2020). It is quite interesting that TGFβ3 could mediate the secretion of prostaglandin E2, which is also an important metabolite in the arachidonic acid metabolic pathway, to affect macrophage polarization. Interestingly, TGFβ3 not only participates in the inflammatory response (Okamura et al., 2015) but also improves glucose tolerance and phenotypic changes in adipocyte morphology (Hall et al., 2013), suggesting that *tgfβ3* may offer potential therapeutic benefits for MAFLD.

Our study has many strengths. First, we used bioinformatics tools, including DEGs analyses and WGCNA, combined with wet-lab experiments to propose and verify our hypothesis. Second, for the first time, we provide evidence that two hub genes, namely, *adamts1* and *tgfβ3*, might play important roles in the association of PUFAs with MAFLD. Nevertheless, this study has limitations. First, although we mined hub genes that might play a role in the PUFAs-MAFLD association, the MAFLD mouse model we used in the validation experiment was established by feeding on HFD. A model of PUFAs diet supplementation could help us further validate our hypothesis. Second, the specific relationship between DEGs and the development of MAFLD should be clarified. Further insight into the molecular function of *adamts1* and *tgfβ3* in PUFA metabolism and MAFLD pathogenesis is needed.

## Conclusion

In conclusion, PUFAs were associated with a brown inflammation-related gene module and a yellow lipid metabolism-related module of MAFLD. The expression levels of *adamts1* in the brown module and *tgβ3* in the yellow module were downregulated in MAFLD mice. These findings provide evidence regarding the role of *adamts1* and *tgfβ3* in the PUFAs-MAFLD association. Overall, the results of this comprehensive analysis have implications for personalized medicine and are of great clinical significance.

## Data Availability

The datasets presented in this study can be found in online repositories. The names of the repository/repositories and accession number(s) can be found below: http://www.ncbi.nlm.nih.gov/geo/; GSE89632.

## Supplementary material

SUPPLEMENTARY FIGURE S1 Combination of DEGs between HC-SS and HC-NASH.

SUPPLEMENTARY FIGURE S2 Sample clustering before network construction. Two outliers were shown by sample clustering. **(B)** Outliers were excluded in further analysis.

SUPPLEMENTARY FIGURE S3 Module membership (MM) versus gene significance (GS) plot. Genes with higher module membership showed stronger gene significance for PUFAs.
