# Supplementary material for: Identification of polyunsaturated fatty acids related key modules and genes in metabolic dysfunction-associated fatty liver disease using WGCNA analysis
Source: Front Genet. 2022 Nov 8;13:951224. doi: 10.3389/fgene.2022.951224 (PMC9679514; doi:10.3389/fgene.2022.951224)
Supplement: Supplementary file 1 [file Table1.DOCX]

| **Table S1. Common DEGs between HC-NASH and HC-SS** | | | | |
| --- | --- | --- | --- | --- |
| **Gene symbol** | **logFC**  **(NASH/HC)** | **adj.P.Val**  **(NASH/HC)** | **logFC**  **(SS/HC)** | **adj.P.Val**  **(SS/HC)** |
| ABCC6P1 | 1.2604941 | 6.02E-06 | 1.3628318 | 2.12E-05 |
| ABCG8 | 1.0479644 | 3.42E-05 | 1.0581275 | 6.17E-05 |
| ACTG2 | -2.087228 | 1.93E-07 | -1.58122 | 3.56E-05 |
| ADAMTS1 | -2.431981 | 5.57E-15 | -2.358221 | 6.36E-11 |
| ADAMTS4 | -1.377921 | 1.88E-05 | -1.317131 | 8.14E-05 |
| AFP | 1.1207588 | 1.00E-05 | 1.186095 | 2.70E-05 |
| AGPAT9 | -1.664967 | 1.68E-15 | -1.162897 | 1.22E-09 |
| ALDH1A3 | -1.204566 | 8.01E-05 | -1.13272 | 0.0010411 |
| AMOT | 1.0262826 | 2.64E-06 | 1.0654546 | 4.38E-05 |
| ANKS4B | 1.4511227 | 8.93E-07 | 1.423505 | 3.60E-06 |
| ANXA9 | 1.2883945 | 9.37E-06 | 1.0595373 | 0.0025459 |
| APOBEC3A | -1.402045 | 0.0001318 | -1.472973 | 0.0005249 |
| APOLD1 | -2.636628 | 4.80E-12 | -2.47773 | 9.36E-10 |
| ARID5B | -1.21026 | 9.24E-14 | -1.210157 | 6.36E-11 |
| ARL14 | -2.065633 | 6.94E-08 | -2.097234 | 2.96E-08 |
| ASPM | 1.327034 | 3.06E-10 | 1.102559 | 1.16E-06 |
| AVPR1A | -1.628108 | 6.26E-06 | -1.730183 | 3.83E-05 |
| AXUD1 | -1.816468 | 3.04E-23 | -1.535156 | 2.41E-11 |
| BACH2 | -1.439573 | 5.43E-08 | -1.218454 | 1.18E-05 |
| BCL2A1 | -1.234779 | 0.0012424 | -1.191172 | 0.002954 |
| BCL3 | -1.389385 | 8.11E-13 | -1.337323 | 1.50E-08 |
| BCL6 | -1.495105 | 4.15E-12 | -1.374912 | 5.37E-08 |
| BIRC3 | -1.128791 | 3.26E-05 | -1.450176 | 6.18E-07 |
| C10orf10 | -1.795007 | 3.70E-10 | -1.663842 | 7.53E-07 |
| C10orf140 | 2.3383869 | 4.29E-12 | 2.5702092 | 1.69E-09 |
| C11orf52 | 1.4922608 | 4.23E-09 | 1.333297 | 1.23E-05 |
| C12orf27 | 1.0668036 | 3.88E-06 | 1.1350628 | 2.42E-06 |
| C13orf15 | -1.598033 | 1.47E-11 | -1.819317 | 5.53E-11 |
| C14orf80 | 1.5583049 | 2.23E-07 | 1.7126855 | 2.24E-06 |
| C17orf96 | -1.607429 | 2.33E-09 | -1.421257 | 3.46E-07 |
| C18orf56 | 1.8195491 | 1.99E-11 | 1.1903837 | 1.19E-05 |
| C19orf23 | -2.070702 | 3.44E-06 | -1.867311 | 8.56E-05 |
| C19orf44 | 1.0612089 | 3.85E-07 | 1.0717454 | 4.59E-06 |
| C19orf59 | -1.09839 | 0.0003467 | -1.154277 | 0.0004601 |
| C1orf203 | 1.4162981 | 6.12E-09 | 1.29548 | 6.54E-07 |
| C20orf77 | -1.437599 | 0.0002035 | -1.434788 | 0.0003092 |
| C21orf63 | -1.316549 | 4.13E-10 | -1.132863 | 1.73E-07 |
| C2CD4A | -1.252246 | 0.0006516 | -1.152917 | 0.0019481 |
| C2CD4B | -1.592407 | 2.10E-07 | -1.265618 | 5.27E-05 |
| C2orf54 | 1.5091892 | 1.07E-06 | 1.1574158 | 0.0003276 |
| C2orf82 | 1.3394493 | 0.0003119 | 1.6399059 | 0.0001227 |
| C5AR1 | -1.276289 | 4.22E-08 | -1.705428 | 1.17E-08 |
| C6orf129 | 1.225057 | 2.25E-07 | 1.5629905 | 3.03E-06 |
| CCL2 | -1.689538 | 1.89E-13 | -1.836392 | 2.03E-09 |
| CCL20 | -1.132701 | 0.0028972 | -2.083758 | 4.36E-07 |
| CCL3 | -1.174245 | 0.0001737 | -1.00284 | 0.0047201 |
| CD274 | -1.327847 | 8.53E-05 | -1.420454 | 0.0001179 |
| CDC42EP3 | -1.020331 | 5.08E-08 | -1.242682 | 1.02E-07 |
| CDH15 | 1.8291539 | 1.86E-06 | 1.5719969 | 7.97E-05 |
| CEBPA | 1.7354279 | 2.60E-07 | 1.6447206 | 2.20E-06 |
| CEBPD | -1.419946 | 3.21E-16 | -1.136786 | 2.18E-10 |
| CENPQ | 1.0758181 | 6.11E-06 | 1.0616974 | 3.18E-05 |
| CH25H | -1.035669 | 0.0029695 | -1.097244 | 0.0013939 |
| CHRNB1 | 1.1884408 | 1.97E-06 | 1.1230365 | 8.25E-06 |
| CISH | -1.581341 | 2.09E-09 | -1.605285 | 2.52E-07 |
| CIT | -1.064268 | 0.0004792 | -1.340935 | 9.73E-06 |
| CLCF1 | -1.386984 | 2.18E-08 | -1.32807 | 4.30E-06 |
| CMTM2 | -1.541348 | 2.09E-06 | -1.071647 | 0.0029096 |
| CNN1 | -1.482536 | 3.85E-05 | -1.372063 | 0.0007549 |
| CRISPLD2 | -1.367285 | 1.41E-09 | -1.733307 | 5.69E-10 |
| CRYAA | 1.6001232 | 7.42E-06 | 1.7026677 | 2.56E-05 |
| CYP7A1 | 3.0595703 | 1.69E-09 | 2.7069106 | 2.05E-07 |
| CYR61 | -1.822676 | 4.56E-15 | -2.230473 | 6.36E-11 |
| DBP | 1.8792736 | 1.88E-07 | 1.8460111 | 2.42E-06 |
| DEFA1 | 1.0733588 | 0.0181227 | 1.0151589 | 0.0258172 |
| DEFB118 | -1.007074 | 0.0210056 | -1.052554 | 0.021372 |
| DKFZP564J102 | 1.0419646 | 4.71E-06 | 1.0441792 | 1.90E-05 |
| DLEU1 | 1.1476313 | 3.54E-06 | 1.3860301 | 2.30E-07 |
| EGR1 | -1.682751 | 4.19E-10 | -1.908211 | 1.68E-08 |
| ELF3 | -1.176949 | 3.19E-07 | -1.426287 | 5.83E-08 |
| ELFN1 | 1.1320919 | 1.84E-06 | 1.1539051 | 8.42E-06 |
| EMP1 | -1.715109 | 5.51E-09 | -2.133835 | 3.78E-08 |
| EPB49 | 1.0641427 | 0.0003174 | 1.2162452 | 5.06E-05 |
| EPHA1 | 1.1097804 | 1.44E-07 | 1.1475979 | 3.22E-05 |
| EPHA2 | -1.881497 | 5.88E-15 | -1.678593 | 2.97E-10 |
| ETS2 | -1.086148 | 1.34E-10 | -1.156601 | 9.66E-08 |
| FADS1 | 1.3163208 | 7.63E-06 | 1.0644611 | 0.0005491 |
| FAM107A | -2.231696 | 3.52E-15 | -1.922381 | 7.73E-11 |
| FAM124B | 1.1509691 | 3.11E-05 | 1.727532 | 2.79E-07 |
| FAM169B | -1.285539 | 0.0002214 | -1.617145 | 2.38E-05 |
| FAM47E | 1.0153984 | 0.0002722 | 1.0495407 | 0.0001603 |
| FBXO2 | 1.2411635 | 1.47E-08 | 1.1906112 | 4.57E-06 |
| FGF14 | -1.044341 | 1.09E-05 | -1.050759 | 1.21E-05 |
| FILIP1L | -1.37075 | 1.04E-12 | -1.633028 | 4.12E-10 |
| FJX1 | -1.131206 | 0.000117 | -1.025599 | 0.0007461 |
| FLJ36031 | -1.251934 | 1.90E-08 | -1.450236 | 6.44E-07 |
| FLJ42957 | -1.009964 | 0.0166294 | -1.011418 | 0.0248596 |
| FLJ45139 | -1.379253 | 5.77E-06 | -1.190792 | 0.0002032 |
| FMO1 | 2.3410386 | 2.07E-13 | 1.206918 | 2.04E-06 |
| FOS | -2.886247 | 2.49E-18 | -2.768875 | 1.24E-09 |
| FOSB | -4.688055 | 6.42E-21 | -3.979716 | 5.53E-11 |
| FOSL1 | -2.410906 | 7.77E-10 | -2.337233 | 1.67E-07 |
| FOSL2 | -1.97526 | 9.32E-16 | -1.918929 | 1.72E-09 |
| FOXC1 | -1.618104 | 3.95E-07 | -1.579815 | 3.28E-06 |
| FOXO1 | -1.127499 | 8.95E-11 | -1.014021 | 1.45E-06 |
| FPR1 | -1.598662 | 4.39E-08 | -1.483124 | 1.37E-05 |
| FRAT1 | 1.3433991 | 2.31E-08 | 1.3309482 | 3.12E-07 |
| FRMD6 | -1.184354 | 4.17E-05 | -1.247141 | 5.82E-05 |
| GADD45B | -1.48326 | 3.04E-23 | -1.294451 | 2.18E-10 |
| GADD45G | -2.734519 | 4.37E-16 | -2.450445 | 2.47E-08 |
| GAGE12C | -1.158314 | 0.0465716 | -1.476803 | 0.0131396 |
| GAGE12J | -1.108454 | 0.0469048 | -1.294506 | 0.0184073 |
| GAGE2B | -1.010798 | 0.037406 | -1.200124 | 0.0140168 |
| GFPT2 | -1.538362 | 6.18E-06 | -1.492835 | 0.0001023 |
| GFRA3 | -1.251519 | 0.0065018 | -1.225495 | 0.0112809 |
| GIMAP2 | 1.1034716 | 9.59E-06 | 1.1212189 | 9.12E-05 |
| GINS2 | 1.9525094 | 1.03E-12 | 1.3710769 | 3.01E-07 |
| GPAM | 1.1967878 | 5.23E-07 | 1.0391089 | 0.0001179 |
| GPER | 1.0008592 | 4.03E-06 | 1.1172179 | 4.02E-06 |
| GPRC5A | -1.802161 | 1.24E-08 | -1.613363 | 1.52E-06 |
| GRAMD4 | -1.296794 | 2.90E-06 | -1.107942 | 0.0001187 |
| GRIA3 | 1.3890807 | 6.69E-10 | 1.1096767 | 1.27E-05 |
| HAS2 | -1.248746 | 1.81E-08 | -1.139619 | 6.20E-06 |
| HBEGF | -1.829595 | 1.93E-13 | -1.69746 | 3.80E-08 |
| HDC | -1.226423 | 4.42E-07 | -1.173224 | 2.11E-06 |
| HIST1H2BF | 1.2196706 | 1.24E-06 | 1.4215964 | 1.73E-06 |
| HIVEP1 | -1.319566 | 1.60E-09 | -1.300376 | 1.29E-07 |
| IER3 | -1.831247 | 2.60E-11 | -1.992105 | 8.46E-08 |
| IER5L | -1.476844 | 1.87E-06 | -1.44747 | 1.11E-05 |
| IFIT2 | 1.3246954 | 1.04E-08 | 1.1306732 | 5.26E-07 |
| IGFBP1 | -1.834761 | 3.30E-08 | -1.992135 | 4.62E-07 |
| IGFBP2 | -1.542447 | 2.60E-07 | -1.232447 | 9.71E-07 |
| IL18RAP | -1.215591 | 7.09E-07 | -1.083823 | 5.02E-05 |
| IL1B | -1.663307 | 3.82E-08 | -1.837626 | 1.64E-06 |
| IL1RL1 | -2.045341 | 1.77E-08 | -2.108549 | 2.49E-07 |
| IL1RN | -1.172891 | 1.86E-07 | -1.238499 | 3.18E-06 |
| IL4R | -1.560568 | 2.34E-12 | -1.102534 | 2.99E-06 |
| IL6 | -3.16338 | 7.54E-10 | -2.857281 | 1.20E-06 |
| IL8 | -2.149848 | 1.69E-09 | -2.453163 | 1.94E-09 |
| IRAK3 | -1.194722 | 1.05E-08 | -1.142934 | 2.36E-07 |
| IRS2 | -1.100925 | 0.0003537 | -1.102249 | 0.0011858 |
| ISM1 | 1.1549921 | 6.34E-09 | 1.014501 | 3.35E-06 |
| ITLN2 | -1.207796 | 1.37E-05 | -1.067368 | 0.0001737 |
| JAKMIP2 | 1.0899492 | 5.05E-05 | 1.2718829 | 7.45E-06 |
| JMJD5 | 1.1038647 | 0.0044208 | 1.1259891 | 0.0020762 |
| JUN | -1.188618 | 1.55E-17 | -1.603705 | 5.13E-10 |
| JUNB | -2.855501 | 6.42E-21 | -2.632605 | 3.86E-11 |
| KANK4 | 1.0185027 | 0.0020349 | 1.2938429 | 0.0001275 |
| KCNE4 | -1.186048 | 5.34E-05 | -1.102095 | 0.0002865 |
| KCNK1 | -1.732281 | 4.71E-09 | -1.410077 | 1.55E-06 |
| KIAA0040 | -1.850177 | 2.19E-08 | -1.801418 | 3.70E-07 |
| KIAA0133 | -1.290369 | 2.67E-07 | -1.046647 | 3.01E-05 |
| KIF22 | 1.0354714 | 0.0001437 | 1.4503308 | 3.58E-05 |
| KLF11 | -1.505505 | 1.55E-12 | -1.08442 | 4.26E-06 |
| KLF4 | -1.427911 | 2.62E-07 | -1.301847 | 1.79E-05 |
| KLF5 | -1.422731 | 1.47E-08 | -1.124488 | 2.19E-05 |
| KLF6 | -1.359232 | 2.03E-13 | -1.535375 | 9.22E-10 |
| KRT222 | 1.9038227 | 6.25E-10 | 1.8165265 | 8.01E-08 |
| LGALS4 | 1.856444 | 1.93E-09 | 1.1359485 | 0.0007369 |
| LIF | -1.526506 | 3.74E-06 | -1.336193 | 0.0001261 |
| LIN28 | -1.186661 | 0.0152304 | -1.332434 | 0.0153229 |
| LIPN | -1.011676 | 5.29E-05 | -1.041133 | 0.0006971 |
| LOC154761 | -1.472695 | 5.05E-06 | -1.711953 | 2.25E-06 |
| LOC440570 | -1.014154 | 0.0215352 | -1.109281 | 0.0198998 |
| LOC55908 | 1.6701266 | 2.42E-10 | 1.6248873 | 1.75E-08 |
| LOC650293 | -1.064078 | 0.0238764 | -1.131555 | 0.0232745 |
| LOC730101 | 1.3010979 | 4.45E-07 | 1.5353616 | 5.22E-07 |
| LOC730417 | 2.5193406 | 1.23E-10 | 2.5363637 | 3.80E-08 |
| LONRF2 | -1.064574 | 3.01E-06 | -1.169999 | 1.52E-06 |
| LRIG3 | 1.1779233 | 9.19E-05 | 1.2055531 | 0.0002286 |
| LRRC19 | 1.1430489 | 0.0066291 | 1.0840847 | 0.0193877 |
| LRRC31 | 1.1761527 | 9.36E-06 | 1.0284759 | 0.0003762 |
| MAFF | -1.324332 | 6.34E-09 | -1.259767 | 2.36E-07 |
| MAP3K8 | -1.574184 | 1.72E-14 | -1.38376 | 3.02E-09 |
| MAPK4 | -1.083023 | 0.002453 | -1.12767 | 0.003289 |
| MCAM | -1.162148 | 3.15E-05 | -1.352612 | 2.69E-05 |
| MGC12965 | 1.0266808 | 3.32E-06 | 1.1892407 | 8.16E-07 |
| MGC13057 | 1.0467858 | 1.10E-06 | 1.2303942 | 2.06E-06 |
| MIR146B | -1.974962 | 1.82E-07 | -1.910877 | 6.98E-06 |
| MIR21 | -2.114411 | 5.87E-22 | -2.027998 | 5.85E-13 |
| MIR221 | -1.119222 | 0.00667 | -1.38997 | 0.0006664 |
| MIR548I1 | -1.027398 | 0.0429641 | -1.07396 | 0.039768 |
| MIR564 | 1.118879 | 2.62E-07 | 1.1878917 | 3.96E-07 |
| MIR886 | -1.341299 | 1.22E-05 | -1.139657 | 0.0012858 |
| MMP19 | -1.422107 | 6.22E-07 | -1.382308 | 2.23E-05 |
| MOGAT2 | 1.3610252 | 3.85E-08 | 1.4041763 | 2.49E-07 |
| MT1A | -2.339208 | 1.73E-07 | -1.213691 | 0.0025016 |
| MTMR4 | 1.0364623 | 5.95E-06 | 1.1091108 | 4.79E-06 |
| MYBPH | -1.644337 | 4.35E-06 | -1.624825 | 2.07E-05 |
| MYC | -2.552602 | 1.54E-18 | -2.687602 | 1.42E-12 |
| MYH11 | -1.527871 | 7.04E-05 | -1.229766 | 0.0032118 |
| NAGS | 1.3844332 | 1.30E-06 | 1.7595357 | 4.43E-08 |
| NAT8B | 1.8962613 | 3.54E-11 | 1.9840222 | 9.41E-10 |
| NCOA7 | -1.04139 | 4.41E-07 | -1.016229 | 5.63E-06 |
| NFE2 | -1.735721 | 4.11E-08 | -1.132532 | 0.0009397 |
| NINL | 1.2954779 | 1.10E-06 | 1.3338088 | 1.24E-05 |
| NLF2 | -1.688947 | 5.71E-08 | -1.520777 | 3.17E-06 |
| NOL4 | 1.1011086 | 7.61E-05 | 1.1553351 | 2.23E-05 |
| NR4A1 | -1.825346 | 5.82E-09 | -1.513552 | 8.84E-06 |
| NR4A2 | -1.629771 | 3.09E-08 | -1.425567 | 7.05E-06 |
| NR4A3 | -1.034556 | 1.93E-06 | -1.033656 | 1.23E-05 |
| ODF3L1 | -1.319643 | 4.51E-06 | -1.318379 | 9.60E-05 |
| ODZ3 | -1.644979 | 2.86E-08 | -1.153991 | 2.44E-05 |
| OR4F21 | -1.064765 | 0.0473129 | -1.105836 | 0.0355433 |
| OR4F5 | -1.074586 | 0.0291288 | -1.000041 | 0.0382308 |
| ORAI3 | 1.3229217 | 1.57E-05 | 1.5240488 | 2.66E-05 |
| OXCT2 | -1.062646 | 0.0001707 | -1.01403 | 0.0002361 |
| P4HA1 | -1.403909 | 1.61E-10 | -1.344454 | 2.88E-08 |
| PADI4 | -1.632188 | 9.57E-09 | -1.341863 | 1.62E-05 |
| PAPPA | -1.089406 | 0.0004434 | -1.195287 | 0.0004015 |
| PAQR7 | 1.0938947 | 1.97E-07 | 1.0638573 | 3.21E-05 |
| PCDH24 | 1.4201704 | 6.64E-06 | 1.198956 | 0.0003413 |
| PEG10 | 2.1470397 | 1.34E-11 | 1.1244234 | 9.91E-05 |
| PFKFB3 | -1.126492 | 8.54E-10 | -1.168503 | 7.39E-08 |
| PHLDA1 | -1.769441 | 8.14E-12 | -1.999034 | 1.73E-11 |
| PIM1 | -2.030194 | 6.06E-16 | -1.951805 | 3.30E-09 |
| PIM3 | -1.422234 | 2.59E-09 | -1.316661 | 1.13E-06 |
| PJCG6 | -1.060741 | 0.0153806 | -1.02136 | 0.0268096 |
| PLAUR | -1.308814 | 5.75E-09 | -1.435837 | 1.11E-07 |
| PNRC1 | -2.10224 | 5.76E-13 | -2.009058 | 2.11E-08 |
| POM121L4P | -1.069382 | 0.0344727 | -1.17862 | 0.0318492 |
| PPAN | -1.040454 | 4.42E-07 | -1.018575 | 9.38E-06 |
| PPP1R15A | -1.743611 | 8.93E-18 | -1.704133 | 2.18E-10 |
| PPRC1 | -1.48641 | 5.57E-15 | -1.376768 | 2.97E-10 |
| PROK2 | -1.976481 | 2.03E-08 | -1.766787 | 9.52E-06 |
| PRSS3 | 1.4137104 | 3.65E-08 | 1.3641352 | 2.38E-05 |
| PTGS2 | -1.95827 | 2.45E-10 | -1.752805 | 1.40E-07 |
| PTX3 | -1.76345 | 2.77E-05 | -1.822034 | 3.83E-05 |
| RAB26 | 1.2167893 | 1.77E-08 | 1.2418407 | 2.36E-08 |
| RALGDS | -1.549682 | 2.95E-09 | -1.550468 | 4.77E-08 |
| RASD1 | -2.079214 | 6.12E-06 | -2.106032 | 3.53E-05 |
| RBP5 | 1.0796805 | 6.40E-05 | 1.1380454 | 0.0001742 |
| RFXDC2 | 1.0258028 | 2.15E-13 | 1.0452957 | 2.23E-10 |
| RGS1 | -1.348276 | 1.18E-12 | -1.363185 | 6.69E-08 |
| RGS16 | -1.197856 | 0.0004286 | -1.731325 | 5.86E-06 |
| RGS2 | -1.731358 | 5.53E-12 | -1.298862 | 2.47E-08 |
| RMND1 | 1.0727278 | 1.79E-08 | 1.0795254 | 2.97E-07 |
| RND1 | -2.223263 | 6.70E-08 | -1.656618 | 0.0001334 |
| RNF43 | 1.8322335 | 1.23E-10 | 1.5689261 | 1.13E-08 |
| RRP12 | -1.299563 | 8.74E-07 | -1.308828 | 5.52E-06 |
| RRS1 | -1.578578 | 2.60E-08 | -1.749542 | 1.51E-08 |
| RTP3 | 1.9847581 | 6.58E-09 | 1.8522452 | 1.01E-06 |
| RTP4 | 1.4226228 | 6.86E-08 | 1.2095448 | 1.75E-05 |
| S100A12 | -2.136697 | 2.00E-07 | -1.726442 | 0.0001036 |
| S100A8 | -1.574803 | 1.47E-05 | -1.17348 | 0.0021746 |
| S100P | -2.040674 | 5.40E-10 | -1.614985 | 3.17E-06 |
| SAA1 | -1.051765 | 0.0123684 | -1.211052 | 0.0051952 |
| SBK2 | -1.020318 | 0.0276691 | -1.097246 | 0.0370501 |
| SBNO2 | -1.00457 | 8.81E-05 | -1.029952 | 0.0001197 |
| SDC4 | -1.394619 | 4.73E-09 | -1.371323 | 1.98E-08 |
| SERPINB9 | -1.26975 | 3.90E-06 | -1.1689 | 1.25E-05 |
| SERPINE1 | -1.51368 | 1.60E-07 | -1.711799 | 3.44E-07 |
| SERTAD1 | -1.174381 | 4.62E-12 | -1.056269 | 4.38E-08 |
| SHD | 1.0303264 | 0.0037181 | 1.416794 | 0.0003467 |
| SIK1 | -1.346219 | 2.40E-10 | -1.286963 | 1.67E-07 |
| SIPA1L2 | -1.380587 | 6.80E-09 | -1.161856 | 2.21E-07 |
| SIRT4 | 1.0647165 | 2.70E-07 | 1.053381 | 4.73E-06 |
| SLC11A1 | -1.098596 | 1.69E-07 | -1.016083 | 4.66E-06 |
| SLC12A1 | 1.6858516 | 2.59E-07 | 1.0506005 | 7.29E-05 |
| SLC25A34 | 1.8085029 | 6.25E-08 | 1.828133 | 1.09E-06 |
| SLC2A3 | -1.504427 | 3.15E-09 | -1.550212 | 5.07E-08 |
| SLC7A1 | -1.542948 | 0.000113 | -1.794472 | 9.73E-06 |
| SLITRK3 | -2.897076 | 2.53E-11 | -2.486682 | 1.92E-09 |
| SOCS1 | -2.055275 | 2.47E-08 | -1.917274 | 1.66E-06 |
| SOCS2 | -2.048604 | 1.36E-15 | -1.744176 | 3.84E-12 |
| SOCS3 | -1.86949 | 1.19E-14 | -1.674212 | 4.23E-09 |
| SOX17 | -1.111235 | 3.65E-05 | -1.330685 | 4.16E-05 |
| SPHK2 | 1.3009429 | 3.81E-05 | 1.1253758 | 0.0012621 |
| SPSB1 | -1.656556 | 6.11E-07 | -2.178721 | 6.78E-08 |
| STC1 | -1.155819 | 1.66E-06 | -1.364148 | 3.01E-06 |
| TBX10 | 1.355177 | 4.33E-10 | 1.2265291 | 2.30E-07 |
| TBX3 | 1.0380265 | 7.31E-07 | 1.0818093 | 2.66E-06 |
| TCAP | 1.2177907 | 6.76E-05 | 1.363648 | 4.62E-05 |
| TGFB3 | -1.775605 | 1.76E-06 | -1.96693 | 4.97E-06 |
| THBD | -1.754805 | 9.61E-09 | -1.979727 | 3.42E-07 |
| THBS1 | -1.807104 | 5.82E-12 | -2.15763 | 5.42E-12 |
| TMEM169 | 1.4929445 | 1.52E-11 | 1.465312 | 5.32E-09 |
| TMEM233 | -1.132073 | 6.32E-06 | -1.137305 | 0.0001501 |
| TMEM98 | 1.00871 | 1.46E-05 | 1.0409823 | 5.05E-05 |
| TMPRSS2 | -1.173925 | 9.17E-05 | -1.370308 | 4.91E-05 |
| TNFAIP6 | -1.239312 | 2.06E-05 | -1.30793 | 1.39E-05 |
| TNFAIP8L3 | -1.110707 | 2.45E-05 | -1.084976 | 5.32E-05 |
| TNFRSF10D | -1.427539 | 3.74E-06 | -1.2956 | 2.70E-05 |
| TNFRSF12A | -1.829065 | 4.65E-06 | -2.352102 | 4.81E-08 |
| TNFSF14 | -1.333351 | 2.36E-07 | -1.209441 | 7.97E-06 |
| TRHDE | 1.1783732 | 1.91E-05 | 1.1449604 | 0.0001248 |
| TRIM15 | -1.322187 | 6.07E-07 | -1.154274 | 1.29E-05 |
| TUBB6 | -1.155135 | 2.45E-07 | -1.145524 | 3.18E-06 |
| URB2 | -1.265528 | 1.19E-06 | -1.120722 | 4.07E-05 |
| VCAN | -1.025144 | 0.000317 | -1.554204 | 8.12E-06 |
| VNN3 | -1.243194 | 2.09E-07 | -1.339865 | 2.44E-07 |
| WNT5A | 1.5762753 | 5.72E-13 | 1.5386125 | 9.36E-10 |
| ZC3H12A | -2.346699 | 4.86E-11 | -2.137855 | 6.40E-08 |
| ZFP36 | -1.715592 | 2.00E-09 | -1.531053 | 4.27E-07 |
| ZNF295 | -1.433485 | 8.12E-06 | -1.247686 | 0.0002382 |
| ZNF878 | -1.440997 | 4.51E-07 | -1.373415 | 7.55E-06 |
