# Supplementary figures and images for: Identification of polyunsaturated fatty acids related key modules and genes in metabolic dysfunction-associated fatty liver disease using WGCNA analysis
Source: Front Genet. 2022 Nov 8;13:951224. doi: 10.3389/fgene.2022.951224 (PMC9679514; doi:10.3389/fgene.2022.951224)

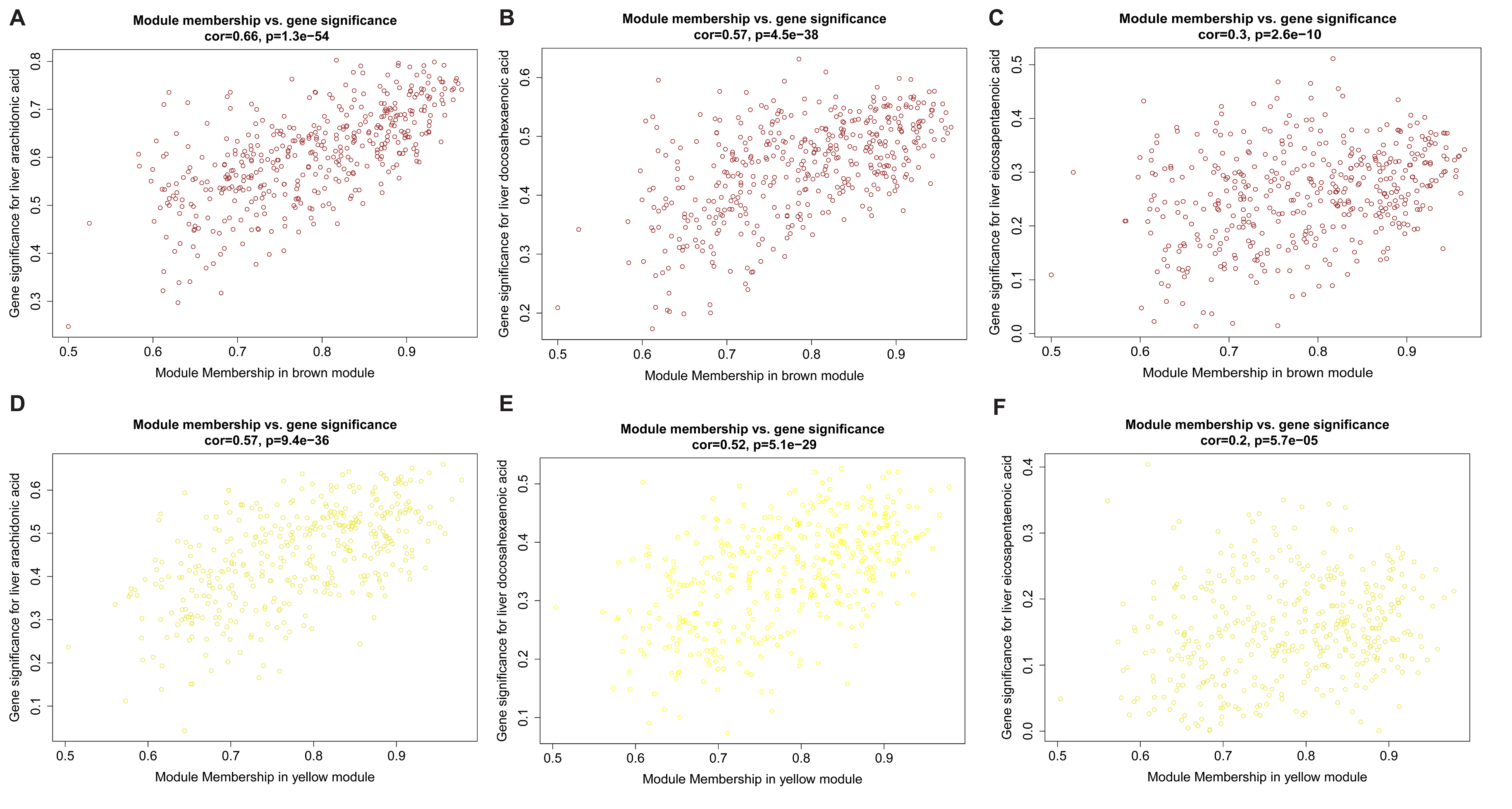

Supplement: Supplementary file 2 [file Image3.TIF]

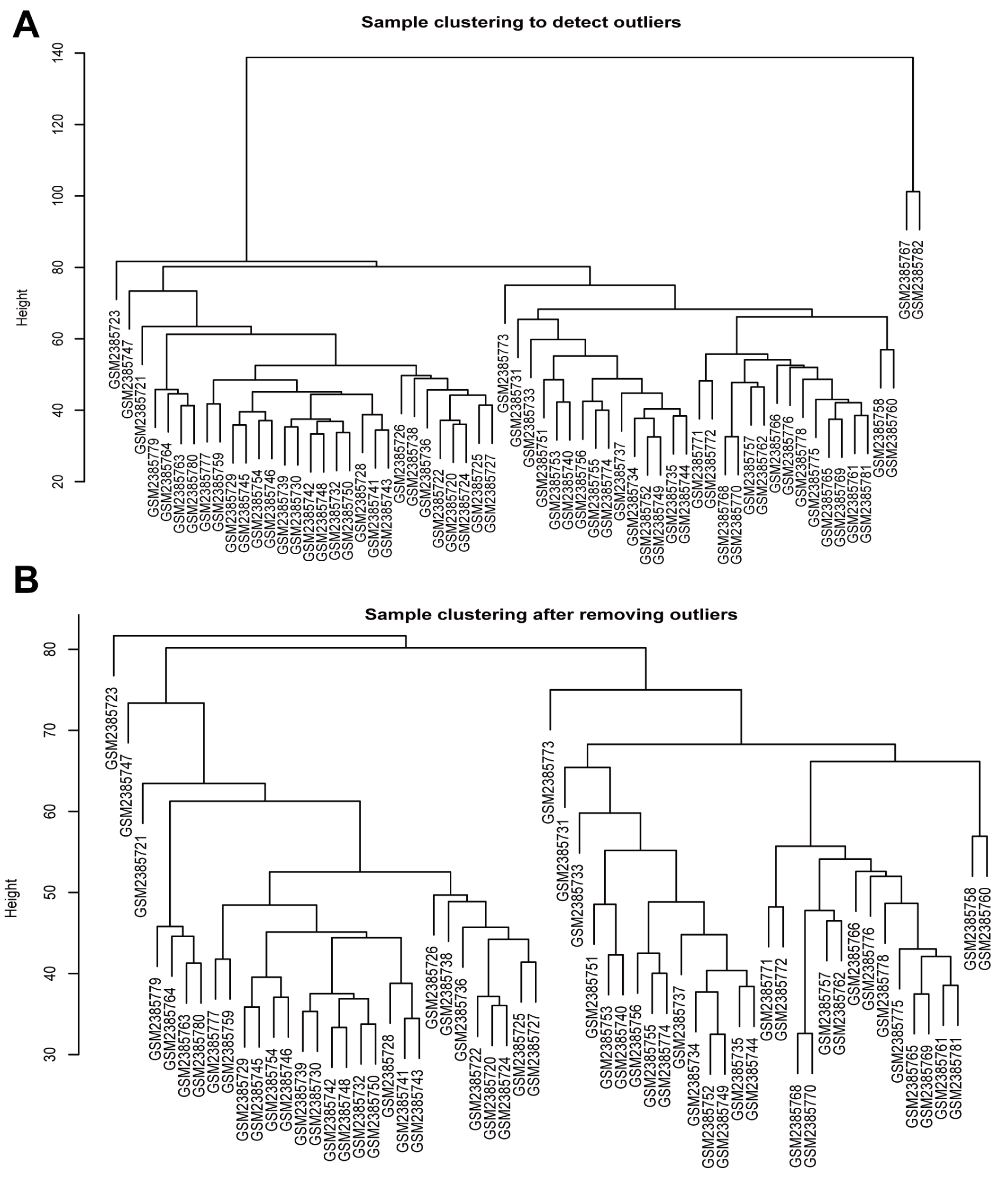

Supplement: Supplementary file 3 [file Image2.TIF]

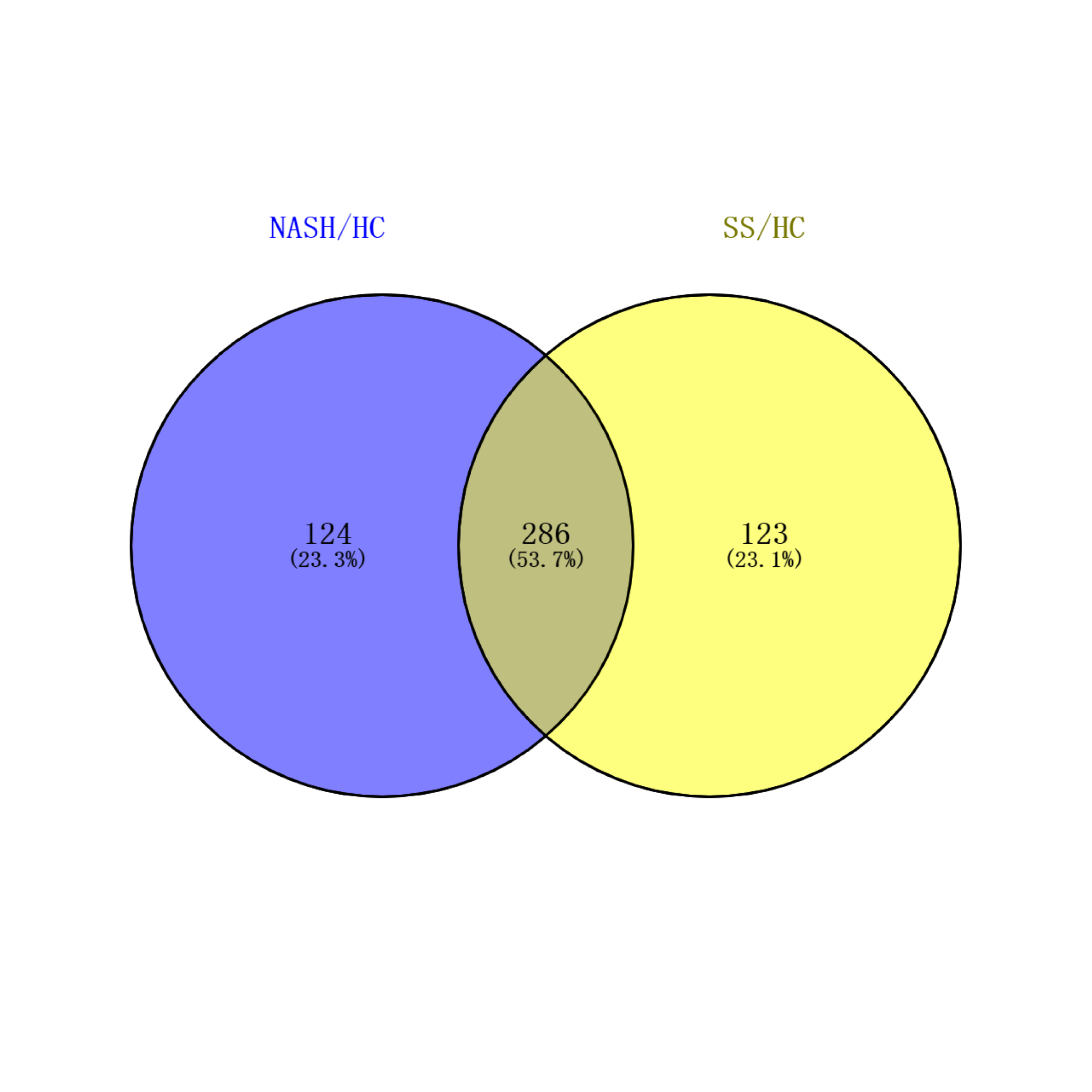

Supplement: Supplementary file 4 [file Image1.TIF]
