# Supplementary material for: Identification of polyunsaturated fatty acids related key modules and genes in metabolic dysfunction-associated fatty liver disease using WGCNA analysis
Source: Front Genet. 2022 Nov 8;13:951224. doi: 10.3389/fgene.2022.951224 (PMC9679514; doi:10.3389/fgene.2022.951224)
Supplement: Supplementary file 5 [file Table2.DOCX]

| **Table S2. GO and KEGG pathway functional enrichment analysis for common DEGs** | | | | | | | | | |  |
| --- | --- | --- | --- | --- | --- | --- | --- | --- | --- | --- |
| **Categary** | **ID** | **Description** | **GeneRatio** | **BgRatio** | **P value** | **p.adjust** | **qvalue** | **geneID** | **Count** | |
| BP | GO:0032496 | response to lipopolysaccharide | 23/217 | 330/18670 | 6.50E-12 | 2.44E-08 | 1.76E-08 | C5AR1/CCL2/CCL3/CD274/DEFA1/FMO1/FOS/IL1B/IL1RN/IL6/IRAK3/JUN/JUNB/MIR21/PTGS2/S100A8/SBNO2/SERPINE1/SLC11A1/THBD/WNT5A/ZC3H12A/ZFP36 | 23 | |
| BP | GO:0002237 | response to molecule of bacterial origin | 23/217 | 343/18670 | 1.43E-11 | 2.69E-08 | 1.94E-08 | C5AR1/CCL2/CCL3/CD274/DEFA1/FMO1/FOS/IL1B/IL1RN/IL6/IRAK3/JUN/JUNB/MIR21/PTGS2/S100A8/SBNO2/SERPINE1/SLC11A1/THBD/WNT5A/ZC3H12A/ZFP36 | 23 | |
| BP | GO:0044706 | multi-multicellular organism process | 17/217 | 222/18670 | 9.78E-10 | 7.89E-07 | 5.69E-07 | AVPR1A/FOS/FOSB/FOSL1/IGFBP2/IL1B/JUNB/LIF/MAFF/MIR21/PAPPA/PTGS2/RGS2/SPHK2/STC1/TGFB3/THBD | 17 | |
| BP | GO:0045444 | fat cell differentiation | 17/217 | 223/18670 | 1.05E-09 | 7.89E-07 | 5.69E-07 | ARID5B/CEBPA/CEBPD/FOXO1/IL6/KLF4/KLF5/MIR21/NR4A1/NR4A2/NR4A3/PTGS2/RGS2/SOCS1/WNT5A/ZC3H12A/ZFP36 | 17 | |
| BP | GO:0042116 | macrophage activation | 12/217 | 95/18670 | 1.05E-09 | 7.89E-07 | 5.69E-07 | C5AR1/CCL3/CEBPA/IL1RL1/IL4R/IL6/JUN/SBNO2/SLC11A1/THBS1/WNT5A/ZC3H12A | 12 | |
| BP | GO:0030728 | ovulation | 7/217 | 21/18670 | 2.63E-09 | 1.60E-06 | 1.15E-06 | ADAMTS1/AFP/IL4R/MMP19/PTGS2/RGS2/TNFAIP6 | 7 | |
| BP | GO:0060326 | cell chemotaxis | 19/217 | 304/18670 | 2.98E-09 | 1.60E-06 | 1.15E-06 | C5AR1/CCL2/CCL20/CCL3/CH25H/DEFA1/EPHA2/HBEGF/IL1B/IL1RN/IL6/NR4A1/S100A12/S100A8/SAA1/SERPINE1/THBS1/TNFSF14/WNT5A | 19 | |
| BP | GO:0048545 | response to steroid hormone | 21/217 | 385/18670 | 4.85E-09 | 2.28E-06 | 1.64E-06 | AVPR1A/DEFA1/FAM107A/FOS/FOSB/FOSL1/FOXO1/GPAM/IGFBP2/IL1RN/IL6/NR4A1/NR4A2/NR4A3/PAPPA/PAQR7/PTGS2/STC1/TGFB3/THBS1/ZFP36 | 21 | |
| BP | GO:0007565 | female pregnancy | 15/217 | 192/18670 | 7.44E-09 | 2.87E-06 | 2.07E-06 | FOS/FOSB/FOSL1/IGFBP2/IL1B/JUNB/LIF/MIR21/PAPPA/PTGS2/RGS2/SPHK2/STC1/TGFB3/THBD | 15 | |
| BP | GO:0032103 | positive regulation of response to external stimulus | 19/217 | 323/18670 | 8.04E-09 | 2.87E-06 | 2.07E-06 | C2CD4A/C2CD4B/C5AR1/CCL3/CEBPA/IL1B/IL1RL1/IL6/MIR21/MIR221/PTGS2/S100A12/S100A8/SERPINE1/THBD/THBS1/TNFSF14/TRIM15/WNT5A | 19 | |
| BP | GO:0030595 | leukocyte chemotaxis | 16/217 | 224/18670 | 8.41E-09 | 2.87E-06 | 2.07E-06 | C5AR1/CCL2/CCL20/CCL3/CH25H/DEFA1/IL1B/IL1RN/IL6/S100A12/S100A8/SAA1/SERPINE1/THBS1/TNFSF14/WNT5A | 16 | |
| BP | GO:0050727 | regulation of inflammatory response | 23/217 | 485/18670 | 1.22E-08 | 3.83E-06 | 2.76E-06 | BCL6/BIRC3/C2CD4A/C2CD4B/C5AR1/CCL3/CEBPA/IL1B/IL1RL1/IL6/KLF4/MIR21/MIR221/PTGS2/S100A12/S100A8/SAA1/SBNO2/SERPINE1/SOCS3/TNFAIP6/WNT5A/ZC3H12A | 23 | |
| BP | GO:0051384 | response to glucocorticoid | 13/217 | 146/18670 | 1.61E-08 | 4.64E-06 | 3.35E-06 | AVPR1A/FAM107A/FOS/FOSB/FOSL1/FOXO1/IGFBP2/IL1RN/IL6/PAPPA/PTGS2/STC1/ZFP36 | 13 | |
| BP | GO:0002696 | positive regulation of leukocyte activation | 20/217 | 380/18670 | 2.09E-08 | 5.60E-06 | 4.04E-06 | BCL6/CCL2/CCL3/CD274/CEBPA/CLCF1/IGFBP2/IL1B/IL1RL1/IL4R/IL6/IRS2/MAP3K8/MIR21/NR4A3/SOCS1/SPHK2/THBS1/TNFSF14/WNT5A | 20 | |
| BP | GO:0050729 | positive regulation of inflammatory response | 13/217 | 153/18670 | 2.82E-08 | 7.07E-06 | 5.10E-06 | C2CD4A/C2CD4B/CCL3/CEBPA/IL1B/IL1RL1/IL6/MIR21/PTGS2/S100A12/S100A8/SERPINE1/WNT5A | 13 | |
| BP | GO:0050867 | positive regulation of cell activation | 20/217 | 394/18670 | 3.80E-08 | 8.92E-06 | 6.43E-06 | BCL6/CCL2/CCL3/CD274/CEBPA/CLCF1/IGFBP2/IL1B/IL1RL1/IL4R/IL6/IRS2/MAP3K8/MIR21/NR4A3/SOCS1/SPHK2/THBS1/TNFSF14/WNT5A | 20 | |
| BP | GO:0031960 | response to corticosteroid | 13/217 | 162/18670 | 5.58E-08 | 1.23E-05 | 8.89E-06 | AVPR1A/FAM107A/FOS/FOSB/FOSL1/FOXO1/IGFBP2/IL1RN/IL6/PAPPA/PTGS2/STC1/ZFP36 | 13 | |
| BP | GO:0007159 | leukocyte cell-cell adhesion | 18/217 | 337/18670 | 8.77E-08 | 1.83E-05 | 1.32E-05 | BCL6/CCL2/CD274/HAS2/IGFBP2/IL1B/IL4R/IL6/KLF4/MAP3K8/MIR21/MIR221/NR4A3/S100A8/SDC4/SOCS1/TNFSF14/ZC3H12A | 18 | |
| BP | GO:1903037 | regulation of leukocyte cell-cell adhesion | 17/217 | 304/18670 | 1.05E-07 | 2.08E-05 | 1.50E-05 | BCL6/CCL2/CD274/HAS2/IGFBP2/IL1B/IL4R/IL6/KLF4/MAP3K8/MIR21/MIR221/NR4A3/SDC4/SOCS1/TNFSF14/ZC3H12A | 17 | |
| BP | GO:0043030 | regulation of macrophage activation | 8/217 | 55/18670 | 2.23E-07 | 4.19E-05 | 3.02E-05 | CCL3/CEBPA/IL1RL1/IL4R/IL6/THBS1/WNT5A/ZC3H12A | 8 | |
| BP | GO:0022407 | regulation of cell-cell adhesion | 19/217 | 403/18670 | 2.65E-07 | 4.75E-05 | 3.42E-05 | BCL6/CCL2/CD274/HAS2/IGFBP2/IL1B/IL1RN/IL4R/IL6/KLF4/MAP3K8/MIR21/MIR221/NR4A3/SDC4/SOCS1/TNFSF14/WNT5A/ZC3H12A | 19 | |
| BP | GO:0002367 | cytokine production involved in immune response | 10/217 | 102/18670 | 3.07E-07 | 5.24E-05 | 3.78E-05 | BCL6/IL18RAP/IL1B/IL6/IRAK3/NR4A3/SLC11A1/SPHK2/TGFB3/WNT5A | 10 | |
| BP | GO:0019216 | regulation of lipid metabolic process | 19/217 | 410/18670 | 3.45E-07 | 5.64E-05 | 4.07E-05 | AVPR1A/CISH/CYP7A1/EGR1/FADS1/GPAM/IL1B/IRS2/KLF4/MTMR4/NR4A3/PTGS2/SIK1/SIRT4/SOCS1/SOCS2/SOCS3/SPHK2/TNFAIP8L3 | 19 | |
| BP | GO:0001819 | positive regulation of cytokine production | 20/217 | 464/18670 | 5.27E-07 | 7.61E-05 | 5.49E-05 | BCL3/BIRC3/C5AR1/CCL3/CD274/EGR1/IL1B/IL1RL1/IL1RN/IL4R/IL6/MIR21/NR4A3/PTGS2/SAA1/SERPINE1/SLC11A1/THBS1/TRIM15/WNT5A | 20 | |
| BP | GO:1901342 | regulation of vasculature development | 19/217 | 422/18670 | 5.35E-07 | 7.61E-05 | 5.49E-05 | AMOT/C5AR1/EGR1/EPHA1/EPHA2/FOXC1/IL1B/IL6/ISM1/KLF4/MIR21/MIR221/PROK2/PTGS2/SERPINE1/THBS1/TNFRSF12A/WNT5A/ZC3H12A | 19 | |
| BP | GO:0007292 | female gamete generation | 11/217 | 136/18670 | 5.55E-07 | 7.61E-05 | 5.49E-05 | ADAMTS1/AFP/ASPM/FOSL1/IL4R/MMP19/PAQR7/PTGS2/PTX3/RGS2/TNFAIP6 | 11 | |
| BP | GO:0043032 | positive regulation of macrophage activation | 6/217 | 27/18670 | 5.55E-07 | 7.61E-05 | 5.49E-05 | CCL3/CEBPA/IL1RL1/IL4R/THBS1/WNT5A | 6 | |
| BP | GO:0045765 | regulation of angiogenesis | 18/217 | 383/18670 | 5.80E-07 | 7.61E-05 | 5.49E-05 | AMOT/C5AR1/EPHA1/EPHA2/FOXC1/IL1B/IL6/ISM1/KLF4/MIR21/MIR221/PROK2/PTGS2/SERPINE1/THBS1/TNFRSF12A/WNT5A/ZC3H12A | 18 | |
| BP | GO:0042088 | T-helper 1 type immune response | 7/217 | 43/18670 | 5.88E-07 | 7.61E-05 | 5.49E-05 | BCL3/IL18RAP/IL1B/IL1RL1/IL4R/SLC11A1/TMEM98 | 7 | |
| BP | GO:0048660 | regulation of smooth muscle cell proliferation | 12/217 | 169/18670 | 6.88E-07 | 8.61E-05 | 6.21E-05 | ADAMTS1/CNN1/HBEGF/IL6/JUN/KLF4/MIR21/MIR221/NR4A3/PTGS2/TGFB3/THBS1 | 12 | |
| BP | GO:0048247 | lymphocyte chemotaxis | 8/217 | 64/18670 | 7.41E-07 | 8.98E-05 | 6.48E-05 | CCL2/CCL20/CCL3/CH25H/DEFA1/SAA1/TNFSF14/WNT5A | 8 | |
| BP | GO:0048659 | smooth muscle cell proliferation | 12/217 | 171/18670 | 7.80E-07 | 9.16E-05 | 6.60E-05 | ADAMTS1/CNN1/HBEGF/IL6/JUN/KLF4/MIR21/MIR221/NR4A3/PTGS2/TGFB3/THBS1 | 12 | |
| BP | GO:0033002 | muscle cell proliferation | 14/217 | 239/18670 | 8.35E-07 | 9.43E-05 | 6.80E-05 | ADAMTS1/CNN1/FOXC1/HBEGF/IL6/JUN/KLF4/MIR21/MIR221/NR4A3/PIM1/PTGS2/TGFB3/THBS1 | 14 | |
| BP | GO:0071222 | cellular response to lipopolysaccharide | 13/217 | 205/18670 | 8.54E-07 | 9.43E-05 | 6.80E-05 | CCL2/CCL3/CD274/DEFA1/IL1B/IL1RN/IL6/MIR21/SBNO2/SERPINE1/WNT5A/ZC3H12A/ZFP36 | 13 | |
| BP | GO:0032602 | chemokine production | 9/217 | 89/18670 | 9.32E-07 | 0.000100064 | 7.22E-05 | EGR1/EPHA2/IL1B/IL1RL1/IL4R/IL6/KLF4/S100A8/WNT5A | 9 | |
| BP | GO:0090130 | tissue migration | 17/217 | 360/18670 | 1.13E-06 | 0.000118119 | 8.52E-05 | ACTG2/AMOT/EPHA2/HAS2/HBEGF/JUN/KLF4/MIR21/MIR221/NR4A1/PRSS3/PTGS2/S100P/STC1/THBS1/WNT5A/ZC3H12A | 17 | |
| BP | GO:0035710 | CD4-positive, alpha-beta T cell activation | 9/217 | 92/18670 | 1.24E-06 | 0.000123217 | 8.89E-05 | BCL3/BCL6/CD274/IL4R/IL6/MIR21/SOCS1/TMEM98/ZC3H12A | 9 | |
| BP | GO:0071219 | cellular response to molecule of bacterial origin | 13/217 | 212/18670 | 1.25E-06 | 0.000123217 | 8.89E-05 | CCL2/CCL3/CD274/DEFA1/IL1B/IL1RN/IL6/MIR21/SBNO2/SERPINE1/WNT5A/ZC3H12A/ZFP36 | 13 | |
| BP | GO:1903039 | positive regulation of leukocyte cell-cell adhesion | 13/217 | 218/18670 | 1.70E-06 | 0.000161175 | 0.000116251 | BCL6/CCL2/CD274/HAS2/IGFBP2/IL1B/IL4R/IL6/MAP3K8/MIR21/NR4A3/SOCS1/TNFSF14 | 13 | |
| BP | GO:0071621 | granulocyte chemotaxis | 10/217 | 123/18670 | 1.75E-06 | 0.000161175 | 0.000116251 | C5AR1/CCL2/CCL20/CCL3/IL1B/IL1RN/S100A12/S100A8/SAA1/THBS1 | 10 | |
| BP | GO:0007369 | gastrulation | 12/217 | 185/18670 | 1.79E-06 | 0.000161175 | 0.000116251 | AMOT/EPHA2/ETS2/FOXC1/IL1RN/KLF4/MIR221/NAT8B/NR4A3/SOX17/TRIM15/WNT5A | 12 | |
| BP | GO:0022409 | positive regulation of cell-cell adhesion | 14/217 | 255/18670 | 1.80E-06 | 0.000161175 | 0.000116251 | BCL6/CCL2/CD274/HAS2/IGFBP2/IL1B/IL4R/IL6/MAP3K8/MIR21/NR4A3/SOCS1/TNFSF14/WNT5A | 14 | |
| BP | GO:0032147 | activation of protein kinase activity | 16/217 | 333/18670 | 1.89E-06 | 0.000164888 | 0.000118929 | C5AR1/FPR1/GADD45B/GADD45G/GPRC5A/IL1B/MAP3K8/MIR21/PROK2/SAA1/SBK2/SLC11A1/SOCS1/TGFB3/THBS1/WNT5A | 16 | |
| BP | GO:0001667 | ameboidal-type cell migration | 19/217 | 461/18670 | 1.99E-06 | 0.000170266 | 0.000122807 | AMOT/ARID5B/EPHA2/HAS2/HBEGF/JUN/KLF4/MIR21/MIR221/NR4A1/PRSS3/PTGS2/S100P/SDC4/SOX17/STC1/THBS1/WNT5A/ZC3H12A | 19 | |
| BP | GO:0001818 | negative regulation of cytokine production | 15/217 | 296/18670 | 2.08E-06 | 0.000173426 | 0.000125087 | BCL3/BCL6/CD274/EPHA2/IL1RL1/IL6/IRAK3/KLF4/MIR21/MIR221/SLC11A1/TGFB3/THBS1/ZC3H12A/ZFP36 | 15 | |
| BP | GO:0043367 | CD4-positive, alpha-beta T cell differentiation | 8/217 | 74/18670 | 2.28E-06 | 0.000186568 | 0.000134566 | BCL3/BCL6/IL4R/IL6/MIR21/SOCS1/TMEM98/ZC3H12A | 8 | |
| BP | GO:0048661 | positive regulation of smooth muscle cell proliferation | 9/217 | 101/18670 | 2.71E-06 | 0.000217058 | 0.000156557 | ADAMTS1/HBEGF/IL6/JUN/MIR21/MIR221/NR4A3/PTGS2/THBS1 | 9 | |
| BP | GO:0048332 | mesoderm morphogenesis | 8/217 | 77/18670 | 3.09E-06 | 0.000242285 | 0.000174753 | EPHA2/FOXC1/KLF4/NR4A3/SOX17/TBX3/TRIM15/WNT5A | 8 | |
| BP | GO:0030593 | neutrophil chemotaxis | 9/217 | 104/18670 | 3.47E-06 | 0.0002658 | 0.000191713 | C5AR1/CCL2/CCL20/CCL3/IL1B/IL1RN/S100A12/S100A8/SAA1 | 9 | |
| BP | GO:0010631 | epithelial cell migration | 16/217 | 351/18670 | 3.71E-06 | 0.000279195 | 0.000201375 | AMOT/EPHA2/HAS2/HBEGF/JUN/KLF4/MIR21/MIR221/NR4A1/PRSS3/PTGS2/S100P/STC1/THBS1/WNT5A/ZC3H12A | 16 | |
| BP | GO:0046683 | response to organophosphorus | 10/217 | 134/18670 | 3.80E-06 | 0.000280292 | 0.000202166 | FOS/FOSB/FOSL1/IL1B/JUN/JUNB/MMP19/PTGS2/STC1/THBD | 10 | |
| BP | GO:0071216 | cellular response to biotic stimulus | 13/217 | 236/18670 | 4.09E-06 | 0.00029358 | 0.00021175 | CCL2/CCL3/CD274/DEFA1/IL1B/IL1RN/IL6/MIR21/SBNO2/SERPINE1/WNT5A/ZC3H12A/ZFP36 | 13 | |
| BP | GO:0090132 | epithelium migration | 16/217 | 354/18670 | 4.14E-06 | 0.00029358 | 0.00021175 | AMOT/EPHA2/HAS2/HBEGF/JUN/KLF4/MIR21/MIR221/NR4A1/PRSS3/PTGS2/S100P/STC1/THBS1/WNT5A/ZC3H12A | 16 | |
| BP | GO:0042093 | T-helper cell differentiation | 7/217 | 58/18670 | 4.73E-06 | 0.000329142 | 0.0002374 | BCL3/BCL6/IL4R/IL6/MIR21/TMEM98/ZC3H12A | 7 | |
| BP | GO:0032642 | regulation of chemokine production | 8/217 | 82/18670 | 4.99E-06 | 0.000340785 | 0.000245797 | EGR1/EPHA2/IL1B/IL1RL1/IL4R/IL6/KLF4/WNT5A | 8 | |
| BP | GO:0045785 | positive regulation of cell adhesion | 17/217 | 403/18670 | 5.17E-06 | 0.000347254 | 0.000250463 | BCL6/CCL2/CD274/EPHA1/HAS2/IGFBP2/IL1B/IL4R/IL6/MAP3K8/MIR21/NR4A3/SAA1/SDC4/SOCS1/TNFSF14/WNT5A | 17 | |
| BP | GO:0002294 | CD4-positive, alpha-beta T cell differentiation involved in immune response | 7/217 | 60/18670 | 5.96E-06 | 0.000388914 | 0.000280511 | BCL3/BCL6/IL4R/IL6/MIR21/TMEM98/ZC3H12A | 7 | |
| BP | GO:0097530 | granulocyte migration | 10/217 | 141/18670 | 6.00E-06 | 0.000388914 | 0.000280511 | C5AR1/CCL2/CCL20/CCL3/IL1B/IL1RN/S100A12/S100A8/SAA1/THBS1 | 10 | |
| BP | GO:0050900 | leukocyte migration | 19/217 | 499/18670 | 6.27E-06 | 0.000399235 | 0.000287956 | C5AR1/CCL2/CCL20/CCL3/CH25H/DEFA1/IL1B/IL1RN/IL6/S100A12/S100A8/SAA1/SDC4/SERPINE1/THBD/THBS1/TNFRSF10D/TNFSF14/WNT5A | 19 | |
| BP | GO:0097529 | myeloid leukocyte migration | 12/217 | 210/18670 | 6.65E-06 | 0.000403863 | 0.000291293 | C5AR1/CCL2/CCL20/CCL3/IL1B/IL1RN/IL6/S100A12/S100A8/SAA1/SERPINE1/THBS1 | 12 | |
| BP | GO:0002287 | alpha-beta T cell activation involved in immune response | 7/217 | 61/18670 | 6.66E-06 | 0.000403863 | 0.000291293 | BCL3/BCL6/IL4R/IL6/MIR21/TMEM98/ZC3H12A | 7 | |
| BP | GO:0002293 | alpha-beta T cell differentiation involved in immune response | 7/217 | 61/18670 | 6.66E-06 | 0.000403863 | 0.000291293 | BCL3/BCL6/IL4R/IL6/MIR21/TMEM98/ZC3H12A | 7 | |
| BP | GO:0007162 | negative regulation of cell adhesion | 14/217 | 289/18670 | 7.67E-06 | 0.000451951 | 0.000325978 | BCL6/CD274/FAM107A/IL1RN/IL4R/KLF4/MIR21/MIR221/RND1/SDC4/SERPINE1/SOCS1/THBS1/ZC3H12A | 14 | |
| BP | GO:0002822 | regulation of adaptive immune response based on somatic recombination of immune receptors built from immunoglobulin superfamily domains | 10/217 | 145/18670 | 7.70E-06 | 0.000451951 | 0.000325978 | BCL6/CD274/CLCF1/IL1B/IL1RL1/IL4R/IL6/MIR21/SLC11A1/ZC3H12A | 10 | |
| BP | GO:0071347 | cellular response to interleukin-1 | 11/217 | 179/18670 | 8.16E-06 | 0.000465073 | 0.000335443 | CCL2/CCL20/CCL3/EGR1/HAS2/IL1B/IL1RN/IL6/IRAK3/MAP3K8/ZC3H12A | 11 | |
| BP | GO:0070486 | leukocyte aggregation | 4/217 | 12/18670 | 8.17E-06 | 0.000465073 | 0.000335443 | HAS2/IL1B/NR4A3/S100A8 | 4 | |
| BP | GO:0002683 | negative regulation of immune system process | 18/217 | 463/18670 | 8.36E-06 | 0.000468993 | 0.00033827 | BCL6/CCL2/CCL3/CD274/GRAMD4/IL1RL1/IL4R/IRAK3/MIR21/MIR221/MYC/SDC4/SERPINB9/SOCS1/TGFB3/THBS1/ZC3H12A/ZFP36 | 18 | |
| BP | GO:0042326 | negative regulation of phosphorylation | 18/217 | 468/18670 | 9.67E-06 | 0.000526249 | 0.000379567 | CEBPA/EPHA1/FOXO1/GADD45B/GPRC5A/IL1B/IRAK3/IRS2/JUN/KLF4/LIF/MIR21/MIR221/MYC/RGS2/SOCS1/SOCS3/ZC3H12A | 18 | |
| BP | GO:0014074 | response to purine-containing compound | 10/217 | 149/18670 | 9.79E-06 | 0.000526249 | 0.000379567 | FOS/FOSB/FOSL1/IL1B/JUN/JUNB/MMP19/PTGS2/STC1/THBD | 10 | |
| BP | GO:1990266 | neutrophil migration | 9/217 | 118/18670 | 9.80E-06 | 0.000526249 | 0.000379567 | C5AR1/CCL2/CCL20/CCL3/IL1B/IL1RN/S100A12/S100A8/SAA1 | 9 | |
| BP | GO:0071674 | mononuclear cell migration | 8/217 | 90/18670 | 1.00E-05 | 0.000530265 | 0.000382463 | C5AR1/CCL2/CCL20/CCL3/IL6/S100A12/SERPINE1/THBS1 | 8 | |
| BP | GO:0001933 | negative regulation of protein phosphorylation | 17/217 | 429/18670 | 1.17E-05 | 0.000612082 | 0.000441475 | CEBPA/EPHA1/FOXO1/GADD45B/GPRC5A/IL1B/IRAK3/JUN/KLF4/LIF/MIR21/MIR221/MYC/RGS2/SOCS1/SOCS3/ZC3H12A | 17 | |
| BP | GO:0051090 | regulation of DNA-binding transcription factor activity | 17/217 | 432/18670 | 1.28E-05 | 0.000660593 | 0.000476465 | ARID5B/FOS/FOSL1/IL18RAP/IL1B/IL6/IRAK3/JUN/KLF4/PIM1/PPRC1/S100A12/S100A8/SIK1/TRIM15/WNT5A/ZC3H12A | 17 | |
| BP | GO:0002292 | T cell differentiation involved in immune response | 7/217 | 68/18670 | 1.38E-05 | 0.000701917 | 0.000506271 | BCL3/BCL6/IL4R/IL6/MIR21/TMEM98/ZC3H12A | 7 | |
| BP | GO:0042698 | ovulation cycle | 7/217 | 69/18670 | 1.52E-05 | 0.000763217 | 0.000550484 | ADAMTS1/AFP/EGR1/HAS2/MMP19/PTX3/TGFB3 | 7 | |
| BP | GO:0051591 | response to cAMP | 8/217 | 97/18670 | 1.74E-05 | 0.000861569 | 0.000621423 | FOS/FOSB/FOSL1/JUN/JUNB/MMP19/STC1/THBD | 8 | |
| BP | GO:0002819 | regulation of adaptive immune response | 10/217 | 160/18670 | 1.83E-05 | 0.000891038 | 0.000642677 | BCL6/CD274/CLCF1/IL1B/IL1RL1/IL4R/IL6/MIR21/SLC11A1/ZC3H12A | 10 | |
| BP | GO:0043542 | endothelial cell migration | 13/217 | 273/18670 | 1.95E-05 | 0.000940829 | 0.00067859 | AMOT/EPHA2/KLF4/MIR21/MIR221/NR4A1/PRSS3/PTGS2/S100P/STC1/THBS1/WNT5A/ZC3H12A | 13 | |
| BP | GO:1903038 | negative regulation of leukocyte cell-cell adhesion | 9/217 | 129/18670 | 2.01E-05 | 0.00095054 | 0.000685594 | BCL6/CD274/IL4R/KLF4/MIR21/MIR221/SDC4/SOCS1/ZC3H12A | 9 | |
| BP | GO:0048525 | negative regulation of viral process | 8/217 | 99/18670 | 2.02E-05 | 0.00095054 | 0.000685594 | APOBEC3A/CCL3/JUN/MIR221/PTX3/TRIM15/ZC3H12A/ZFP36 | 8 | |
| BP | GO:0045073 | regulation of chemokine biosynthetic process | 4/217 | 15/18670 | 2.19E-05 | 0.001016709 | 0.00073332 | EGR1/IL1B/IL6/WNT5A | 4 | |
| BP | GO:0061082 | myeloid leukocyte cytokine production | 5/217 | 30/18670 | 2.28E-05 | 0.001043915 | 0.000752943 | BCL6/IRAK3/NR4A3/TGFB3/WNT5A | 5 | |
| BP | GO:0046632 | alpha-beta T cell differentiation | 8/217 | 101/18670 | 2.34E-05 | 0.001060263 | 0.000764734 | BCL3/BCL6/IL4R/IL6/MIR21/SOCS1/TMEM98/ZC3H12A | 8 | |
| BP | GO:0010718 | positive regulation of epithelial to mesenchymal transition | 6/217 | 50/18670 | 2.39E-05 | 0.001067501 | 0.000769954 | FOXC1/IL1B/IL6/MIR21/MIR221/TGFB3 | 6 | |
| BP | GO:0045598 | regulation of fat cell differentiation | 9/217 | 132/18670 | 2.42E-05 | 0.001069011 | 0.000771044 | CEBPA/FOXO1/IL6/KLF5/MIR21/PTGS2/WNT5A/ZC3H12A/ZFP36 | 9 | |
| BP | GO:0050870 | positive regulation of T cell activation | 11/217 | 202/18670 | 2.53E-05 | 0.001107405 | 0.000798736 | BCL6/CCL2/CD274/IGFBP2/IL1B/IL4R/IL6/MAP3K8/MIR21/SOCS1/TNFSF14 | 11 | |
| BP | GO:0001707 | mesoderm formation | 7/217 | 75/18670 | 2.64E-05 | 0.001141174 | 0.000823093 | EPHA2/FOXC1/KLF4/NR4A3/SOX17/TRIM15/WNT5A | 7 | |
| BP | GO:0002675 | positive regulation of acute inflammatory response | 5/217 | 31/18670 | 2.69E-05 | 0.001142652 | 0.000824159 | C2CD4A/C2CD4B/IL1B/IL6/PTGS2 | 5 | |
| BP | GO:0002697 | regulation of immune effector process | 17/217 | 458/18670 | 2.71E-05 | 0.001142652 | 0.000824159 | BCL6/BIRC3/C5AR1/CLCF1/IL18RAP/IL1B/IL4R/IL6/IRAK3/MIR21/NR4A3/SERPINB9/SPHK2/TGFB3/TRIM15/WNT5A/ZC3H12A | 17 | |
| BP | GO:0007498 | mesoderm development | 9/217 | 135/18670 | 2.89E-05 | 0.001182673 | 0.000853025 | EPHA2/ETS2/FOXC1/KLF4/NR4A3/SOX17/TBX3/TRIM15/WNT5A | 9 | |
| BP | GO:0042033 | chemokine biosynthetic process | 4/217 | 16/18670 | 2.90E-05 | 0.001182673 | 0.000853025 | EGR1/IL1B/IL6/WNT5A | 4 | |
| BP | GO:0050755 | chemokine metabolic process | 4/217 | 16/18670 | 2.90E-05 | 0.001182673 | 0.000853025 | EGR1/IL1B/IL6/WNT5A | 4 | |
| BP | GO:0070555 | response to interleukin-1 | 11/217 | 207/18670 | 3.17E-05 | 0.001274522 | 0.000919272 | CCL2/CCL20/CCL3/EGR1/HAS2/IL1B/IL1RN/IL6/IRAK3/MAP3K8/ZC3H12A | 11 | |
| BP | GO:0042110 | T cell activation | 17/217 | 464/18670 | 3.19E-05 | 0.001274522 | 0.000919272 | BCL3/BCL6/CCL2/CD274/EGR1/IGFBP2/IL1B/IL4R/IL6/MAP3K8/MIR21/SDC4/SLC11A1/SOCS1/TMEM98/TNFSF14/ZC3H12A | 17 | |
| BP | GO:0003012 | muscle system process | 17/217 | 465/18670 | 3.28E-05 | 0.001295651 | 0.000934512 | ACTG2/CHRNB1/CNN1/FOXO1/IL1B/KCNE4/KLF4/MIR21/MYBPH/MYH11/NR4A3/PROK2/PTGS2/RGS2/STC1/TCAP/ZC3H12A | 17 | |
| BP | GO:0002286 | T cell activation involved in immune response | 8/217 | 106/18670 | 3.33E-05 | 0.001301743 | 0.000938906 | BCL3/BCL6/IL4R/IL6/MIR21/SLC11A1/TMEM98/ZC3H12A | 8 | |
| BP | GO:0046631 | alpha-beta T cell activation | 9/217 | 138/18670 | 3.44E-05 | 0.001332594 | 0.000961158 | BCL3/BCL6/CD274/IL4R/IL6/MIR21/SOCS1/TMEM98/ZC3H12A | 9 | |
| BP | GO:0009612 | response to mechanical stimulus | 11/217 | 210/18670 | 3.62E-05 | 0.00138218 | 0.000996923 | FOS/FOSB/FOSL1/IGFBP2/IL1B/JUN/JUNB/PTGS2/TCAP/THBS1/TNFSF14 | 11 | |
| BP | GO:0002700 | regulation of production of molecular mediator of immune response | 9/217 | 139/18670 | 3.64E-05 | 0.00138218 | 0.000996923 | BCL6/CLCF1/IL1B/IL4R/IL6/IRAK3/NR4A3/TGFB3/WNT5A | 9 | |
| BP | GO:0043551 | regulation of phosphatidylinositol 3-kinase activity | 6/217 | 55/18670 | 4.15E-05 | 0.001558713 | 0.00112425 | CISH/KLF4/SOCS1/SOCS2/SOCS3/TNFAIP8L3 | 6 | |
| BP | GO:0043405 | regulation of MAP kinase activity | 14/217 | 337/18670 | 4.23E-05 | 0.001574199 | 0.00113542 | C5AR1/FPR1/GADD45B/GADD45G/IL1B/IRAK3/MAP3K8/PROK2/RGS2/S100A12/SAA1/TGFB3/THBS1/WNT5A | 14 | |
| BP | GO:1903706 | regulation of hemopoiesis | 17/217 | 475/18670 | 4.27E-05 | 0.001574199 | 0.00113542 | BCL6/CCL3/FOS/FOXC1/IL4R/JUN/LIF/MIR21/MIR221/MYC/NFE2/NR4A3/PIM1/SOCS1/THBS1/ZC3H12A/ZFP36 | 17 | |
| BP | GO:0071496 | cellular response to external stimulus | 14/217 | 339/18670 | 4.52E-05 | 0.001647347 | 0.001188179 | AVPR1A/FADS1/FAM107A/FOS/FOSL1/FOXO1/IL1B/JUN/NR4A2/PIM1/PTGS2/SIK1/TNFSF14/ZC3H12A | 14 | |
| BP | GO:0072676 | lymphocyte migration | 8/217 | 111/18670 | 4.63E-05 | 0.001674132 | 0.001207498 | CCL2/CCL20/CCL3/CH25H/DEFA1/SAA1/TNFSF14/WNT5A | 8 | |
| BP | GO:0048608 | reproductive structure development | 16/217 | 431/18670 | 4.68E-05 | 0.001674438 | 0.001207719 | ADAMTS1/AFP/ARID5B/ASPM/CEBPA/FOSL1/FOXC1/JUNB/LIF/MMP19/PTGS2/PTX3/SOCS3/STC1/TBX3/WNT5A | 16 | |
| BP | GO:0061458 | reproductive system development | 16/217 | 434/18670 | 5.08E-05 | 0.001800965 | 0.001298979 | ADAMTS1/AFP/ARID5B/ASPM/CEBPA/FOSL1/FOXC1/JUNB/LIF/MMP19/PTGS2/PTX3/SOCS3/STC1/TBX3/WNT5A | 16 | |
| BP | GO:0043406 | positive regulation of MAP kinase activity | 12/217 | 258/18670 | 5.14E-05 | 0.001804006 | 0.001301172 | C5AR1/FPR1/GADD45B/GADD45G/IL1B/MAP3K8/PROK2/S100A12/SAA1/TGFB3/THBS1/WNT5A | 12 | |
| BP | GO:0022408 | negative regulation of cell-cell adhesion | 10/217 | 181/18670 | 5.25E-05 | 0.001812 | 0.001306938 | BCL6/CD274/IL1RN/IL4R/KLF4/MIR21/MIR221/SDC4/SOCS1/ZC3H12A | 10 | |
| BP | GO:0032609 | interferon-gamma production | 8/217 | 113/18670 | 5.26E-05 | 0.001812 | 0.001306938 | BCL3/CD274/IL18RAP/IL1B/IL1RL1/SLC11A1/WNT5A/ZC3H12A | 8 | |
| BP | GO:0090257 | regulation of muscle system process | 12/217 | 259/18670 | 5.33E-05 | 0.001812 | 0.001306938 | CNN1/FOXO1/KLF4/MIR21/MTMR4/MYBPH/NR4A3/PROK2/PTGS2/RGS2/STC1/ZC3H12A | 12 | |
| BP | GO:0045834 | positive regulation of lipid metabolic process | 9/217 | 146/18670 | 5.35E-05 | 0.001812 | 0.001306938 | AVPR1A/CYP7A1/IL1B/IRS2/NR4A3/PTGS2/SIRT4/SPHK2/TNFAIP8L3 | 9 | |
| BP | GO:0002718 | regulation of cytokine production involved in immune response | 7/217 | 84/18670 | 5.52E-05 | 0.001822533 | 0.001314536 | BCL6/IL1B/IL6/IRAK3/NR4A3/TGFB3/WNT5A | 7 | |
| BP | GO:0043154 | negative regulation of cysteine-type endopeptidase activity involved in apoptotic process | 7/217 | 84/18670 | 5.52E-05 | 0.001822533 | 0.001314536 | BIRC3/KLF4/PLAUR/PTGS2/SERPINB9/THBS1/TNFSF14 | 7 | |
| BP | GO:0002688 | regulation of leukocyte chemotaxis | 8/217 | 114/18670 | 5.61E-05 | 0.001822533 | 0.001314536 | C5AR1/CCL2/CCL3/IL6/SERPINE1/THBS1/TNFSF14/WNT5A | 8 | |
| BP | GO:0042035 | regulation of cytokine biosynthetic process | 8/217 | 114/18670 | 5.61E-05 | 0.001822533 | 0.001314536 | BCL3/EGR1/IL1B/IL6/KLF4/THBS1/WNT5A/ZFP36 | 8 | |
| BP | GO:0032722 | positive regulation of chemokine production | 6/217 | 58/18670 | 5.63E-05 | 0.001822533 | 0.001314536 | EGR1/IL1B/IL1RL1/IL4R/IL6/WNT5A | 6 | |
| BP | GO:0046627 | negative regulation of insulin receptor signaling pathway | 5/217 | 36/18670 | 5.69E-05 | 0.001828823 | 0.001319072 | CISH/IL1B/SOCS1/SOCS2/SOCS3 | 5 | |
| BP | GO:1904705 | regulation of vascular smooth muscle cell proliferation | 7/217 | 85/18670 | 5.96E-05 | 0.001868156 | 0.001347442 | ADAMTS1/CNN1/JUN/MIR21/MIR221/NR4A3/TGFB3 | 7 | |
| BP | GO:1990874 | vascular smooth muscle cell proliferation | 7/217 | 85/18670 | 5.96E-05 | 0.001868156 | 0.001347442 | ADAMTS1/CNN1/JUN/MIR21/MIR221/NR4A3/TGFB3 | 7 | |
| BP | GO:0046660 | female sex differentiation | 8/217 | 115/18670 | 5.97E-05 | 0.001868156 | 0.001347442 | ADAMTS1/AFP/ARID5B/FOXC1/MMP19/PTX3/TBX3/WNT5A | 8 | |
| BP | GO:0045923 | positive regulation of fatty acid metabolic process | 5/217 | 37/18670 | 6.52E-05 | 0.002025461 | 0.001460901 | AVPR1A/IL1B/IRS2/NR4A3/PTGS2 | 5 | |
| BP | GO:0002690 | positive regulation of leukocyte chemotaxis | 7/217 | 87/18670 | 6.91E-05 | 0.002129851 | 0.001536194 | C5AR1/CCL3/IL6/SERPINE1/THBS1/TNFSF14/WNT5A | 7 | |
| BP | GO:0031668 | cellular response to extracellular stimulus | 12/217 | 268/18670 | 7.40E-05 | 0.002201158 | 0.001587626 | AVPR1A/FADS1/FAM107A/FOS/FOSL1/FOXO1/JUN/NR4A2/PIM1/PTGS2/SIK1/ZC3H12A | 12 | |
| BP | GO:1903725 | regulation of phospholipid metabolic process | 7/217 | 88/18670 | 7.44E-05 | 0.002201158 | 0.001587626 | CISH/KLF4/MTMR4/SOCS1/SOCS2/SOCS3/TNFAIP8L3 | 7 | |
| BP | GO:0046636 | negative regulation of alpha-beta T cell activation | 5/217 | 38/18670 | 7.44E-05 | 0.002201158 | 0.001587626 | BCL6/CD274/IL4R/SOCS1/ZC3H12A | 5 | |
| BP | GO:0048246 | macrophage chemotaxis | 5/217 | 38/18670 | 7.44E-05 | 0.002201158 | 0.001587626 | C5AR1/CCL2/CCL3/SAA1/THBS1 | 5 | |
| BP | GO:1900077 | negative regulation of cellular response to insulin stimulus | 5/217 | 38/18670 | 7.44E-05 | 0.002201158 | 0.001587626 | CISH/IL1B/SOCS1/SOCS2/SOCS3 | 5 | |
| BP | GO:2000514 | regulation of CD4-positive, alpha-beta T cell activation | 6/217 | 61/18670 | 7.50E-05 | 0.002201158 | 0.001587626 | BCL6/CD274/IL4R/MIR21/SOCS1/ZC3H12A | 6 | |
| BP | GO:0062013 | positive regulation of small molecule metabolic process | 9/217 | 153/18670 | 7.70E-05 | 0.002242182 | 0.001617215 | AVPR1A/CYP7A1/FOXO1/HAS2/IL1B/IRS2/NR4A3/PFKFB3/PTGS2 | 9 | |
| BP | GO:1904018 | positive regulation of vasculature development | 11/217 | 230/18670 | 8.24E-05 | 0.002366027 | 0.00170654 | C5AR1/EGR1/EPHA1/IL1B/KLF4/MIR21/PTGS2/SERPINE1/THBS1/WNT5A/ZC3H12A | 11 | |
| BP | GO:0050863 | regulation of T cell activation | 13/217 | 314/18670 | 8.25E-05 | 0.002366027 | 0.00170654 | BCL6/CCL2/CD274/IGFBP2/IL1B/IL4R/IL6/MAP3K8/MIR21/SDC4/SOCS1/TNFSF14/ZC3H12A | 13 | |
| BP | GO:1903707 | negative regulation of hemopoiesis | 9/217 | 155/18670 | 8.51E-05 | 0.002416076 | 0.001742639 | BCL6/CCL3/IL4R/MIR21/MIR221/MYC/SOCS1/ZC3H12A/ZFP36 | 9 | |
| BP | GO:0001704 | formation of primary germ layer | 8/217 | 121/18670 | 8.55E-05 | 0.002416076 | 0.001742639 | EPHA2/ETS2/FOXC1/KLF4/NR4A3/SOX17/TRIM15/WNT5A | 8 | |
| BP | GO:0007259 | JAK-STAT cascade | 9/217 | 156/18670 | 8.94E-05 | 0.002477129 | 0.001786674 | BCL3/CCL2/CLCF1/IL6/LIF/MIR221/SOCS1/SOCS2/SOCS3 | 9 | |
| BP | GO:0030856 | regulation of epithelial cell differentiation | 9/217 | 156/18670 | 8.94E-05 | 0.002477129 | 0.001786674 | APOLD1/FOXC1/IL1B/LIF/MAFF/MIR21/SERPINE1/TBX3/ZFP36 | 9 | |
| BP | GO:0070371 | ERK1 and ERK2 cascade | 13/217 | 317/18670 | 9.07E-05 | 0.002477129 | 0.001786674 | C5AR1/CCL2/CCL20/CCL3/EPHA2/IL1B/JUN/KLF4/LIF/MIR21/MIR221/MYC/TNFAIP8L3 | 13 | |
| BP | GO:0051412 | response to corticosterone | 4/217 | 21/18670 | 9.10E-05 | 0.002477129 | 0.001786674 | AVPR1A/FOS/FOSB/FOSL1 | 4 | |
| BP | GO:0090280 | positive regulation of calcium ion import | 4/217 | 21/18670 | 9.10E-05 | 0.002477129 | 0.001786674 | CCL2/CCL3/SPHK2/STC1 | 4 | |
| BP | GO:0042089 | cytokine biosynthetic process | 8/217 | 123/18670 | 9.60E-05 | 0.002594241 | 0.001871144 | BCL3/EGR1/IL1B/IL6/KLF4/THBS1/WNT5A/ZFP36 | 8 | |
| BP | GO:0043550 | regulation of lipid kinase activity | 6/217 | 64/18670 | 9.83E-05 | 0.002612298 | 0.001884168 | CISH/KLF4/SOCS1/SOCS2/SOCS3/TNFAIP8L3 | 6 | |
| BP | GO:0045600 | positive regulation of fat cell differentiation | 6/217 | 64/18670 | 9.83E-05 | 0.002612298 | 0.001884168 | CEBPA/KLF5/MIR21/PTGS2/ZC3H12A/ZFP36 | 6 | |
| BP | GO:2000117 | negative regulation of cysteine-type endopeptidase activity | 7/217 | 92/18670 | 9.87E-05 | 0.002612298 | 0.001884168 | BIRC3/KLF4/PLAUR/PTGS2/SERPINB9/THBS1/TNFSF14 | 7 | |
| BP | GO:0042107 | cytokine metabolic process | 8/217 | 124/18670 | 0.000101562 | 0.002669032 | 0.001925088 | BCL3/EGR1/IL1B/IL6/KLF4/THBS1/WNT5A/ZFP36 | 8 | |
| BP | GO:0002548 | monocyte chemotaxis | 6/217 | 65/18670 | 0.000107308 | 0.002752006 | 0.001984935 | CCL2/CCL20/CCL3/IL6/S100A12/SERPINE1 | 6 | |
| BP | GO:0072678 | T cell migration | 6/217 | 65/18670 | 0.000107308 | 0.002752006 | 0.001984935 | CCL2/CCL20/CCL3/DEFA1/TNFSF14/WNT5A | 6 | |
| BP | GO:0010955 | negative regulation of protein processing | 5/217 | 41/18670 | 0.000107967 | 0.002752006 | 0.001984935 | BIRC3/SERPINE1/SIRT4/THBS1/TMEM98 | 5 | |
| BP | GO:1903318 | negative regulation of protein maturation | 5/217 | 41/18670 | 0.000107967 | 0.002752006 | 0.001984935 | BIRC3/SERPINE1/SIRT4/THBS1/TMEM98 | 5 | |
| BP | GO:0030198 | extracellular matrix organization | 14/217 | 368/18670 | 0.000108381 | 0.002752006 | 0.001984935 | ADAMTS4/BCL3/CRISPLD2/ELF3/FOXC1/HAS2/IL6/MMP19/MYH11/P4HA1/PTX3/SERPINE1/THBS1/VCAN | 14 | |
| BP | GO:0030099 | myeloid cell differentiation | 15/217 | 416/18670 | 0.000111791 | 0.002819542 | 0.002033646 | BCL6/CCL3/CEBPA/EPHA2/FOS/JUN/JUNB/LIF/MIR221/MYC/NFE2/NR4A3/SBNO2/THBS1/ZFP36 | 15 | |
| BP | GO:0050663 | cytokine secretion | 11/217 | 240/18670 | 0.000120234 | 0.003012261 | 0.002172648 | CCL3/CD274/IL1B/IL1RL1/IL4R/S100A12/SAA1/SOCS1/SPHK2/WNT5A/ZC3H12A | 11 | |
| BP | GO:0002687 | positive regulation of leukocyte migration | 8/217 | 128/18670 | 0.000126777 | 0.003131061 | 0.002258335 | C5AR1/CCL20/CCL3/IL6/SERPINE1/THBS1/TNFSF14/WNT5A | 8 | |
| BP | GO:0042108 | positive regulation of cytokine biosynthetic process | 6/217 | 67/18670 | 0.000127174 | 0.003131061 | 0.002258335 | BCL3/EGR1/IL1B/IL6/THBS1/WNT5A | 6 | |
| BP | GO:0019217 | regulation of fatty acid metabolic process | 7/217 | 96/18670 | 0.000129142 | 0.003131061 | 0.002258335 | AVPR1A/CYP7A1/IL1B/IRS2/NR4A3/PTGS2/SIRT4 | 7 | |
| BP | GO:1990823 | response to leukemia inhibitory factor | 7/217 | 96/18670 | 0.000129142 | 0.003131061 | 0.002258335 | ARID5B/GFPT2/KLF4/KLF5/SBNO2/SOCS3/SOX17 | 7 | |
| BP | GO:1990830 | cellular response to leukemia inhibitory factor | 7/217 | 96/18670 | 0.000129142 | 0.003131061 | 0.002258335 | ARID5B/GFPT2/KLF4/KLF5/SBNO2/SOCS3/SOX17 | 7 | |
| BP | GO:0043062 | extracellular structure organization | 15/217 | 422/18670 | 0.000130837 | 0.003134499 | 0.002260815 | ADAMTS4/BCL3/CRISPLD2/ELF3/FOXC1/HAS2/IL6/MMP19/MYH11/P4HA1/PTX3/SDC4/SERPINE1/THBS1/VCAN | 15 | |
| BP | GO:0035821 | modification of morphology or physiology of other organism | 9/217 | 164/18670 | 0.000130952 | 0.003134499 | 0.002260815 | CCL3/DEFA1/DEFB118/JUN/MIR221/PTX3/S100A12/SERPINB9/ZC3H12A | 9 | |
| BP | GO:0002440 | production of molecular mediator of immune response | 12/217 | 286/18670 | 0.000136665 | 0.003250542 | 0.002344513 | BCL6/CLCF1/IL18RAP/IL1B/IL4R/IL6/IRAK3/NR4A3/SLC11A1/SPHK2/TGFB3/WNT5A | 12 | |
| BP | GO:0050671 | positive regulation of lymphocyte proliferation | 8/217 | 130/18670 | 0.0001412 | 0.003331451 | 0.00240287 | BCL6/CD274/CLCF1/IGFBP2/IL1B/IL6/IRS2/MIR21 | 8 | |
| BP | GO:0045766 | positive regulation of angiogenesis | 10/217 | 204/18670 | 0.000141839 | 0.003331451 | 0.00240287 | C5AR1/EPHA1/IL1B/KLF4/MIR21/PTGS2/SERPINE1/THBS1/WNT5A/ZC3H12A | 10 | |
| BP | GO:0097696 | STAT cascade | 9/217 | 166/18670 | 0.000143554 | 0.003350784 | 0.002416814 | BCL3/CCL2/CLCF1/IL6/LIF/MIR221/SOCS1/SOCS2/SOCS3 | 9 | |
| BP | GO:0032946 | positive regulation of mononuclear cell proliferation | 8/217 | 131/18670 | 0.000148904 | 0.003454199 | 0.002491404 | BCL6/CD274/CLCF1/IGFBP2/IL1B/IL6/IRS2/MIR21 | 8 | |
| BP | GO:0051251 | positive regulation of lymphocyte activation | 13/217 | 334/18670 | 0.000152543 | 0.003515385 | 0.002535536 | BCL6/CCL2/CD274/CLCF1/IGFBP2/IL1B/IL4R/IL6/IRS2/MAP3K8/MIR21/SOCS1/TNFSF14 | 13 | |
| BP | GO:0042832 | defense response to protozoan | 4/217 | 24/18670 | 0.000157146 | 0.003515385 | 0.002535536 | BCL3/DEFA1/IL4R/SLC11A1 | 4 | |
| BP | GO:0046426 | negative regulation of JAK-STAT cascade | 4/217 | 24/18670 | 0.000157146 | 0.003515385 | 0.002535536 | BCL3/SOCS1/SOCS2/SOCS3 | 4 | |
| BP | GO:0046639 | negative regulation of alpha-beta T cell differentiation | 4/217 | 24/18670 | 0.000157146 | 0.003515385 | 0.002535536 | BCL6/IL4R/SOCS1/ZC3H12A | 4 | |
| BP | GO:0046697 | decidualization | 4/217 | 24/18670 | 0.000157146 | 0.003515385 | 0.002535536 | JUNB/LIF/PTGS2/STC1 | 4 | |
| BP | GO:0071706 | tumor necrosis factor superfamily cytokine production | 9/217 | 168/18670 | 0.000157154 | 0.003515385 | 0.002535536 | BCL3/CCL3/CD274/IRAK3/SPHK2/THBS1/WNT5A/ZC3H12A/ZFP36 | 9 | |
| BP | GO:0010632 | regulation of epithelial cell migration | 12/217 | 291/18670 | 0.00016061 | 0.003571423 | 0.002575955 | EPHA2/HAS2/HBEGF/JUN/KLF4/MIR21/MIR221/PTGS2/STC1/THBS1/WNT5A/ZC3H12A | 12 | |
| BP | GO:0032570 | response to progesterone | 5/217 | 45/18670 | 0.000169509 | 0.003725229 | 0.002686889 | FOS/FOSB/FOSL1/TGFB3/THBS1 | 5 | |
| BP | GO:0045581 | negative regulation of T cell differentiation | 5/217 | 45/18670 | 0.000169509 | 0.003725229 | 0.002686889 | BCL6/IL4R/MIR21/SOCS1/ZC3H12A | 5 | |
| BP | GO:1901653 | cellular response to peptide | 14/217 | 385/18670 | 0.000173491 | 0.003790573 | 0.00273402 | CISH/FOXO1/IGFBP1/IL1B/IRS2/KLF11/KLF4/KLF5/NR4A1/NR4A2/NR4A3/SOCS1/SOCS2/SOCS3 | 14 | |
| BP | GO:0051918 | negative regulation of fibrinolysis | 3/217 | 10/18670 | 0.000174969 | 0.00380077 | 0.002741375 | SERPINE1/THBD/THBS1 | 3 | |
| BP | GO:0032649 | regulation of interferon-gamma production | 7/217 | 101/18670 | 0.000177459 | 0.003810805 | 0.002748613 | BCL3/CD274/IL1B/IL1RL1/SLC11A1/WNT5A/ZC3H12A | 7 | |
| BP | GO:1903426 | regulation of reactive oxygen species biosynthetic process | 7/217 | 101/18670 | 0.000177459 | 0.003810805 | 0.002748613 | IL1B/KLF4/MIR21/PTGS2/PTX3/SPHK2/ZC3H12A | 7 | |
| BP | GO:0010634 | positive regulation of epithelial cell migration | 9/217 | 171/18670 | 0.000179559 | 0.003833987 | 0.002765334 | HAS2/HBEGF/JUN/MIR21/MIR221/PTGS2/THBS1/WNT5A/ZC3H12A | 9 | |
| BP | GO:0048511 | rhythmic process | 12/217 | 295/18670 | 0.000182269 | 0.003869869 | 0.002791214 | ADAMTS1/AFP/DBP/EGR1/HAS2/JUN/MMP19/PROK2/PTX3/SERPINE1/SIK1/TGFB3 | 12 | |
| BP | GO:0001562 | response to protozoan | 4/217 | 25/18670 | 0.000185382 | 0.003913861 | 0.002822944 | BCL3/DEFA1/IL4R/SLC11A1 | 4 | |
| BP | GO:0022602 | ovulation cycle process | 5/217 | 46/18670 | 0.000188398 | 0.0039553 | 0.002852832 | ADAMTS1/AFP/MMP19/PTX3/TGFB3 | 5 | |
| BP | GO:0016525 | negative regulation of angiogenesis | 9/217 | 173/18670 | 0.000195926 | 0.004090497 | 0.002950346 | AMOT/EPHA2/FOXC1/ISM1/KLF4/MIR21/MIR221/SERPINE1/THBS1 | 9 | |
| BP | GO:1902106 | negative regulation of leukocyte differentiation | 7/217 | 103/18670 | 0.00020047 | 0.004162238 | 0.003002091 | BCL6/CCL3/IL4R/MIR21/MYC/SOCS1/ZC3H12A | 7 | |
| BP | GO:0046425 | regulation of JAK-STAT cascade | 8/217 | 137/18670 | 0.000202749 | 0.004186439 | 0.003019546 | BCL3/CLCF1/IL6/LIF/MIR221/SOCS1/SOCS2/SOCS3 | 8 | |
| BP | GO:0014911 | positive regulation of smooth muscle cell migration | 5/217 | 47/18670 | 0.00020885 | 0.004242479 | 0.003059966 | ADAMTS1/HAS2/MIR21/MIR221/NR4A3 | 5 | |
| BP | GO:0043370 | regulation of CD4-positive, alpha-beta T cell differentiation | 5/217 | 47/18670 | 0.00020885 | 0.004242479 | 0.003059966 | BCL6/IL4R/MIR21/SOCS1/ZC3H12A | 5 | |
| BP | GO:0061614 | pri-miRNA transcription by RNA polymerase II | 5/217 | 47/18670 | 0.00020885 | 0.004242479 | 0.003059966 | FOS/FOSL1/JUN/KLF4/KLF5 | 5 | |
| BP | GO:0070372 | regulation of ERK1 and ERK2 cascade | 12/217 | 300/18670 | 0.000212813 | 0.004290875 | 0.003094873 | C5AR1/CCL2/CCL20/CCL3/EPHA2/IL1B/JUN/KLF4/LIF/MIR21/MIR221/TNFAIP8L3 | 12 | |
| BP | GO:2000181 | negative regulation of blood vessel morphogenesis | 9/217 | 175/18670 | 0.000213516 | 0.004290875 | 0.003094873 | AMOT/EPHA2/FOXC1/ISM1/KLF4/MIR21/MIR221/SERPINE1/THBS1 | 9 | |
| BP | GO:0034114 | regulation of heterotypic cell-cell adhesion | 4/217 | 26/18670 | 0.000217103 | 0.004297476 | 0.003099633 | IL1B/IL1RN/KLF4/MIR221 | 4 | |
| BP | GO:1904754 | positive regulation of vascular associated smooth muscle cell migration | 4/217 | 26/18670 | 0.000217103 | 0.004297476 | 0.003099633 | ADAMTS1/MIR21/MIR221/NR4A3 | 4 | |
| BP | GO:0043281 | regulation of cysteine-type endopeptidase activity involved in apoptotic process | 10/217 | 215/18670 | 0.000217275 | 0.004297476 | 0.003099633 | BIRC3/GRAMD4/KLF4/MYC/PLAUR/PTGS2/S100A8/SERPINB9/THBS1/TNFSF14 | 10 | |
| BP | GO:0014015 | positive regulation of gliogenesis | 6/217 | 74/18670 | 0.000220684 | 0.004319424 | 0.003115464 | CCL3/CLCF1/IL1B/IL6/LIF/MIR221 | 6 | |
| BP | GO:1903036 | positive regulation of response to wounding | 6/217 | 74/18670 | 0.000220684 | 0.004319424 | 0.003115464 | HBEGF/MIR21/MIR221/SERPINE1/THBD/THBS1 | 6 | |
| BP | GO:0070665 | positive regulation of leukocyte proliferation | 8/217 | 139/18670 | 0.0002239 | 0.004359673 | 0.003144494 | BCL6/CD274/CLCF1/IGFBP2/IL1B/IL6/IRS2/MIR21 | 8 | |
| BP | GO:0002699 | positive regulation of immune effector process | 10/217 | 216/18670 | 0.00022555 | 0.00436915 | 0.00315133 | CLCF1/IL18RAP/IL1B/IL4R/IL6/MIR21/NR4A3/SPHK2/TRIM15/WNT5A | 10 | |
| BP | GO:0070997 | neuron death | 13/217 | 348/18670 | 0.000227877 | 0.004391599 | 0.003167521 | C5AR1/CCL2/CCL3/CIT/CLCF1/EGR1/FOS/JUN/NCOA7/NR4A2/NR4A3/TGFB3/WNT5A | 13 | |
| BP | GO:0060324 | face development | 5/217 | 48/18670 | 0.000230951 | 0.004428123 | 0.003193865 | ALDH1A3/ARID5B/CRISPLD2/TGFB3/WNT5A | 5 | |
| BP | GO:0001837 | epithelial to mesenchymal transition | 8/217 | 140/18670 | 0.000235135 | 0.004437445 | 0.003200588 | FOXC1/HAS2/IL1B/IL6/MIR21/MIR221/TGFB3/WNT5A | 8 | |
| BP | GO:0150076 | neuroinflammatory response | 6/217 | 75/18670 | 0.000237576 | 0.004437445 | 0.003200588 | C5AR1/CCL3/IL1B/IL6/JUN/PTGS2 | 6 | |
| BP | GO:0032494 | response to peptidoglycan | 3/217 | 11/18670 | 0.000238522 | 0.004437445 | 0.003200588 | C5AR1/IL6/IRAK3 | 3 | |
| BP | GO:0034115 | negative regulation of heterotypic cell-cell adhesion | 3/217 | 11/18670 | 0.000238522 | 0.004437445 | 0.003200588 | IL1RN/KLF4/MIR221 | 3 | |
| BP | GO:0044793 | negative regulation by host of viral process | 3/217 | 11/18670 | 0.000238522 | 0.004437445 | 0.003200588 | MIR221/PTX3/ZC3H12A | 3 | |
| BP | GO:0070587 | regulation of cell-cell adhesion involved in gastrulation | 3/217 | 11/18670 | 0.000238522 | 0.004437445 | 0.003200588 | IL1RN/KLF4/MIR221 | 3 | |
| BP | GO:0001503 | ossification | 14/217 | 398/18670 | 0.000243894 | 0.00451505 | 0.003256563 | CCL3/CEBPA/CEBPD/EPHA2/FOXC1/IL6/JUNB/MIR21/PTGS2/SBNO2/STC1/TGFB3/VCAN/WNT5A | 14 | |
| BP | GO:0008286 | insulin receptor signaling pathway | 8/217 | 141/18670 | 0.000246828 | 0.004546953 | 0.003279573 | CISH/FOXO1/IGFBP1/IL1B/IRS2/SOCS1/SOCS2/SOCS3 | 8 | |
| BP | GO:0051091 | positive regulation of DNA-binding transcription factor activity | 11/217 | 261/18670 | 0.000249489 | 0.004564716 | 0.003292385 | ARID5B/FOSL1/IL18RAP/IL1B/IL6/IRAK3/PPRC1/S100A12/S100A8/TRIM15/WNT5A | 11 | |
| BP | GO:0002825 | regulation of T-helper 1 type immune response | 4/217 | 27/18670 | 0.000252551 | 0.004564716 | 0.003292385 | IL1B/IL1RL1/IL4R/SLC11A1 | 4 | |
| BP | GO:1901623 | regulation of lymphocyte chemotaxis | 4/217 | 27/18670 | 0.000252551 | 0.004564716 | 0.003292385 | CCL2/CCL3/TNFSF14/WNT5A | 4 | |
| BP | GO:1903034 | regulation of response to wounding | 9/217 | 179/18670 | 0.000252651 | 0.004564716 | 0.003292385 | HBEGF/KLF4/MIR21/MIR221/PLAUR/SERPINE1/THBD/THBS1/TNFRSF12A | 9 | |
| BP | GO:1904707 | positive regulation of vascular smooth muscle cell proliferation | 5/217 | 49/18670 | 0.000254786 | 0.004581265 | 0.003304321 | ADAMTS1/JUN/MIR21/MIR221/NR4A3 | 5 | |
| BP | GO:0002285 | lymphocyte activation involved in immune response | 9/217 | 181/18670 | 0.000274344 | 0.004864248 | 0.003508428 | BCL3/BCL6/CLCF1/IL4R/IL6/MIR21/SLC11A1/TMEM98/ZC3H12A | 9 | |
| BP | GO:0010565 | regulation of cellular ketone metabolic process | 9/217 | 181/18670 | 0.000274344 | 0.004864248 | 0.003508428 | AVPR1A/CYP7A1/EGR1/IL1B/IRS2/MIR21/NR4A3/PTGS2/SIRT4 | 9 | |
| BP | GO:0051851 | modification by host of symbiont morphology or physiology | 6/217 | 77/18670 | 0.000274407 | 0.004864248 | 0.003508428 | CCL3/DEFA1/JUN/MIR221/PTX3/ZC3H12A | 6 | |
| BP | GO:0001702 | gastrulation with mouth forming second | 4/217 | 28/18670 | 0.000291975 | 0.005103448 | 0.003680956 | AMOT/ETS2/NAT8B/WNT5A | 4 | |
| BP | GO:0010818 | T cell chemotaxis | 4/217 | 28/18670 | 0.000291975 | 0.005103448 | 0.003680956 | CCL3/DEFA1/TNFSF14/WNT5A | 4 | |
| BP | GO:0042730 | fibrinolysis | 4/217 | 28/18670 | 0.000291975 | 0.005103448 | 0.003680956 | PLAUR/SERPINE1/THBD/THBS1 | 4 | |
| BP | GO:0034612 | response to tumor necrosis factor | 12/217 | 312/18670 | 0.000304412 | 0.005276425 | 0.003805718 | BIRC3/CCL2/CCL20/CCL3/CEBPA/HAS2/PTGS2/THBS1/TNFRSF12A/TNFSF14/ZC3H12A/ZFP36 | 12 | |
| BP | GO:0007566 | embryo implantation | 5/217 | 51/18670 | 0.000308016 | 0.005276425 | 0.003805718 | IL1B/LIF/MIR21/PTGS2/STC1 | 5 | |
| BP | GO:0043409 | negative regulation of MAPK cascade | 9/217 | 184/18670 | 0.000309762 | 0.005276425 | 0.003805718 | FOXO1/IL1B/IRAK3/KLF4/LIF/MIR21/MIR221/MYC/RGS2 | 9 | |
| BP | GO:0042542 | response to hydrogen peroxide | 8/217 | 146/18670 | 0.000312664 | 0.005276425 | 0.003805718 | FOSL1/FOXO1/IL18RAP/IL6/JUN/KLF4/MIR21/NR4A3 | 8 | |
| BP | GO:1904892 | regulation of STAT cascade | 8/217 | 146/18670 | 0.000312664 | 0.005276425 | 0.003805718 | BCL3/CLCF1/IL6/LIF/MIR221/SOCS1/SOCS2/SOCS3 | 8 | |
| BP | GO:1901214 | regulation of neuron death | 12/217 | 313/18670 | 0.000313364 | 0.005276425 | 0.003805718 | C5AR1/CCL2/CCL3/CLCF1/EGR1/FOS/JUN/NCOA7/NR4A2/NR4A3/TGFB3/WNT5A | 12 | |
| BP | GO:0031392 | regulation of prostaglandin biosynthetic process | 3/217 | 12/18670 | 0.000315306 | 0.005276425 | 0.003805718 | AVPR1A/IL1B/PTGS2 | 3 | |
| BP | GO:0045080 | positive regulation of chemokine biosynthetic process | 3/217 | 12/18670 | 0.000315306 | 0.005276425 | 0.003805718 | EGR1/IL1B/WNT5A | 3 | |
| BP | GO:0070586 | cell-cell adhesion involved in gastrulation | 3/217 | 12/18670 | 0.000315306 | 0.005276425 | 0.003805718 | IL1RN/KLF4/MIR221 | 3 | |
| BP | GO:0006936 | muscle contraction | 13/217 | 360/18670 | 0.000315912 | 0.005276425 | 0.003805718 | ACTG2/CHRNB1/CNN1/KCNE4/MIR21/MYBPH/MYH11/PROK2/PTGS2/RGS2/STC1/TCAP/ZC3H12A | 13 | |
| BP | GO:1903708 | positive regulation of hemopoiesis | 9/217 | 185/18670 | 0.000322376 | 0.005323666 | 0.003839792 | BCL6/FOS/FOXC1/IL4R/JUN/LIF/MIR21/MIR221/SOCS1 | 9 | |
| BP | GO:0043491 | protein kinase B signaling | 11/217 | 269/18670 | 0.000322911 | 0.005323666 | 0.003839792 | CCL2/CCL3/EPHA2/HBEGF/IL1B/IRS2/KLF4/MIR21/MIR221/THBS1/TNFAIP8L3 | 11 | |
| BP | GO:0062012 | regulation of small molecule metabolic process | 15/217 | 459/18670 | 0.000323161 | 0.005323666 | 0.003839792 | AVPR1A/CYP7A1/EGR1/FOXO1/GPAM/HAS2/IL1B/IRS2/MIR21/NR4A3/PFKFB3/PTGS2/SIK1/SIRT4/SPHK2 | 15 | |
| BP | GO:0002460 | adaptive immune response based on somatic recombination of immune receptors built from immunoglobulin superfamily domains | 13/217 | 361/18670 | 0.000324406 | 0.005323666 | 0.003839792 | BCL3/BCL6/CD274/CLCF1/IL18RAP/IL1B/IL1RL1/IL4R/IL6/MIR21/SLC11A1/TMEM98/ZC3H12A | 13 | |
| BP | GO:0071236 | cellular response to antibiotic | 8/217 | 147/18670 | 0.00032741 | 0.005349596 | 0.003858494 | EGR1/FOXO1/IL18RAP/IL6/KLF4/MIR21/NR4A3/ZC3H12A | 8 | |
| BP | GO:0010575 | positive regulation of vascular endothelial growth factor production | 4/217 | 29/18670 | 0.000335625 | 0.00539124 | 0.003888531 | C5AR1/IL1B/IL6/PTGS2 | 4 | |
| BP | GO:2000515 | negative regulation of CD4-positive, alpha-beta T cell activation | 4/217 | 29/18670 | 0.000335625 | 0.00539124 | 0.003888531 | BCL6/CD274/IL4R/ZC3H12A | 4 | |
| BP | GO:0050868 | negative regulation of T cell activation | 7/217 | 112/18670 | 0.000335697 | 0.00539124 | 0.003888531 | BCL6/CD274/IL4R/MIR21/SDC4/SOCS1/ZC3H12A | 7 | |
| BP | GO:0051817 | modification of morphology or physiology of other organism involved in symbiotic interaction | 7/217 | 112/18670 | 0.000335697 | 0.00539124 | 0.003888531 | CCL3/DEFA1/JUN/MIR221/PTX3/SERPINB9/ZC3H12A | 7 | |
| BP | GO:1901343 | negative regulation of vasculature development | 9/217 | 187/18670 | 0.000348881 | 0.005579122 | 0.004024044 | AMOT/EPHA2/FOXC1/ISM1/KLF4/MIR21/MIR221/SERPINE1/THBS1 | 9 | |
| BP | GO:0046651 | lymphocyte proliferation | 11/217 | 272/18670 | 0.000354774 | 0.00564933 | 0.004074682 | BCL6/CD274/CLCF1/IGFBP2/IL1B/IL6/IRS2/MIR21/SDC4/SLC11A1/TNFSF14 | 11 | |
| BP | GO:0048708 | astrocyte differentiation | 6/217 | 81/18670 | 0.000361443 | 0.005731241 | 0.004133763 | C5AR1/CLCF1/IL1B/IL6/LIF/S100A8 | 6 | |
| BP | GO:0038066 | p38MAPK cascade | 5/217 | 53/18670 | 0.000369271 | 0.005806366 | 0.004187948 | GADD45B/GADD45G/IL1B/ZC3H12A/ZFP36 | 5 | |
| BP | GO:1905517 | macrophage migration | 5/217 | 53/18670 | 0.000369271 | 0.005806366 | 0.004187948 | C5AR1/CCL2/CCL3/SAA1/THBS1 | 5 | |
| BP | GO:0050777 | negative regulation of immune response | 8/217 | 150/18670 | 0.00037509 | 0.005873279 | 0.00423621 | BCL6/IL1RL1/IL4R/IRAK3/MIR21/SERPINB9/TGFB3/ZC3H12A | 8 | |
| BP | GO:0032943 | mononuclear cell proliferation | 11/217 | 274/18670 | 0.000377454 | 0.00588469 | 0.00424444 | BCL6/CD274/CLCF1/IGFBP2/IL1B/IL6/IRS2/MIR21/SDC4/SLC11A1/TNFSF14 | 11 | |
| BP | GO:0051346 | negative regulation of hydrolase activity | 15/217 | 466/18670 | 0.00037895 | 0.00588469 | 0.00424444 | AMOT/BIRC3/ELFN1/KLF4/MIR21/PLAUR/PPP1R15A/PTGS2/PTX3/RGS2/SERPINB9/SERPINE1/SPHK2/THBS1/TNFSF14 | 15 | |
| BP | GO:0038111 | interleukin-7-mediated signaling pathway | 4/217 | 30/18670 | 0.000383755 | 0.005900495 | 0.00425584 | CISH/IRS2/SOCS1/SOCS2 | 4 | |
| BP | GO:1904893 | negative regulation of STAT cascade | 4/217 | 30/18670 | 0.000383755 | 0.005900495 | 0.00425584 | BCL3/SOCS1/SOCS2/SOCS3 | 4 | |
| BP | GO:0032637 | interleukin-8 production | 6/217 | 82/18670 | 0.000386248 | 0.005900495 | 0.00425584 | BCL3/IL1B/KLF4/LRRC19/SERPINE1/WNT5A | 6 | |
| BP | GO:0051702 | interaction with symbiont | 6/217 | 82/18670 | 0.000386248 | 0.005900495 | 0.00425584 | CCL3/DEFA1/JUN/MIR221/PTX3/ZC3H12A | 6 | |
| BP | GO:0060759 | regulation of response to cytokine stimulus | 9/217 | 190/18670 | 0.000391993 | 0.005964013 | 0.004301653 | BIRC3/IL1RN/IL6/IRAK3/KLF4/MIR21/SOCS1/SOCS3/WNT5A | 9 | |
| BP | GO:1903960 | negative regulation of anion transmembrane transport | 3/217 | 13/18670 | 0.000406391 | 0.00615813 | 0.004441664 | IRS2/RGS2/THBS1 | 3 | |
| BP | GO:0000187 | activation of MAPK activity | 8/217 | 152/18670 | 0.000409907 | 0.006173967 | 0.004453087 | C5AR1/FPR1/IL1B/PROK2/SAA1/TGFB3/THBS1/WNT5A | 8 | |
| BP | GO:0097305 | response to alcohol | 10/217 | 233/18670 | 0.000412167 | 0.006173967 | 0.004453087 | AVPR1A/CCL3/CYP7A1/FOS/FOSB/FOSL1/KLF4/RGS2/S100A8/SPHK2 | 10 | |
| BP | GO:0042509 | regulation of tyrosine phosphorylation of STAT protein | 6/217 | 83/18670 | 0.000412364 | 0.006173967 | 0.004453087 | CLCF1/IL6/LIF/MIR221/SOCS1/SOCS3 | 6 | |
| BP | GO:0048839 | inner ear development | 9/217 | 192/18670 | 0.000423105 | 0.006309634 | 0.004550939 | ALDH1A3/CEBPA/CEBPD/LRIG3/NR4A3/SDC4/TCAP/TGFB3/WNT5A | 9 | |
| BP | GO:0045620 | negative regulation of lymphocyte differentiation | 5/217 | 55/18670 | 0.000439312 | 0.006456701 | 0.004657014 | BCL6/IL4R/MIR21/SOCS1/ZC3H12A | 5 | |
| BP | GO:1901654 | response to ketone | 9/217 | 193/18670 | 0.000439407 | 0.006456701 | 0.004657014 | AVPR1A/FOS/FOSB/FOSL1/FOXO1/KLF4/SPHK2/TGFB3/THBS1 | 9 | |
| BP | GO:0014910 | regulation of smooth muscle cell migration | 6/217 | 84/18670 | 0.000439839 | 0.006456701 | 0.004657014 | ADAMTS1/HAS2/MIR21/MIR221/NR4A3/SERPINE1 | 6 | |
| BP | GO:0046889 | positive regulation of lipid biosynthetic process | 6/217 | 84/18670 | 0.000439839 | 0.006456701 | 0.004657014 | AVPR1A/CYP7A1/IL1B/PTGS2/SIRT4/SPHK2 | 6 | |
| BP | GO:0001701 | in utero embryonic development | 13/217 | 373/18670 | 0.000442553 | 0.006471262 | 0.004667516 | AMOT/CEBPA/ELF3/FOSL1/FOXC1/JUNB/LIF/MAFF/SLC25A34/SOCS3/SOX17/TBX3/TGFB3 | 13 | |
| BP | GO:0009615 | response to virus | 12/217 | 326/18670 | 0.000451659 | 0.006578816 | 0.004745091 | APOBEC3A/BCL3/BIRC3/DEFA1/FOSL1/IFIT2/IL1B/IL6/IRAK3/RTP4/TRIM15/ZC3H12A | 12 | |
| BP | GO:0046677 | response to antibiotic | 12/217 | 327/18670 | 0.000464152 | 0.006734683 | 0.004857513 | EGR1/FOSL1/FOXO1/IL18RAP/IL6/JUN/KLF4/MIR21/NR4A3/RGS2/S100A8/ZC3H12A | 12 | |
| BP | GO:2000377 | regulation of reactive oxygen species metabolic process | 9/217 | 195/18670 | 0.000473563 | 0.006844808 | 0.004936943 | FOXO1/IL1B/KLF4/MIR21/PTGS2/PTX3/SPHK2/THBS1/ZC3H12A | 9 | |
| BP | GO:0002685 | regulation of leukocyte migration | 9/217 | 196/18670 | 0.000491442 | 0.007059003 | 0.005091435 | C5AR1/CCL2/CCL20/CCL3/IL6/SERPINE1/THBS1/TNFSF14/WNT5A | 9 | |
| BP | GO:0051385 | response to mineralocorticoid | 4/217 | 32/18670 | 0.000494484 | 0.007059003 | 0.005091435 | AVPR1A/FOS/FOSB/FOSL1 | 4 | |
| BP | GO:1900745 | positive regulation of p38MAPK cascade | 4/217 | 32/18670 | 0.000494484 | 0.007059003 | 0.005091435 | GADD45B/GADD45G/IL1B/ZC3H12A | 4 | |
| BP | GO:0007260 | tyrosine phosphorylation of STAT protein | 6/217 | 86/18670 | 0.00049906 | 0.007059003 | 0.005091435 | CLCF1/IL6/LIF/MIR221/SOCS1/SOCS3 | 6 | |
| BP | GO:2000116 | regulation of cysteine-type endopeptidase activity | 10/217 | 239/18670 | 0.000502999 | 0.007059003 | 0.005091435 | BIRC3/GRAMD4/KLF4/MYC/PLAUR/PTGS2/S100A8/SERPINB9/THBS1/TNFSF14 | 10 | |
| BP | GO:0048568 | embryonic organ development | 14/217 | 428/18670 | 0.000505526 | 0.007059003 | 0.005091435 | ALDH1A3/CEBPA/EPHA2/FOXC1/JUNB/LIF/LRIG3/NR4A3/SOCS3/SOX17/TBX3/TCAP/TGFB3/WNT5A | 14 | |
| BP | GO:0051047 | positive regulation of secretion | 14/217 | 428/18670 | 0.000505526 | 0.007059003 | 0.005091435 | AVPR1A/CCL3/CD274/IL1B/IL1RL1/IL4R/IL6/IRS2/S100A8/SAA1/SDC4/SPHK2/TGFB3/WNT5A | 14 | |
| BP | GO:0072089 | stem cell proliferation | 7/217 | 120/18670 | 0.000509839 | 0.007059003 | 0.005091435 | ASPM/MIR221/PIM1/RNF43/SOX17/TBX3/WNT5A | 7 | |
| BP | GO:0010935 | regulation of macrophage cytokine production | 3/217 | 14/18670 | 0.000512801 | 0.007059003 | 0.005091435 | IRAK3/TGFB3/WNT5A | 3 | |
| BP | GO:0045064 | T-helper 2 cell differentiation | 3/217 | 14/18670 | 0.000512801 | 0.007059003 | 0.005091435 | BCL3/BCL6/IL4R | 3 | |
| BP | GO:0051917 | regulation of fibrinolysis | 3/217 | 14/18670 | 0.000512801 | 0.007059003 | 0.005091435 | SERPINE1/THBD/THBS1 | 3 | |
| BP | GO:0072216 | positive regulation of metanephros development | 3/217 | 14/18670 | 0.000512801 | 0.007059003 | 0.005091435 | EGR1/LIF/MYC | 3 | |
| BP | GO:2001279 | regulation of unsaturated fatty acid biosynthetic process | 3/217 | 14/18670 | 0.000512801 | 0.007059003 | 0.005091435 | AVPR1A/IL1B/PTGS2 | 3 | |
| BP | GO:0051348 | negative regulation of transferase activity | 11/217 | 285/18670 | 0.000525142 | 0.007202495 | 0.005194932 | CEBPA/EPHA1/GADD45B/GPRC5A/IL1B/IRAK3/IRS2/RGS2/SOCS1/SOCS3/ZFP36 | 11 | |
| BP | GO:0046890 | regulation of lipid biosynthetic process | 9/217 | 198/18670 | 0.000528867 | 0.007227208 | 0.005212756 | AVPR1A/CYP7A1/EGR1/GPAM/IL1B/PTGS2/SIK1/SIRT4/SPHK2 | 9 | |
| BP | GO:0031032 | actomyosin structure organization | 9/217 | 199/18670 | 0.000548438 | 0.0074675 | 0.005386071 | CIT/CNN1/EPHA1/FRMD6/MIR21/MYH11/SDC4/TCAP/TGFB3 | 9 | |
| BP | GO:0071902 | positive regulation of protein serine/threonine kinase activity | 12/217 | 334/18670 | 0.000560038 | 0.007529365 | 0.005430692 | C5AR1/FPR1/GADD45B/GADD45G/IL1B/MAP3K8/PROK2/S100A12/SAA1/TGFB3/THBS1/WNT5A | 12 | |
| BP | GO:0061900 | glial cell activation | 5/217 | 58/18670 | 0.000562571 | 0.007529365 | 0.005430692 | C5AR1/CCL3/IL1B/IL6/JUN | 5 | |
| BP | GO:0019730 | antimicrobial humoral response | 7/217 | 122/18670 | 0.000562999 | 0.007529365 | 0.005430692 | BCL3/DEFA1/DEFB118/PRSS3/S100A12/S100A8/SLC11A1 | 7 | |
| BP | GO:0043500 | muscle adaptation | 7/217 | 122/18670 | 0.000562999 | 0.007529365 | 0.005430692 | FOXO1/IL1B/KLF4/MIR21/NR4A3/RGS2/TCAP | 7 | |
| BP | GO:1903409 | reactive oxygen species biosynthetic process | 7/217 | 122/18670 | 0.000562999 | 0.007529365 | 0.005430692 | IL1B/KLF4/MIR21/PTGS2/PTX3/SPHK2/ZC3H12A | 7 | |
| BP | GO:0002793 | positive regulation of peptide secretion | 11/217 | 288/18670 | 0.000572914 | 0.007620728 | 0.00549659 | CCL3/CD274/IL1B/IL1RL1/IL4R/IL6/IRS2/S100A8/SAA1/TGFB3/WNT5A | 11 | |
| BP | GO:0051249 | regulation of lymphocyte activation | 15/217 | 485/18670 | 0.000573887 | 0.007620728 | 0.00549659 | BCL6/CCL2/CD274/CLCF1/IGFBP2/IL1B/IL4R/IL6/IRS2/MAP3K8/MIR21/SDC4/SOCS1/TNFSF14/ZC3H12A | 15 | |
| BP | GO:0048732 | gland development | 14/217 | 434/18670 | 0.000579723 | 0.007671125 | 0.005532939 | ALDH1A3/ARID5B/CEBPA/CIT/DBP/ELF3/EPHA2/FOXC1/IRS2/JUN/SOCS2/TBX3/TGFB3/WNT5A | 14 | |
| BP | GO:0042692 | muscle cell differentiation | 13/217 | 385/18670 | 0.000595422 | 0.007851214 | 0.005662832 | AVPR1A/CDH15/CHRNB1/IL4R/KLF5/MIR21/MIR221/MYH11/RGS2/SIK1/TBX3/TCAP/TNFSF14 | 13 | |
| BP | GO:0046427 | positive regulation of JAK-STAT cascade | 6/217 | 89/18670 | 0.000599323 | 0.007864906 | 0.005672707 | CLCF1/IL6/LIF/MIR221/SOCS1/SOCS3 | 6 | |
| BP | GO:0032635 | interleukin-6 production | 8/217 | 161/18670 | 0.000600646 | 0.007864906 | 0.005672707 | IL18RAP/IL1B/IL1RN/IL6/IRAK3/SPHK2/WNT5A/ZC3H12A | 8 | |
| BP | GO:0032890 | regulation of organic acid transport | 5/217 | 59/18670 | 0.000608918 | 0.00794553 | 0.005730859 | AVPR1A/IL1B/IRS2/RGS2/THBS1 | 5 | |
| BP | GO:0014013 | regulation of gliogenesis | 7/217 | 124/18670 | 0.000620477 | 0.008059618 | 0.005813147 | CCL3/CLCF1/IL1B/IL6/LIF/MIR221/TMEM98 | 7 | |
| BP | GO:0071356 | cellular response to tumor necrosis factor | 11/217 | 291/18670 | 0.000624264 | 0.008059618 | 0.005813147 | BIRC3/CCL2/CCL20/CCL3/CEBPA/HAS2/THBS1/TNFRSF12A/TNFSF14/ZC3H12A/ZFP36 | 11 | |
| BP | GO:0010574 | regulation of vascular endothelial growth factor production | 4/217 | 34/18670 | 0.00062624 | 0.008059618 | 0.005813147 | C5AR1/IL1B/IL6/PTGS2 | 4 | |
| BP | GO:0060795 | cell fate commitment involved in formation of primary germ layer | 4/217 | 34/18670 | 0.00062624 | 0.008059618 | 0.005813147 | ETS2/KLF4/SOX17/TRIM15 | 4 | |
| BP | GO:0042074 | cell migration involved in gastrulation | 3/217 | 15/18670 | 0.000635523 | 0.00810199 | 0.005843708 | AMOT/SOX17/WNT5A | 3 | |
| BP | GO:0010717 | regulation of epithelial to mesenchymal transition | 6/217 | 90/18670 | 0.000636 | 0.00810199 | 0.005843708 | FOXC1/IL1B/IL6/MIR21/MIR221/TGFB3 | 6 | |
| BP | GO:0045778 | positive regulation of ossification | 6/217 | 90/18670 | 0.000636 | 0.00810199 | 0.005843708 | CEBPA/CEBPD/IL6/MIR21/TGFB3/WNT5A | 6 | |
| BP | GO:0032640 | tumor necrosis factor production | 8/217 | 163/18670 | 0.000651491 | 0.008243451 | 0.00594574 | BCL3/CCL3/IRAK3/SPHK2/THBS1/WNT5A/ZC3H12A/ZFP36 | 8 | |
| BP | GO:1903555 | regulation of tumor necrosis factor superfamily cytokine production | 8/217 | 163/18670 | 0.000651491 | 0.008243451 | 0.00594574 | BCL3/CCL3/CD274/IRAK3/THBS1/WNT5A/ZC3H12A/ZFP36 | 8 | |
| BP | GO:0002573 | myeloid leukocyte differentiation | 9/217 | 204/18670 | 0.000655416 | 0.008265284 | 0.005961487 | CCL3/CEBPA/EPHA2/FOS/JUN/JUNB/LIF/MYC/SBNO2 | 9 | |
| BP | GO:0042180 | cellular ketone metabolic process | 10/217 | 248/18670 | 0.000669945 | 0.00840751 | 0.00606407 | AFP/AVPR1A/CYP7A1/EGR1/IL1B/IRS2/MIR21/NR4A3/PTGS2/SIRT4 | 10 | |
| BP | GO:0014706 | striated muscle tissue development | 13/217 | 390/18670 | 0.000671169 | 0.00840751 | 0.00606407 | EGR1/FOS/FOXC1/KLF5/MAFF/MYH11/PIM1/RGS2/SIK1/SOX17/TBX3/TCAP/WNT5A | 13 | |
| BP | GO:0014909 | smooth muscle cell migration | 6/217 | 91/18670 | 0.000674393 | 0.00841983 | 0.006072956 | ADAMTS1/HAS2/MIR21/MIR221/NR4A3/SERPINE1 | 6 | |
| BP | GO:0001893 | maternal placenta development | 4/217 | 35/18670 | 0.000700658 | 0.008661428 | 0.006247213 | JUNB/LIF/PTGS2/STC1 | 4 | |
| BP | GO:0042092 | type 2 immune response | 4/217 | 35/18670 | 0.000700658 | 0.008661428 | 0.006247213 | BCL3/BCL6/IL4R/IL6 | 4 | |
| BP | GO:0045622 | regulation of T-helper cell differentiation | 4/217 | 35/18670 | 0.000700658 | 0.008661428 | 0.006247213 | BCL6/IL4R/MIR21/ZC3H12A | 4 | |
| BP | GO:0090303 | positive regulation of wound healing | 5/217 | 61/18670 | 0.000710131 | 0.008746922 | 0.006308878 | HBEGF/MIR221/SERPINE1/THBD/THBS1 | 5 | |
| BP | GO:0071383 | cellular response to steroid hormone stimulus | 10/217 | 250/18670 | 0.000712659 | 0.008746922 | 0.006308878 | DEFA1/FAM107A/FOXO1/GPAM/NR4A1/NR4A2/NR4A3/PAQR7/STC1/ZFP36 | 10 | |
| BP | GO:1904894 | positive regulation of STAT cascade | 6/217 | 92/18670 | 0.000714557 | 0.008746922 | 0.006308878 | CLCF1/IL6/LIF/MIR221/SOCS1/SOCS3 | 6 | |
| BP | GO:0045637 | regulation of myeloid cell differentiation | 10/217 | 251/18670 | 0.000734844 | 0.008966046 | 0.006466925 | CCL3/FOS/JUN/LIF/MIR221/MYC/NFE2/NR4A3/THBS1/ZFP36 | 10 | |
| BP | GO:0050670 | regulation of lymphocyte proliferation | 9/217 | 208/18670 | 0.000752784 | 0.009125687 | 0.006582069 | BCL6/CD274/CLCF1/IGFBP2/IL1B/IL6/IRS2/MIR21/SDC4 | 9 | |
| BP | GO:0050792 | regulation of viral process | 9/217 | 208/18670 | 0.000752784 | 0.009125687 | 0.006582069 | APOBEC3A/CCL3/JUN/MIR221/PTX3/TMPRSS2/TRIM15/ZC3H12A/ZFP36 | 9 | |
| BP | GO:0046634 | regulation of alpha-beta T cell activation | 6/217 | 93/18670 | 0.000756547 | 0.009141811 | 0.006593699 | BCL6/CD274/IL4R/MIR21/SOCS1/ZC3H12A | 6 | |
| BP | GO:0070661 | leukocyte proliferation | 11/217 | 298/18670 | 0.000759162 | 0.009144007 | 0.006595283 | BCL6/CD274/CLCF1/IGFBP2/IL1B/IL6/IRS2/MIR21/SDC4/SLC11A1/TNFSF14 | 11 | |
| BP | GO:0010934 | macrophage cytokine production | 3/217 | 16/18670 | 0.000775501 | 0.009281315 | 0.006694318 | IRAK3/TGFB3/WNT5A | 3 | |
| BP | GO:0045623 | negative regulation of T-helper cell differentiation | 3/217 | 16/18670 | 0.000775501 | 0.009281315 | 0.006694318 | BCL6/IL4R/ZC3H12A | 3 | |
| BP | GO:0032944 | regulation of mononuclear cell proliferation | 9/217 | 209/18670 | 0.000778884 | 0.009283947 | 0.006696216 | BCL6/CD274/CLCF1/IGFBP2/IL1B/IL6/IRS2/MIR21/SDC4 | 9 | |
| BP | GO:0010573 | vascular endothelial growth factor production | 4/217 | 36/18670 | 0.000781123 | 0.009283947 | 0.006696216 | C5AR1/IL1B/IL6/PTGS2 | 4 | |
| BP | GO:0002791 | regulation of peptide secretion | 15/217 | 500/18670 | 0.000783132 | 0.009283947 | 0.006696216 | CCL3/CD274/IL1B/IL1RL1/IL4R/IL6/IRS2/PIM3/S100A8/SAA1/SIRT4/SOCS1/TGFB3/WNT5A/ZC3H12A | 15 | |
| BP | GO:0034614 | cellular response to reactive oxygen species | 8/217 | 168/18670 | 0.000793927 | 0.009382323 | 0.006767172 | FOS/FOXO1/IL18RAP/IL6/JUN/KLF4/MIR21/NR4A3 | 8 | |
| BP | GO:0050707 | regulation of cytokine secretion | 9/217 | 210/18670 | 0.000805716 | 0.00949179 | 0.006846127 | CCL3/CD274/IL1B/IL1RL1/IL4R/SAA1/SOCS1/WNT5A/ZC3H12A | 9 | |
| BP | GO:0046637 | regulation of alpha-beta T cell differentiation | 5/217 | 63/18670 | 0.000823421 | 0.009639923 | 0.006952971 | BCL6/IL4R/MIR21/SOCS1/ZC3H12A | 5 | |
| BP | GO:2000401 | regulation of lymphocyte migration | 5/217 | 63/18670 | 0.000823421 | 0.009639923 | 0.006952971 | CCL2/CCL20/CCL3/TNFSF14/WNT5A | 5 | |
| BP | GO:1903532 | positive regulation of secretion by cell | 13/217 | 399/18670 | 0.000828056 | 0.009664077 | 0.006970392 | AVPR1A/CCL3/CD274/IL1B/IL1RL1/IL4R/IL6/IRS2/SAA1/SDC4/SPHK2/TGFB3/WNT5A | 13 | |
| BP | GO:0002702 | positive regulation of production of molecular mediator of immune response | 6/217 | 95/18670 | 0.000846229 | 0.009788877 | 0.007060407 | CLCF1/IL1B/IL4R/IL6/NR4A3/WNT5A | 6 | |
| BP | GO:0044070 | regulation of anion transport | 6/217 | 95/18670 | 0.000846229 | 0.009788877 | 0.007060407 | AVPR1A/IL1B/IRS2/RGS2/STC1/THBS1 | 6 | |
| BP | GO:0034599 | cellular response to oxidative stress | 11/217 | 302/18670 | 0.000846563 | 0.009788877 | 0.007060407 | FOS/FOXO1/IL18RAP/IL6/JUN/KLF4/MIR21/NCOA7/NR4A2/NR4A3/ZC3H12A | 11 | |
| BP | GO:0034405 | response to fluid shear stress | 4/217 | 37/18670 | 0.0008679 | 0.009974215 | 0.007194086 | HAS2/KLF4/PTGS2/TGFB3 | 4 | |
| BP | GO:2000403 | positive regulation of lymphocyte migration | 4/217 | 37/18670 | 0.0008679 | 0.009974215 | 0.007194086 | CCL20/CCL3/TNFSF14/WNT5A | 4 | |
| BP | GO:0033673 | negative regulation of kinase activity | 10/217 | 257/18670 | 0.000880238 | 0.010076766 | 0.007268052 | CEBPA/EPHA1/GADD45B/GPRC5A/IL1B/IRAK3/IRS2/RGS2/SOCS1/SOCS3 | 10 | |
| BP | GO:0046834 | lipid phosphorylation | 5/217 | 64/18670 | 0.000884868 | 0.010076766 | 0.007268052 | CISH/SOCS1/SOCS2/SOCS3/SPHK2 | 5 | |
| BP | GO:0060135 | maternal process involved in female pregnancy | 5/217 | 64/18670 | 0.000884868 | 0.010076766 | 0.007268052 | JUNB/LIF/PTGS2/RGS2/STC1 | 5 | |
| BP | GO:0006937 | regulation of muscle contraction | 8/217 | 171/18670 | 0.000890749 | 0.01008264 | 0.007272289 | CNN1/MIR21/MYBPH/PROK2/PTGS2/RGS2/STC1/ZC3H12A | 8 | |
| BP | GO:0043901 | negative regulation of multi-organism process | 8/217 | 171/18670 | 0.000890749 | 0.01008264 | 0.007272289 | APOBEC3A/CCL3/JUN/MIR221/PTX3/TRIM15/ZC3H12A/ZFP36 | 8 | |
| BP | GO:0008585 | female gonad development | 6/217 | 96/18670 | 0.000894035 | 0.010089435 | 0.007277189 | ADAMTS1/AFP/ARID5B/FOXC1/MMP19/PTX3 | 6 | |
| BP | GO:0003007 | heart morphogenesis | 10/217 | 259/18670 | 0.000933656 | 0.010505027 | 0.007576943 | ADAMTS1/FOXC1/HAS2/JUN/MIR21/PIM1/SOX17/TBX3/TCAP/WNT5A | 10 | |
| BP | GO:0006940 | regulation of smooth muscle contraction | 5/217 | 65/18670 | 0.000949664 | 0.010653249 | 0.007683851 | CNN1/MIR21/PROK2/PTGS2/RGS2 | 5 | |
| BP | GO:0060537 | muscle tissue development | 13/217 | 408/18670 | 0.001014705 | 0.011284233 | 0.00813896 | EGR1/FOS/FOXC1/KLF5/MAFF/MYH11/PIM1/RGS2/SIK1/SOX17/TBX3/TCAP/WNT5A | 13 | |
| BP | GO:0071241 | cellular response to inorganic substance | 9/217 | 217/18670 | 0.001015381 | 0.011284233 | 0.00813896 | CEBPA/FOS/FOSB/FOXO1/JUN/JUNB/MT1A/PTGS2/WNT5A | 9 | |
| BP | GO:0045428 | regulation of nitric oxide biosynthetic process | 5/217 | 66/18670 | 0.001017923 | 0.011284233 | 0.00813896 | IL1B/KLF4/PTGS2/PTX3/ZC3H12A | 5 | |
| BP | GO:0046626 | regulation of insulin receptor signaling pathway | 5/217 | 66/18670 | 0.001017923 | 0.011284233 | 0.00813896 | CISH/IL1B/SOCS1/SOCS2/SOCS3 | 5 | |
| BP | GO:0050921 | positive regulation of chemotaxis | 7/217 | 135/18670 | 0.001025176 | 0.01133121 | 0.008172842 | C5AR1/CCL3/IL6/SERPINE1/THBS1/TNFSF14/WNT5A | 7 | |
| BP | GO:0070301 | cellular response to hydrogen peroxide | 6/217 | 99/18670 | 0.00105001 | 0.011571668 | 0.008346278 | FOXO1/IL18RAP/IL6/KLF4/MIR21/NR4A3 | 6 | |
| BP | GO:0007517 | muscle organ development | 13/217 | 410/18670 | 0.001060647 | 0.011654717 | 0.008406178 | ARID5B/EGR1/FOS/FOXC1/HBEGF/KLF5/LIF/MAFF/PIM1/RGS2/SOX17/TCAP/WNT5A | 13 | |
| BP | GO:0043583 | ear development | 9/217 | 219/18670 | 0.001082789 | 0.011863324 | 0.00855664 | ALDH1A3/CEBPA/CEBPD/LRIG3/NR4A3/SDC4/TCAP/TGFB3/WNT5A | 9 | |
| BP | GO:0002824 | positive regulation of adaptive immune response based on somatic recombination of immune receptors built from immunoglobulin superfamily domains | 6/217 | 100/18670 | 0.001106387 | 0.012016833 | 0.008667361 | CD274/CLCF1/IL1B/IL6/MIR21/SLC11A1 | 6 | |
| BP | GO:0070498 | interleukin-1-mediated signaling pathway | 6/217 | 100/18670 | 0.001106387 | 0.012016833 | 0.008667361 | EGR1/IL1B/IL1RN/IL6/IRAK3/MAP3K8 | 6 | |
| BP | GO:0035994 | response to muscle stretch | 3/217 | 18/18670 | 0.001110809 | 0.012016833 | 0.008667361 | FOS/JUN/TCAP | 3 | |
| BP | GO:0150078 | positive regulation of neuroinflammatory response | 3/217 | 18/18670 | 0.001110809 | 0.012016833 | 0.008667361 | CCL3/IL1B/IL6 | 3 | |
| BP | GO:0001959 | regulation of cytokine-mediated signaling pathway | 8/217 | 177/18670 | 0.001112788 | 0.012016833 | 0.008667361 | BIRC3/IL1RN/IL6/IRAK3/MIR21/SOCS1/SOCS3/WNT5A | 8 | |
| BP | GO:0046545 | development of primary female sexual characteristics | 6/217 | 101/18670 | 0.001165056 | 0.012407311 | 0.008949001 | ADAMTS1/AFP/ARID5B/FOXC1/MMP19/PTX3 | 6 | |
| BP | GO:0002369 | T cell cytokine production | 4/217 | 40/18670 | 0.001168757 | 0.012407311 | 0.008949001 | IL18RAP/IL1B/IL6/SLC11A1 | 4 | |
| BP | GO:0002823 | negative regulation of adaptive immune response based on somatic recombination of immune receptors built from immunoglobulin superfamily domains | 4/217 | 40/18670 | 0.001168757 | 0.012407311 | 0.008949001 | BCL6/IL1RL1/IL4R/ZC3H12A | 4 | |
| BP | GO:0042036 | negative regulation of cytokine biosynthetic process | 4/217 | 40/18670 | 0.001168757 | 0.012407311 | 0.008949001 | BCL3/IL6/KLF4/ZFP36 | 4 | |
| BP | GO:0098760 | response to interleukin-7 | 4/217 | 40/18670 | 0.001168757 | 0.012407311 | 0.008949001 | CISH/IRS2/SOCS1/SOCS2 | 4 | |
| BP | GO:0098761 | cellular response to interleukin-7 | 4/217 | 40/18670 | 0.001168757 | 0.012407311 | 0.008949001 | CISH/IRS2/SOCS1/SOCS2 | 4 | |
| BP | GO:0031098 | stress-activated protein kinase signaling cascade | 11/217 | 315/18670 | 0.001190222 | 0.012533584 | 0.009040077 | FOXO1/GADD45B/GADD45G/IL1B/IL1RN/MAP3K8/MYC/SBK2/WNT5A/ZC3H12A/ZFP36 | 11 | |
| BP | GO:0043903 | regulation of symbiosis, encompassing mutualism through parasitism | 9/217 | 222/18670 | 0.001190657 | 0.012533584 | 0.009040077 | APOBEC3A/CCL3/JUN/MIR221/PTX3/TMPRSS2/TRIM15/ZC3H12A/ZFP36 | 9 | |
| BP | GO:0070663 | regulation of leukocyte proliferation | 9/217 | 222/18670 | 0.001190657 | 0.012533584 | 0.009040077 | BCL6/CD274/CLCF1/IGFBP2/IL1B/IL6/IRS2/MIR21/SDC4 | 9 | |
| BP | GO:0050714 | positive regulation of protein secretion | 10/217 | 268/18670 | 0.001208047 | 0.012621133 | 0.009103223 | CCL3/CD274/IL1B/IL1RL1/IL4R/IL6/IRS2/SAA1/TGFB3/WNT5A | 10 | |
| BP | GO:0032102 | negative regulation of response to external stimulus | 12/217 | 365/18670 | 0.00120884 | 0.012621133 | 0.009103223 | CCL2/KLF4/MIR221/PLAUR/SAA1/SERPINE1/SOCS3/THBD/THBS1/TNFAIP6/WNT5A/ZC3H12A | 12 | |
| BP | GO:0001558 | regulation of cell growth | 13/217 | 416/18670 | 0.001209049 | 0.012621133 | 0.009103223 | AVPR1A/BCL6/CISH/FAM107A/HBEGF/IGFBP1/RGS2/S100A8/SOCS2/SOX17/SPHK2/TNFRSF12A/WNT5A | 13 | |
| BP | GO:0050715 | positive regulation of cytokine secretion | 7/217 | 139/18670 | 0.001215289 | 0.012651121 | 0.009124853 | CCL3/CD274/IL1B/IL1RL1/IL4R/SAA1/WNT5A | 7 | |
| BP | GO:2000379 | positive regulation of reactive oxygen species metabolic process | 6/217 | 102/18670 | 0.001226079 | 0.012728191 | 0.009180441 | IL1B/KLF4/PTGS2/PTX3/THBS1/ZC3H12A | 6 | |
| BP | GO:0090279 | regulation of calcium ion import | 4/217 | 41/18670 | 0.001283437 | 0.013250427 | 0.009557113 | CCL2/CCL3/SPHK2/STC1 | 4 | |
| BP | GO:0150077 | regulation of neuroinflammatory response | 4/217 | 41/18670 | 0.001283437 | 0.013250427 | 0.009557113 | CCL3/IL1B/IL6/PTGS2 | 4 | |
| BP | GO:0050708 | regulation of protein secretion | 14/217 | 472/18670 | 0.001298719 | 0.013371464 | 0.009644413 | CCL3/CD274/IL1B/IL1RL1/IL4R/IL6/IRS2/PIM3/SAA1/SIRT4/SOCS1/TGFB3/WNT5A/ZC3H12A | 14 | |
| BP | GO:0001710 | mesodermal cell fate commitment | 3/217 | 19/18670 | 0.001307836 | 0.013391958 | 0.009659195 | KLF4/SOX17/TRIM15 | 3 | |
| BP | GO:0060252 | positive regulation of glial cell proliferation | 3/217 | 19/18670 | 0.001307836 | 0.013391958 | 0.009659195 | IL1B/IL6/MIR221 | 3 | |
| BP | GO:0035690 | cellular response to drug | 12/217 | 369/18670 | 0.00132595 | 0.013523362 | 0.009753973 | EGR1/FOXO1/IL18RAP/IL1B/IL6/KLF4/MIR21/MYC/NR4A3/PTGS2/SPHK2/ZC3H12A | 12 | |
| BP | GO:0072091 | regulation of stem cell proliferation | 5/217 | 70/18670 | 0.001327866 | 0.013523362 | 0.009753973 | ASPM/MIR221/PIM1/SOX17/TBX3 | 5 | |
| BP | GO:1902105 | regulation of leukocyte differentiation | 10/217 | 272/18670 | 0.001349513 | 0.013692835 | 0.009876208 | BCL6/CCL3/FOS/IL4R/JUN/LIF/MIR21/MYC/SOCS1/ZC3H12A | 10 | |
| BP | GO:0014812 | muscle cell migration | 6/217 | 104/18670 | 0.001355438 | 0.013692835 | 0.009876208 | ADAMTS1/HAS2/MIR21/MIR221/NR4A3/SERPINE1 | 6 | |
| BP | GO:0071887 | leukocyte apoptotic process | 6/217 | 104/18670 | 0.001355438 | 0.013692835 | 0.009876208 | BCL6/CD274/IL6/IRS2/NR4A3/WNT5A | 6 | |
| BP | GO:0007568 | aging | 11/217 | 321/18670 | 0.001383754 | 0.013904134 | 0.010028611 | BCL2A1/BCL6/FOS/IGFBP1/IGFBP2/JUN/MIR21/PTGS2/SERPINE1/TBX3/TGFB3 | 11 | |
| BP | GO:0071375 | cellular response to peptide hormone stimulus | 11/217 | 321/18670 | 0.001383754 | 0.013904134 | 0.010028611 | CISH/FOXO1/IGFBP1/IL1B/IRS2/NR4A1/NR4A2/NR4A3/SOCS1/SOCS2/SOCS3 | 11 | |
| BP | GO:0010470 | regulation of gastrulation | 4/217 | 42/18670 | 0.001405756 | 0.014087546 | 0.010160901 | IL1RN/KLF4/MIR221/SOX17 | 4 | |
| BP | GO:0042531 | positive regulation of tyrosine phosphorylation of STAT protein | 5/217 | 71/18670 | 0.001415158 | 0.014144053 | 0.010201657 | CLCF1/IL6/LIF/MIR221/SOCS3 | 5 | |
| BP | GO:0002821 | positive regulation of adaptive immune response | 6/217 | 105/18670 | 0.001423899 | 0.014171982 | 0.010221801 | CD274/CLCF1/IL1B/IL6/MIR21/SLC11A1 | 6 | |
| BP | GO:0042098 | T cell proliferation | 8/217 | 184/18670 | 0.001425495 | 0.014171982 | 0.010221801 | CD274/IGFBP2/IL1B/IL6/MIR21/SDC4/SLC11A1/TNFSF14 | 8 | |
| BP | GO:0030879 | mammary gland development | 7/217 | 143/18670 | 0.001431985 | 0.014198947 | 0.01024125 | ELF3/EPHA2/IRS2/SOCS2/TBX3/TGFB3/WNT5A | 7 | |
| BP | GO:0010594 | regulation of endothelial cell migration | 9/217 | 229/18670 | 0.001476352 | 0.014600348 | 0.010530768 | EPHA2/KLF4/MIR21/MIR221/PTGS2/STC1/THBS1/WNT5A/ZC3H12A | 9 | |
| BP | GO:1902107 | positive regulation of leukocyte differentiation | 7/217 | 144/18670 | 0.001490601 | 0.014666674 | 0.010578607 | BCL6/FOS/IL4R/JUN/LIF/MIR21/SOCS1 | 7 | |
| BP | GO:0043087 | regulation of GTPase activity | 14/217 | 479/18670 | 0.001490865 | 0.014666674 | 0.010578607 | AMOT/BCL6/CCL2/CCL20/CCL3/EPHA1/EPHA2/JUN/MIR21/RGS1/RGS16/RGS2/SIPA1L2/WNT5A | 14 | |
| BP | GO:0042136 | neurotransmitter biosynthetic process | 6/217 | 106/18670 | 0.001494966 | 0.014668618 | 0.010580009 | HDC/IL1B/KLF4/PTGS2/PTX3/ZC3H12A | 6 | |
| BP | GO:0002532 | production of molecular mediator involved in inflammatory response | 5/217 | 72/18670 | 0.001506607 | 0.014737513 | 0.010629701 | IL4R/MIR21/MIR221/SERPINE1/ZC3H12A | 5 | |
| BP | GO:2001234 | negative regulation of apoptotic signaling pathway | 9/217 | 230/18670 | 0.00152132 | 0.014737513 | 0.010629701 | BCL2A1/IL1B/MIR21/MIR221/NR4A2/PLAUR/PTGS2/SERPINE1/THBS1 | 9 | |
| BP | GO:0006525 | arginine metabolic process | 3/217 | 20/18670 | 0.001525517 | 0.014737513 | 0.010629701 | MIR21/NAGS/PADI4 | 3 | |
| BP | GO:0033005 | positive regulation of mast cell activation | 3/217 | 20/18670 | 0.001525517 | 0.014737513 | 0.010629701 | IL4R/NR4A3/SPHK2 | 3 | |
| BP | GO:0043371 | negative regulation of CD4-positive, alpha-beta T cell differentiation | 3/217 | 20/18670 | 0.001525517 | 0.014737513 | 0.010629701 | BCL6/IL4R/ZC3H12A | 3 | |
| BP | GO:1905523 | positive regulation of macrophage migration | 3/217 | 20/18670 | 0.001525517 | 0.014737513 | 0.010629701 | C5AR1/CCL3/THBS1 | 3 | |
| BP | GO:0001953 | negative regulation of cell-matrix adhesion | 4/217 | 43/18670 | 0.001535978 | 0.014762669 | 0.010647845 | BCL6/FAM107A/SERPINE1/THBS1 | 4 | |
| BP | GO:0045429 | positive regulation of nitric oxide biosynthetic process | 4/217 | 43/18670 | 0.001535978 | 0.014762669 | 0.010647845 | IL1B/KLF4/PTGS2/PTX3 | 4 | |
| BP | GO:0043502 | regulation of muscle adaptation | 6/217 | 107/18670 | 0.001568703 | 0.01503874 | 0.010846967 | FOXO1/KLF4/MIR21/MTMR4/NR4A3/RGS2 | 6 | |
| BP | GO:0060485 | mesenchyme development | 10/217 | 278/18670 | 0.001586774 | 0.015173278 | 0.010944004 | ACTG2/FOXC1/HAS2/IL1B/IL6/MIR21/MIR221/MYC/TGFB3/WNT5A | 10 | |
| BP | GO:0051250 | negative regulation of lymphocyte activation | 7/217 | 146/18670 | 0.001613437 | 0.015360794 | 0.011079254 | BCL6/CD274/IL4R/MIR21/SDC4/SOCS1/ZC3H12A | 7 | |
| BP | GO:0000302 | response to reactive oxygen species | 9/217 | 232/18670 | 0.001614559 | 0.015360794 | 0.011079254 | FOS/FOSL1/FOXO1/IL18RAP/IL6/JUN/KLF4/MIR21/NR4A3 | 9 | |
| BP | GO:0014002 | astrocyte development | 4/217 | 44/18670 | 0.001674365 | 0.015841564 | 0.011426018 | C5AR1/IL1B/IL6/S100A8 | 4 | |
| BP | GO:1904407 | positive regulation of nitric oxide metabolic process | 4/217 | 44/18670 | 0.001674365 | 0.015841564 | 0.011426018 | IL1B/KLF4/PTGS2/PTX3 | 4 | |
| BP | GO:0043524 | negative regulation of neuron apoptotic process | 7/217 | 147/18670 | 0.001677739 | 0.015841564 | 0.011426018 | C5AR1/CCL2/CLCF1/JUN/NR4A2/NR4A3/TGFB3 | 7 | |
| BP | GO:0009064 | glutamine family amino acid metabolic process | 5/217 | 74/18670 | 0.001702451 | 0.015954642 | 0.011507578 | GFPT2/MIR21/NAGS/PADI4/SIRT4 | 5 | |
| BP | GO:0032677 | regulation of interleukin-8 production | 5/217 | 74/18670 | 0.001702451 | 0.015954642 | 0.011507578 | BCL3/IL1B/KLF4/SERPINE1/WNT5A | 5 | |
| BP | GO:1900076 | regulation of cellular response to insulin stimulus | 5/217 | 74/18670 | 0.001702451 | 0.015954642 | 0.011507578 | CISH/IL1B/SOCS1/SOCS2/SOCS3 | 5 | |
| BP | GO:0042742 | defense response to bacterium | 11/217 | 330/18670 | 0.001721927 | 0.016097019 | 0.01161027 | BCL3/C5AR1/CCL20/DEFA1/DEFB118/EPHA2/IL6/S100A12/S100A8/SERPINE1/SLC11A1 | 11 | |
| BP | GO:0061041 | regulation of wound healing | 7/217 | 148/18670 | 0.001744016 | 0.016134795 | 0.011637517 | HBEGF/MIR221/PLAUR/SERPINE1/THBD/THBS1/TNFRSF12A | 7 | |
| BP | GO:0071248 | cellular response to metal ion | 8/217 | 190/18670 | 0.001745807 | 0.016134795 | 0.011637517 | CEBPA/FOS/FOSB/JUN/JUNB/MT1A/PTGS2/WNT5A | 8 | |
| BP | GO:0006469 | negative regulation of protein kinase activity | 9/217 | 235/18670 | 0.00176297 | 0.016134795 | 0.011637517 | CEBPA/EPHA1/GADD45B/GPRC5A/IL1B/IRAK3/RGS2/SOCS1/SOCS3 | 9 | |
| BP | GO:0032682 | negative regulation of chemokine production | 3/217 | 21/18670 | 0.001764609 | 0.016134795 | 0.011637517 | EPHA2/IL6/KLF4 | 3 | |
| BP | GO:0032891 | negative regulation of organic acid transport | 3/217 | 21/18670 | 0.001764609 | 0.016134795 | 0.011637517 | IRS2/RGS2/THBS1 | 3 | |
| BP | GO:0035743 | CD4-positive, alpha-beta T cell cytokine production | 3/217 | 21/18670 | 0.001764609 | 0.016134795 | 0.011637517 | IL18RAP/IL1B/IL6 | 3 | |
| BP | GO:0036499 | PERK-mediated unfolded protein response | 3/217 | 21/18670 | 0.001764609 | 0.016134795 | 0.011637517 | CCL2/IGFBP1/PPP1R15A | 3 | |
| BP | GO:0071498 | cellular response to fluid shear stress | 3/217 | 21/18670 | 0.001764609 | 0.016134795 | 0.011637517 | HAS2/KLF4/PTGS2 | 3 | |
| BP | GO:0140131 | positive regulation of lymphocyte chemotaxis | 3/217 | 21/18670 | 0.001764609 | 0.016134795 | 0.011637517 | CCL3/TNFSF14/WNT5A | 3 | |
| BP | GO:0006939 | smooth muscle contraction | 6/217 | 110/18670 | 0.00180659 | 0.016478555 | 0.011885459 | CNN1/MIR21/MYH11/PROK2/PTGS2/RGS2 | 6 | |
| BP | GO:0051092 | positive regulation of NF-kappaB transcription factor activity | 7/217 | 149/18670 | 0.001812311 | 0.016490715 | 0.01189423 | IL18RAP/IL1B/IRAK3/S100A12/S100A8/TRIM15/WNT5A | 7 | |
| BP | GO:0002820 | negative regulation of adaptive immune response | 4/217 | 45/18670 | 0.00182118 | 0.016531391 | 0.011923569 | BCL6/IL1RL1/IL4R/ZC3H12A | 4 | |
| BP | GO:0043434 | response to peptide hormone | 13/217 | 436/18670 | 0.001834553 | 0.016612654 | 0.011982181 | CISH/EGR1/FOXO1/IGFBP1/IL1B/IRS2/NR4A1/NR4A2/NR4A3/PTGS2/SOCS1/SOCS2/SOCS3 | 13 | |
| BP | GO:0030258 | lipid modification | 9/217 | 238/18670 | 0.00192212 | 0.017322127 | 0.012493901 | CISH/IRS2/MTMR4/NR4A3/SIRT4/SOCS1/SOCS2/SOCS3/SPHK2 | 9 | |
| BP | GO:0051607 | defense response to virus | 9/217 | 238/18670 | 0.00192212 | 0.017322127 | 0.012493901 | APOBEC3A/BIRC3/DEFA1/IFIT2/IL1B/IL6/RTP4/TRIM15/ZC3H12A | 9 | |
| BP | GO:0051403 | stress-activated MAPK cascade | 10/217 | 286/18670 | 0.001954711 | 0.017487087 | 0.012612882 | FOXO1/GADD45B/GADD45G/IL1B/IL1RN/MAP3K8/MYC/WNT5A/ZC3H12A/ZFP36 | 10 | |
| BP | GO:0044003 | modification by symbiont of host morphology or physiology | 4/217 | 46/18670 | 0.001976684 | 0.017487087 | 0.012612882 | DEFA1/MIR221/SERPINB9/ZC3H12A | 4 | |
| BP | GO:0060443 | mammary gland morphogenesis | 4/217 | 46/18670 | 0.001976684 | 0.017487087 | 0.012612882 | ELF3/EPHA2/TBX3/WNT5A | 4 | |
| BP | GO:0071354 | cellular response to interleukin-6 | 4/217 | 46/18670 | 0.001976684 | 0.017487087 | 0.012612882 | CEBPA/IL6/SBNO2/SOCS3 | 4 | |
| BP | GO:1904738 | vascular associated smooth muscle cell migration | 4/217 | 46/18670 | 0.001976684 | 0.017487087 | 0.012612882 | ADAMTS1/MIR21/MIR221/NR4A3 | 4 | |
| BP | GO:1904752 | regulation of vascular associated smooth muscle cell migration | 4/217 | 46/18670 | 0.001976684 | 0.017487087 | 0.012612882 | ADAMTS1/MIR21/MIR221/NR4A3 | 4 | |
| BP | GO:0031348 | negative regulation of defense response | 9/217 | 239/18670 | 0.001977651 | 0.017487087 | 0.012612882 | IRAK3/KLF4/MIR21/MIR221/SAA1/SERPINB9/SOCS3/TNFAIP6/ZC3H12A | 9 | |
| BP | GO:0051402 | neuron apoptotic process | 9/217 | 239/18670 | 0.001977651 | 0.017487087 | 0.012612882 | C5AR1/CCL2/CCL3/CIT/CLCF1/JUN/NR4A2/NR4A3/TGFB3 | 9 | |
| BP | GO:0010226 | response to lithium ion | 3/217 | 22/18670 | 0.002025836 | 0.017780203 | 0.012824297 | CEBPA/IGFBP2/PTGS2 | 3 | |
| BP | GO:0045723 | positive regulation of fatty acid biosynthetic process | 3/217 | 22/18670 | 0.002025836 | 0.017780203 | 0.012824297 | AVPR1A/IL1B/PTGS2 | 3 | |
| BP | GO:0001890 | placenta development | 7/217 | 152/18670 | 0.002029715 | 0.017780203 | 0.012824297 | CEBPA/FOSL1/JUNB/LIF/PTGS2/SOCS3/STC1 | 7 | |
| BP | GO:0006809 | nitric oxide biosynthetic process | 5/217 | 77/18670 | 0.002030378 | 0.017780203 | 0.012824297 | IL1B/KLF4/PTGS2/PTX3/ZC3H12A | 5 | |
| BP | GO:0030217 | T cell differentiation | 9/217 | 240/18670 | 0.002034456 | 0.017780203 | 0.012824297 | BCL3/BCL6/EGR1/IL4R/IL6/MIR21/SOCS1/TMEM98/ZC3H12A | 9 | |
| BP | GO:0042119 | neutrophil activation | 14/217 | 498/18670 | 0.002135557 | 0.01858286 | 0.013403228 | C5AR1/CRISPLD2/DEFA1/FPR1/IL18RAP/PLAUR/PRSS3/PTX3/S100A12/S100A8/S100P/SLC11A1/SLC2A3/TNFAIP6 | 14 | |
| BP | GO:0006953 | acute-phase response | 4/217 | 47/18670 | 0.002141133 | 0.01858286 | 0.013403228 | IL1B/IL6/PTGS2/SAA1 | 4 | |
| BP | GO:1900744 | regulation of p38MAPK cascade | 4/217 | 47/18670 | 0.002141133 | 0.01858286 | 0.013403228 | GADD45B/GADD45G/IL1B/ZC3H12A | 4 | |
| BP | GO:0042063 | gliogenesis | 10/217 | 290/18670 | 0.00216295 | 0.018728957 | 0.013508603 | C5AR1/CCL2/CCL3/CLCF1/IL1B/IL6/LIF/MIR221/S100A8/TMEM98 | 10 | |
| BP | GO:0002446 | neutrophil mediated immunity | 14/217 | 499/18670 | 0.00217505 | 0.018790433 | 0.013552944 | C5AR1/CRISPLD2/DEFA1/FPR1/IL6/PLAUR/PRSS3/PTX3/S100A12/S100A8/S100P/SLC11A1/SLC2A3/TNFAIP6 | 14 | |
| BP | GO:0032612 | interleukin-1 production | 6/217 | 115/18670 | 0.002262634 | 0.019473509 | 0.014045625 | CCL3/EGR1/IL1B/SAA1/WNT5A/ZC3H12A | 6 | |
| BP | GO:0001570 | vasculogenesis | 5/217 | 79/18670 | 0.002273177 | 0.019473509 | 0.014045625 | AMOT/EPHA2/HAS2/JUNB/SOX17 | 5 | |
| BP | GO:0003151 | outflow tract morphogenesis | 5/217 | 79/18670 | 0.002273177 | 0.019473509 | 0.014045625 | FOXC1/JUN/SOX17/TBX3/WNT5A | 5 | |
| BP | GO:0051896 | regulation of protein kinase B signaling | 9/217 | 244/18670 | 0.002274846 | 0.019473509 | 0.014045625 | CCL3/EPHA2/HBEGF/IRS2/KLF4/MIR21/MIR221/THBS1/TNFAIP8L3 | 9 | |
| BP | GO:0071425 | hematopoietic stem cell proliferation | 3/217 | 23/18670 | 0.002309889 | 0.019592266 | 0.014131281 | MIR221/PIM1/WNT5A | 3 | |
| BP | GO:0072215 | regulation of metanephros development | 3/217 | 23/18670 | 0.002309889 | 0.019592266 | 0.014131281 | EGR1/LIF/MYC | 3 | |
| BP | GO:0001774 | microglial cell activation | 4/217 | 48/18670 | 0.002314786 | 0.019592266 | 0.014131281 | C5AR1/CCL3/IL6/JUN | 4 | |
| BP | GO:0002269 | leukocyte activation involved in inflammatory response | 4/217 | 48/18670 | 0.002314786 | 0.019592266 | 0.014131281 | C5AR1/CCL3/IL6/JUN | 4 | |
| BP | GO:0045933 | positive regulation of muscle contraction | 4/217 | 48/18670 | 0.002314786 | 0.019592266 | 0.014131281 | MIR21/PROK2/PTGS2/RGS2 | 4 | |
| BP | GO:0050866 | negative regulation of cell activation | 8/217 | 199/18670 | 0.002330239 | 0.019593495 | 0.014132167 | BCL6/CD274/IL4R/MIR21/SDC4/SOCS1/THBD/ZC3H12A | 8 | |
| BP | GO:0051146 | striated muscle cell differentiation | 10/217 | 293/18670 | 0.002330573 | 0.019593495 | 0.014132167 | AVPR1A/CHRNB1/IL4R/KLF5/MYH11/RGS2/SIK1/TBX3/TCAP/TNFSF14 | 10 | |
| BP | GO:0072001 | renal system development | 10/217 | 293/18670 | 0.002330573 | 0.019593495 | 0.014132167 | ADAMTS1/ARID5B/EGR1/FOXC1/HAS2/LIF/MYC/SDC4/SOX17/WNT5A | 10 | |
| BP | GO:0019827 | stem cell population maintenance | 7/217 | 157/18670 | 0.00243633 | 0.020436889 | 0.014740481 | ASPM/EPHA1/FOXO1/KLF4/LIF/PADI4/TBX3 | 7 | |
| BP | GO:0006979 | response to oxidative stress | 13/217 | 451/18670 | 0.002462151 | 0.020577847 | 0.014842149 | FOS/FOSL1/FOXO1/IL18RAP/IL6/JUN/KLF4/MIR21/NCOA7/NR4A2/NR4A3/PTGS2/ZC3H12A | 13 | |
| BP | GO:1901222 | regulation of NIK/NF-kappaB signaling | 6/217 | 117/18670 | 0.002467478 | 0.020577847 | 0.014842149 | BCL3/IL1B/LRRC19/MIR21/TNFSF14/ZC3H12A | 6 | |
| BP | GO:0097237 | cellular response to toxic substance | 9/217 | 247/18670 | 0.002469561 | 0.020577847 | 0.014842149 | EGR1/FOXO1/IL18RAP/IL6/KLF4/MIR21/NR4A3/PTGS2/ZC3H12A | 9 | |
| BP | GO:0002703 | regulation of leukocyte mediated immunity | 8/217 | 201/18670 | 0.002478844 | 0.020609507 | 0.014864984 | BCL6/CLCF1/IL18RAP/IL1B/IL4R/IL6/SERPINB9/SPHK2 | 8 | |
| BP | GO:0002448 | mast cell mediated immunity | 4/217 | 49/18670 | 0.002497897 | 0.020722071 | 0.014946173 | IL4R/NR4A3/SERPINB9/SPHK2 | 4 | |
| BP | GO:0060761 | negative regulation of response to cytokine stimulus | 5/217 | 81/18670 | 0.002536455 | 0.020995594 | 0.015143456 | IL1RN/IL6/IRAK3/KLF4/MIR21 | 5 | |
| BP | GO:0098727 | maintenance of cell number | 7/217 | 159/18670 | 0.002615481 | 0.021570784 | 0.015558323 | ASPM/EPHA1/FOXO1/KLF4/LIF/PADI4/TBX3 | 7 | |
| BP | GO:0032897 | negative regulation of viral transcription | 3/217 | 24/18670 | 0.002617424 | 0.021570784 | 0.015558323 | CCL3/JUN/ZFP36 | 3 | |
| BP | GO:0007178 | transmembrane receptor protein serine/threonine kinase signaling pathway | 11/217 | 349/18670 | 0.002659246 | 0.021867496 | 0.015772332 | AFP/EGR1/FOS/HIVEP1/JUN/MIR21/MTMR4/PEG10/TGFB3/THBS1/WNT5A | 11 | |
| BP | GO:0046209 | nitric oxide metabolic process | 5/217 | 82/18670 | 0.002676079 | 0.021934324 | 0.015820533 | IL1B/KLF4/PTGS2/PTX3/ZC3H12A | 5 | |
| BP | GO:0001952 | regulation of cell-matrix adhesion | 6/217 | 119/18670 | 0.002685963 | 0.021934324 | 0.015820533 | BCL6/EPHA1/FAM107A/SDC4/SERPINE1/THBS1 | 6 | |
| BP | GO:0046854 | phosphatidylinositol phosphorylation | 4/217 | 50/18670 | 0.002690719 | 0.021934324 | 0.015820533 | CISH/SOCS1/SOCS2/SOCS3 | 4 | |
| BP | GO:0070741 | response to interleukin-6 | 4/217 | 50/18670 | 0.002690719 | 0.021934324 | 0.015820533 | CEBPA/IL6/SBNO2/SOCS3 | 4 | |
| BP | GO:0032680 | regulation of tumor necrosis factor production | 7/217 | 160/18670 | 0.00270877 | 0.022033671 | 0.015892189 | BCL3/CCL3/IRAK3/THBS1/WNT5A/ZC3H12A/ZFP36 | 7 | |
| BP | GO:0002698 | negative regulation of immune effector process | 6/217 | 120/18670 | 0.002800496 | 0.022730594 | 0.016394857 | BCL6/IL4R/IRAK3/SERPINB9/TGFB3/ZC3H12A | 6 | |
| BP | GO:0071277 | cellular response to calcium ion | 5/217 | 83/18670 | 0.002821189 | 0.022800062 | 0.016444962 | FOS/FOSB/JUN/JUNB/WNT5A | 5 | |
| BP | GO:2000106 | regulation of leukocyte apoptotic process | 5/217 | 83/18670 | 0.002821189 | 0.022800062 | 0.016444962 | BCL6/CD274/IRS2/NR4A3/WNT5A | 5 | |
| BP | GO:0050679 | positive regulation of epithelial cell proliferation | 8/217 | 206/18670 | 0.00288295 | 0.023249197 | 0.016768909 | C5AR1/HAS2/JUN/MIR21/MYC/NR4A1/NR4A3/WNT5A | 8 | |
| BP | GO:0048146 | positive regulation of fibroblast proliferation | 4/217 | 51/18670 | 0.002893503 | 0.023284332 | 0.01679425 | FOSL2/JUN/MYC/WNT5A | 4 | |
| BP | GO:0030098 | lymphocyte differentiation | 11/217 | 353/18670 | 0.002901315 | 0.02329731 | 0.016803611 | BCL3/BCL6/CLCF1/EGR1/IL4R/IL6/KLF6/MIR21/SOCS1/TMEM98/ZC3H12A | 11 | |
| BP | GO:0002053 | positive regulation of mesenchymal cell proliferation | 3/217 | 25/18670 | 0.002949065 | 0.023529911 | 0.016971379 | IRS2/MYC/WNT5A | 3 | |
| BP | GO:0002719 | negative regulation of cytokine production involved in immune response | 3/217 | 25/18670 | 0.002949065 | 0.023529911 | 0.016971379 | BCL6/IRAK3/TGFB3 | 3 | |
| BP | GO:0060444 | branching involved in mammary gland duct morphogenesis | 3/217 | 25/18670 | 0.002949065 | 0.023529911 | 0.016971379 | EPHA2/TBX3/WNT5A | 3 | |
| BP | GO:1901215 | negative regulation of neuron death | 8/217 | 208/18670 | 0.003058278 | 0.024349594 | 0.01756259 | C5AR1/CCL2/CLCF1/JUN/NCOA7/NR4A2/NR4A3/TGFB3 | 8 | |
| BP | GO:0006959 | humoral immune response | 11/217 | 356/18670 | 0.003094286 | 0.024577281 | 0.017726814 | BCL3/C5AR1/CCL2/DEFA1/DEFB118/IL1B/IL6/PRSS3/S100A12/S100A8/SLC11A1 | 11 | |
| BP | GO:0009620 | response to fungus | 4/217 | 52/18670 | 0.003106495 | 0.024577281 | 0.017726814 | DEFA1/PTX3/S100A12/S100A8 | 4 | |
| BP | GO:0072132 | mesenchyme morphogenesis | 4/217 | 52/18670 | 0.003106495 | 0.024577281 | 0.017726814 | ACTG2/FOXC1/MYC/WNT5A | 4 | |
| BP | GO:1903901 | negative regulation of viral life cycle | 5/217 | 85/18670 | 0.003128357 | 0.024646466 | 0.017776715 | APOBEC3A/MIR221/PTX3/TRIM15/ZC3H12A | 5 | |
| BP | GO:2001057 | reactive nitrogen species metabolic process | 5/217 | 85/18670 | 0.003128357 | 0.024646466 | 0.017776715 | IL1B/KLF4/PTGS2/PTX3/ZC3H12A | 5 | |
| BP | GO:0051701 | interaction with host | 8/217 | 209/18670 | 0.003149 | 0.024757201 | 0.017856585 | DEFA1/EPHA2/MIR221/PTX3/SERPINB9/TMPRSS2/TRIM15/ZC3H12A | 8 | |
| BP | GO:0043523 | regulation of neuron apoptotic process | 8/217 | 210/18670 | 0.003241803 | 0.025433606 | 0.018344454 | C5AR1/CCL2/CCL3/CLCF1/JUN/NR4A2/NR4A3/TGFB3 | 8 | |
| BP | GO:0051101 | regulation of DNA binding | 6/217 | 124/18670 | 0.003295537 | 0.025664721 | 0.01851115 | BCL3/FOXC1/JUN/KLF4/LIF/PLAUR | 6 | |
| BP | GO:0030194 | positive regulation of blood coagulation | 3/217 | 26/18670 | 0.003305408 | 0.025664721 | 0.01851115 | SERPINE1/THBD/THBS1 | 3 | |
| BP | GO:0072539 | T-helper 17 cell differentiation | 3/217 | 26/18670 | 0.003305408 | 0.025664721 | 0.01851115 | IL6/MIR21/ZC3H12A | 3 | |
| BP | GO:1900048 | positive regulation of hemostasis | 3/217 | 26/18670 | 0.003305408 | 0.025664721 | 0.01851115 | SERPINE1/THBD/THBS1 | 3 | |
| BP | GO:1903959 | regulation of anion transmembrane transport | 3/217 | 26/18670 | 0.003305408 | 0.025664721 | 0.01851115 | IRS2/RGS2/THBS1 | 3 | |
| BP | GO:0030195 | negative regulation of blood coagulation | 4/217 | 53/18670 | 0.003329942 | 0.025695937 | 0.018533665 | PLAUR/SERPINE1/THBD/THBS1 | 4 | |
| BP | GO:0050704 | regulation of interleukin-1 secretion | 4/217 | 53/18670 | 0.003329942 | 0.025695937 | 0.018533665 | CCL3/SAA1/WNT5A/ZC3H12A | 4 | |
| BP | GO:0070228 | regulation of lymphocyte apoptotic process | 4/217 | 53/18670 | 0.003329942 | 0.025695937 | 0.018533665 | BCL6/CD274/IRS2/WNT5A | 4 | |
| BP | GO:0035051 | cardiocyte differentiation | 7/217 | 167/18670 | 0.003435309 | 0.026454697 | 0.019080934 | MIR21/MYH11/RGS2/SIK1/SOX17/TBX3/TCAP | 7 | |
| BP | GO:0002720 | positive regulation of cytokine production involved in immune response | 4/217 | 54/18670 | 0.003564086 | 0.027278684 | 0.01967525 | IL1B/IL6/NR4A3/WNT5A | 4 | |
| BP | GO:0006636 | unsaturated fatty acid biosynthetic process | 4/217 | 54/18670 | 0.003564086 | 0.027278684 | 0.01967525 | AVPR1A/FADS1/IL1B/PTGS2 | 4 | |
| BP | GO:1900047 | negative regulation of hemostasis | 4/217 | 54/18670 | 0.003564086 | 0.027278684 | 0.01967525 | PLAUR/SERPINE1/THBD/THBS1 | 4 | |
| BP | GO:0048644 | muscle organ morphogenesis | 5/217 | 88/18670 | 0.003633312 | 0.027639647 | 0.019935602 | ARID5B/FOXC1/LIF/TCAP/WNT5A | 5 | |
| BP | GO:0050829 | defense response to Gram-negative bacterium | 5/217 | 88/18670 | 0.003633312 | 0.027639647 | 0.019935602 | DEFA1/DEFB118/IL6/SERPINE1/SLC11A1 | 5 | |
| BP | GO:1904035 | regulation of epithelial cell apoptotic process | 5/217 | 88/18670 | 0.003633312 | 0.027639647 | 0.019935602 | CCL2/IL6/SERPINE1/THBS1/ZFP36 | 5 | |
| BP | GO:0010038 | response to metal ion | 11/217 | 364/18670 | 0.003659819 | 0.027758649 | 0.020021434 | CEBPA/FOS/FOSB/IGFBP2/JUN/JUNB/MT1A/PTGS2/S100A8/THBS1/WNT5A | 11 | |
| BP | GO:0045932 | negative regulation of muscle contraction | 3/217 | 27/18670 | 0.003687016 | 0.027758649 | 0.020021434 | PTGS2/RGS2/ZC3H12A | 3 | |
| BP | GO:0048143 | astrocyte activation | 3/217 | 27/18670 | 0.003687016 | 0.027758649 | 0.020021434 | C5AR1/IL1B/IL6 | 3 | |
| BP | GO:0050820 | positive regulation of coagulation | 3/217 | 27/18670 | 0.003687016 | 0.027758649 | 0.020021434 | SERPINE1/THBD/THBS1 | 3 | |
| BP | GO:2000191 | regulation of fatty acid transport | 3/217 | 27/18670 | 0.003687016 | 0.027758649 | 0.020021434 | IL1B/IRS2/THBS1 | 3 | |
| BP | GO:0051222 | positive regulation of protein transport | 12/217 | 418/18670 | 0.003693274 | 0.027758649 | 0.020021434 | CCL3/CD274/IL1B/IL1RL1/IL4R/IL6/IRS2/PTGS2/SAA1/TGFB3/WNT5A/ZC3H12A | 12 | |
| BP | GO:0070374 | positive regulation of ERK1 and ERK2 cascade | 8/217 | 215/18670 | 0.003738164 | 0.028039963 | 0.020224336 | C5AR1/CCL2/CCL20/CCL3/JUN/MIR21/MIR221/TNFAIP8L3 | 8 | |
| BP | GO:0042304 | regulation of fatty acid biosynthetic process | 4/217 | 55/18670 | 0.003809166 | 0.028458938 | 0.02052653 | AVPR1A/CYP7A1/IL1B/PTGS2 | 4 | |
| BP | GO:0046456 | icosanoid biosynthetic process | 4/217 | 55/18670 | 0.003809166 | 0.028458938 | 0.02052653 | AVPR1A/FADS1/IL1B/PTGS2 | 4 | |
| BP | GO:0032869 | cellular response to insulin stimulus | 8/217 | 216/18670 | 0.003844133 | 0.028663198 | 0.020673856 | CISH/FOXO1/IGFBP1/IL1B/IRS2/SOCS1/SOCS2/SOCS3 | 8 | |
| BP | GO:0010595 | positive regulation of endothelial cell migration | 6/217 | 128/18670 | 0.003853048 | 0.028672781 | 0.020680768 | MIR21/MIR221/PTGS2/THBS1/WNT5A/ZC3H12A | 6 | |
| BP | GO:0008406 | gonad development | 8/217 | 217/18670 | 0.003952411 | 0.029296172 | 0.0211304 | ADAMTS1/AFP/ARID5B/ASPM/FOXC1/MMP19/PTX3/WNT5A | 8 | |
| BP | GO:0050920 | regulation of chemotaxis | 8/217 | 217/18670 | 0.003952411 | 0.029296172 | 0.0211304 | C5AR1/CCL2/CCL3/IL6/SERPINE1/THBS1/TNFSF14/WNT5A | 8 | |
| BP | GO:0001516 | prostaglandin biosynthetic process | 3/217 | 28/18670 | 0.00409442 | 0.030052407 | 0.021675849 | AVPR1A/IL1B/PTGS2 | 3 | |
| BP | GO:0046457 | prostanoid biosynthetic process | 3/217 | 28/18670 | 0.00409442 | 0.030052407 | 0.021675849 | AVPR1A/IL1B/PTGS2 | 3 | |
| BP | GO:1903792 | negative regulation of anion transport | 3/217 | 28/18670 | 0.00409442 | 0.030052407 | 0.021675849 | IRS2/RGS2/THBS1 | 3 | |
| BP | GO:1904994 | regulation of leukocyte adhesion to vascular endothelial cell | 3/217 | 28/18670 | 0.00409442 | 0.030052407 | 0.021675849 | KLF4/MIR21/MIR221 | 3 | |
| BP | GO:2000108 | positive regulation of leukocyte apoptotic process | 3/217 | 28/18670 | 0.00409442 | 0.030052407 | 0.021675849 | CD274/NR4A3/WNT5A | 3 | |
| BP | GO:0003018 | vascular process in circulatory system | 7/217 | 173/18670 | 0.004168829 | 0.030532106 | 0.02202184 | AVPR1A/C2CD4A/C2CD4B/FOXC1/MIR21/PTGS2/RGS2 | 7 | |
| BP | GO:0048762 | mesenchymal cell differentiation | 8/217 | 219/18670 | 0.004176025 | 0.030532106 | 0.02202184 | FOXC1/HAS2/IL1B/IL6/MIR21/MIR221/TGFB3/WNT5A | 8 | |
| BP | GO:0052548 | regulation of endopeptidase activity | 12/217 | 425/18670 | 0.004212766 | 0.030740923 | 0.022172454 | BIRC3/GRAMD4/KLF4/MIR21/MYC/PLAUR/PTGS2/S100A8/SERPINB9/SERPINE1/THBS1/TNFSF14 | 12 | |
| BP | GO:0002526 | acute inflammatory response | 8/217 | 220/18670 | 0.004291429 | 0.031254243 | 0.022542695 | C2CD4A/C2CD4B/C5AR1/IL1B/IL6/PTGS2/S100A8/SAA1 | 8 | |
| BP | GO:0006941 | striated muscle contraction | 7/217 | 174/18670 | 0.004301787 | 0.031269084 | 0.022553399 | CHRNB1/KCNE4/MYBPH/RGS2/STC1/TCAP/ZC3H12A | 7 | |
| BP | GO:0050819 | negative regulation of coagulation | 4/217 | 57/18670 | 0.00433308 | 0.031375169 | 0.022629915 | PLAUR/SERPINE1/THBD/THBS1 | 4 | |
| BP | GO:1903428 | positive regulation of reactive oxygen species biosynthetic process | 4/217 | 57/18670 | 0.00433308 | 0.031375169 | 0.022629915 | IL1B/KLF4/PTGS2/PTX3 | 4 | |
| BP | GO:0002695 | negative regulation of leukocyte activation | 7/217 | 175/18670 | 0.00443793 | 0.032029434 | 0.023101816 | BCL6/CD274/IL4R/MIR21/SDC4/SOCS1/ZC3H12A | 7 | |
| BP | GO:0007548 | sex differentiation | 9/217 | 270/18670 | 0.004440483 | 0.032029434 | 0.023101816 | ADAMTS1/AFP/ARID5B/ASPM/FOXC1/MMP19/PTX3/TBX3/WNT5A | 9 | |
| BP | GO:0016049 | cell growth | 13/217 | 484/18670 | 0.004473023 | 0.032173794 | 0.023205938 | AVPR1A/BCL6/CISH/FAM107A/HBEGF/IGFBP1/RGS2/S100A8/SOCS2/SOX17/SPHK2/TNFRSF12A/WNT5A | 13 | |
| BP | GO:0045995 | regulation of embryonic development | 6/217 | 132/18670 | 0.00447762 | 0.032173794 | 0.023205938 | AMOT/IL1RN/KLF4/MIR221/SOX17/WNT5A | 6 | |
| BP | GO:0007263 | nitric oxide mediated signal transduction | 3/217 | 29/18670 | 0.004528128 | 0.032228606 | 0.023245472 | FPR1/RASD1/THBS1 | 3 | |
| BP | GO:0044068 | modulation by symbiont of host cellular process | 3/217 | 29/18670 | 0.004528128 | 0.032228606 | 0.023245472 | MIR221/SERPINB9/ZC3H12A | 3 | |
| BP | GO:0044788 | modulation by host of viral process | 3/217 | 29/18670 | 0.004528128 | 0.032228606 | 0.023245472 | MIR221/PTX3/ZC3H12A | 3 | |
| BP | GO:0070102 | interleukin-6-mediated signaling pathway | 3/217 | 29/18670 | 0.004528128 | 0.032228606 | 0.023245472 | CEBPA/IL6/SOCS3 | 3 | |
| BP | GO:0072538 | T-helper 17 type immune response | 3/217 | 29/18670 | 0.004528128 | 0.032228606 | 0.023245472 | IL6/MIR21/ZC3H12A | 3 | |
| BP | GO:0043312 | neutrophil degranulation | 13/217 | 485/18670 | 0.004550116 | 0.032323887 | 0.023314195 | C5AR1/CRISPLD2/DEFA1/FPR1/PLAUR/PRSS3/PTX3/S100A12/S100A8/S100P/SLC11A1/SLC2A3/TNFAIP6 | 13 | |
| BP | GO:0051897 | positive regulation of protein kinase B signaling | 7/217 | 176/18670 | 0.004577306 | 0.032455693 | 0.023409263 | CCL3/HBEGF/IRS2/MIR21/MIR221/THBS1/TNFAIP8L3 | 7 | |
| BP | GO:0071385 | cellular response to glucocorticoid stimulus | 4/217 | 58/18670 | 0.004612376 | 0.03264277 | 0.023544196 | FAM107A/FOXO1/STC1/ZFP36 | 4 | |
| BP | GO:0002705 | positive regulation of leukocyte mediated immunity | 6/217 | 133/18670 | 0.004644782 | 0.032776015 | 0.023640301 | CLCF1/IL18RAP/IL1B/IL4R/IL6/SPHK2 | 6 | |
| BP | GO:0045137 | development of primary sexual characteristics | 8/217 | 223/18670 | 0.004652429 | 0.032776015 | 0.023640301 | ADAMTS1/AFP/ARID5B/ASPM/FOXC1/MMP19/PTX3/WNT5A | 8 | |
| BP | GO:0032868 | response to insulin | 9/217 | 272/18670 | 0.004657369 | 0.032776015 | 0.023640301 | CISH/EGR1/FOXO1/IGFBP1/IL1B/IRS2/SOCS1/SOCS2/SOCS3 | 9 | |
| BP | GO:0002283 | neutrophil activation involved in immune response | 13/217 | 488/18670 | 0.004787852 | 0.033631305 | 0.024257195 | C5AR1/CRISPLD2/DEFA1/FPR1/PLAUR/PRSS3/PTX3/S100A12/S100A8/S100P/SLC11A1/SLC2A3/TNFAIP6 | 13 | |
| BP | GO:1901216 | positive regulation of neuron death | 5/217 | 94/18670 | 0.004814419 | 0.033754824 | 0.024346285 | CCL3/EGR1/FOS/JUN/WNT5A | 5 | |
| BP | GO:0050678 | regulation of epithelial cell proliferation | 11/217 | 378/18670 | 0.004846592 | 0.033917118 | 0.024463343 | C5AR1/CCL2/HAS2/JUN/MIR21/MYC/NR4A1/NR4A3/THBS1/WNT5A/ZFP36 | 11 | |
| BP | GO:0032732 | positive regulation of interleukin-1 production | 4/217 | 59/18670 | 0.004903537 | 0.03420379 | 0.02467011 | CCL3/EGR1/SAA1/WNT5A | 4 | |
| BP | GO:0001649 | osteoblast differentiation | 8/217 | 225/18670 | 0.004905759 | 0.03420379 | 0.02467011 | CCL3/CEBPA/CEBPD/EPHA2/IL6/JUNB/MIR21/VCAN | 8 | |
| BP | GO:0050673 | epithelial cell proliferation | 12/217 | 434/18670 | 0.004965331 | 0.034461782 | 0.024856192 | C5AR1/CCL2/EPHA2/HAS2/JUN/MIR21/MYC/NR4A1/NR4A3/THBS1/WNT5A/ZFP36 | 12 | |
| BP | GO:0002828 | regulation of type 2 immune response | 3/217 | 30/18670 | 0.004988614 | 0.034461782 | 0.024856192 | BCL6/IL4R/IL6 | 3 | |
| BP | GO:0035066 | positive regulation of histone acetylation | 3/217 | 30/18670 | 0.004988614 | 0.034461782 | 0.024856192 | IL1B/LIF/SPHK2 | 3 | |
| BP | GO:0043372 | positive regulation of CD4-positive, alpha-beta T cell differentiation | 3/217 | 30/18670 | 0.004988614 | 0.034461782 | 0.024856192 | IL4R/MIR21/SOCS1 | 3 | |
| BP | GO:0046320 | regulation of fatty acid oxidation | 3/217 | 30/18670 | 0.004988614 | 0.034461782 | 0.024856192 | IRS2/NR4A3/SIRT4 | 3 | |
| BP | GO:0042102 | positive regulation of T cell proliferation | 5/217 | 95/18670 | 0.005034874 | 0.034653948 | 0.024994795 | CD274/IGFBP2/IL1B/IL6/MIR21 | 5 | |
| BP | GO:0055006 | cardiac cell development | 5/217 | 95/18670 | 0.005034874 | 0.034653948 | 0.024994795 | MIR21/MYH11/RGS2/TBX3/TCAP | 5 | |
| BP | GO:0043534 | blood vessel endothelial cell migration | 7/217 | 180/18670 | 0.005168098 | 0.035441078 | 0.025562527 | AMOT/EPHA2/KLF4/MIR221/NR4A1/PTGS2/THBS1 | 7 | |
| BP | GO:0070613 | regulation of protein processing | 7/217 | 180/18670 | 0.005168098 | 0.035441078 | 0.025562527 | BIRC3/C5AR1/IL1B/SERPINE1/SIRT4/THBS1/TMEM98 | 7 | |
| BP | GO:0045576 | mast cell activation | 4/217 | 60/18670 | 0.005206785 | 0.035641343 | 0.025706972 | IL4R/NR4A3/S100A12/SPHK2 | 4 | |
| BP | GO:0051147 | regulation of muscle cell differentiation | 7/217 | 181/18670 | 0.005324358 | 0.036379888 | 0.02623966 | CDH15/IL4R/MIR21/MIR221/RGS2/SIK1/TNFSF14 | 7 | |
| BP | GO:0001655 | urogenital system development | 10/217 | 330/18670 | 0.005391395 | 0.036771075 | 0.026521811 | ADAMTS1/ARID5B/EGR1/FOXC1/HAS2/LIF/MYC/SDC4/SOX17/WNT5A | 10 | |
| BP | GO:0048710 | regulation of astrocyte differentiation | 3/217 | 31/18670 | 0.005476327 | 0.036861371 | 0.026586939 | CLCF1/IL6/LIF | 3 | |
| BP | GO:1902895 | positive regulation of pri-miRNA transcription by RNA polymerase II | 3/217 | 31/18670 | 0.005476327 | 0.036861371 | 0.026586939 | FOS/JUN/KLF5 | 3 | |
| BP | GO:2000406 | positive regulation of T cell migration | 3/217 | 31/18670 | 0.005476327 | 0.036861371 | 0.026586939 | CCL20/TNFSF14/WNT5A | 3 | |
| BP | GO:1903317 | regulation of protein maturation | 7/217 | 182/18670 | 0.005484141 | 0.036861371 | 0.026586939 | BIRC3/C5AR1/IL1B/SERPINE1/SIRT4/THBS1/TMEM98 | 7 | |
| BP | GO:0010596 | negative regulation of endothelial cell migration | 5/217 | 97/18670 | 0.005497093 | 0.036861371 | 0.026586939 | KLF4/MIR21/MIR221/STC1/THBS1 | 5 | |
| BP | GO:0030316 | osteoclast differentiation | 5/217 | 97/18670 | 0.005497093 | 0.036861371 | 0.026586939 | CCL3/EPHA2/FOS/JUNB/SBNO2 | 5 | |
| BP | GO:1990868 | response to chemokine | 5/217 | 97/18670 | 0.005497093 | 0.036861371 | 0.026586939 | CCL2/CCL20/CCL3/FOXC1/ZC3H12A | 5 | |
| BP | GO:1990869 | cellular response to chemokine | 5/217 | 97/18670 | 0.005497093 | 0.036861371 | 0.026586939 | CCL2/CCL20/CCL3/FOXC1/ZC3H12A | 5 | |
| BP | GO:0034113 | heterotypic cell-cell adhesion | 4/217 | 61/18670 | 0.00552234 | 0.036861371 | 0.026586939 | IL1B/IL1RN/KLF4/MIR221 | 4 | |
| BP | GO:0050701 | interleukin-1 secretion | 4/217 | 61/18670 | 0.00552234 | 0.036861371 | 0.026586939 | CCL3/SAA1/WNT5A/ZC3H12A | 4 | |
| BP | GO:0071384 | cellular response to corticosteroid stimulus | 4/217 | 61/18670 | 0.00552234 | 0.036861371 | 0.026586939 | FAM107A/FOXO1/STC1/ZFP36 | 4 | |
| BP | GO:1905953 | negative regulation of lipid localization | 4/217 | 61/18670 | 0.00552234 | 0.036861371 | 0.026586939 | ABCG8/IL6/IRS2/THBS1 | 4 | |
| BP | GO:0061008 | hepaticobiliary system development | 6/217 | 138/18670 | 0.005550336 | 0.036982556 | 0.026674346 | ARID5B/CEBPA/CIT/DBP/JUN/SOX17 | 6 | |
| BP | GO:0009743 | response to carbohydrate | 8/217 | 230/18670 | 0.0055854 | 0.037150327 | 0.026795354 | CYP7A1/EGR1/IL1B/IRS2/MIR221/PIM3/PTGS2/THBS1 | 8 | |
| BP | GO:0038061 | NIK/NF-kappaB signaling | 7/217 | 183/18670 | 0.005647494 | 0.037257519 | 0.026872668 | BCL3/BIRC3/IL1B/LRRC19/MIR21/TNFSF14/ZC3H12A | 7 | |
| BP | GO:0001660 | fever generation | 2/217 | 10/18670 | 0.005690744 | 0.037257519 | 0.026872668 | IL1B/PTGS2 | 2 | |
| BP | GO:0015809 | arginine transport | 2/217 | 10/18670 | 0.005690744 | 0.037257519 | 0.026872668 | SLC11A1/SLC7A1 | 2 | |
| BP | GO:0048340 | paraxial mesoderm morphogenesis | 2/217 | 10/18670 | 0.005690744 | 0.037257519 | 0.026872668 | FOXC1/WNT5A | 2 | |
| BP | GO:0048570 | notochord morphogenesis | 2/217 | 10/18670 | 0.005690744 | 0.037257519 | 0.026872668 | EPHA2/WNT5A | 2 | |
| BP | GO:0072203 | cell proliferation involved in metanephros development | 2/217 | 10/18670 | 0.005690744 | 0.037257519 | 0.026872668 | EGR1/MYC | 2 | |
| BP | GO:0090009 | primitive streak formation | 2/217 | 10/18670 | 0.005690744 | 0.037257519 | 0.026872668 | ETS2/WNT5A | 2 | |
| BP | GO:0090037 | positive regulation of protein kinase C signaling | 2/217 | 10/18670 | 0.005690744 | 0.037257519 | 0.026872668 | SPHK2/WNT5A | 2 | |
| BP | GO:1902033 | regulation of hematopoietic stem cell proliferation | 2/217 | 10/18670 | 0.005690744 | 0.037257519 | 0.026872668 | MIR221/PIM1 | 2 | |
| BP | GO:0002042 | cell migration involved in sprouting angiogenesis | 5/217 | 98/18670 | 0.005739095 | 0.037427453 | 0.026995236 | KLF4/MIR221/NR4A1/PTGS2/THBS1 | 5 | |
| BP | GO:0042472 | inner ear morphogenesis | 5/217 | 98/18670 | 0.005739095 | 0.037427453 | 0.026995236 | ALDH1A3/LRIG3/NR4A3/TCAP/WNT5A | 5 | |
| BP | GO:0031667 | response to nutrient levels | 13/217 | 499/18670 | 0.005746578 | 0.037427453 | 0.026995236 | ABCG8/FADS1/FAM107A/FOXO1/IGFBP2/IL1B/JUN/PIM1/PTGS2/SIK1/STC1/ZC3H12A/ZFP36 | 13 | |
| BP | GO:0032970 | regulation of actin filament-based process | 11/217 | 388/18670 | 0.005867485 | 0.038148807 | 0.027515526 | CDC42EP3/CIT/EPHA1/FAM107A/FRMD6/KANK4/MIR21/RND1/SDC4/STC1/TGFB3 | 11 | |
| BP | GO:0060070 | canonical Wnt signaling pathway | 10/217 | 335/18670 | 0.005975192 | 0.038556114 | 0.027809303 | ASPM/EGR1/FOXO1/FRAT1/IGFBP1/IGFBP2/KLF4/NR4A2/SOX17/WNT5A | 10 | |
| BP | GO:0045589 | regulation of regulatory T cell differentiation | 3/217 | 32/18670 | 0.00599169 | 0.038556114 | 0.027809303 | BCL6/MIR21/SOCS1 | 3 | |
| BP | GO:0045907 | positive regulation of vasoconstriction | 3/217 | 32/18670 | 0.00599169 | 0.038556114 | 0.027809303 | AVPR1A/MIR21/PTGS2 | 3 | |
| BP | GO:0045987 | positive regulation of smooth muscle contraction | 3/217 | 32/18670 | 0.00599169 | 0.038556114 | 0.027809303 | MIR21/PROK2/PTGS2 | 3 | |
| BP | GO:0060325 | face morphogenesis | 3/217 | 32/18670 | 0.00599169 | 0.038556114 | 0.027809303 | ARID5B/CRISPLD2/TGFB3 | 3 | |
| BP | GO:0060674 | placenta blood vessel development | 3/217 | 32/18670 | 0.00599169 | 0.038556114 | 0.027809303 | FOSL1/JUNB/SOCS3 | 3 | |
| BP | GO:0048738 | cardiac muscle tissue development | 8/217 | 233/18670 | 0.006026327 | 0.03871271 | 0.027922251 | FOXC1/MYH11/PIM1/RGS2/SIK1/TBX3/TCAP/WNT5A | 8 | |
| BP | GO:0072593 | reactive oxygen species metabolic process | 9/217 | 284/18670 | 0.006137379 | 0.039358823 | 0.028388272 | FOXO1/IL1B/KLF4/MIR21/PTGS2/PTX3/SPHK2/THBS1/ZC3H12A | 9 | |
| BP | GO:0002637 | regulation of immunoglobulin production | 4/217 | 63/18670 | 0.006191231 | 0.039636533 | 0.028588575 | BCL6/CLCF1/IL4R/IL6 | 4 | |
| BP | GO:0032611 | interleukin-1 beta production | 5/217 | 101/18670 | 0.006510073 | 0.041407517 | 0.02986593 | CCL3/EGR1/IL1B/WNT5A/ZC3H12A | 5 | |
| BP | GO:0010464 | regulation of mesenchymal cell proliferation | 3/217 | 33/18670 | 0.006535099 | 0.041407517 | 0.02986593 | IRS2/MYC/WNT5A | 3 | |
| BP | GO:0010837 | regulation of keratinocyte proliferation | 3/217 | 33/18670 | 0.006535099 | 0.041407517 | 0.02986593 | HAS2/MIR21/ZFP36 | 3 | |
| BP | GO:0060603 | mammary gland duct morphogenesis | 3/217 | 33/18670 | 0.006535099 | 0.041407517 | 0.02986593 | EPHA2/TBX3/WNT5A | 3 | |
| BP | GO:2000758 | positive regulation of peptidyl-lysine acetylation | 3/217 | 33/18670 | 0.006535099 | 0.041407517 | 0.02986593 | IL1B/LIF/SPHK2 | 3 | |
| BP | GO:2001024 | negative regulation of response to drug | 3/217 | 33/18670 | 0.006535099 | 0.041407517 | 0.02986593 | MIR21/NR4A3/RGS2 | 3 | |
| BP | GO:0045669 | positive regulation of osteoblast differentiation | 4/217 | 64/18670 | 0.006544988 | 0.041407517 | 0.02986593 | CEBPA/CEBPD/IL6/MIR21 | 4 | |
| BP | GO:0070482 | response to oxygen levels | 11/217 | 394/18670 | 0.006556981 | 0.04141367 | 0.029870368 | APOLD1/EGR1/FOXO1/MIR21/MYC/NR4A2/PTGS2/SIRT4/STC1/TGFB3/THBS1 | 11 | |
| BP | GO:0031669 | cellular response to nutrient levels | 8/217 | 237/18670 | 0.006654845 | 0.041890967 | 0.030214627 | FADS1/FAM107A/FOXO1/JUN/PIM1/PTGS2/SIK1/ZC3H12A | 8 | |
| BP | GO:0032872 | regulation of stress-activated MAPK cascade | 8/217 | 237/18670 | 0.006654845 | 0.041890967 | 0.030214627 | FOXO1/GADD45B/GADD45G/IL1B/IL1RN/MYC/WNT5A/ZC3H12A | 8 | |
| BP | GO:0048562 | embryonic organ morphogenesis | 9/217 | 288/18670 | 0.006703775 | 0.042128405 | 0.030385884 | ALDH1A3/EPHA2/LRIG3/NR4A3/SOX17/TBX3/TCAP/TGFB3/WNT5A | 9 | |
| BP | GO:0045088 | regulation of innate immune response | 12/217 | 452/18670 | 0.006791678 | 0.042304397 | 0.030512821 | BIRC3/GRAMD4/IL18RAP/IRAK3/LRRC19/MIR21/S100A8/SERPINB9/SOCS1/SOCS3/TRIM15/WNT5A | 12 | |
| BP | GO:0052547 | regulation of peptidase activity | 12/217 | 452/18670 | 0.006791678 | 0.042304397 | 0.030512821 | BIRC3/GRAMD4/KLF4/MIR21/MYC/PLAUR/PTGS2/S100A8/SERPINB9/SERPINE1/THBS1/TNFSF14 | 12 | |
| BP | GO:0000050 | urea cycle | 2/217 | 11/18670 | 0.006902349 | 0.042304397 | 0.030512821 | CEBPA/NAGS | 2 | |
| BP | GO:0000185 | activation of MAPKKK activity | 2/217 | 11/18670 | 0.006902349 | 0.042304397 | 0.030512821 | GADD45B/GADD45G | 2 | |
| BP | GO:0002604 | regulation of dendritic cell antigen processing and presentation | 2/217 | 11/18670 | 0.006902349 | 0.042304397 | 0.030512821 | SLC11A1/THBS1 | 2 | |
| BP | GO:0009629 | response to gravity | 2/217 | 11/18670 | 0.006902349 | 0.042304397 | 0.030512821 | FOS/FOSL1 | 2 | |
| BP | GO:0030241 | skeletal muscle myosin thick filament assembly | 2/217 | 11/18670 | 0.006902349 | 0.042304397 | 0.030512821 | MYH11/TCAP | 2 | |
| BP | GO:0031652 | positive regulation of heat generation | 2/217 | 11/18670 | 0.006902349 | 0.042304397 | 0.030512821 | IL1B/PTGS2 | 2 | |
| BP | GO:0035747 | natural killer cell chemotaxis | 2/217 | 11/18670 | 0.006902349 | 0.042304397 | 0.030512821 | CCL2/CCL3 | 2 | |
| BP | GO:0045628 | regulation of T-helper 2 cell differentiation | 2/217 | 11/18670 | 0.006902349 | 0.042304397 | 0.030512821 | BCL6/IL4R | 2 | |
| BP | GO:0046886 | positive regulation of hormone biosynthetic process | 2/217 | 11/18670 | 0.006902349 | 0.042304397 | 0.030512821 | EGR1/IL1B | 2 | |
| BP | GO:0051673 | membrane disruption in other organism | 2/217 | 11/18670 | 0.006902349 | 0.042304397 | 0.030512821 | DEFA1/DEFB118 | 2 | |
| BP | GO:0071688 | striated muscle myosin thick filament assembly | 2/217 | 11/18670 | 0.006902349 | 0.042304397 | 0.030512821 | MYH11/TCAP | 2 | |
| BP | GO:1903977 | positive regulation of glial cell migration | 2/217 | 11/18670 | 0.006902349 | 0.042304397 | 0.030512821 | CCL3/MIR221 | 2 | |
| BP | GO:1904995 | negative regulation of leukocyte adhesion to vascular endothelial cell | 2/217 | 11/18670 | 0.006902349 | 0.042304397 | 0.030512821 | KLF4/MIR221 | 2 | |
| BP | GO:0032729 | positive regulation of interferon-gamma production | 4/217 | 65/18670 | 0.006911895 | 0.042304397 | 0.030512821 | BCL3/IL1B/SLC11A1/WNT5A | 4 | |
| BP | GO:0070302 | regulation of stress-activated protein kinase signaling cascade | 8/217 | 239/18670 | 0.006987143 | 0.042695422 | 0.030794855 | FOXO1/GADD45B/GADD45G/IL1B/IL1RN/MYC/WNT5A/ZC3H12A | 8 | |
| BP | GO:0032652 | regulation of interleukin-1 production | 5/217 | 103/18670 | 0.007062695 | 0.043087028 | 0.031077308 | CCL3/EGR1/SAA1/WNT5A/ZC3H12A | 5 | |
| BP | GO:0001935 | endothelial cell proliferation | 7/217 | 191/18670 | 0.007088687 | 0.043175501 | 0.031141121 | CCL2/EPHA2/JUN/MIR21/NR4A1/THBS1/WNT5A | 7 | |
| BP | GO:0045066 | regulatory T cell differentiation | 3/217 | 34/18670 | 0.007106924 | 0.043216536 | 0.031170718 | BCL6/MIR21/SOCS1 | 3 | |
| BP | GO:1904951 | positive regulation of establishment of protein localization | 12/217 | 456/18670 | 0.007261797 | 0.044086971 | 0.031798535 | CCL3/CD274/IL1B/IL1RL1/IL4R/IL6/IRS2/PTGS2/SAA1/TGFB3/WNT5A/ZC3H12A | 12 | |
| BP | GO:0046888 | negative regulation of hormone secretion | 4/217 | 66/18670 | 0.007292151 | 0.044199843 | 0.031879946 | IL1B/LIF/PIM3/SIRT4 | 4 | |
| BP | GO:0030858 | positive regulation of epithelial cell differentiation | 4/217 | 67/18670 | 0.007685954 | 0.046269691 | 0.033372862 | FOXC1/LIF/MIR21/SERPINE1 | 4 | |
| BP | GO:0002701 | negative regulation of production of molecular mediator of immune response | 3/217 | 35/18670 | 0.007707511 | 0.046269691 | 0.033372862 | BCL6/IRAK3/TGFB3 | 3 | |
| BP | GO:0032689 | negative regulation of interferon-gamma production | 3/217 | 35/18670 | 0.007707511 | 0.046269691 | 0.033372862 | CD274/IL1RL1/ZC3H12A | 3 | |
| BP | GO:0048333 | mesodermal cell differentiation | 3/217 | 35/18670 | 0.007707511 | 0.046269691 | 0.033372862 | KLF4/SOX17/TRIM15 | 3 | |
| BP | GO:0060251 | regulation of glial cell proliferation | 3/217 | 35/18670 | 0.007707511 | 0.046269691 | 0.033372862 | IL1B/IL6/MIR221 | 3 | |
| BP | GO:1990774 | tumor necrosis factor secretion | 3/217 | 35/18670 | 0.007707511 | 0.046269691 | 0.033372862 | SPHK2/WNT5A/ZC3H12A | 3 | |
| BP | GO:0051592 | response to calcium ion | 6/217 | 148/18670 | 0.007739073 | 0.046385065 | 0.033456078 | FOS/FOSB/JUN/JUNB/THBS1/WNT5A | 6 | |
| BP | GO:0003300 | cardiac muscle hypertrophy | 5/217 | 106/18670 | 0.007951863 | 0.047496768 | 0.034257914 | FOXO1/MIR21/NR4A3/RGS2/TCAP | 5 | |
| BP | GO:0043547 | positive regulation of GTPase activity | 11/217 | 405/18670 | 0.007984954 | 0.047496768 | 0.034257914 | CCL2/CCL20/CCL3/EPHA1/EPHA2/JUN/RGS1/RGS16/RGS2/SIPA1L2/WNT5A | 11 | |
| BP | GO:0043900 | regulation of multi-organism process | 11/217 | 405/18670 | 0.007984954 | 0.047496768 | 0.034257914 | APOBEC3A/BIRC3/CCL3/IL1B/JUN/MIR221/PTX3/TMPRSS2/TRIM15/ZC3H12A/ZFP36 | 11 | |
| BP | GO:0002706 | regulation of lymphocyte mediated immunity | 6/217 | 149/18670 | 0.007987748 | 0.047496768 | 0.034257914 | BCL6/CLCF1/IL18RAP/IL1B/IL6/SERPINB9 | 6 | |
| BP | GO:1903900 | regulation of viral life cycle | 6/217 | 149/18670 | 0.007987748 | 0.047496768 | 0.034257914 | APOBEC3A/MIR221/PTX3/TMPRSS2/TRIM15/ZC3H12A | 6 | |
| BP | GO:0010812 | negative regulation of cell-substrate adhesion | 4/217 | 68/18670 | 0.008093496 | 0.047891239 | 0.034542433 | BCL6/FAM107A/SERPINE1/THBS1 | 4 | |
| BP | GO:0035914 | skeletal muscle cell differentiation | 4/217 | 68/18670 | 0.008093496 | 0.047891239 | 0.034542433 | EGR1/FOS/KLF5/MAFF | 4 | |
| BP | GO:1903522 | regulation of blood circulation | 9/217 | 297/18670 | 0.008124633 | 0.047891239 | 0.034542433 | AVPR1A/HBEGF/KCNE4/KCNK1/MIR21/PTGS2/RGS2/STC1/ZC3H12A | 9 | |
| BP | GO:2001233 | regulation of apoptotic signaling pathway | 11/217 | 406/18670 | 0.008125899 | 0.047891239 | 0.034542433 | BCL2A1/IL1B/MIR21/MIR221/NR4A2/PLAUR/PTGS2/S100A8/SERPINE1/THBS1/TNFRSF12A | 11 | |
| BP | GO:0002468 | dendritic cell antigen processing and presentation | 2/217 | 12/18670 | 0.008219758 | 0.047891239 | 0.034542433 | SLC11A1/THBS1 | 2 | |
| BP | GO:0002903 | negative regulation of B cell apoptotic process | 2/217 | 12/18670 | 0.008219758 | 0.047891239 | 0.034542433 | BCL6/IRS2 | 2 | |
| BP | GO:0006527 | arginine catabolic process | 2/217 | 12/18670 | 0.008219758 | 0.047891239 | 0.034542433 | MIR21/PADI4 | 2 | |
| BP | GO:0031034 | myosin filament assembly | 2/217 | 12/18670 | 0.008219758 | 0.047891239 | 0.034542433 | MYH11/TCAP | 2 | |
| BP | GO:0043922 | negative regulation by host of viral transcription | 2/217 | 12/18670 | 0.008219758 | 0.047891239 | 0.034542433 | CCL3/JUN | 2 | |
| BP | GO:0051549 | positive regulation of keratinocyte migration | 2/217 | 12/18670 | 0.008219758 | 0.047891239 | 0.034542433 | HAS2/HBEGF | 2 | |
| BP | GO:0060707 | trophoblast giant cell differentiation | 2/217 | 12/18670 | 0.008219758 | 0.047891239 | 0.034542433 | LIF/SOCS3 | 2 | |
| BP | GO:0060900 | embryonic camera-type eye formation | 2/217 | 12/18670 | 0.008219758 | 0.047891239 | 0.034542433 | ALDH1A3/WNT5A | 2 | |
| BP | GO:1900376 | regulation of secondary metabolite biosynthetic process | 2/217 | 12/18670 | 0.008219758 | 0.047891239 | 0.034542433 | MIR21/WNT5A | 2 | |
| BP | GO:2000516 | positive regulation of CD4-positive, alpha-beta T cell activation | 3/217 | 36/18670 | 0.008337183 | 0.048500205 | 0.034981661 | IL4R/MIR21/SOCS1 | 3 | |
| BP | GO:0070227 | lymphocyte apoptotic process | 4/217 | 69/18670 | 0.008514965 | 0.049457866 | 0.035672391 | BCL6/CD274/IRS2/WNT5A | 4 | |
| BP | GO:0031099 | regeneration | 7/217 | 198/18670 | 0.008559339 | 0.049638883 | 0.035802953 | C5AR1/IGFBP1/JUN/KLF4/KLF5/MIR221/NR4A3 | 7 | |
| MF | GO:0001228 | DNA-binding transcription activator activity, RNA polymerase II-specific | 23/214 | 439/17697 | 3.86E-09 | 1.63E-06 | 1.46E-06 | CEBPA/CEBPD/DBP/EGR1/ELF3/FOS/FOSB/FOSL1/FOSL2/FOXC1/JUN/JUNB/KLF4/KLF5/KLF6/MAFF/MYC/NR4A1/NR4A2/NR4A3/SOX17/TBX10/TBX3 | 23 | |
| MF | GO:0035014 | phosphatidylinositol 3-kinase regulator activity | 5/214 | 17/17697 | 1.36E-06 | 0.000286912 | 0.000255604 | CISH/KLF4/SOCS1/SOCS2/SOCS3 | 5 | |
| MF | GO:0050786 | RAGE receptor binding | 4/214 | 11/17697 | 6.42E-06 | 0.000905149 | 0.000806379 | FPR1/S100A12/S100A8/S100P | 4 | |
| MF | GO:0005125 | cytokine activity | 12/214 | 220/17697 | 1.57E-05 | 0.00160102 | 0.001426316 | CCL2/CCL20/CCL3/CLCF1/CMTM2/IL1B/IL1RN/IL6/LIF/TGFB3/TNFSF14/WNT5A | 12 | |
| MF | GO:0046935 | 1-phosphatidylinositol-3-kinase regulator activity | 4/214 | 14/17697 | 1.89E-05 | 0.00160102 | 0.001426316 | CISH/SOCS1/SOCS2/SOCS3 | 4 | |
| MF | GO:0035259 | glucocorticoid receptor binding | 4/214 | 16/17697 | 3.38E-05 | 0.00238011 | 0.002120392 | ETS2/NR4A1/NR4A2/NR4A3 | 4 | |
| MF | GO:0048018 | receptor ligand activity | 16/214 | 482/17697 | 0.00026551 | 0.016044415 | 0.014293643 | CCL2/CCL20/CCL3/CLCF1/CMTM2/FGF14/HBEGF/IL1B/IL1RN/IL6/LIF/SAA1/STC1/TGFB3/TNFSF14/WNT5A | 16 | |
| MF | GO:0001664 | G protein-coupled receptor binding | 11/214 | 280/17697 | 0.000626227 | 0.033111737 | 0.029498574 | AVPR1A/CCL2/CCL20/CCL3/FPR1/PROK2/RNF43/RTP3/RTP4/SAA1/WNT5A | 11 | |
| MF | GO:0005126 | cytokine receptor binding | 11/214 | 286/17697 | 0.000745771 | 0.035051249 | 0.031226446 | CCL2/CCL20/CCL3/CLCF1/IL1B/IL1RN/IL6/LIF/SOCS2/TGFB3/TNFSF14 | 11 | |
| KEGG pathway | hsa04668 | TNF signaling pathway | 13/120 | 112/8105 | 8.43E-09 | 1.75E-06 | 1.40E-06 | 602/330/6347/6364/2353/3553/3569/3725/3726/3976/1326/5743/9021 | 13 | |
| KEGG pathway | hsa04657 | IL-17 signaling pathway | 11/120 | 94/8105 | 1.16E-07 | 1.20E-05 | 9.63E-06 | 6347/6364/2353/2354/8061/3553/3569/3725/5596/5743/6279 | 11 | |
| KEGG pathway | hsa04933 | AGE-RAGE signaling pathway in diabetic complications | 10/120 | 100/8105 | 1.96E-06 | 0.000135087 | 0.000108537 | 6347/1958/2308/3553/3569/3725/5292/5054/7043/7056 | 10 | |
| KEGG pathway | hsa05202 | Transcriptional misregulation in cancer | 13/120 | 192/8105 | 4.69E-06 | 0.000242679 | 0.000194982 | 597/604/330/1050/1667/2308/4616/10912/2892/3569/4609/8013/7113 | 13 | |
| KEGG pathway | hsa04060 | Cytokine-cytokine receptor interaction | 15/120 | 295/8105 | 2.74E-05 | 0.001135211 | 0.000912094 | 6347/6364/6348/23529/8807/3553/9173/3557/3566/3569/3976/7043/8793/51330/8740 | 15 | |
| KEGG pathway | hsa05323 | Rheumatoid arthritis | 8/120 | 93/8105 | 6.51E-05 | 0.002246639 | 0.00180508 | 6347/6364/6348/2353/3553/3569/3725/7043 | 8 | |
| KEGG pathway | hsa04380 | Osteoclast differentiation | 9/120 | 128/8105 | 0.000110105 | 0.003247986 | 0.00260962 | 2353/2354/8061/2355/3553/3725/3726/8651/9021 | 9 | |
| KEGG pathway | hsa05142 | Chagas disease | 8/120 | 102/8105 | 0.000125526 | 0.003247986 | 0.00260962 | 6347/6348/2353/3553/3569/3725/5054/7043 | 8 | |
| KEGG pathway | hsa05210 | Colorectal cancer | 7/120 | 86/8105 | 0.000267372 | 0.006149563 | 0.004940915 | 2353/4616/10912/3725/4609/5900/7043 | 7 | |
| KEGG pathway | hsa05321 | Inflammatory bowel disease | 6/120 | 65/8105 | 0.000376393 | 0.007791336 | 0.00626001 | 8807/3553/3566/3569/3725/7043 | 6 | |
| KEGG pathway | hsa04630 | JAK-STAT signaling pathway | 9/120 | 162/8105 | 0.000640937 | 0.011588031 | 0.009310495 | 1154/3566/3569/3976/4609/5292/8651/8835/9021 | 9 | |
| KEGG pathway | hsa04061 | Viral protein interaction with cytokine and cytokine receptor | 7/120 | 100/8105 | 0.00067177 | 0.011588031 | 0.009310495 | 6347/6364/6348/8807/3569/8793/8740 | 7 | |
| KEGG pathway | hsa05144 | Malaria | 5/120 | 50/8105 | 0.000814326 | 0.012555895 | 0.010088134 | 6347/3553/3569/7043/7057 | 5 | |
| KEGG pathway | hsa04064 | NF-kappa B signaling pathway | 7/120 | 104/8105 | 0.000849191 | 0.012555895 | 0.010088134 | 597/330/4616/10912/3553/5743/8740 | 7 | |
| KEGG pathway | hsa04390 | Hippo signaling pathway | 8/120 | 157/8105 | 0.002226861 | 0.03073068 | 0.024690808 | 174/154796/330/122786/4609/5054/7043/7474 | 8 | |
| KEGG pathway | hsa04068 | FoxO signaling pathway | 7/120 | 131/8105 | 0.00321566 | 0.041602605 | 0.033425942 | 604/2308/4616/10912/3569/8660/7043 | 7 | |
| KEGG pathway | hsa04917 | Prolactin signaling pathway | 5/120 | 70/8105 | 0.003679007 | 0.044797318 | 0.03599276 | 1154/2353/8651/8835/9021 | 5 | |
| KEGG pathway | hsa04620 | Toll-like receptor signaling pathway | 6/120 | 104/8105 | 0.004335206 | 0.049854866 | 0.040056287 | 6348/2353/3553/3569/3725/1326 | 6 | |

|  |
| --- |

|  |
| --- |
|  |
