# Supplementary material for: Identification of polyunsaturated fatty acids related key modules and genes in metabolic dysfunction-associated fatty liver disease using WGCNA analysis
Source: Front Genet. 2022 Nov 8;13:951224. doi: 10.3389/fgene.2022.951224 (PMC9679514; doi:10.3389/fgene.2022.951224)
Supplement: Supplementary file 6 [file Table5.DOCX]

| **Class description** | **Ingredients** | **Grams** |
| --- | --- | --- |
| Protein | Casein, Lactic, 30 Mesh | 200.00 g |
| Protein | Cystine, L | 3.00 g |
| Carbohydrate | Lodex 10 | 125.00 g |
| Carbohydrate | Sucrose, Fine Granulated | 72.80 g |
| Fiber | Solka Floc, FCC200 | 50.00 g |
| Fat | Lard | 245.00 g |
| Fat | Soybean Oil, USP | 25.00 g |
| Mineral | [S10026B](https://researchdiets.com/en/formulas/S10026B)(Research Diets,Inc) | 50.00 g |
| Vitamin | Choline Bitartrate | 2.00 g |
| Vitamin | [V10001C](https://researchdiets.com/en/formulas/V10001C)(Research Diets,Inc) | 1.00 g |
| Dye | Dye, Blue FD&C #1, Alum. Lake 35-42% | 0.05 g |
|  | Total: | 773.85 g |

Table S5. High fat diet formulation
