# Supplementary material for: Identification of polyunsaturated fatty acids related key modules and genes in metabolic dysfunction-associated fatty liver disease using WGCNA analysis
Source: Front Genet. 2022 Nov 8;13:951224. doi: 10.3389/fgene.2022.951224 (PMC9679514; doi:10.3389/fgene.2022.951224)
Supplement: Supplementary file 7 [file Table3.DOCX]

| **Table S3. GO enrichment analysis for PUFA-related modules** | | | | | | | | | |  |  |
| --- | --- | --- | --- | --- | --- | --- | --- | --- | --- | --- | --- |
| **Module** | **Categary** | **ID** | **Description** | **GeneRatio** | **BgRatio** | **pvalue** | **p.adjust** | **qvalue** | **geneID** | | **Count** |
| *Brown* | BP | GO:0032496 | response to lipopolysaccharide | 33/336 | 330/18670 | 1.31E-15 | 5.56E-12 | 3.90E-12 | AKAP12/C5AR1/CCL2/CCL3/CXCL1/EDN1/FMO1/FOS/GJA1/ICAM1/IL10/IL1B/IL1RN/IL6/IRAK3/IRF8/JUN/JUNB/MIR21/PELI1/PTGER2/PTGS2/S100A8/SBNO2/SERPINE1/SIRPA/SLC11A1/TLR2/TNFAIP3/TNIP3/WNT5A/ZC3H12A/ZFP36 | | 33 |
|  | BP | GO:0002237 | response to molecule of bacterial origin | 33/336 | 343/18670 | 4.07E-15 | 8.61E-12 | 6.05E-12 | AKAP12/C5AR1/CCL2/CCL3/CXCL1/EDN1/FMO1/FOS/GJA1/ICAM1/IL10/IL1B/IL1RN/IL6/IRAK3/IRF8/JUN/JUNB/MIR21/PELI1/PTGER2/PTGS2/S100A8/SBNO2/SERPINE1/SIRPA/SLC11A1/TLR2/TNFAIP3/TNIP3/WNT5A/ZC3H12A/ZFP36 | | 33 |
|  | BP | GO:0060326 | cell chemotaxis | 30/336 | 304/18670 | 3.91E-14 | 5.51E-11 | 3.87E-11 | C5AR1/CALCA/CCL2/CCL3/CCL8/CCR1/CH25H/CXCL1/CXCR1/EDN1/EGR3/EPHA2/FPR2/HBEGF/IL10/IL1B/IL1RN/IL6/LPAR1/MIR223/NBL1/NR4A1/S100A12/S100A8/S100A9/SAA1/SAA2/SERPINE1/THBS1/WNT5A | | 30 |
|  | BP | GO:0097529 | myeloid leukocyte migration | 24/336 | 210/18670 | 6.34E-13 | 6.70E-10 | 4.71E-10 | C5AR1/CALCA/CCL2/CCL3/CCL8/CCR1/CD9/CXCL1/CXCR1/EDN1/FPR2/IL1B/IL1R1/IL1RN/IL6/MIR223/NBL1/S100A12/S100A8/S100A9/SAA1/SERPINE1/SIRPA/THBS1 | | 24 |
|  | BP | GO:0030595 | leukocyte chemotaxis | 24/336 | 224/18670 | 2.60E-12 | 2.20E-09 | 1.54E-09 | C5AR1/CALCA/CCL2/CCL3/CCL8/CCR1/CH25H/CXCL1/CXCR1/EDN1/FPR2/IL10/IL1B/IL1RN/IL6/MIR223/NBL1/S100A12/S100A8/S100A9/SAA1/SERPINE1/THBS1/WNT5A | | 24 |
|  | BP | GO:0032103 | positive regulation of response to external stimulus | 28/336 | 323/18670 | 6.80E-12 | 4.79E-09 | 3.37E-09 | C2CD4A/C2CD4B/C5AR1/CCL3/CCR1/CREB3L3/EDN1/F3/FPR2/IL1B/IL1RL1/IL6/LDLR/LPAR1/MIR21/NFKBIZ/OSM/PTGS2/S100A12/S100A8/S100A9/SERPINE1/STX3/THBS1/TLR2/TNFRSF1A/TRIM15/WNT5A | | 28 |
|  | BP | GO:0042116 | macrophage activation | 16/336 | 95/18670 | 1.29E-11 | 7.23E-09 | 5.08E-09 | C5AR1/CCL3/FPR2/IL10/IL1RL1/IL4R/IL6/JUN/LDLR/NAMPT/SBNO2/SLC11A1/THBS1/TLR2/WNT5A/ZC3H12A | | 16 |
|  | BP | GO:0050727 | regulation of inflammatory response | 34/336 | 485/18670 | 1.37E-11 | 7.23E-09 | 5.08E-09 | BCL6/BIRC3/C2CD4A/C2CD4B/C5AR1/CCL3/CREB3L3/FPR2/IL10/IL1B/IL1R1/IL1RL1/IL6/KLF4/LDLR/MIR21/MIR223/NFKBIZ/OSM/PTGS2/S100A12/S100A8/S100A9/SAA1/SBNO2/SERPINE1/SIRPA/SOCS3/TLR2/TNFAIP3/TNFAIP6/TNFRSF1A/WNT5A/ZC3H12A | | 34 |
|  | BP | GO:0050729 | positive regulation of inflammatory response | 19/336 | 153/18670 | 3.96E-11 | 1.86E-08 | 1.31E-08 | C2CD4A/C2CD4B/CCL3/CREB3L3/IL1B/IL1RL1/IL6/LDLR/MIR21/NFKBIZ/OSM/PTGS2/S100A12/S100A8/S100A9/SERPINE1/TLR2/TNFRSF1A/WNT5A | | 19 |
|  | BP | GO:0001819 | positive regulation of cytokine production | 32/336 | 464/18670 | 8.61E-11 | 3.64E-08 | 2.56E-08 | AKAP12/BCL3/BIRC3/C5AR1/CCL3/CD83/CLEC4E/EGR1/GATA4/IL10/IL1B/IL1R1/IL1RL1/IL1RN/IL4R/IL6/IRF1/IRF8/MIR21/MNDA/NR4A3/OSM/PANX1/PELI1/PTGS2/SAA1/SERPINE1/SLC11A1/THBS1/TLR2/TRIM15/WNT5A | | 32 |
|  | BP | GO:0050900 | leukocyte migration | 33/336 | 499/18670 | 1.29E-10 | 4.96E-08 | 3.48E-08 | B4GALT1/C5AR1/CALCA/CCL2/CCL3/CCL8/CCR1/CD9/CH25H/CXCL1/CXCR1/EDN1/FPR2/ICAM1/IL10/IL1B/IL1R1/IL1RN/IL6/MIR223/NBL1/S100A12/S100A8/S100A9/SAA1/SDC4/SELL/SERPINE1/SIRPA/SLC16A3/THBS1/TNFRSF10D/WNT5A | | 33 |
|  | BP | GO:0007159 | leukocyte cell-cell adhesion | 26/336 | 337/18670 | 5.07E-10 | 1.79E-07 | 1.26E-07 | BCL6/CCL2/CD83/DTX1/EGR3/HAS2/ICAM1/IGFBP2/IL10/IL1B/IL4R/IL6/IRF1/KLF4/MAP3K8/MIR21/NFKBIZ/NR4A3/PELI1/S100A8/S100A9/SDC4/SELL/SIRPA/SOCS1/ZC3H12A | | 26 |
|  | BP | GO:0031960 | response to corticosteroid | 18/336 | 162/18670 | 8.25E-10 | 2.68E-07 | 1.89E-07 | ANXA3/AVPR1A/EDN1/FAM107A/FOS/FOSB/FOSL1/FOXO1/ICAM1/IGFBP2/IL10/IL1RN/IL6/PARP1/PTGS2/STC1/TYMS/ZFP36 | | 18 |
|  | BP | GO:0022407 | regulation of cell-cell adhesion | 28/336 | 403/18670 | 1.16E-09 | 3.33E-07 | 2.34E-07 | BCL6/BMP2/CCL2/CD83/CD9/DTX1/EGR3/FSTL3/HAS2/ICAM1/IGFBP2/IL10/IL1B/IL1RN/IL4R/IL6/IRF1/KLF4/MAP3K8/MIR21/NFKBIZ/NR4A3/PELI1/SDC4/SIRPA/SOCS1/WNT5A/ZC3H12A | | 28 |
|  | BP | GO:0051384 | response to glucocorticoid | 17/336 | 146/18670 | 1.18E-09 | 3.33E-07 | 2.34E-07 | ANXA3/AVPR1A/EDN1/FAM107A/FOS/FOSB/FOSL1/FOXO1/ICAM1/IGFBP2/IL10/IL1RN/IL6/PTGS2/STC1/TYMS/ZFP36 | | 17 |
|  | BP | GO:0071496 | cellular response to external stimulus | 25/336 | 339/18670 | 2.80E-09 | 7.39E-07 | 5.19E-07 | ATF3/AVPR1A/FADS1/FAM107A/FOS/FOSL1/FOXO1/GJA1/ICAM1/IL1B/INHBB/IRF1/JUN/KLF10/MAP1LC3B/MAP3K14/NAMPT/NR4A2/P2RY11/PIM1/PTGS2/RNF152/SIK1/TNFRSF1A/ZC3H12A | | 25 |
|  | BP | GO:1990266 | neutrophil migration | 15/336 | 118/18670 | 3.34E-09 | 8.32E-07 | 5.84E-07 | C5AR1/CCL2/CCL3/CCL8/CXCL1/CXCR1/EDN1/IL1B/IL1R1/IL1RN/MIR223/S100A12/S100A8/S100A9/SAA1 | | 15 |
|  | BP | GO:0030593 | neutrophil chemotaxis | 14/336 | 104/18670 | 5.27E-09 | 1.19E-06 | 8.36E-07 | C5AR1/CCL2/CCL3/CCL8/CXCL1/CXCR1/EDN1/IL1B/IL1RN/MIR223/S100A12/S100A8/S100A9/SAA1 | | 14 |
|  | BP | GO:0097530 | granulocyte migration | 16/336 | 141/18670 | 5.35E-09 | 1.19E-06 | 8.36E-07 | C5AR1/CCL2/CCL3/CCL8/CXCL1/CXCR1/EDN1/IL1B/IL1R1/IL1RN/MIR223/S100A12/S100A8/S100A9/SAA1/THBS1 | | 16 |
|  | BP | GO:0071222 | cellular response to lipopolysaccharide | 19/336 | 205/18670 | 5.98E-09 | 1.20E-06 | 8.45E-07 | CCL2/CCL3/CXCL1/ICAM1/IL10/IL1B/IL1RN/IL6/IRF8/MIR21/SBNO2/SERPINE1/SIRPA/TLR2/TNFAIP3/TNIP3/WNT5A/ZC3H12A/ZFP36 | | 19 |
|  | BP | GO:0071621 | granulocyte chemotaxis | 15/336 | 123/18670 | 5.98E-09 | 1.20E-06 | 8.45E-07 | C5AR1/CCL2/CCL3/CCL8/CXCL1/CXCR1/EDN1/IL1B/IL1RN/MIR223/S100A12/S100A8/S100A9/SAA1/THBS1 | | 15 |
|  | BP | GO:0032602 | chemokine production | 13/336 | 89/18670 | 6.78E-09 | 1.30E-06 | 9.15E-07 | EGR1/EPHA2/IL10/IL1B/IL1RL1/IL4R/IL6/KLF4/S100A8/S100A9/SIRPA/TLR2/WNT5A | | 13 |
|  | BP | GO:1903037 | regulation of leukocyte cell-cell adhesion | 23/336 | 304/18670 | 7.57E-09 | 1.37E-06 | 9.65E-07 | BCL6/CCL2/CD83/DTX1/EGR3/HAS2/ICAM1/IGFBP2/IL10/IL1B/IL4R/IL6/IRF1/KLF4/MAP3K8/MIR21/NFKBIZ/NR4A3/PELI1/SDC4/SIRPA/SOCS1/ZC3H12A | | 23 |
|  | BP | GO:0071674 | mononuclear cell migration | 13/336 | 90/18670 | 7.80E-09 | 1.37E-06 | 9.65E-07 | C5AR1/CALCA/CCL2/CCL3/CCL8/CCR1/FPR2/IL6/NBL1/S100A12/SERPINE1/SIRPA/THBS1 | | 13 |
|  | BP | GO:0071219 | cellular response to molecule of bacterial origin | 19/336 | 212/18670 | 1.04E-08 | 1.76E-06 | 1.23E-06 | CCL2/CCL3/CXCL1/ICAM1/IL10/IL1B/IL1RN/IL6/IRF8/MIR21/SBNO2/SERPINE1/SIRPA/TLR2/TNFAIP3/TNIP3/WNT5A/ZC3H12A/ZFP36 | | 19 |
|  | BP | GO:0045444 | fat cell differentiation | 19/336 | 223/18670 | 2.37E-08 | 3.85E-06 | 2.71E-06 | ARID5B/BMP2/CEBPD/FOXO1/IL6/INHBB/KLF4/KLF5/LRG1/MIR21/NR4A1/NR4A2/NR4A3/PTGS2/RGS2/SOCS1/WNT5A/ZC3H12A/ZFP36 | | 19 |
|  | BP | GO:0042110 | T cell activation | 28/336 | 464/18670 | 2.54E-08 | 3.98E-06 | 2.80E-06 | BCL3/BCL6/CCL2/CD83/CLEC4D/CLEC4E/DTX1/EGR1/EGR3/GJA1/ICAM1/IGFBP2/IL10/IL1B/IL4R/IL6/IRF1/MAP3K8/MIR21/NFKBIZ/NHEJ1/PELI1/RELB/SDC4/SIRPA/SLC11A1/SOCS1/ZC3H12A | | 28 |
|  | BP | GO:0071347 | cellular response to interleukin-1 | 17/336 | 179/18670 | 2.66E-08 | 4.01E-06 | 2.82E-06 | AKAP12/CCL2/CCL3/CCL8/EDN1/EGR1/HAS2/ICAM1/IL1B/IL1R1/IL1RN/IL6/IRAK3/MAP3K8/PELI1/SIRPA/ZC3H12A | | 17 |
|  | BP | GO:0048545 | response to steroid hormone | 25/336 | 385/18670 | 3.55E-08 | 5.18E-06 | 3.64E-06 | ANXA3/AVPR1A/EDN1/FAM107A/FOS/FOSB/FOSL1/FOXO1/ICAM1/IGFBP2/IL10/IL1RN/IL6/NR4A1/NR4A2/NR4A3/PARP1/PTGER2/PTGS2/STC1/STRN3/THBS1/TLR2/TYMS/ZFP36 | | 25 |
|  | BP | GO:0032102 | negative regulation of response to external stimulus | 24/336 | 365/18670 | 5.32E-08 | 7.49E-06 | 5.26E-06 | CCL2/CD9/EDN1/FPR2/GJA1/IL10/KLF4/LDLR/MIR223/NBL1/PLAU/PLAUR/SAA1/SEMA3C/SEMA4B/SERPINE1/SIRPA/SOCS3/THBS1/TNFAIP3/TNFAIP6/TNFRSF1A/WNT5A/ZC3H12A | | 24 |
|  | BP | GO:0071216 | cellular response to biotic stimulus | 19/336 | 236/18670 | 5.88E-08 | 8.03E-06 | 5.64E-06 | CCL2/CCL3/CXCL1/ICAM1/IL10/IL1B/IL1RN/IL6/IRF8/MIR21/SBNO2/SERPINE1/SIRPA/TLR2/TNFAIP3/TNIP3/WNT5A/ZC3H12A/ZFP36 | | 19 |
|  | BP | GO:0070371 | ERK1 and ERK2 cascade | 22/336 | 317/18670 | 7.55E-08 | 9.98E-06 | 7.01E-06 | AKAP12/ATF3/BMP2/C5AR1/CCL2/CCL3/CCL8/CCR1/DUSP6/EPHA2/FPR2/GATA4/GLIPR2/ICAM1/IL1B/JUN/KLF4/MIR21/MYC/SIRPA/SPRY4/TNFAIP8L3 | | 22 |
|  | BP | GO:0051090 | regulation of DNA-binding transcription factor activity | 26/336 | 432/18670 | 8.60E-08 | 1.10E-05 | 7.74E-06 | ANXA3/ARID5B/EDN1/EGLN1/FOS/FOSL1/ICAM1/IL10/IL18RAP/IL1B/IL6/IRAK3/JUN/KLF4/PELI1/PIM1/PPRC1/S100A12/S100A8/S100A9/SIK1/TLR2/TNFAIP3/TRIM15/WNT5A/ZC3H12A | | 26 |
|  | BP | GO:0031668 | cellular response to extracellular stimulus | 20/336 | 268/18670 | 9.22E-08 | 1.15E-05 | 8.05E-06 | ATF3/AVPR1A/FADS1/FAM107A/FOS/FOSL1/FOXO1/ICAM1/INHBB/JUN/KLF10/MAP1LC3B/NAMPT/NR4A2/P2RY11/PIM1/PTGS2/RNF152/SIK1/ZC3H12A | | 20 |
|  | BP | GO:0007249 | I-kappaB kinase/NF-kappaB signaling | 20/336 | 269/18670 | 9.80E-08 | 1.18E-05 | 8.32E-06 | BCL3/BIRC3/EDAR/GJA1/IL1B/IL1RN/LPAR1/MAP3K14/PELI1/PIM2/RELB/S100A12/SIRPA/TLR2/TNFAIP3/TNFRSF1A/TNFSF10/TNIP3/WNT5A/ZC3H12A | | 20 |
|  | BP | GO:0044706 | multi-multicellular organism process | 18/336 | 222/18670 | 1.18E-07 | 1.39E-05 | 9.78E-06 | AVPR1A/B4GALT1/CALCA/DHODH/EDN1/FOS/FOSB/FOSL1/GJA1/IGFBP2/IL1B/JUNB/MAFF/MIR21/NAMPT/PTGS2/RGS2/STC1 | | 18 |
|  | BP | GO:0070372 | regulation of ERK1 and ERK2 cascade | 21/336 | 300/18670 | 1.30E-07 | 1.45E-05 | 1.02E-05 | AKAP12/ATF3/BMP2/C5AR1/CCL2/CCL3/CCL8/CCR1/DUSP6/EPHA2/FPR2/GATA4/GLIPR2/ICAM1/IL1B/JUN/KLF4/MIR21/SIRPA/SPRY4/TNFAIP8L3 | | 21 |
|  | BP | GO:0046677 | response to antibiotic | 22/336 | 327/18670 | 1.30E-07 | 1.45E-05 | 1.02E-05 | CRYAB/EDN1/EGR1/FOSL1/FOXO1/ICAM1/IL10/IL18RAP/IL6/JUN/KDM6B/KLF4/MIR21/NR4A3/OGG1/RGS2/S100A8/SIRPA/SLC1A3/TNFAIP3/TYMS/ZC3H12A | | 22 |
|  | BP | GO:1901653 | cellular response to peptide | 24/336 | 385/18670 | 1.44E-07 | 1.56E-05 | 1.10E-05 | CASP4/CISH/EDN1/FOXO1/FPR2/GCK/ICAM1/IGFBP1/IL1B/INHBB/IRS2/KLF10/KLF11/KLF4/KLF5/LPIN2/NAMPT/NR4A1/NR4A2/NR4A3/PARP1/SOCS1/SOCS2/SOCS3 | | 24 |
|  | BP | GO:0022409 | positive regulation of cell-cell adhesion | 19/336 | 255/18670 | 1.99E-07 | 2.10E-05 | 1.48E-05 | BCL6/CCL2/CD83/EGR3/FSTL3/HAS2/ICAM1/IGFBP2/IL10/IL1B/IL4R/IL6/MAP3K8/MIR21/NFKBIZ/NR4A3/SIRPA/SOCS1/WNT5A | | 19 |
|  | BP | GO:0070555 | response to interleukin-1 | 17/336 | 207/18670 | 2.22E-07 | 2.29E-05 | 1.61E-05 | AKAP12/CCL2/CCL3/CCL8/EDN1/EGR1/HAS2/ICAM1/IL1B/IL1R1/IL1RN/IL6/IRAK3/MAP3K8/PELI1/SIRPA/ZC3H12A | | 17 |
|  | BP | GO:0002548 | monocyte chemotaxis | 10/336 | 65/18670 | 2.32E-07 | 2.34E-05 | 1.64E-05 | CALCA/CCL2/CCL3/CCL8/CCR1/FPR2/IL6/NBL1/S100A12/SERPINE1 | | 10 |
|  | BP | GO:0032642 | regulation of chemokine production | 11/336 | 82/18670 | 2.44E-07 | 2.40E-05 | 1.69E-05 | EGR1/EPHA2/IL10/IL1B/IL1RL1/IL4R/IL6/KLF4/SIRPA/TLR2/WNT5A | | 11 |
|  | BP | GO:0048661 | positive regulation of smooth muscle cell proliferation | 12/336 | 101/18670 | 2.70E-07 | 2.57E-05 | 1.80E-05 | ADAMTS1/EDN1/ERN1/HBEGF/IL10/IL6/JUN/MIR21/NAMPT/NR4A3/PTGS2/THBS1 | | 12 |
|  | BP | GO:0009612 | response to mechanical stimulus | 17/336 | 210/18670 | 2.73E-07 | 2.57E-05 | 1.80E-05 | BTG2/EDN1/FOS/FOSB/FOSL1/GATA4/GJA1/IGFBP2/IL1B/IRF1/JUN/JUNB/MAP3K14/PTGS2/SLC1A3/THBS1/TNFRSF1A | | 17 |
|  | BP | GO:0033002 | muscle cell proliferation | 18/336 | 239/18670 | 3.57E-07 | 3.23E-05 | 2.27E-05 | ADAMTS1/EDN1/ERN1/FOXC1/GJA1/HBEGF/IL10/IL6/JUN/KLF4/MIR21/MIR223/NAMPT/NR4A3/PIM1/PTGS2/THBS1/TNFAIP3 | | 18 |
|  | BP | GO:0002292 | T cell differentiation involved in immune response | 10/336 | 68/18670 | 3.59E-07 | 3.23E-05 | 2.27E-05 | BCL3/BCL6/CLEC4D/CLEC4E/IL4R/IL6/MIR21/NFKBIZ/RELB/ZC3H12A | | 10 |
|  | BP | GO:0048660 | regulation of smooth muscle cell proliferation | 15/336 | 169/18670 | 4.27E-07 | 3.76E-05 | 2.64E-05 | ADAMTS1/EDN1/ERN1/HBEGF/IL10/IL6/JUN/KLF4/MIR21/MIR223/NAMPT/NR4A3/PTGS2/THBS1/TNFAIP3 | | 15 |
|  | BP | GO:0050920 | regulation of chemotaxis | 17/336 | 217/18670 | 4.35E-07 | 3.76E-05 | 2.64E-05 | C5AR1/CCL2/CCL3/CCR1/EDN1/F3/FPR2/IL6/LPAR1/MIR223/NBL1/SEMA3C/SEMA4B/SERPINE1/STX3/THBS1/WNT5A | | 17 |
|  | BP | GO:0002286 | T cell activation involved in immune response | 12/336 | 106/18670 | 4.59E-07 | 3.89E-05 | 2.73E-05 | BCL3/BCL6/CLEC4D/CLEC4E/ICAM1/IL4R/IL6/MIR21/NFKBIZ/RELB/SLC11A1/ZC3H12A | | 12 |
|  | BP | GO:0048659 | smooth muscle cell proliferation | 15/336 | 171/18670 | 4.97E-07 | 4.12E-05 | 2.90E-05 | ADAMTS1/EDN1/ERN1/HBEGF/IL10/IL6/JUN/KLF4/MIR21/MIR223/NAMPT/NR4A3/PTGS2/THBS1/TNFAIP3 | | 15 |
|  | BP | GO:0043030 | regulation of macrophage activation | 9/336 | 55/18670 | 5.46E-07 | 4.38E-05 | 3.08E-05 | CCL3/IL10/IL1RL1/IL4R/IL6/LDLR/THBS1/WNT5A/ZC3H12A | | 9 |
|  | BP | GO:0002685 | regulation of leukocyte migration | 16/336 | 196/18670 | 5.49E-07 | 4.38E-05 | 3.08E-05 | C5AR1/CCL2/CCL3/CCL8/CCR1/CD9/EDN1/FPR2/ICAM1/IL1R1/IL6/MIR223/NBL1/SERPINE1/THBS1/WNT5A | | 16 |
|  | BP | GO:0043367 | CD4-positive, alpha-beta T cell differentiation | 10/336 | 74/18670 | 8.07E-07 | 6.32E-05 | 4.44E-05 | BCL3/BCL6/CD83/IL4R/IL6/MIR21/NFKBIZ/RELB/SOCS1/ZC3H12A | | 10 |
|  | BP | GO:0042088 | T-helper 1 type immune response | 8/336 | 43/18670 | 8.48E-07 | 6.52E-05 | 4.58E-05 | BCL3/IL18RAP/IL1B/IL1R1/IL1RL1/IL4R/RELB/SLC11A1 | | 8 |
|  | BP | GO:0061900 | glial cell activation | 9/336 | 58/18670 | 8.72E-07 | 6.58E-05 | 4.62E-05 | C5AR1/CCL3/FPR2/IL1B/IL6/JUN/LDLR/NAMPT/TLR2 | | 9 |
|  | BP | GO:0043405 | regulation of MAP kinase activity | 21/336 | 337/18670 | 8.87E-07 | 6.58E-05 | 4.62E-05 | BMP2/C5AR1/DUSP16/DUSP6/EDN1/ERN1/FPR1/GADD45B/GADD45G/IL1B/IRAK3/LPAR1/MAP3K14/MAP3K8/PROK2/RGS2/S100A12/SAA1/SPRY4/THBS1/WNT5A | | 21 |
|  | BP | GO:0150076 | neuroinflammatory response | 10/336 | 75/18670 | 9.17E-07 | 6.68E-05 | 4.69E-05 | C5AR1/CCL3/FPR2/IL1B/IL6/JUN/LDLR/NAMPT/PTGS2/TLR2 | | 10 |
|  | BP | GO:0002688 | regulation of leukocyte chemotaxis | 12/336 | 114/18670 | 1.01E-06 | 7.15E-05 | 5.02E-05 | C5AR1/CCL2/CCL3/CCR1/EDN1/FPR2/IL6/MIR223/NBL1/SERPINE1/THBS1/WNT5A | | 12 |
|  | BP | GO:0042035 | regulation of cytokine biosynthetic process | 12/336 | 114/18670 | 1.01E-06 | 7.15E-05 | 5.02E-05 | AKAP12/BCL3/EGR1/IL10/IL1B/IL6/INHBB/IRF1/KLF4/THBS1/WNT5A/ZFP36 | | 12 |
|  | BP | GO:0002675 | positive regulation of acute inflammatory response | 7/336 | 31/18670 | 1.04E-06 | 7.22E-05 | 5.07E-05 | C2CD4A/C2CD4B/CREB3L3/IL1B/IL6/OSM/PTGS2 | | 7 |
|  | BP | GO:0002683 | negative regulation of immune system process | 25/336 | 463/18670 | 1.15E-06 | 7.85E-05 | 5.51E-05 | BCL6/CCL2/CCL3/DTX1/FSTL3/GRAMD4/IL10/IL1RL1/IL4R/IRAK3/IRF1/LDLR/MIR21/MIR223/MNDA/MYC/NBL1/PELI1/SDC4/SERPINB9/SOCS1/THBS1/TNFAIP3/ZC3H12A/ZFP36 | | 25 |
|  | BP | GO:0070486 | leukocyte aggregation | 5/336 | 12/18670 | 1.31E-06 | 8.47E-05 | 5.95E-05 | HAS2/IL1B/NR4A3/S100A8/S100A9 | | 5 |
|  | BP | GO:0042119 | neutrophil activation | 26/336 | 498/18670 | 1.31E-06 | 8.47E-05 | 5.95E-05 | ANXA3/B4GALT1/C5AR1/CLEC4D/CRISPLD2/CXCL1/CXCR1/FCAR/FPR1/FPR2/IL18RAP/LRG1/MNDA/PLAU/PLAUR/S100A12/S100A8/S100A9/S100P/SELL/SIRPA/SLC11A1/SLC2A3/TLR2/TMEM63A/TNFAIP6 | | 26 |
|  | BP | GO:0007162 | negative regulation of cell adhesion | 19/336 | 289/18670 | 1.34E-06 | 8.47E-05 | 5.95E-05 | BCL6/BMP2/CD9/DTX1/FAM107A/IL10/IL1RN/IL4R/IRF1/KLF4/MIR21/PELI1/RND1/SDC4/SERPINE1/SOCS1/TACSTD2/THBS1/ZC3H12A | | 19 |
|  | BP | GO:0043434 | response to peptide hormone | 24/336 | 436/18670 | 1.35E-06 | 8.47E-05 | 5.95E-05 | BTG2/CISH/EDN1/EGR1/FOXO1/GCK/GJA1/ICAM1/IGFBP1/IL10/IL1B/INHBB/IRS2/LPIN2/NR4A1/NR4A2/NR4A3/PARP1/PTGS2/SOCS1/SOCS2/SOCS3/TLR2/TNFSF10 | | 24 |
|  | BP | GO:0002446 | neutrophil mediated immunity | 26/336 | 499/18670 | 1.36E-06 | 8.47E-05 | 5.95E-05 | ANXA3/B4GALT1/C5AR1/CLEC4D/CRISPLD2/CXCL1/CXCR1/FCAR/FPR1/FPR2/IL6/LRG1/MNDA/PLAU/PLAUR/S100A12/S100A8/S100A9/S100P/SELL/SIRPA/SLC11A1/SLC2A3/TLR2/TMEM63A/TNFAIP6 | | 26 |
|  | BP | GO:0031667 | response to nutrient levels | 26/336 | 499/18670 | 1.36E-06 | 8.47E-05 | 5.95E-05 | ATF3/DHODH/FADS1/FAM107A/FOXO1/GATA4/ICAM1/IGFBP2/IL1B/INHBB/JUN/KLF10/LDLR/MAP1LC3B/NAMPT/OGG1/P2RY11/PANX1/PIM1/PTGS2/RNF152/SIK1/STC1/TYMS/ZC3H12A/ZFP36 | | 26 |
|  | BP | GO:0030728 | ovulation | 6/336 | 21/18670 | 1.40E-06 | 8.60E-05 | 6.04E-05 | ADAMTS1/IL4R/INHBB/PTGS2/RGS2/TNFAIP6 | | 6 |
|  | BP | GO:0043122 | regulation of I-kappaB kinase/NF-kappaB signaling | 17/336 | 237/18670 | 1.49E-06 | 8.99E-05 | 6.32E-05 | BIRC3/EDAR/GJA1/IL1B/IL1RN/LPAR1/MAP3K14/PELI1/PIM2/S100A12/SIRPA/TNFAIP3/TNFRSF1A/TNFSF10/TNIP3/WNT5A/ZC3H12A | | 17 |
|  | BP | GO:0002696 | positive regulation of leukocyte activation | 22/336 | 380/18670 | 1.66E-06 | 9.86E-05 | 6.93E-05 | BCL6/CCL2/CCL3/CD83/CLEC4D/EGR3/IGFBP2/IL10/IL1B/IL1RL1/IL4R/IL6/IRS2/MAP3K8/MIR21/NFKBIZ/NR4A3/PELI1/SIRPA/SOCS1/THBS1/WNT5A | | 22 |
|  | BP | GO:0030217 | T cell differentiation | 17/336 | 240/18670 | 1.77E-06 | 0.00010388 | 7.30E-05 | BCL3/BCL6/CD83/CLEC4D/CLEC4E/DTX1/EGR1/EGR3/IL4R/IL6/IRF1/MIR21/NFKBIZ/NHEJ1/RELB/SOCS1/ZC3H12A | | 17 |
|  | BP | GO:1903706 | regulation of hemopoiesis | 25/336 | 475/18670 | 1.83E-06 | 0.000105739 | 7.43E-05 | BCL6/CCL3/CCR1/CD83/DTX1/EGR3/FOS/FOXC1/FSTL3/IL4R/IRF1/JUN/KLF10/MIR21/MIR223/MYC/NFE2/NFKBIZ/NR4A3/OSM/PIM1/SOCS1/THBS1/ZC3H12A/ZFP36 | | 25 |
|  | BP | GO:0030098 | lymphocyte differentiation | 21/336 | 353/18670 | 1.86E-06 | 0.000106501 | 7.48E-05 | BCL3/BCL6/BLNK/CD83/CLEC4D/CLEC4E/DTX1/EGR1/EGR3/IL10/IL4R/IL6/IRF1/KLF6/MIR21/NFKBIZ/NHEJ1/ONECUT1/RELB/SOCS1/ZC3H12A | | 21 |
|  | BP | GO:0060759 | regulation of response to cytokine stimulus | 15/336 | 190/18670 | 1.89E-06 | 0.00010681 | 7.50E-05 | BIRC3/CASP4/EDN1/IL1R1/IL1RN/IL6/IRAK3/KLF4/MIR21/SOCS1/SOCS3/TLR2/TNFAIP3/TNFRSF1A/WNT5A | | 15 |
|  | BP | GO:0001774 | microglial cell activation | 8/336 | 48/18670 | 2.04E-06 | 0.000110891 | 7.79E-05 | C5AR1/CCL3/FPR2/IL6/JUN/LDLR/NAMPT/TLR2 | | 8 |
|  | BP | GO:0002269 | leukocyte activation involved in inflammatory response | 8/336 | 48/18670 | 2.04E-06 | 0.000110891 | 7.79E-05 | C5AR1/CCL3/FPR2/IL6/JUN/LDLR/NAMPT/TLR2 | | 8 |
|  | BP | GO:0046632 | alpha-beta T cell differentiation | 11/336 | 101/18670 | 2.05E-06 | 0.000110891 | 7.79E-05 | BCL3/BCL6/CD83/IL4R/IL6/IRF1/MIR21/NFKBIZ/RELB/SOCS1/ZC3H12A | | 11 |
|  | BP | GO:1903039 | positive regulation of leukocyte cell-cell adhesion | 16/336 | 218/18670 | 2.25E-06 | 0.000119266 | 8.38E-05 | BCL6/CCL2/CD83/EGR3/HAS2/ICAM1/IGFBP2/IL1B/IL4R/IL6/MAP3K8/MIR21/NFKBIZ/NR4A3/SIRPA/SOCS1 | | 16 |
|  | BP | GO:0002367 | cytokine production involved in immune response | 11/336 | 102/18670 | 2.26E-06 | 0.000119266 | 8.38E-05 | BCL6/IL10/IL18RAP/IL1B/IL1R1/IL6/IRAK3/NR4A3/SLC11A1/TLR2/WNT5A | | 11 |
|  | BP | GO:0042089 | cytokine biosynthetic process | 12/336 | 123/18670 | 2.28E-06 | 0.000119293 | 8.38E-05 | AKAP12/BCL3/EGR1/IL10/IL1B/IL6/INHBB/IRF1/KLF4/THBS1/WNT5A/ZFP36 | | 12 |
|  | BP | GO:1902105 | regulation of leukocyte differentiation | 18/336 | 272/18670 | 2.32E-06 | 0.000119845 | 8.42E-05 | BCL6/CCL3/CCR1/CD83/DTX1/EGR3/FOS/FSTL3/IL4R/IRF1/JUN/KLF10/MIR21/MIR223/MYC/NFKBIZ/SOCS1/ZC3H12A | | 18 |
|  | BP | GO:0006979 | response to oxidative stress | 24/336 | 451/18670 | 2.44E-06 | 0.000122773 | 8.62E-05 | CRYAB/EDN1/EGLN1/FOS/FOSL1/FOXO1/GATA4/IL10/IL18RAP/IL6/JUN/KDM6B/KLF4/MCL1/MIR21/NCOA7/NR4A2/NR4A3/OGG1/PARP1/PTGS2/SIRPA/TNFAIP3/ZC3H12A | | 24 |
|  | BP | GO:0042542 | response to hydrogen peroxide | 13/336 | 146/18670 | 2.44E-06 | 0.000122773 | 8.62E-05 | CRYAB/FOSL1/FOXO1/IL10/IL18RAP/IL6/JUN/KDM6B/KLF4/MIR21/NR4A3/SIRPA/TNFAIP3 | | 13 |
|  | BP | GO:0042107 | cytokine metabolic process | 12/336 | 124/18670 | 2.49E-06 | 0.000123856 | 8.70E-05 | AKAP12/BCL3/EGR1/IL10/IL1B/IL6/INHBB/IRF1/KLF4/THBS1/WNT5A/ZFP36 | | 12 |
|  | BP | GO:0002526 | acute inflammatory response | 16/336 | 220/18670 | 2.53E-06 | 0.000124627 | 8.75E-05 | B4GALT1/C2CD4A/C2CD4B/C5AR1/CREB3L3/F3/ICAM1/IL1B/IL6/OGG1/OSM/PLSCR1/PTGS2/S100A8/SAA1/SAA2 | | 16 |
|  | BP | GO:0043312 | neutrophil degranulation | 25/336 | 485/18670 | 2.65E-06 | 0.000128089 | 9.00E-05 | ANXA3/B4GALT1/C5AR1/CLEC4D/CRISPLD2/CXCL1/CXCR1/FCAR/FPR1/FPR2/LRG1/MNDA/PLAU/PLAUR/S100A12/S100A8/S100A9/S100P/SELL/SIRPA/SLC11A1/SLC2A3/TLR2/TMEM63A/TNFAIP6 | | 25 |
|  | BP | GO:1901342 | regulation of vasculature development | 23/336 | 422/18670 | 2.67E-06 | 0.000128089 | 9.00E-05 | ANXA3/C5AR1/EGLN1/EGR1/EPHA2/F3/FOXC1/GATA4/IL10/IL1B/IL6/KLF4/LRG1/MIR21/MIR223/PROK2/PTGS2/SERPINE1/THBS1/TNFAIP3/TNFRSF12A/WNT5A/ZC3H12A | | 23 |
|  | BP | GO:0032147 | activation of protein kinase activity | 20/336 | 333/18670 | 2.84E-06 | 0.000135158 | 9.49E-05 | ACVR2B/BMP2/C5AR1/CALCA/DUSP6/ERN1/FPR1/GADD45B/GADD45G/IL1B/LPAR1/MAP3K14/MAP3K8/MIR21/PROK2/SAA1/SLC11A1/SOCS1/THBS1/WNT5A | | 20 |
|  | BP | GO:0002283 | neutrophil activation involved in immune response | 25/336 | 488/18670 | 2.95E-06 | 0.00013867 | 9.74E-05 | ANXA3/B4GALT1/C5AR1/CLEC4D/CRISPLD2/CXCL1/CXCR1/FCAR/FPR1/FPR2/LRG1/MNDA/PLAU/PLAUR/S100A12/S100A8/S100A9/S100P/SELL/SIRPA/SLC11A1/SLC2A3/TLR2/TMEM63A/TNFAIP6 | | 25 |
|  | BP | GO:0050867 | positive regulation of cell activation | 22/336 | 394/18670 | 2.99E-06 | 0.000138808 | 9.75E-05 | BCL6/CCL2/CCL3/CD83/CLEC4D/EGR3/IGFBP2/IL10/IL1B/IL1RL1/IL4R/IL6/IRS2/MAP3K8/MIR21/NFKBIZ/NR4A3/PELI1/SIRPA/SOCS1/THBS1/WNT5A | | 22 |
|  | BP | GO:0002687 | positive regulation of leukocyte migration | 12/336 | 128/18670 | 3.48E-06 | 0.000159891 | 0.000112311 | C5AR1/CCL3/CCL8/CCR1/EDN1/FPR2/ICAM1/IL1R1/IL6/SERPINE1/THBS1/WNT5A | | 12 |
|  | BP | GO:0045785 | positive regulation of cell adhesion | 22/336 | 403/18670 | 4.30E-06 | 0.00019542 | 0.000137267 | BCL6/CCL2/CD83/EGR3/FSTL3/HAS2/ICAM1/IGFBP2/IL10/IL1B/IL4R/IL6/MAP3K8/MIR21/NFKBIZ/NR4A3/SAA1/SDC4/SIRPA/SOCS1/STX3/WNT5A | | 22 |
|  | BP | GO:0050863 | regulation of T cell activation | 19/336 | 314/18670 | 4.53E-06 | 0.000203993 | 0.000143288 | BCL6/CCL2/CD83/DTX1/EGR3/IGFBP2/IL10/IL1B/IL4R/IL6/IRF1/MAP3K8/MIR21/NFKBIZ/PELI1/SDC4/SIRPA/SOCS1/ZC3H12A | | 19 |
|  | BP | GO:1903034 | regulation of response to wounding | 14/336 | 179/18670 | 4.65E-06 | 0.000207207 | 0.000145546 | CD9/EDN1/F3/GJA1/HBEGF/IL10/KLF4/MIR21/PLAU/PLAUR/SERPINE1/THBS1/TNFAIP3/TNFRSF12A | | 14 |
|  | BP | GO:0043406 | positive regulation of MAP kinase activity | 17/336 | 258/18670 | 4.71E-06 | 0.000207291 | 0.000145605 | BMP2/C5AR1/DUSP6/EDN1/ERN1/FPR1/GADD45B/GADD45G/IL1B/LPAR1/MAP3K14/MAP3K8/PROK2/S100A12/SAA1/THBS1/WNT5A | | 17 |
|  | BP | GO:0000302 | response to reactive oxygen species | 16/336 | 232/18670 | 5.03E-06 | 0.000219152 | 0.000153936 | CRYAB/EDN1/EGLN1/FOS/FOSL1/FOXO1/IL10/IL18RAP/IL6/JUN/KDM6B/KLF4/MIR21/NR4A3/SIRPA/TNFAIP3 | | 16 |
|  | BP | GO:0050679 | positive regulation of epithelial cell proliferation | 15/336 | 206/18670 | 5.14E-06 | 0.000221978 | 0.000155921 | B4GALT1/BMP2/C5AR1/EGR3/F3/HAS2/IL10/JUN/LRG1/MIR21/MYC/NR4A1/NR4A3/TNFAIP3/WNT5A | | 15 |
|  | BP | GO:0022408 | negative regulation of cell-cell adhesion | 14/336 | 181/18670 | 5.30E-06 | 0.000226255 | 0.000158926 | BCL6/BMP2/CD9/DTX1/IL10/IL1RN/IL4R/IRF1/KLF4/MIR21/PELI1/SDC4/SOCS1/ZC3H12A | | 14 |
|  | BP | GO:0051091 | positive regulation of DNA-binding transcription factor activity | 17/336 | 261/18670 | 5.49E-06 | 0.00023217 | 0.00016308 | ANXA3/ARID5B/EDN1/FOSL1/ICAM1/IL10/IL18RAP/IL1B/IL6/IRAK3/PPRC1/S100A12/S100A8/S100A9/TLR2/TRIM15/WNT5A | | 17 |
|  | BP | GO:0046683 | response to organophosphorus | 12/336 | 134/18670 | 5.61E-06 | 0.000234902 | 0.000164999 | FOS/FOSB/FOSL1/IL1B/JUN/JUNB/LPAR1/P2RY11/PANX1/PTGS2/STC1/TYMS | | 12 |
|  | BP | GO:0050921 | positive regulation of chemotaxis | 12/336 | 135/18670 | 6.06E-06 | 0.000249872 | 0.000175514 | C5AR1/CCL3/CCR1/EDN1/F3/FPR2/IL6/LPAR1/SERPINE1/STX3/THBS1/WNT5A | | 12 |
|  | BP | GO:0035710 | CD4-positive, alpha-beta T cell activation | 10/336 | 92/18670 | 6.09E-06 | 0.000249872 | 0.000175514 | BCL3/BCL6/CD83/IL4R/IL6/MIR21/NFKBIZ/RELB/SOCS1/ZC3H12A | | 10 |
|  | BP | GO:0032609 | interferon-gamma production | 11/336 | 113/18670 | 6.18E-06 | 0.000251146 | 0.000176409 | BCL3/IL10/IL18RAP/IL1B/IL1R1/IL1RL1/IRF8/SIRPA/SLC11A1/WNT5A/ZC3H12A | | 11 |
|  | BP | GO:0050707 | regulation of cytokine secretion | 15/336 | 210/18670 | 6.50E-06 | 0.000261789 | 0.000183885 | AKAP12/CCL3/CLEC4E/IL10/IL1B/IL1RL1/IL4R/OSM/PANX1/SAA1/SOCS1/TLR2/TNFAIP3/WNT5A/ZC3H12A | | 15 |
|  | BP | GO:0031669 | cellular response to nutrient levels | 16/336 | 237/18670 | 6.59E-06 | 0.00026296 | 0.000184708 | ATF3/FADS1/FAM107A/FOXO1/ICAM1/INHBB/JUN/KLF10/MAP1LC3B/NAMPT/P2RY11/PIM1/PTGS2/RNF152/SIK1/ZC3H12A | | 16 |
|  | BP | GO:0045765 | regulation of angiogenesis | 21/336 | 383/18670 | 6.65E-06 | 0.00026296 | 0.000184708 | ANXA3/C5AR1/EGLN1/EPHA2/F3/FOXC1/GATA4/IL10/IL1B/IL6/KLF4/LRG1/MIR21/PROK2/PTGS2/SERPINE1/THBS1/TNFAIP3/TNFRSF12A/WNT5A/ZC3H12A | | 21 |
|  | BP | GO:0002819 | regulation of adaptive immune response | 13/336 | 160/18670 | 6.73E-06 | 0.000263353 | 0.000184984 | BCL6/IL10/IL1B/IL1R1/IL1RL1/IL4R/IL6/IRF1/MIR21/NFKBIZ/SLC11A1/TNFAIP3/ZC3H12A | | 13 |
|  | BP | GO:0043032 | positive regulation of macrophage activation | 6/336 | 27/18670 | 6.99E-06 | 0.00027118 | 0.000190482 | CCL3/IL10/IL1RL1/IL4R/THBS1/WNT5A | | 6 |
|  | BP | GO:0032612 | interleukin-1 production | 11/336 | 115/18670 | 7.32E-06 | 0.00028147 | 0.00019771 | CCL3/EGR1/IL10/IL1B/MNDA/PANX1/SAA1/SIRPA/TNFAIP3/WNT5A/ZC3H12A | | 11 |
|  | BP | GO:0001818 | negative regulation of cytokine production | 18/336 | 296/18670 | 7.56E-06 | 0.000287993 | 0.000202292 | BCL3/BCL6/CD83/EPHA2/IL10/IL1RL1/IL6/INHBB/IRAK3/KLF4/MIR21/RELB/SIRPA/SLC11A1/THBS1/TNFAIP3/ZC3H12A/ZFP36 | | 18 |
|  | BP | GO:0050663 | cytokine secretion | 16/336 | 240/18670 | 7.73E-06 | 0.000292012 | 0.000205115 | AKAP12/CCL3/CLEC4E/IL10/IL1B/IL1RL1/IL4R/OSM/PANX1/S100A12/SAA1/SOCS1/TLR2/TNFAIP3/WNT5A/ZC3H12A | | 16 |
|  | BP | GO:0050715 | positive regulation of cytokine secretion | 12/336 | 139/18670 | 8.19E-06 | 0.00030647 | 0.00021527 | AKAP12/CCL3/CLEC4E/IL10/IL1B/IL1RL1/IL4R/OSM/PANX1/SAA1/TLR2/WNT5A | | 12 |
|  | BP | GO:0045088 | regulation of innate immune response | 23/336 | 452/18670 | 8.27E-06 | 0.000306619 | 0.000215375 | BIRC3/CLEC4D/CLEC4E/FPR2/GRAMD4/IL18RAP/IRAK3/IRF1/MIR21/MNDA/PELI1/PLSCR1/RELB/S100A8/S100A9/SERPINB9/SOCS1/SOCS3/TLR2/TNFAIP3/TNIP3/TRIM15/WNT5A | | 23 |
|  | BP | GO:0043281 | regulation of cysteine-type endopeptidase activity involved in apoptotic process | 15/336 | 215/18670 | 8.64E-06 | 0.000317617 | 0.0002231 | BIRC3/CASP4/CRYAB/F3/GRAMD4/KLF4/MYC/PLAUR/PTGS2/S100A8/S100A9/SERPINB9/SOX7/THBS1/TNFSF10 | | 15 |
|  | BP | GO:0042093 | T-helper cell differentiation | 8/336 | 58/18670 | 8.87E-06 | 0.000323238 | 0.000227048 | BCL3/BCL6/IL4R/IL6/MIR21/NFKBIZ/RELB/ZC3H12A | | 8 |
|  | BP | GO:0042742 | defense response to bacterium | 19/336 | 330/18670 | 9.24E-06 | 0.000333857 | 0.000234508 | ADAMTS5/ANXA3/BCL3/C5AR1/CLEC4D/CLEC4E/EPHA2/FPR2/IL10/IL6/IRF8/MIR223/S100A12/S100A8/S100A9/SERPINE1/SLC11A1/TLR2/TNFRSF1A | | 19 |
|  | BP | GO:0032868 | response to insulin | 17/336 | 272/18670 | 9.47E-06 | 0.000339413 | 0.00023841 | CISH/EGR1/FOXO1/GCK/ICAM1/IGFBP1/IL10/IL1B/INHBB/IRS2/LPIN2/PARP1/SOCS1/SOCS2/SOCS3/TLR2/TNFSF10 | | 17 |
|  | BP | GO:0001935 | endothelial cell proliferation | 14/336 | 191/18670 | 9.84E-06 | 0.000349621 | 0.00024558 | BMP2/CCL2/EGR3/EPHA2/ERN1/F3/GJA1/IL10/JUN/LRG1/MIR21/NR4A1/THBS1/WNT5A | | 14 |
|  | BP | GO:0034599 | cellular response to oxidative stress | 18/336 | 302/18670 | 9.95E-06 | 0.000350514 | 0.000246207 | FOS/FOXO1/GATA4/IL10/IL18RAP/IL6/JUN/KDM6B/KLF4/MCL1/MIR21/NCOA7/NR4A2/NR4A3/PARP1/SIRPA/TNFAIP3/ZC3H12A | | 18 |
|  | BP | GO:0007565 | female pregnancy | 14/336 | 192/18670 | 1.04E-05 | 0.000364951 | 0.000256348 | CALCA/DHODH/FOS/FOSB/FOSL1/GJA1/IGFBP2/IL1B/JUNB/MIR21/NAMPT/PTGS2/RGS2/STC1 | | 14 |
|  | BP | GO:0097237 | cellular response to toxic substance | 16/336 | 247/18670 | 1.11E-05 | 0.000384777 | 0.000270274 | ALOX5AP/EGR1/FOXO1/IL10/IL18RAP/IL6/KDM6B/KLF4/MIR21/MSX1/NR4A3/PTGS2/S100A9/SIRPA/TNFAIP3/ZC3H12A | | 16 |
|  | BP | GO:0001667 | ameboidal-type cell migration | 23/336 | 461/18670 | 1.14E-05 | 0.0003906 | 0.000274364 | AKAP12/ANXA3/ARID5B/EDN1/EGR3/EPHA2/GLIPR2/HAS2/HBEGF/JUN/KLF4/MIR21/NR4A1/PTGS2/S100P/SDC4/SEMA3C/SEMA4B/STC1/TACSTD2/THBS1/WNT5A/ZC3H12A | | 23 |
|  | BP | GO:0002294 | CD4-positive, alpha-beta T cell differentiation involved in immune response | 8/336 | 60/18670 | 1.15E-05 | 0.000391225 | 0.000274803 | BCL3/BCL6/IL4R/IL6/MIR21/NFKBIZ/RELB/ZC3H12A | | 8 |
|  | BP | GO:0070301 | cellular response to hydrogen peroxide | 10/336 | 99/18670 | 1.18E-05 | 0.000398075 | 0.000279615 | FOXO1/IL10/IL18RAP/IL6/KDM6B/KLF4/MIR21/NR4A3/SIRPA/TNFAIP3 | | 10 |
|  | BP | GO:2000377 | regulation of reactive oxygen species metabolic process | 14/336 | 195/18670 | 1.25E-05 | 0.000418047 | 0.000293644 | CRYAB/EDN1/FOXO1/FPR2/ICAM1/IL10/IL1B/KLF4/MIR21/PTGS2/SIRPA/STK17A/THBS1/ZC3H12A | | 14 |
|  | BP | GO:0002822 | regulation of adaptive immune response based on somatic recombination of immune receptors built from immunoglobulin superfamily domains | 12/336 | 145/18670 | 1.26E-05 | 0.000419917 | 0.000294957 | BCL6/IL10/IL1B/IL1R1/IL1RL1/IL4R/IL6/MIR21/NFKBIZ/SLC11A1/TNFAIP3/ZC3H12A | | 12 |
|  | BP | GO:0002287 | alpha-beta T cell activation involved in immune response | 8/336 | 61/18670 | 1.30E-05 | 0.000426121 | 0.000299315 | BCL3/BCL6/IL4R/IL6/MIR21/NFKBIZ/RELB/ZC3H12A | | 8 |
|  | BP | GO:0002293 | alpha-beta T cell differentiation involved in immune response | 8/336 | 61/18670 | 1.30E-05 | 0.000426121 | 0.000299315 | BCL3/BCL6/IL4R/IL6/MIR21/NFKBIZ/RELB/ZC3H12A | | 8 |
|  | BP | GO:0051250 | negative regulation of lymphocyte activation | 12/336 | 146/18670 | 1.35E-05 | 0.000439859 | 0.000308965 | BCL6/DTX1/IL10/IL4R/IRF1/MIR21/MNDA/PELI1/SDC4/SOCS1/TNFAIP3/ZC3H12A | | 12 |
|  | BP | GO:0048732 | gland development | 22/336 | 434/18670 | 1.38E-05 | 0.000441102 | 0.000309838 | ALDH1A3/ARID5B/BMP2/CIT/DHODH/EDAR/ELF3/EPHA2/FOXC1/FSTL3/GJA1/IL10/IRS2/JUN/MSX1/ONECUT1/SALL1/SEMA3C/SOCS2/TNFAIP3/TYMS/WNT5A | | 22 |
|  | BP | GO:0050673 | epithelial cell proliferation | 22/336 | 434/18670 | 1.38E-05 | 0.000441102 | 0.000309838 | B4GALT1/BMP2/C5AR1/CCL2/EGR3/EPHA2/ERN1/F3/GJA1/HAS2/IL10/JUN/LRG1/MIR21/MYC/NR4A1/NR4A3/TACSTD2/THBS1/TNFAIP3/WNT5A/ZFP36 | | 22 |
|  | BP | GO:0002791 | regulation of peptide secretion | 24/336 | 500/18670 | 1.39E-05 | 0.000441102 | 0.000309838 | AKAP12/CCL3/CLEC4E/GCK/GJA1/IL10/IL1B/IL1RL1/IL4R/IL6/INHBB/IRS2/MIDN/OSM/PANX1/PIM3/S100A8/SAA1/SIRT4/SOCS1/TLR2/TNFAIP3/WNT5A/ZC3H12A | | 24 |
|  | BP | GO:0032649 | regulation of interferon-gamma production | 10/336 | 101/18670 | 1.41E-05 | 0.000443638 | 0.000311619 | BCL3/IL10/IL1B/IL1R1/IL1RL1/IRF8/SIRPA/SLC11A1/WNT5A/ZC3H12A | | 10 |
|  | BP | GO:0032570 | response to progesterone | 7/336 | 45/18670 | 1.45E-05 | 0.000447262 | 0.000314165 | FOS/FOSB/FOSL1/PTGER2/THBS1/TLR2/TYMS | | 7 |
|  | BP | GO:0045581 | negative regulation of T cell differentiation | 7/336 | 45/18670 | 1.45E-05 | 0.000447262 | 0.000314165 | BCL6/DTX1/IL4R/IRF1/MIR21/SOCS1/ZC3H12A | | 7 |
|  | BP | GO:0071236 | cellular response to antibiotic | 12/336 | 147/18670 | 1.45E-05 | 0.000447262 | 0.000314165 | EGR1/FOXO1/IL10/IL18RAP/IL6/KDM6B/KLF4/MIR21/NR4A3/SIRPA/TNFAIP3/ZC3H12A | | 12 |
|  | BP | GO:0034612 | response to tumor necrosis factor | 18/336 | 312/18670 | 1.55E-05 | 0.000473352 | 0.000332491 | AKAP12/BIRC3/CASP4/CCL2/CCL3/CCL8/EDAR/EDN1/HAS2/ICAM1/MAP3K14/PTGS2/THBS1/TNFAIP3/TNFRSF12A/TNFRSF1A/ZC3H12A/ZFP36 | | 18 |
|  | BP | GO:0034341 | response to interferon-gamma | 14/336 | 199/18670 | 1.57E-05 | 0.000473352 | 0.000332491 | CCL2/CCL3/CCL8/EDN1/ICAM1/IRF1/IRF8/RAB20/SIRPA/SLC11A1/SOCS1/SOCS3/TLR2/WNT5A | | 14 |
|  | BP | GO:0050866 | negative regulation of cell activation | 14/336 | 199/18670 | 1.57E-05 | 0.000473352 | 0.000332491 | BCL6/CD9/DTX1/IL10/IL4R/IRF1/LDLR/MIR21/MNDA/PELI1/SDC4/SOCS1/TNFAIP3/ZC3H12A | | 14 |
|  | BP | GO:0001101 | response to acid chemical | 19/336 | 343/18670 | 1.59E-05 | 0.000476871 | 0.000334963 | AVPR1A/CD9/DHODH/EDN1/EGR1/FOXO1/GJA1/ICAM1/IGFBP2/KLF4/LDLR/OGG1/PTGER2/PTGS2/SOCS1/TLR2/TYMS/WNT5A/ZC3H12A | | 19 |
|  | BP | GO:2001233 | regulation of apoptotic signaling pathway | 21/336 | 406/18670 | 1.61E-05 | 0.000478802 | 0.000336319 | ATF3/BCL2A1/GATA4/ICAM1/IL1B/INHBB/ITPRIP/MCL1/MIR21/MSX1/NR4A2/PARP1/PLAUR/PTGS2/S100A8/S100A9/SERPINE1/THBS1/TNFAIP3/TNFRSF12A/TNFSF10 | | 21 |
|  | BP | GO:0046209 | nitric oxide metabolic process | 9/336 | 82/18670 | 1.64E-05 | 0.000484364 | 0.000340226 | EDN1/ICAM1/IL10/IL1B/KLF4/PTGS2/SIRPA/TLR2/ZC3H12A | | 9 |
|  | BP | GO:0050708 | regulation of protein secretion | 23/336 | 472/18670 | 1.66E-05 | 0.000484364 | 0.000340226 | AKAP12/CCL3/CLEC4E/GCK/GJA1/IL10/IL1B/IL1RL1/IL4R/IL6/INHBB/IRS2/MIDN/OSM/PANX1/PIM3/SAA1/SIRT4/SOCS1/TLR2/TNFAIP3/WNT5A/ZC3H12A | | 23 |
|  | BP | GO:0032652 | regulation of interleukin-1 production | 10/336 | 103/18670 | 1.67E-05 | 0.000484364 | 0.000340226 | CCL3/EGR1/IL10/MNDA/PANX1/SAA1/SIRPA/TNFAIP3/WNT5A/ZC3H12A | | 10 |
|  | BP | GO:1902106 | negative regulation of leukocyte differentiation | 10/336 | 103/18670 | 1.67E-05 | 0.000484364 | 0.000340226 | BCL6/CCL3/DTX1/FSTL3/IL4R/IRF1/MIR21/MYC/SOCS1/ZC3H12A | | 10 |
|  | BP | GO:0002544 | chronic inflammatory response | 5/336 | 19/18670 | 1.73E-05 | 0.000498146 | 0.000349906 | GJA1/IL10/S100A8/THBS1/TNFAIP3 | | 5 |
|  | BP | GO:0002695 | negative regulation of leukocyte activation | 13/336 | 175/18670 | 1.77E-05 | 0.000505794 | 0.000355279 | BCL6/DTX1/IL10/IL4R/IRF1/LDLR/MIR21/MNDA/PELI1/SDC4/SOCS1/TNFAIP3/ZC3H12A | | 13 |
|  | BP | GO:0050678 | regulation of epithelial cell proliferation | 20/336 | 378/18670 | 1.85E-05 | 0.000524503 | 0.000368421 | B4GALT1/BMP2/C5AR1/CCL2/EGR3/F3/GJA1/HAS2/IL10/JUN/LRG1/MIR21/MYC/NR4A1/NR4A3/TACSTD2/THBS1/TNFAIP3/WNT5A/ZFP36 | | 20 |
|  | BP | GO:0019216 | regulation of lipid metabolic process | 21/336 | 410/18670 | 1.86E-05 | 0.000524503 | 0.000368421 | AVPR1A/BMP2/CISH/EGR1/FADS1/FPR2/GRHL1/IL1B/IRS2/KLF4/LDLR/ME1/NR4A3/PTGS2/SIK1/SIRT4/SOCS1/SOCS2/SOCS3/TNFAIP8L3/TNFRSF1A | | 21 |
|  | BP | GO:1904018 | positive regulation of vasculature development | 15/336 | 230/18670 | 1.93E-05 | 0.000537951 | 0.000377867 | ANXA3/C5AR1/EGR1/F3/GATA4/IL10/IL1B/KLF4/LRG1/MIR21/PTGS2/SERPINE1/THBS1/WNT5A/ZC3H12A | | 15 |
|  | BP | GO:0043370 | regulation of CD4-positive, alpha-beta T cell differentiation | 7/336 | 47/18670 | 1.95E-05 | 0.000537951 | 0.000377867 | BCL6/CD83/IL4R/MIR21/NFKBIZ/SOCS1/ZC3H12A | | 7 |
|  | BP | GO:0061614 | pri-miRNA transcription by RNA polymerase II | 7/336 | 47/18670 | 1.95E-05 | 0.000537951 | 0.000377867 | BMP2/FOS/FOSL1/IL10/JUN/KLF4/KLF5 | | 7 |
|  | BP | GO:0051385 | response to mineralocorticoid | 6/336 | 32/18670 | 1.98E-05 | 0.000541508 | 0.000380365 | AVPR1A/EDN1/FOS/FOSB/FOSL1/PARP1 | | 6 |
|  | BP | GO:0002793 | positive regulation of peptide secretion | 17/336 | 288/18670 | 1.99E-05 | 0.000541508 | 0.000380365 | AKAP12/CCL3/CLEC4E/GCK/GJA1/IL10/IL1B/IL1RL1/IL4R/IL6/IRS2/OSM/PANX1/S100A8/SAA1/TLR2/WNT5A | | 17 |
|  | BP | GO:0001959 | regulation of cytokine-mediated signaling pathway | 13/336 | 177/18670 | 2.00E-05 | 0.000541508 | 0.000380365 | BIRC3/CASP4/EDN1/IL1R1/IL1RN/IL6/IRAK3/MIR21/SOCS1/SOCS3/TNFAIP3/TNFRSF1A/WNT5A | | 13 |
|  | BP | GO:0007178 | transmembrane receptor protein serine/threonine kinase signaling pathway | 19/336 | 349/18670 | 2.02E-05 | 0.000544894 | 0.000382744 | ACVR2B/BMP2/EGR1/FOS/FSTL3/GATA4/HIVEP1/INHBB/INHBE/JUN/LRG1/MIR21/MSX1/NBL1/ONECUT1/PARP1/PEG10/THBS1/WNT5A | | 19 |
|  | BP | GO:0002573 | myeloid leukocyte differentiation | 14/336 | 204/18670 | 2.07E-05 | 0.000550661 | 0.000386794 | CCL3/CCR1/EPHA2/FOS/FSTL3/JUN/JUNB/KLF10/MIR223/MYC/PARP1/RELB/SBNO2/TLR2 | | 14 |
|  | BP | GO:0045766 | positive regulation of angiogenesis | 14/336 | 204/18670 | 2.07E-05 | 0.000550661 | 0.000386794 | ANXA3/C5AR1/F3/GATA4/IL10/IL1B/KLF4/LRG1/MIR21/PTGS2/SERPINE1/THBS1/WNT5A/ZC3H12A | | 14 |
|  | BP | GO:0042133 | neurotransmitter metabolic process | 12/336 | 153/18670 | 2.17E-05 | 0.000572769 | 0.000402323 | EDN1/ICAM1/IL10/IL1B/KLF4/PTGS2/RNF180/SIRPA/SLC1A3/SLC25A32/TLR2/ZC3H12A | | 12 |
|  | BP | GO:1903038 | negative regulation of leukocyte cell-cell adhesion | 11/336 | 129/18670 | 2.19E-05 | 0.000575113 | 0.00040397 | BCL6/DTX1/IL10/IL4R/IRF1/KLF4/MIR21/PELI1/SDC4/SOCS1/ZC3H12A | | 11 |
|  | BP | GO:2001057 | reactive nitrogen species metabolic process | 9/336 | 85/18670 | 2.20E-05 | 0.000575144 | 0.000403992 | EDN1/ICAM1/IL10/IL1B/KLF4/PTGS2/SIRPA/TLR2/ZC3H12A | | 9 |
|  | BP | GO:0071356 | cellular response to tumor necrosis factor | 17/336 | 291/18670 | 2.27E-05 | 0.00058304 | 0.000409538 | AKAP12/BIRC3/CASP4/CCL2/CCL3/CCL8/EDAR/EDN1/HAS2/ICAM1/MAP3K14/THBS1/TNFAIP3/TNFRSF12A/TNFRSF1A/ZC3H12A/ZFP36 | | 17 |
|  | BP | GO:0051767 | nitric-oxide synthase biosynthetic process | 5/336 | 20/18670 | 2.27E-05 | 0.00058304 | 0.000409538 | AKAP12/CCL2/EDN1/NAMPT/TLR2 | | 5 |
|  | BP | GO:0051769 | regulation of nitric-oxide synthase biosynthetic process | 5/336 | 20/18670 | 2.27E-05 | 0.00058304 | 0.000409538 | AKAP12/CCL2/EDN1/NAMPT/TLR2 | | 5 |
|  | BP | GO:0045428 | regulation of nitric oxide biosynthetic process | 8/336 | 66/18670 | 2.34E-05 | 0.00059739 | 0.000419618 | EDN1/ICAM1/IL10/IL1B/KLF4/PTGS2/SIRPA/ZC3H12A | | 8 |
|  | BP | GO:0071346 | cellular response to interferon-gamma | 13/336 | 180/18670 | 2.39E-05 | 0.000604383 | 0.00042453 | CCL2/CCL3/CCL8/EDN1/ICAM1/IRF1/IRF8/RAB20/SIRPA/SOCS1/SOCS3/TLR2/WNT5A | | 13 |
|  | BP | GO:0002285 | lymphocyte activation involved in immune response | 13/336 | 181/18670 | 2.53E-05 | 0.000636958 | 0.000447411 | BCL3/BCL6/CLEC4D/CLEC4E/ICAM1/IL10/IL4R/IL6/MIR21/NFKBIZ/RELB/SLC11A1/ZC3H12A | | 13 |
|  | BP | GO:1904707 | positive regulation of vascular smooth muscle cell proliferation | 7/336 | 49/18670 | 2.58E-05 | 0.000645013 | 0.000453069 | ADAMTS1/EDN1/ERN1/IL10/JUN/MIR21/NR4A3 | | 7 |
|  | BP | GO:0042108 | positive regulation of cytokine biosynthetic process | 8/336 | 67/18670 | 2.62E-05 | 0.000652181 | 0.000458104 | AKAP12/BCL3/EGR1/IL1B/IL6/IRF1/THBS1/WNT5A | | 8 |
|  | BP | GO:0002690 | positive regulation of leukocyte chemotaxis | 9/336 | 87/18670 | 2.66E-05 | 0.000657748 | 0.000462014 | C5AR1/CCL3/CCR1/EDN1/FPR2/IL6/SERPINE1/THBS1/WNT5A | | 9 |
|  | BP | GO:0009615 | response to virus | 18/336 | 326/18670 | 2.77E-05 | 0.000681868 | 0.000478956 | APOBEC3A/BCL3/BIRC3/CCL8/FOSL1/IFIT2/IFIT3/IL1B/IL6/IRAK3/IRF1/ISG20/MAP3K14/PIM2/PLSCR1/TNFAIP3/TRIM15/ZC3H12A | | 18 |
|  | BP | GO:0032682 | negative regulation of chemokine production | 5/336 | 21/18670 | 2.94E-05 | 0.000719131 | 0.00050513 | EPHA2/IL10/IL6/KLF4/SIRPA | | 5 |
|  | BP | GO:0031348 | negative regulation of defense response | 15/336 | 239/18670 | 3.02E-05 | 0.000728823 | 0.000511938 | FPR2/IL10/IRAK3/KLF4/LDLR/MIR21/MIR223/SAA1/SERPINB9/SIRPA/SOCS3/TNFAIP3/TNFAIP6/TNFRSF1A/ZC3H12A | | 15 |
|  | BP | GO:2000116 | regulation of cysteine-type endopeptidase activity | 15/336 | 239/18670 | 3.02E-05 | 0.000728823 | 0.000511938 | BIRC3/CASP4/CRYAB/F3/GRAMD4/KLF4/MYC/PLAUR/PTGS2/S100A8/S100A9/SERPINB9/SOX7/THBS1/TNFSF10 | | 15 |
|  | BP | GO:0050714 | positive regulation of protein secretion | 16/336 | 268/18670 | 3.03E-05 | 0.000728823 | 0.000511938 | AKAP12/CCL3/CLEC4E/GCK/GJA1/IL10/IL1B/IL1RL1/IL4R/IL6/IRS2/OSM/PANX1/SAA1/TLR2/WNT5A | | 16 |
|  | BP | GO:0090130 | tissue migration | 19/336 | 360/18670 | 3.10E-05 | 0.000740268 | 0.000519978 | ACTG2/ANXA3/EDN1/EGR3/EPHA2/GLIPR2/HAS2/HBEGF/JUN/KLF4/MIR21/NR4A1/PTGS2/S100P/STC1/TACSTD2/THBS1/WNT5A/ZC3H12A | | 19 |
|  | BP | GO:0007369 | gastrulation | 13/336 | 185/18670 | 3.18E-05 | 0.000756388 | 0.0005313 | ACVR2B/EPHA2/ETS2/FOXC1/IL10/IL1RN/KDM6B/KLF4/NAT8B/NR4A3/SOX7/TRIM15/WNT5A | | 13 |
|  | BP | GO:0032680 | regulation of tumor necrosis factor production | 12/336 | 160/18670 | 3.38E-05 | 0.000798634 | 0.000560975 | AKAP12/BCL3/CCL3/IL10/IRAK3/SIRPA/THBS1/TLR2/TNFAIP3/WNT5A/ZC3H12A/ZFP36 | | 12 |
|  | BP | GO:0050868 | negative regulation of T cell activation | 10/336 | 112/18670 | 3.48E-05 | 0.000817193 | 0.000574011 | BCL6/DTX1/IL10/IL4R/IRF1/MIR21/PELI1/SDC4/SOCS1/ZC3H12A | | 10 |
|  | BP | GO:0051047 | positive regulation of secretion | 21/336 | 428/18670 | 3.50E-05 | 0.000817366 | 0.000574132 | AKAP12/AVPR1A/CCL3/CLEC4E/EDN1/GCK/GJA1/IL10/IL1B/IL1RL1/IL4R/IL6/INHBB/IRS2/OSM/PANX1/S100A8/SAA1/SDC4/TLR2/WNT5A | | 21 |
|  | BP | GO:0070374 | positive regulation of ERK1 and ERK2 cascade | 14/336 | 215/18670 | 3.70E-05 | 0.000860813 | 0.000604651 | AKAP12/BMP2/C5AR1/CCL2/CCL3/CCL8/CCR1/FPR2/GATA4/GLIPR2/ICAM1/JUN/MIR21/TNFAIP8L3 | | 14 |
|  | BP | GO:1903532 | positive regulation of secretion by cell | 20/336 | 399/18670 | 3.99E-05 | 0.000921275 | 0.00064712 | AKAP12/AVPR1A/CCL3/CLEC4E/EDN1/GCK/GJA1/IL10/IL1B/IL1RL1/IL4R/IL6/INHBB/IRS2/OSM/PANX1/SAA1/SDC4/TLR2/WNT5A | | 20 |
|  | BP | GO:0032640 | tumor necrosis factor production | 12/336 | 163/18670 | 4.06E-05 | 0.000927673 | 0.000651615 | AKAP12/BCL3/CCL3/IL10/IRAK3/SIRPA/THBS1/TLR2/TNFAIP3/WNT5A/ZC3H12A/ZFP36 | | 12 |
|  | BP | GO:1903555 | regulation of tumor necrosis factor superfamily cytokine production | 12/336 | 163/18670 | 4.06E-05 | 0.000927673 | 0.000651615 | AKAP12/BCL3/CCL3/IL10/IRAK3/SIRPA/THBS1/TLR2/TNFAIP3/WNT5A/ZC3H12A/ZFP36 | | 12 |
|  | BP | GO:0071241 | cellular response to inorganic substance | 14/336 | 217/18670 | 4.10E-05 | 0.000927673 | 0.000651615 | ALOX5AP/EDN1/FOS/FOSB/FOXO1/FSTL3/JUN/JUNB/MT1A/OGG1/PARP1/PTGS2/SYT12/WNT5A | | 14 |
|  | BP | GO:0046631 | alpha-beta T cell activation | 11/336 | 138/18670 | 4.10E-05 | 0.000927673 | 0.000651615 | BCL3/BCL6/CD83/IL4R/IL6/IRF1/MIR21/NFKBIZ/RELB/SOCS1/ZC3H12A | | 11 |
|  | BP | GO:0071248 | cellular response to metal ion | 13/336 | 190/18670 | 4.20E-05 | 0.000945676 | 0.00066426 | ALOX5AP/EDN1/FOS/FOSB/FSTL3/JUN/JUNB/MT1A/OGG1/PARP1/PTGS2/SYT12/WNT5A | | 13 |
|  | BP | GO:0035690 | cellular response to drug | 19/336 | 369/18670 | 4.33E-05 | 0.000966559 | 0.000678928 | EDN1/EGR1/FOXO1/ICAM1/IL10/IL18RAP/IL1B/IL6/KDM6B/KLF4/MIR21/MSX1/MYC/NR4A3/P2RY11/PTGS2/SIRPA/TNFAIP3/ZC3H12A | | 19 |
|  | BP | GO:0042326 | negative regulation of phosphorylation | 22/336 | 468/18670 | 4.34E-05 | 0.000966559 | 0.000678928 | ATF3/DUSP16/DUSP6/FOXO1/GADD45B/HEXIM2/IL1B/IRAK3/IRS2/ITPRIP/JUN/KLF4/MIDN/MIR21/MYC/RGS2/SIRPA/SOCS1/SOCS3/SPRY4/TNFAIP3/ZC3H12A | | 22 |
|  | BP | GO:1903524 | positive regulation of blood circulation | 8/336 | 73/18670 | 4.92E-05 | 0.001089 | 0.000764933 | AVPR1A/EDN1/GJA1/ICAM1/MIR21/PTGER2/PTGS2/RGS2 | | 8 |
|  | BP | GO:1901654 | response to ketone | 13/336 | 193/18670 | 4.94E-05 | 0.001089 | 0.000764933 | AVPR1A/EDN1/FOS/FOSB/FOSL1/FOXO1/ICAM1/KLF4/PARP1/PTGER2/THBS1/TLR2/TYMS | | 13 |
|  | BP | GO:0034614 | cellular response to reactive oxygen species | 12/336 | 168/18670 | 5.45E-05 | 0.001188624 | 0.000834911 | FOS/FOXO1/IL10/IL18RAP/IL6/JUN/KDM6B/KLF4/MIR21/NR4A3/SIRPA/TNFAIP3 | | 12 |
|  | BP | GO:0071706 | tumor necrosis factor superfamily cytokine production | 12/336 | 168/18670 | 5.45E-05 | 0.001188624 | 0.000834911 | AKAP12/BCL3/CCL3/IL10/IRAK3/SIRPA/THBS1/TLR2/TNFAIP3/WNT5A/ZC3H12A/ZFP36 | | 12 |
|  | BP | GO:0043551 | regulation of phosphatidylinositol 3-kinase activity | 7/336 | 55/18670 | 5.55E-05 | 0.001197934 | 0.000841451 | CISH/FPR2/KLF4/SOCS1/SOCS2/SOCS3/TNFAIP8L3 | | 7 |
|  | BP | GO:0045620 | negative regulation of lymphocyte differentiation | 7/336 | 55/18670 | 5.55E-05 | 0.001197934 | 0.000841451 | BCL6/DTX1/IL4R/IRF1/MIR21/SOCS1/ZC3H12A | | 7 |
|  | BP | GO:0050728 | negative regulation of inflammatory response | 12/336 | 169/18670 | 5.78E-05 | 0.001240003 | 0.000871001 | FPR2/IL10/KLF4/LDLR/MIR223/SAA1/SIRPA/SOCS3/TNFAIP3/TNFAIP6/TNFRSF1A/ZC3H12A | | 12 |
|  | BP | GO:0042832 | defense response to protozoan | 5/336 | 24/18670 | 5.88E-05 | 0.001255476 | 0.000881869 | BCL3/IL10/IL4R/IRF8/SLC11A1 | | 5 |
|  | BP | GO:1902107 | positive regulation of leukocyte differentiation | 11/336 | 144/18670 | 6.06E-05 | 0.001287682 | 0.000904491 | BCL6/CCR1/CD83/EGR3/FOS/IL4R/JUN/KLF10/MIR21/NFKBIZ/SOCS1 | | 11 |
|  | BP | GO:0031098 | stress-activated protein kinase signaling cascade | 17/336 | 315/18670 | 6.14E-05 | 0.001297807 | 0.000911603 | BMP2/CRYAB/EDAR/EDN1/ERN1/FOXO1/GADD45B/GADD45G/IL1B/IL1RN/MAP3K14/MAP3K8/MYC/SIRPA/WNT5A/ZC3H12A/ZFP36 | | 17 |
|  | BP | GO:1990868 | response to chemokine | 9/336 | 97/18670 | 6.33E-05 | 0.001325723 | 0.000931212 | CCL2/CCL3/CCL8/CCR1/CXCL1/CXCR1/EDN1/FOXC1/ZC3H12A | | 9 |
|  | BP | GO:1990869 | cellular response to chemokine | 9/336 | 97/18670 | 6.33E-05 | 0.001325723 | 0.000931212 | CCL2/CCL3/CCL8/CCR1/CXCL1/CXCR1/EDN1/FOXC1/ZC3H12A | | 9 |
|  | BP | GO:0031099 | regeneration | 13/336 | 198/18670 | 6.43E-05 | 0.001339721 | 0.000941044 | ANXA3/C5AR1/CD9/GATA4/GJA1/IGFBP1/IL10/JUN/KLF4/KLF5/NNMT/NR4A3/TYMS | | 13 |
|  | BP | GO:0051403 | stress-activated MAPK cascade | 16/336 | 286/18670 | 6.60E-05 | 0.001369154 | 0.000961719 | BMP2/CRYAB/EDAR/EDN1/ERN1/FOXO1/GADD45B/GADD45G/IL1B/IL1RN/MAP3K8/MYC/SIRPA/WNT5A/ZC3H12A/ZFP36 | | 16 |
|  | BP | GO:0045089 | positive regulation of innate immune response | 19/336 | 381/18670 | 6.65E-05 | 0.001370922 | 0.000962961 | BIRC3/CLEC4D/CLEC4E/FPR2/GRAMD4/IL18RAP/IRAK3/IRF1/MNDA/PELI1/PLSCR1/RELB/S100A8/S100A9/TLR2/TNFAIP3/TNIP3/TRIM15/WNT5A | | 19 |
|  | BP | GO:0001558 | regulation of cell growth | 20/336 | 416/18670 | 7.11E-05 | 0.001451923 | 0.001019857 | AVPR1A/BCL6/CISH/CRYAB/EDN1/FAM107A/GJA1/HBEGF/IGFBP1/MSX1/OSGIN2/RGS2/S100A8/S100A9/SEMA3C/SEMA4B/SOCS2/SUPV3L1/TNFRSF12A/WNT5A | | 20 |
|  | BP | GO:0030099 | myeloid cell differentiation | 20/336 | 416/18670 | 7.11E-05 | 0.001451923 | 0.001019857 | BCL6/CCL3/CCR1/EPHA2/FOS/FSTL3/IRF8/JUN/JUNB/KLF10/MIR223/MYC/NFE2/NR4A3/PARP1/RELB/SBNO2/THBS1/TLR2/ZFP36 | | 20 |
|  | BP | GO:0010631 | epithelial cell migration | 18/336 | 351/18670 | 7.23E-05 | 0.001457966 | 0.001024102 | ANXA3/EDN1/EGR3/EPHA2/GLIPR2/HAS2/HBEGF/JUN/KLF4/MIR21/NR4A1/PTGS2/S100P/STC1/TACSTD2/THBS1/WNT5A/ZC3H12A | | 18 |
|  | BP | GO:0006809 | nitric oxide biosynthetic process | 8/336 | 77/18670 | 7.24E-05 | 0.001457966 | 0.001024102 | EDN1/ICAM1/IL10/IL1B/KLF4/PTGS2/SIRPA/ZC3H12A | | 8 |
|  | BP | GO:0001562 | response to protozoan | 5/336 | 25/18670 | 7.24E-05 | 0.001457966 | 0.001024102 | BCL3/IL10/IL4R/IRF8/SLC11A1 | | 5 |
|  | BP | GO:0051249 | regulation of lymphocyte activation | 22/336 | 485/18670 | 7.35E-05 | 0.001472706 | 0.001034455 | BCL6/CCL2/CD83/DTX1/EGR3/IGFBP2/IL10/IL1B/IL4R/IL6/IRF1/IRS2/MAP3K8/MIR21/MNDA/NFKBIZ/PELI1/SDC4/SIRPA/SOCS1/TNFAIP3/ZC3H12A | | 22 |
|  | BP | GO:0048525 | negative regulation of viral process | 9/336 | 99/18670 | 7.43E-05 | 0.001477752 | 0.001038 | APOBEC3A/CCL3/CCL8/ISG20/JUN/PLSCR1/TRIM15/ZC3H12A/ZFP36 | | 9 |
|  | BP | GO:0042036 | negative regulation of cytokine biosynthetic process | 6/336 | 40/18670 | 7.44E-05 | 0.001477752 | 0.001038 | BCL3/IL10/IL6/INHBB/KLF4/ZFP36 | | 6 |
|  | BP | GO:0009743 | response to carbohydrate | 14/336 | 230/18670 | 7.70E-05 | 0.001514128 | 0.001063551 | ACVR2B/EGR1/ERN1/GATA4/GCK/GJA1/ICAM1/IL1B/IRS2/ME1/NAMPT/PIM3/PTGS2/THBS1 | | 14 |
|  | BP | GO:2001234 | negative regulation of apoptotic signaling pathway | 14/336 | 230/18670 | 7.70E-05 | 0.001514128 | 0.001063551 | BCL2A1/GATA4/ICAM1/IL1B/ITPRIP/MCL1/MIR21/NR4A2/PLAUR/PTGS2/SERPINE1/THBS1/TNFAIP3/TNFSF10 | | 14 |
|  | BP | GO:0007568 | aging | 17/336 | 321/18670 | 7.74E-05 | 0.001514128 | 0.001063551 | BCL2A1/BCL6/CRYAB/EDN1/FOS/ICAM1/IGFBP1/IGFBP2/IL10/JUN/MIR21/NAMPT/OGG1/PTGS2/SERPINE1/TYMS/ZKSCAN3 | | 17 |
|  | BP | GO:0061041 | regulation of wound healing | 11/336 | 148/18670 | 7.77E-05 | 0.001514128 | 0.001063551 | CD9/EDN1/F3/GJA1/HBEGF/PLAU/PLAUR/SERPINE1/THBS1/TNFAIP3/TNFRSF12A | | 11 |
|  | BP | GO:0032722 | positive regulation of chemokine production | 7/336 | 58/18670 | 7.86E-05 | 0.001515906 | 0.0010648 | EGR1/IL1B/IL1RL1/IL4R/IL6/TLR2/WNT5A | | 7 |
|  | BP | GO:1902041 | regulation of extrinsic apoptotic signaling pathway via death domain receptors | 7/336 | 58/18670 | 7.86E-05 | 0.001515906 | 0.0010648 | ATF3/ICAM1/ITPRIP/SERPINE1/THBS1/TNFAIP3/TNFSF10 | | 7 |
|  | BP | GO:0050870 | positive regulation of T cell activation | 13/336 | 202/18670 | 7.89E-05 | 0.001515906 | 0.0010648 | BCL6/CCL2/CD83/EGR3/IGFBP2/IL1B/IL4R/IL6/MAP3K8/MIR21/NFKBIZ/SIRPA/SOCS1 | | 13 |
|  | BP | GO:0090132 | epithelium migration | 18/336 | 354/18670 | 8.06E-05 | 0.001542899 | 0.00108376 | ANXA3/EDN1/EGR3/EPHA2/GLIPR2/HAS2/HBEGF/JUN/KLF4/MIR21/NR4A1/PTGS2/S100P/STC1/TACSTD2/THBS1/WNT5A/ZC3H12A | | 18 |
|  | BP | GO:0014074 | response to purine-containing compound | 11/336 | 149/18670 | 8.26E-05 | 0.001572781 | 0.00110475 | DHODH/FOS/FOSB/FOSL1/IL1B/JUN/JUNB/P2RY11/PANX1/PTGS2/STC1 | | 11 |
|  | BP | GO:0001936 | regulation of endothelial cell proliferation | 12/336 | 176/18670 | 8.54E-05 | 0.001620182 | 0.001138045 | BMP2/CCL2/EGR3/F3/GJA1/IL10/JUN/LRG1/MIR21/NR4A1/THBS1/WNT5A | | 12 |
|  | BP | GO:0032611 | interleukin-1 beta production | 9/336 | 101/18670 | 8.69E-05 | 0.001633279 | 0.001147245 | CCL3/EGR1/IL1B/MNDA/PANX1/SIRPA/TNFAIP3/WNT5A/ZC3H12A | | 9 |
|  | BP | GO:1903426 | regulation of reactive oxygen species biosynthetic process | 9/336 | 101/18670 | 8.69E-05 | 0.001633279 | 0.001147245 | EDN1/ICAM1/IL10/IL1B/KLF4/MIR21/PTGS2/SIRPA/ZC3H12A | | 9 |
|  | BP | GO:1903203 | regulation of oxidative stress-induced neuron death | 5/336 | 26/18670 | 8.83E-05 | 0.001652715 | 0.001160897 | IL10/MCL1/NCOA7/NR4A3/PARP1 | | 5 |
|  | BP | GO:0072503 | cellular divalent inorganic cation homeostasis | 22/336 | 493/18670 | 9.32E-05 | 0.001736083 | 0.001219456 | ANK2/AVPR1A/C5AR1/CALCA/CCL3/CCL8/CCR1/CXCR1/EDN1/FPR1/FPR2/GJA1/LPAR1/MT1A/PROK2/PTGER2/S100A8/S100A9/SAA1/SLC11A1/STC1/WNT5A | | 22 |
|  | BP | GO:0000187 | activation of MAPK activity | 11/336 | 152/18670 | 9.88E-05 | 0.001831959 | 0.001286801 | BMP2/C5AR1/DUSP6/ERN1/FPR1/IL1B/LPAR1/PROK2/SAA1/THBS1/WNT5A | | 11 |
|  | BP | GO:0002064 | epithelial cell development | 13/336 | 207/18670 | 0.000100995 | 0.001865101 | 0.001310081 | B4GALT1/EPHA2/FOSL2/FRMD6/GJA1/ICAM1/IL1B/KLF5/ONECUT1/STC1/TNFRSF1A/TYMS/WNT5A | | 13 |
|  | BP | GO:0070482 | response to oxygen levels | 19/336 | 394/18670 | 0.000103355 | 0.001900392 | 0.00133487 | APOLD1/BMP2/CRYAB/EDN1/EGLN1/EGR1/FOXO1/ICAM1/LPAR1/MIR21/MYC/NAMPT/NR4A2/PLAU/PTGS2/SIRT4/STC1/THBS1/TLR2 | | 19 |
|  | BP | GO:0002825 | regulation of T-helper 1 type immune response | 5/336 | 27/18670 | 0.000106807 | 0.001938563 | 0.001361682 | IL1B/IL1R1/IL1RL1/IL4R/SLC11A1 | | 5 |
|  | BP | GO:0036475 | neuron death in response to oxidative stress | 5/336 | 27/18670 | 0.000106807 | 0.001938563 | 0.001361682 | IL10/MCL1/NCOA7/NR4A3/PARP1 | | 5 |
|  | BP | GO:0048143 | astrocyte activation | 5/336 | 27/18670 | 0.000106807 | 0.001938563 | 0.001361682 | C5AR1/FPR2/IL1B/IL6/LDLR | | 5 |
|  | BP | GO:0001933 | negative regulation of protein phosphorylation | 20/336 | 429/18670 | 0.000107987 | 0.001951616 | 0.001370851 | ATF3/DUSP16/DUSP6/FOXO1/GADD45B/HEXIM2/IL1B/IRAK3/ITPRIP/JUN/KLF4/MIR21/MYC/RGS2/SIRPA/SOCS1/SOCS3/SPRY4/TNFAIP3/ZC3H12A | | 20 |
|  | BP | GO:2000514 | regulation of CD4-positive, alpha-beta T cell activation | 7/336 | 61/18670 | 0.000108846 | 0.001954416 | 0.001372817 | BCL6/CD83/IL4R/MIR21/NFKBIZ/SOCS1/ZC3H12A | | 7 |
|  | BP | GO:0090100 | positive regulation of transmembrane receptor protein serine/threonine kinase signaling pathway | 9/336 | 104/18670 | 0.000109066 | 0.001954416 | 0.001372817 | ACVR2B/BMP2/GATA4/INHBB/INHBE/LRG1/MSX1/PARP1/THBS1 | | 9 |
|  | BP | GO:0010038 | response to metal ion | 18/336 | 364/18670 | 0.000114592 | 0.002044766 | 0.00143628 | ALOX5AP/ARL13B/EDN1/FOS/FOSB/FSTL3/ICAM1/IGFBP2/JUN/JUNB/MT1A/OGG1/PARP1/PTGS2/S100A8/SYT12/THBS1/WNT5A | | 18 |
|  | BP | GO:1903707 | negative regulation of hemopoiesis | 11/336 | 155/18670 | 0.000117613 | 0.002089859 | 0.001467955 | BCL6/CCL3/DTX1/FSTL3/IL4R/IRF1/MIR21/MYC/SOCS1/ZC3H12A/ZFP36 | | 11 |
|  | BP | GO:0045073 | regulation of chemokine biosynthetic process | 4/336 | 15/18670 | 0.000120226 | 0.002118489 | 0.001488065 | EGR1/IL1B/IL6/WNT5A | | 4 |
|  | BP | GO:0051770 | positive regulation of nitric-oxide synthase biosynthetic process | 4/336 | 15/18670 | 0.000120226 | 0.002118489 | 0.001488065 | AKAP12/CCL2/NAMPT/TLR2 | | 4 |
|  | BP | GO:0071277 | cellular response to calcium ion | 8/336 | 83/18670 | 0.000123587 | 0.002164023 | 0.001520049 | ALOX5AP/EDN1/FOS/FOSB/JUN/JUNB/SYT12/WNT5A | | 8 |
|  | BP | GO:0043123 | positive regulation of I-kappaB kinase/NF-kappaB signaling | 12/336 | 183/18670 | 0.000123834 | 0.002164023 | 0.001520049 | BIRC3/EDAR/GJA1/IL1B/IL1RN/LPAR1/MAP3K14/PELI1/PIM2/S100A12/TNFRSF1A/TNFSF10 | | 12 |
|  | BP | GO:0071902 | positive regulation of protein serine/threonine kinase activity | 17/336 | 334/18670 | 0.000125262 | 0.002179979 | 0.001531257 | BMP2/C5AR1/DUSP6/EDN1/ERN1/FPR1/GADD45B/GADD45G/IL1B/LPAR1/MAP3K14/MAP3K8/PROK2/S100A12/SAA1/THBS1/WNT5A | | 17 |
|  | BP | GO:0042136 | neurotransmitter biosynthetic process | 9/336 | 106/18670 | 0.000126333 | 0.002181902 | 0.001532608 | EDN1/ICAM1/IL10/IL1B/KLF4/PTGS2/SIRPA/SLC1A3/ZC3H12A | | 9 |
|  | BP | GO:0090092 | regulation of transmembrane receptor protein serine/threonine kinase signaling pathway | 14/336 | 241/18670 | 0.000126405 | 0.002181902 | 0.001532608 | ACVR2B/BMP2/FSTL3/GATA4/INHBB/INHBE/LRG1/MSX1/NBL1/ONECUT1/PARP1/PEG10/THBS1/WNT5A | | 14 |
|  | BP | GO:0014002 | astrocyte development | 6/336 | 44/18670 | 0.000128852 | 0.002215094 | 0.001555922 | C5AR1/FPR2/IL1B/IL6/LDLR/S100A8 | | 6 |
|  | BP | GO:0043409 | negative regulation of MAPK cascade | 12/336 | 184/18670 | 0.000130375 | 0.00223221 | 0.001567945 | ATF3/DUSP16/DUSP6/FOXO1/IL1B/IRAK3/KLF4/MIR21/MYC/RGS2/SIRPA/SPRY4 | | 12 |
|  | BP | GO:0046637 | regulation of alpha-beta T cell differentiation | 7/336 | 63/18670 | 0.000133862 | 0.002275033 | 0.001598025 | BCL6/CD83/IL4R/MIR21/NFKBIZ/SOCS1/ZC3H12A | | 7 |
|  | BP | GO:0002718 | regulation of cytokine production involved in immune response | 8/336 | 84/18670 | 0.00013449 | 0.002275033 | 0.001598025 | BCL6/IL10/IL1B/IL1R1/IL6/IRAK3/NR4A3/WNT5A | | 8 |
|  | BP | GO:0014910 | regulation of smooth muscle cell migration | 8/336 | 84/18670 | 0.00013449 | 0.002275033 | 0.001598025 | ADAMTS1/HAS2/LPAR1/MIR21/MIR223/NR4A3/PLAU/SERPINE1 | | 8 |
|  | BP | GO:1903708 | positive regulation of hemopoiesis | 12/336 | 185/18670 | 0.00013721 | 0.002311797 | 0.001623848 | BCL6/CCR1/CD83/EGR3/FOS/FOXC1/IL4R/JUN/KLF10/MIR21/NFKBIZ/SOCS1 | | 12 |
|  | BP | GO:1904705 | regulation of vascular smooth muscle cell proliferation | 8/336 | 85/18670 | 0.000146174 | 0.002443358 | 0.001716259 | ADAMTS1/EDN1/ERN1/IL10/JUN/MIR21/MIR223/NR4A3 | | 8 |
|  | BP | GO:1990874 | vascular smooth muscle cell proliferation | 8/336 | 85/18670 | 0.000146174 | 0.002443358 | 0.001716259 | ADAMTS1/EDN1/ERN1/IL10/JUN/MIR21/MIR223/NR4A3 | | 8 |
|  | BP | GO:0043550 | regulation of lipid kinase activity | 7/336 | 64/18670 | 0.000148011 | 0.002454666 | 0.001724202 | CISH/FPR2/KLF4/SOCS1/SOCS2/SOCS3/TNFAIP8L3 | | 7 |
|  | BP | GO:0050922 | negative regulation of chemotaxis | 7/336 | 64/18670 | 0.000148011 | 0.002454666 | 0.001724202 | CCL2/MIR223/NBL1/SEMA3C/SEMA4B/THBS1/WNT5A | | 7 |
|  | BP | GO:0010575 | positive regulation of vascular endothelial growth factor production | 5/336 | 29/18670 | 0.000152546 | 0.002519982 | 0.001770081 | C5AR1/GATA4/IL1B/IL6/PTGS2 | | 5 |
|  | BP | GO:0042033 | chemokine biosynthetic process | 4/336 | 16/18670 | 0.000158035 | 0.002590429 | 0.001819564 | EGR1/IL1B/IL6/WNT5A | | 4 |
|  | BP | GO:0050755 | chemokine metabolic process | 4/336 | 16/18670 | 0.000158035 | 0.002590429 | 0.001819564 | EGR1/IL1B/IL6/WNT5A | | 4 |
|  | BP | GO:0008625 | extrinsic apoptotic signaling pathway via death domain receptors | 8/336 | 86/18670 | 0.000158682 | 0.002590985 | 0.001819955 | ATF3/ICAM1/ITPRIP/SERPINE1/THBS1/TNFAIP3/TNFRSF1A/TNFSF10 | | 8 |
|  | BP | GO:0032635 | interleukin-6 production | 11/336 | 161/18670 | 0.000164626 | 0.002677677 | 0.001880849 | IL10/IL18RAP/IL1B/IL1RN/IL6/IRAK3/SIRPA/TLR2/TNFAIP3/WNT5A/ZC3H12A | | 11 |
|  | BP | GO:0060485 | mesenchyme development | 15/336 | 278/18670 | 0.000165503 | 0.002677677 | 0.001880849 | ACTG2/BMP2/EDN1/FOXC1/GATA4/GLIPR2/HAS2/IL1B/IL6/MIR21/MSX1/MYC/SEMA3C/SEMA4B/WNT5A | | 15 |
|  | BP | GO:0071675 | regulation of mononuclear cell migration | 6/336 | 46/18670 | 0.000165891 | 0.002677677 | 0.001880849 | C5AR1/CCR1/FPR2/NBL1/SERPINE1/THBS1 | | 6 |
|  | BP | GO:0007517 | muscle organ development | 19/336 | 410/18670 | 0.000172815 | 0.002778847 | 0.001951912 | ARID5B/ATF3/BMP2/BTG2/CRYAB/EDN1/EGR1/EGR3/FOS/FOXC1/GJA1/HBEGF/KLF5/MAFF/MSX1/PIM1/RGS2/TAGLN/WNT5A | | 19 |
|  | BP | GO:0046888 | negative regulation of hormone secretion | 7/336 | 66/18670 | 0.000179947 | 0.00287852 | 0.002021925 | EDN1/IL1B/INHBB/MIDN/OSM/PIM3/SIRT4 | | 7 |
|  | BP | GO:0043372 | positive regulation of CD4-positive, alpha-beta T cell differentiation | 5/336 | 30/18670 | 0.000180375 | 0.00287852 | 0.002021925 | CD83/IL4R/MIR21/NFKBIZ/SOCS1 | | 5 |
|  | BP | GO:0042594 | response to starvation | 12/336 | 191/18670 | 0.000185016 | 0.002940527 | 0.002065479 | ATF3/DHODH/FADS1/FOXO1/INHBB/JUN/KLF10/MAP1LC3B/RNF152/SIK1/ZC3H12A/ZFP36 | | 12 |
|  | BP | GO:0070098 | chemokine-mediated signaling pathway | 8/336 | 88/18670 | 0.000186347 | 0.002940527 | 0.002065479 | CCL2/CCL3/CCL8/CCR1/CXCL1/CXCR1/EDN1/FOXC1 | | 8 |
|  | BP | GO:1903725 | regulation of phospholipid metabolic process | 8/336 | 88/18670 | 0.000186347 | 0.002940527 | 0.002065479 | CISH/FPR2/KLF4/LDLR/SOCS1/SOCS2/SOCS3/TNFAIP8L3 | | 8 |
|  | BP | GO:0006953 | acute-phase response | 6/336 | 47/18670 | 0.000187319 | 0.002944879 | 0.002068536 | IL1B/IL6/PLSCR1/PTGS2/SAA1/SAA2 | | 6 |
|  | BP | GO:0001938 | positive regulation of endothelial cell proliferation | 9/336 | 112/18670 | 0.000192401 | 0.003013563 | 0.002116781 | BMP2/EGR3/F3/IL10/JUN/LRG1/MIR21/NR4A1/WNT5A | | 9 |
|  | BP | GO:0016049 | cell growth | 21/336 | 484/18670 | 0.000197238 | 0.003077926 | 0.002161991 | AVPR1A/BCL6/CISH/CRYAB/EDN1/FAM107A/GATA4/GJA1/HBEGF/IGFBP1/MSX1/OSGIN2/RGS2/S100A8/S100A9/SEMA3C/SEMA4B/SOCS2/SUPV3L1/TNFRSF12A/WNT5A | | 21 |
|  | BP | GO:0070663 | regulation of leukocyte proliferation | 13/336 | 222/18670 | 0.000202481 | 0.003148125 | 0.0022113 | BCL6/CCL8/IGFBP2/IL10/IL1B/IL6/IRF1/IRS2/MIR21/MNDA/PELI1/SDC4/TNFAIP3 | | 13 |
|  | BP | GO:0072593 | reactive oxygen species metabolic process | 15/336 | 284/18670 | 0.000208694 | 0.003232844 | 0.002270808 | CRYAB/EDN1/FOXO1/FPR2/ICAM1/IL10/IL1B/KLF4/MIR21/PTGS2/SIRPA/STK17A/THBS1/TLR2/ZC3H12A | | 15 |
|  | BP | GO:0060324 | face development | 6/336 | 48/18670 | 0.000210874 | 0.003254689 | 0.002286153 | ALDH1A3/ARID5B/CRISPLD2/MSX1/TIPARP/WNT5A | | 6 |
|  | BP | GO:1902895 | positive regulation of pri-miRNA transcription by RNA polymerase II | 5/336 | 31/18670 | 0.000211917 | 0.003258892 | 0.002289105 | BMP2/FOS/IL10/JUN/KLF5 | | 5 |
|  | BP | GO:0045580 | regulation of T cell differentiation | 10/336 | 139/18670 | 0.000213624 | 0.003273253 | 0.002299192 | BCL6/CD83/DTX1/EGR3/IL4R/IRF1/MIR21/NFKBIZ/SOCS1/ZC3H12A | | 10 |
|  | BP | GO:0051348 | negative regulation of transferase activity | 15/336 | 285/18670 | 0.000216768 | 0.003309422 | 0.002324598 | DUSP16/DUSP6/GADD45B/HEXIM2/IL1B/IRAK3/IRS2/ITPRIP/MIDN/RGS2/SOCS1/SOCS3/SPRY4/TNFAIP3/ZFP36 | | 15 |
|  | BP | GO:0032651 | regulation of interleukin-1 beta production | 8/336 | 90/18670 | 0.000217857 | 0.003314092 | 0.002327879 | CCL3/EGR1/MNDA/PANX1/SIRPA/TNFAIP3/WNT5A/ZC3H12A | | 8 |
|  | BP | GO:0030282 | bone mineralization | 9/336 | 114/18670 | 0.000219967 | 0.003334191 | 0.002341996 | ACVR2B/BMP2/CCL3/CCR1/GJA1/GPM6B/KLF10/PTGS2/SBNO2 | | 9 |
|  | BP | GO:0051222 | positive regulation of protein transport | 19/336 | 418/18670 | 0.000220918 | 0.003336647 | 0.002343722 | ABLIM3/AKAP12/CCL3/CLEC4E/GCK/GJA1/IL10/IL1B/IL1RL1/IL4R/IL6/IRS2/OSM/PANX1/PTGS2/SAA1/TLR2/WNT5A/ZC3H12A | | 19 |
|  | BP | GO:0014909 | smooth muscle cell migration | 8/336 | 91/18670 | 0.000235177 | 0.003539373 | 0.00248612 | ADAMTS1/HAS2/LPAR1/MIR21/MIR223/NR4A3/PLAU/SERPINE1 | | 8 |
|  | BP | GO:0033673 | negative regulation of kinase activity | 14/336 | 257/18670 | 0.000246325 | 0.003672365 | 0.002579536 | DUSP16/DUSP6/GADD45B/HEXIM2/IL1B/IRAK3/IRS2/ITPRIP/MIDN/RGS2/SOCS1/SOCS3/SPRY4/TNFAIP3 | | 14 |
|  | BP | GO:0002221 | pattern recognition receptor signaling pathway | 12/336 | 197/18670 | 0.000246376 | 0.003672365 | 0.002579536 | BIRC3/CLEC4E/GRAMD4/IRAK3/IRF1/PELI1/S100A8/S100A9/TLR2/TNFAIP3/TNIP3/TRIM15 | | 12 |
|  | BP | GO:0045907 | positive regulation of vasoconstriction | 5/336 | 32/18670 | 0.000247487 | 0.003672365 | 0.002579536 | AVPR1A/GJA1/ICAM1/MIR21/PTGS2 | | 5 |
|  | BP | GO:1900745 | positive regulation of p38MAPK cascade | 5/336 | 32/18670 | 0.000247487 | 0.003672365 | 0.002579536 | BMP2/GADD45B/GADD45G/IL1B/ZC3H12A | | 5 |
|  | BP | GO:0001505 | regulation of neurotransmitter levels | 17/336 | 354/18670 | 0.000249435 | 0.003676211 | 0.002582238 | EDN1/GPM6B/ICAM1/IL10/IL1B/KLF4/PTGS2/RGS2/RNF180/SIRPA/SLC1A3/SLC25A32/STX11/STX3/SYT12/TLR2/ZC3H12A | | 17 |
|  | BP | GO:0071375 | cellular response to peptide hormone stimulus | 16/336 | 321/18670 | 0.000249485 | 0.003676211 | 0.002582238 | CISH/EDN1/FOXO1/GCK/IGFBP1/IL1B/INHBB/IRS2/LPIN2/NR4A1/NR4A2/NR4A3/PARP1/SOCS1/SOCS2/SOCS3 | | 16 |
|  | BP | GO:0021782 | glial cell development | 9/336 | 116/18670 | 0.000250737 | 0.003681824 | 0.00258618 | C5AR1/CD9/FPR2/IL1B/IL6/LDLR/LPAR1/S100A8/TLR2 | | 9 |
|  | BP | GO:0097193 | intrinsic apoptotic signaling pathway | 15/336 | 289/18670 | 0.000251825 | 0.003685016 | 0.002588422 | BCL2A1/BCL3/CASP4/EPHA2/ERN1/MCL1/MIR21/MSX1/PARP1/PLAUR/PPP1R15A/PTGS2/S100A8/S100A9/TNFRSF1A | | 15 |
|  | BP | GO:0034121 | regulation of toll-like receptor signaling pathway | 7/336 | 70/18670 | 0.000260511 | 0.003798969 | 0.002668465 | BIRC3/GRAMD4/IRAK3/IRF1/PELI1/TLR2/TNFAIP3 | | 7 |
|  | BP | GO:0010718 | positive regulation of epithelial to mesenchymal transition | 6/336 | 50/18670 | 0.000264954 | 0.003850484 | 0.00270465 | BMP2/FOXC1/GLIPR2/IL1B/IL6/MIR21 | | 6 |
|  | BP | GO:0090257 | regulation of muscle system process | 14/336 | 259/18670 | 0.000266638 | 0.00386168 | 0.002712514 | ANK2/EDN1/FOXO1/GATA4/KLF4/MIR21/NR4A3/PARP1/PROK2/PTGS2/RGS2/STC1/TNFRSF1A/ZC3H12A | | 14 |
|  | BP | GO:0010632 | regulation of epithelial cell migration | 15/336 | 291/18670 | 0.000271119 | 0.003913188 | 0.002748694 | ANXA3/EDN1/EPHA2/GLIPR2/HAS2/HBEGF/JUN/KLF4/MIR21/PTGS2/STC1/TACSTD2/THBS1/WNT5A/ZC3H12A | | 15 |
|  | BP | GO:0046634 | regulation of alpha-beta T cell activation | 8/336 | 93/18670 | 0.000273207 | 0.003929905 | 0.002760437 | BCL6/CD83/IL4R/IRF1/MIR21/NFKBIZ/SOCS1/ZC3H12A | | 8 |
|  | BP | GO:0010634 | positive regulation of epithelial cell migration | 11/336 | 171/18670 | 0.000278281 | 0.00398933 | 0.002802178 | ANXA3/EDN1/GLIPR2/HAS2/HBEGF/JUN/MIR21/PTGS2/THBS1/WNT5A/ZC3H12A | | 11 |
|  | BP | GO:0003018 | vascular process in circulatory system | 11/336 | 173/18670 | 0.000307566 | 0.004394248 | 0.0030866 | AKAP12/AVPR1A/C2CD4A/C2CD4B/EDN1/FOXC1/GJA1/ICAM1/MIR21/PTGS2/RGS2 | | 11 |
|  | BP | GO:0009746 | response to hexose | 12/336 | 202/18670 | 0.00030999 | 0.004413964 | 0.003100448 | ACVR2B/EGR1/ERN1/GATA4/GCK/GJA1/ICAM1/IRS2/NAMPT/PIM3/PTGS2/THBS1 | | 12 |
|  | BP | GO:0060337 | type I interferon signaling pathway | 8/336 | 95/18670 | 0.000316122 | 0.004464802 | 0.003136158 | EGR1/IFIT2/IFIT3/IRF1/IRF8/ISG20/MIR21/WNT5A | | 8 |
|  | BP | GO:0071357 | cellular response to type I interferon | 8/336 | 95/18670 | 0.000316122 | 0.004464802 | 0.003136158 | EGR1/IFIT2/IFIT3/IRF1/IRF8/ISG20/MIR21/WNT5A | | 8 |
|  | BP | GO:0002224 | toll-like receptor signaling pathway | 10/336 | 146/18670 | 0.000317783 | 0.004464802 | 0.003136158 | BIRC3/GRAMD4/IRAK3/IRF1/PELI1/S100A8/S100A9/TLR2/TNFAIP3/TNIP3 | | 10 |
|  | BP | GO:0045834 | positive regulation of lipid metabolic process | 10/336 | 146/18670 | 0.000317783 | 0.004464802 | 0.003136158 | AVPR1A/FPR2/IL1B/IRS2/LDLR/NR4A3/PTGS2/SIRT4/TNFAIP8L3/TNFRSF1A | | 10 |
|  | BP | GO:0097305 | response to alcohol | 13/336 | 233/18670 | 0.000323911 | 0.004535827 | 0.003186047 | AVPR1A/CCL3/FOS/FOSB/FOSL1/ICAM1/KLF4/OGG1/PARP1/PTGER2/RGS2/S100A8/TYMS | | 13 |
|  | BP | GO:0072132 | mesenchyme morphogenesis | 6/336 | 52/18670 | 0.000329371 | 0.004597064 | 0.003229061 | ACTG2/BMP2/FOXC1/MSX1/MYC/WNT5A | | 6 |
|  | BP | GO:0010574 | regulation of vascular endothelial growth factor production | 5/336 | 34/18670 | 0.000332049 | 0.004619191 | 0.003244603 | C5AR1/GATA4/IL1B/IL6/PTGS2 | | 5 |
|  | BP | GO:1903522 | regulation of blood circulation | 15/336 | 297/18670 | 0.000336847 | 0.004670576 | 0.003280698 | ANK2/AVPR1A/EDN1/GATA4/GJA1/HBEGF/ICAM1/KCNE4/KCNK1/MIR21/PTGER2/PTGS2/RGS2/STC1/ZC3H12A | | 15 |
|  | BP | GO:0001503 | ossification | 18/336 | 398/18670 | 0.000342003 | 0.00472657 | 0.003320029 | ACVR2B/BMP2/CCL3/CCR1/CEBPD/EPHA2/FOXC1/FSTL3/GJA1/GPM6B/IL6/JUNB/KLF10/MIR21/PTGS2/SBNO2/STC1/WNT5A | | 18 |
|  | BP | GO:0001704 | formation of primary germ layer | 9/336 | 121/18670 | 0.000343576 | 0.004732837 | 0.00332443 | EPHA2/ETS2/FOXC1/KDM6B/KLF4/NR4A3/SOX7/TRIM15/WNT5A | | 9 |
|  | BP | GO:0070661 | leukocyte proliferation | 15/336 | 298/18670 | 0.000349037 | 0.004792462 | 0.003366313 | BCL6/CCL8/GJA1/IGFBP2/IL10/IL1B/IL6/IRF1/IRS2/MIR21/MNDA/PELI1/SDC4/SLC11A1/TNFAIP3 | | 15 |
|  | BP | GO:0030316 | osteoclast differentiation | 8/336 | 97/18670 | 0.000364385 | 0.004911902 | 0.003450209 | CCL3/CCR1/EPHA2/FOS/FSTL3/JUNB/KLF10/SBNO2 | | 8 |
|  | BP | GO:0043500 | muscle adaptation | 9/336 | 122/18670 | 0.000365181 | 0.004911902 | 0.003450209 | EDN1/FOXO1/IL1B/KLF4/MIR21/NR4A3/PARP1/RGS2/TNFRSF1A | | 9 |
|  | BP | GO:1903409 | reactive oxygen species biosynthetic process | 9/336 | 122/18670 | 0.000365181 | 0.004911902 | 0.003450209 | EDN1/ICAM1/IL10/IL1B/KLF4/MIR21/PTGS2/SIRPA/ZC3H12A | | 9 |
|  | BP | GO:0030195 | negative regulation of blood coagulation | 6/336 | 53/18670 | 0.000365866 | 0.004911902 | 0.003450209 | CD9/EDN1/PLAU/PLAUR/SERPINE1/THBS1 | | 6 |
|  | BP | GO:0038066 | p38MAPK cascade | 6/336 | 53/18670 | 0.000365866 | 0.004911902 | 0.003450209 | BMP2/GADD45B/GADD45G/IL1B/ZC3H12A/ZFP36 | | 6 |
|  | BP | GO:0050704 | regulation of interleukin-1 secretion | 6/336 | 53/18670 | 0.000365866 | 0.004911902 | 0.003450209 | CCL3/PANX1/SAA1/TNFAIP3/WNT5A/ZC3H12A | | 6 |
|  | BP | GO:1905517 | macrophage migration | 6/336 | 53/18670 | 0.000365866 | 0.004911902 | 0.003450209 | C5AR1/CCL2/CCL3/CD9/SAA1/THBS1 | | 6 |
|  | BP | GO:0032677 | regulation of interleukin-8 production | 7/336 | 74/18670 | 0.000367833 | 0.004922679 | 0.003457779 | BCL3/IL10/IL1B/KLF4/SERPINE1/TLR2/WNT5A | | 7 |
|  | BP | GO:0009267 | cellular response to starvation | 10/336 | 149/18670 | 0.00037389 | 0.004972263 | 0.003492607 | ATF3/FADS1/FOXO1/INHBB/JUN/KLF10/MAP1LC3B/RNF152/SIK1/ZC3H12A | | 10 |
|  | BP | GO:0051092 | positive regulation of NF-kappaB transcription factor activity | 10/336 | 149/18670 | 0.00037389 | 0.004972263 | 0.003492607 | ICAM1/IL18RAP/IL1B/IRAK3/S100A12/S100A8/S100A9/TLR2/TRIM15/WNT5A | | 10 |
|  | BP | GO:0032872 | regulation of stress-activated MAPK cascade | 13/336 | 237/18670 | 0.000381312 | 0.005029112 | 0.00353254 | BMP2/EDAR/EDN1/ERN1/FOXO1/GADD45B/GADD45G/IL1B/IL1RN/MYC/SIRPA/WNT5A/ZC3H12A | | 13 |
|  | BP | GO:0042092 | type 2 immune response | 5/336 | 35/18670 | 0.000381732 | 0.005029112 | 0.00353254 | BCL3/BCL6/IL10/IL4R/IL6 | | 5 |
|  | BP | GO:0045622 | regulation of T-helper cell differentiation | 5/336 | 35/18670 | 0.000381732 | 0.005029112 | 0.00353254 | BCL6/IL4R/MIR21/NFKBIZ/ZC3H12A | | 5 |
|  | BP | GO:0034284 | response to monosaccharide | 12/336 | 207/18670 | 0.000387022 | 0.00508297 | 0.00357037 | ACVR2B/EGR1/ERN1/GATA4/GCK/GJA1/ICAM1/IRS2/NAMPT/PIM3/PTGS2/THBS1 | | 12 |
|  | BP | GO:0043491 | protein kinase B signaling | 14/336 | 269/18670 | 0.00039121 | 0.005122068 | 0.003597834 | CCL2/CCL3/EPHA2/F3/FAM110C/GATA4/HBEGF/IL1B/IRS2/KLF4/MIR21/OSM/THBS1/TNFAIP8L3 | | 14 |
|  | BP | GO:0051607 | defense response to virus | 13/336 | 238/18670 | 0.000396944 | 0.005167083 | 0.003629453 | APOBEC3A/BIRC3/IFIT2/IFIT3/IL1B/IL6/IRF1/ISG20/MAP3K14/PLSCR1/TNFAIP3/TRIM15/ZC3H12A | | 13 |
|  | BP | GO:0001707 | mesoderm formation | 7/336 | 75/18670 | 0.000399536 | 0.005167083 | 0.003629453 | EPHA2/FOXC1/KDM6B/KLF4/NR4A3/TRIM15/WNT5A | | 7 |
|  | BP | GO:0010611 | regulation of cardiac muscle hypertrophy | 7/336 | 75/18670 | 0.000399536 | 0.005167083 | 0.003629453 | EDN1/FOXO1/MIR21/NR4A3/PARP1/RGS2/TNFRSF1A | | 7 |
|  | BP | GO:1903201 | regulation of oxidative stress-induced cell death | 7/336 | 75/18670 | 0.000399536 | 0.005167083 | 0.003629453 | GATA4/IL10/MCL1/MIR21/NCOA7/NR4A3/PARP1 | | 7 |
|  | BP | GO:0006636 | unsaturated fatty acid biosynthetic process | 6/336 | 54/18670 | 0.000405449 | 0.005195892 | 0.003649689 | ALOX5AP/AVPR1A/EDN1/FADS1/IL1B/PTGS2 | | 6 |
|  | BP | GO:0032655 | regulation of interleukin-12 production | 6/336 | 54/18670 | 0.000405449 | 0.005195892 | 0.003649689 | IL10/IRAK3/IRF1/IRF8/THBS1/TLR2 | | 6 |
|  | BP | GO:1900047 | negative regulation of hemostasis | 6/336 | 54/18670 | 0.000405449 | 0.005195892 | 0.003649689 | CD9/EDN1/PLAU/PLAUR/SERPINE1/THBS1 | | 6 |
|  | BP | GO:0070302 | regulation of stress-activated protein kinase signaling cascade | 13/336 | 239/18670 | 0.000413117 | 0.005278161 | 0.003707476 | BMP2/EDAR/EDN1/ERN1/FOXO1/GADD45B/GADD45G/IL1B/IL1RN/MYC/SIRPA/WNT5A/ZC3H12A | | 13 |
|  | BP | GO:0036293 | response to decreased oxygen levels | 17/336 | 370/18670 | 0.00041536 | 0.005290838 | 0.00371638 | APOLD1/BMP2/CRYAB/EDN1/EGLN1/EGR1/ICAM1/MIR21/MYC/NAMPT/NR4A2/PLAU/PTGS2/SIRT4/STC1/THBS1/TLR2 | | 17 |
|  | BP | GO:0034340 | response to type I interferon | 8/336 | 99/18670 | 0.000418484 | 0.005314625 | 0.003733089 | EGR1/IFIT2/IFIT3/IRF1/IRF8/ISG20/MIR21/WNT5A | | 8 |
|  | BP | GO:0061045 | negative regulation of wound healing | 7/336 | 76/18670 | 0.000433385 | 0.005480388 | 0.003849524 | CD9/EDN1/GJA1/PLAU/PLAUR/SERPINE1/THBS1 | | 7 |
|  | BP | GO:0010573 | vascular endothelial growth factor production | 5/336 | 36/18670 | 0.00043683 | 0.005480388 | 0.003849524 | C5AR1/GATA4/IL1B/IL6/PTGS2 | | 5 |
|  | BP | GO:0046627 | negative regulation of insulin receptor signaling pathway | 5/336 | 36/18670 | 0.00043683 | 0.005480388 | 0.003849524 | CISH/IL1B/SOCS1/SOCS2/SOCS3 | | 5 |
|  | BP | GO:2000516 | positive regulation of CD4-positive, alpha-beta T cell activation | 5/336 | 36/18670 | 0.00043683 | 0.005480388 | 0.003849524 | CD83/IL4R/MIR21/NFKBIZ/SOCS1 | | 5 |
|  | BP | GO:0032675 | regulation of interleukin-6 production | 10/336 | 152/18670 | 0.000438016 | 0.005480388 | 0.003849524 | IL10/IL1B/IL1RN/IL6/IRAK3/SIRPA/TLR2/TNFAIP3/WNT5A/ZC3H12A | | 10 |
|  | BP | GO:0070498 | interleukin-1-mediated signaling pathway | 8/336 | 100/18670 | 0.000447882 | 0.005576031 | 0.003916705 | EGR1/IL1B/IL1R1/IL1RN/IL6/IRAK3/MAP3K8/PELI1 | | 8 |
|  | BP | GO:0046456 | icosanoid biosynthetic process | 6/336 | 55/18670 | 0.000448298 | 0.005576031 | 0.003916705 | ALOX5AP/AVPR1A/EDN1/FADS1/IL1B/PTGS2 | | 6 |
|  | BP | GO:0060537 | muscle tissue development | 18/336 | 408/18670 | 0.000459109 | 0.005693761 | 0.003999401 | ATF3/BMP2/BTG2/EDN1/EGR1/FOS/FOXC1/GATA4/GJA1/KDM6B/KLF5/MAFF/PIM1/RGS2/SEMA3C/SIK1/TIPARP/WNT5A | | 18 |
|  | BP | GO:0048332 | mesoderm morphogenesis | 7/336 | 77/18670 | 0.000469486 | 0.005805423 | 0.004077834 | EPHA2/FOXC1/KDM6B/KLF4/NR4A3/TRIM15/WNT5A | | 7 |
|  | BP | GO:0035743 | CD4-positive, alpha-beta T cell cytokine production | 4/336 | 21/18670 | 0.000484035 | 0.005916137 | 0.004155602 | IL18RAP/IL1B/IL1R1/IL6 | | 4 |
|  | BP | GO:0036499 | PERK-mediated unfolded protein response | 4/336 | 21/18670 | 0.000484035 | 0.005916137 | 0.004155602 | ATF3/CCL2/IGFBP1/PPP1R15A | | 4 |
|  | BP | GO:0051412 | response to corticosterone | 4/336 | 21/18670 | 0.000484035 | 0.005916137 | 0.004155602 | AVPR1A/FOS/FOSB/FOSL1 | | 4 |
|  | BP | GO:2000738 | positive regulation of stem cell differentiation | 4/336 | 21/18670 | 0.000484035 | 0.005916137 | 0.004155602 | FOXC1/GATA4/SEMA3C/TACSTD2 | | 4 |
|  | BP | GO:0035270 | endocrine system development | 9/336 | 127/18670 | 0.000490719 | 0.005980552 | 0.004200848 | ARID5B/BMP2/FOXO1/FSTL3/IL6/MSX1/ONECUT1/SALL1/WNT5A | | 9 |
|  | BP | GO:0032615 | interleukin-12 production | 6/336 | 56/18670 | 0.000494595 | 0.006009173 | 0.004220952 | IL10/IRAK3/IRF1/IRF8/THBS1/TLR2 | | 6 |
|  | BP | GO:0038061 | NIK/NF-kappaB signaling | 11/336 | 183/18670 | 0.00049591 | 0.006009173 | 0.004220952 | BCL3/BIRC3/EDAR/EDN1/IL1B/MAP3K14/MIR21/MIR223/RELB/TLR2/ZC3H12A | | 11 |
|  | BP | GO:0045923 | positive regulation of fatty acid metabolic process | 5/336 | 37/18670 | 0.000497714 | 0.006013805 | 0.004224206 | AVPR1A/IL1B/IRS2/NR4A3/PTGS2 | | 5 |
|  | BP | GO:0014743 | regulation of muscle hypertrophy | 7/336 | 78/18670 | 0.000507944 | 0.006119931 | 0.004298751 | EDN1/FOXO1/MIR21/NR4A3/PARP1/RGS2/TNFRSF1A | | 7 |
|  | BP | GO:2001236 | regulation of extrinsic apoptotic signaling pathway | 10/336 | 155/18670 | 0.000511031 | 0.006130378 | 0.004306089 | ATF3/ICAM1/IL1B/ITPRIP/MCL1/SERPINE1/THBS1/TNFAIP3/TNFRSF12A/TNFSF10 | | 10 |
|  | BP | GO:2000379 | positive regulation of reactive oxygen species metabolic process | 8/336 | 102/18670 | 0.00051171 | 0.006130378 | 0.004306089 | EDN1/FPR2/ICAM1/IL1B/KLF4/PTGS2/THBS1/ZC3H12A | | 8 |
|  | BP | GO:0050819 | negative regulation of coagulation | 6/336 | 57/18670 | 0.000544531 | 0.006505142 | 0.00456933 | CD9/EDN1/PLAU/PLAUR/SERPINE1/THBS1 | | 6 |
|  | BP | GO:0030193 | regulation of blood coagulation | 7/336 | 79/18670 | 0.00054887 | 0.006538514 | 0.004592771 | CD9/EDN1/F3/PLAU/PLAUR/SERPINE1/THBS1 | | 7 |
|  | BP | GO:0048246 | macrophage chemotaxis | 5/336 | 38/18670 | 0.000564765 | 0.006674691 | 0.004688424 | C5AR1/CCL2/CCL3/SAA1/THBS1 | | 5 |
|  | BP | GO:1900077 | negative regulation of cellular response to insulin stimulus | 5/336 | 38/18670 | 0.000564765 | 0.006674691 | 0.004688424 | CISH/IL1B/SOCS1/SOCS2/SOCS3 | | 5 |
|  | BP | GO:0030509 | BMP signaling pathway | 10/336 | 157/18670 | 0.000565101 | 0.006674691 | 0.004688424 | ACVR2B/BMP2/EGR1/FSTL3/GATA4/HIVEP1/MIR21/MSX1/NBL1/WNT5A | | 10 |
|  | BP | GO:0032869 | cellular response to insulin stimulus | 12/336 | 216/18670 | 0.000566615 | 0.006674691 | 0.004688424 | CISH/FOXO1/GCK/IGFBP1/IL1B/INHBB/IRS2/LPIN2/PARP1/SOCS1/SOCS2/SOCS3 | | 12 |
|  | BP | GO:0014812 | muscle cell migration | 8/336 | 104/18670 | 0.000582714 | 0.006813856 | 0.004786176 | ADAMTS1/HAS2/LPAR1/MIR21/MIR223/NR4A3/PLAU/SERPINE1 | | 8 |
|  | BP | GO:2001237 | negative regulation of extrinsic apoptotic signaling pathway | 8/336 | 104/18670 | 0.000582714 | 0.006813856 | 0.004786176 | ICAM1/IL1B/ITPRIP/MCL1/SERPINE1/THBS1/TNFAIP3/TNFSF10 | | 8 |
|  | BP | GO:0044321 | response to leptin | 4/336 | 22/18670 | 0.000583262 | 0.006813856 | 0.004786176 | EDN1/GCK/INHBB/NR4A3 | | 4 |
|  | BP | GO:0008406 | gonad development | 12/336 | 217/18670 | 0.000590313 | 0.006877228 | 0.004830689 | ADAMTS1/ARID5B/FOXC1/FSTL3/GATA4/GJA1/ICAM1/INHBB/SALL1/TIPARP/TNFSF10/WNT5A | | 12 |
|  | BP | GO:1900046 | regulation of hemostasis | 7/336 | 80/18670 | 0.000592376 | 0.006882307 | 0.004834258 | CD9/EDN1/F3/PLAU/PLAUR/SERPINE1/THBS1 | | 7 |
|  | BP | GO:0019229 | regulation of vasoconstriction | 6/336 | 58/18670 | 0.000598297 | 0.006913112 | 0.004855895 | AVPR1A/EDN1/GJA1/ICAM1/MIR21/PTGS2 | | 6 |
|  | BP | GO:0071385 | cellular response to glucocorticoid stimulus | 6/336 | 58/18670 | 0.000598297 | 0.006913112 | 0.004855895 | EDN1/FAM107A/FOXO1/ICAM1/STC1/ZFP36 | | 6 |
|  | BP | GO:0070997 | neuron death | 16/336 | 348/18670 | 0.000605459 | 0.006976806 | 0.004900635 | BTG2/C5AR1/CCL2/CCL3/CIT/EGR1/FOS/IL10/JUN/MCL1/NAMPT/NCOA7/NR4A2/NR4A3/PARP1/WNT5A | | 16 |
|  | BP | GO:1902042 | negative regulation of extrinsic apoptotic signaling pathway via death domain receptors | 5/336 | 39/18670 | 0.000638373 | 0.007317465 | 0.00513992 | ICAM1/ITPRIP/SERPINE1/TNFAIP3/TNFSF10 | | 5 |
|  | BP | GO:0048708 | astrocyte differentiation | 7/336 | 81/18670 | 0.000638578 | 0.007317465 | 0.00513992 | BMP2/C5AR1/FPR2/IL1B/IL6/LDLR/S100A8 | | 7 |
|  | BP | GO:0048762 | mesenchymal cell differentiation | 12/336 | 219/18670 | 0.000640213 | 0.007317465 | 0.00513992 | BMP2/EDN1/FOXC1/GLIPR2/HAS2/IL1B/IL6/MIR21/MSX1/SEMA3C/SEMA4B/WNT5A | | 12 |
|  | BP | GO:1904951 | positive regulation of establishment of protein localization | 19/336 | 456/18670 | 0.000645433 | 0.007346508 | 0.00516032 | ABLIM3/AKAP12/CCL3/CLEC4E/GCK/GJA1/IL10/IL1B/IL1RL1/IL4R/IL6/IRS2/OSM/PANX1/PTGS2/SAA1/TLR2/WNT5A/ZC3H12A | | 19 |
|  | BP | GO:0003158 | endothelium development | 9/336 | 132/18670 | 0.000649703 | 0.007346508 | 0.00516032 | ACVR2B/APOLD1/GJA1/ICAM1/IL1B/KDM6B/MIR21/STC1/TNFRSF1A | | 9 |
|  | BP | GO:0042476 | odontogenesis | 9/336 | 132/18670 | 0.000649703 | 0.007346508 | 0.00516032 | ACVR2B/ADAMTS5/BMP2/EDAR/EDN1/FOXC1/FOXO1/MSX1/SERPINE1 | | 9 |
|  | BP | GO:0045598 | regulation of fat cell differentiation | 9/336 | 132/18670 | 0.000649703 | 0.007346508 | 0.00516032 | BMP2/FOXO1/IL6/KLF5/MIR21/PTGS2/WNT5A/ZC3H12A/ZFP36 | | 9 |
|  | BP | GO:0032732 | positive regulation of interleukin-1 production | 6/336 | 59/18670 | 0.000656093 | 0.007379298 | 0.005183353 | CCL3/EGR1/MNDA/PANX1/SAA1/WNT5A | | 6 |
|  | BP | GO:0032890 | regulation of organic acid transport | 6/336 | 59/18670 | 0.000656093 | 0.007379298 | 0.005183353 | AVPR1A/EDN1/IL1B/IRS2/RGS2/THBS1 | | 6 |
|  | BP | GO:0003300 | cardiac muscle hypertrophy | 8/336 | 106/18670 | 0.000661476 | 0.007420107 | 0.005212018 | EDN1/FOXO1/GATA4/MIR21/NR4A3/PARP1/RGS2/TNFRSF1A | | 8 |
|  | BP | GO:0032637 | interleukin-8 production | 7/336 | 82/18670 | 0.000687592 | 0.007692663 | 0.005403466 | BCL3/IL10/IL1B/KLF4/SERPINE1/TLR2/WNT5A | | 7 |
|  | BP | GO:0060055 | angiogenesis involved in wound healing | 4/336 | 23/18670 | 0.000696112 | 0.00776743 | 0.005455984 | B4GALT1/MCAM/SERPINE1/TNFAIP3 | | 4 |
|  | BP | GO:0043502 | regulation of muscle adaptation | 8/336 | 107/18670 | 0.000703955 | 0.007834283 | 0.005502942 | EDN1/FOXO1/KLF4/MIR21/NR4A3/PARP1/RGS2/TNFRSF1A | | 8 |
|  | BP | GO:0002218 | activation of innate immune response | 15/336 | 319/18670 | 0.000708261 | 0.007840927 | 0.005507609 | BIRC3/CLEC4D/CLEC4E/GRAMD4/IRAK3/IRF1/MNDA/PELI1/RELB/S100A8/S100A9/TLR2/TNFAIP3/TNIP3/TRIM15 | | 15 |
|  | BP | GO:0007204 | positive regulation of cytosolic calcium ion concentration | 15/336 | 319/18670 | 0.000708261 | 0.007840927 | 0.005507609 | ANK2/AVPR1A/C5AR1/CALCA/CCL3/CCR1/CXCR1/EDN1/FPR1/FPR2/GJA1/LPAR1/PROK2/PTGER2/SAA1 | | 15 |
|  | BP | GO:0002369 | T cell cytokine production | 5/336 | 40/18670 | 0.000718937 | 0.007938338 | 0.005576033 | IL18RAP/IL1B/IL1R1/IL6/SLC11A1 | | 5 |
|  | BP | GO:0052548 | regulation of endopeptidase activity | 18/336 | 425/18670 | 0.000738363 | 0.008123412 | 0.005706032 | BIRC3/CASP4/CRYAB/CSTA/F3/GRAMD4/KLF4/MIR21/MYC/PLAUR/PTGS2/S100A8/S100A9/SERPINB9/SERPINE1/SOX7/THBS1/TNFSF10 | | 18 |
|  | BP | GO:0045582 | positive regulation of T cell differentiation | 7/336 | 83/18670 | 0.00073954 | 0.008123412 | 0.005706032 | BCL6/CD83/EGR3/IL4R/MIR21/NFKBIZ/SOCS1 | | 7 |
|  | BP | GO:0014706 | striated muscle tissue development | 17/336 | 390/18670 | 0.000750268 | 0.008203149 | 0.005762041 | ATF3/BMP2/BTG2/EDN1/EGR1/FOS/FOXC1/GATA4/GJA1/KDM6B/KLF5/MAFF/PIM1/RGS2/SEMA3C/SIK1/WNT5A | | 17 |
|  | BP | GO:0045137 | development of primary sexual characteristics | 12/336 | 223/18670 | 0.000750678 | 0.008203149 | 0.005762041 | ADAMTS1/ARID5B/FOXC1/FSTL3/GATA4/GJA1/ICAM1/INHBB/SALL1/TIPARP/TNFSF10/WNT5A | | 12 |
|  | BP | GO:0031214 | biomineral tissue development | 10/336 | 163/18670 | 0.000756443 | 0.008244843 | 0.005791327 | ACVR2B/BMP2/CCL3/CCR1/FOXO1/GJA1/GPM6B/KLF10/PTGS2/SBNO2 | | 10 |
|  | BP | GO:0007498 | mesoderm development | 9/336 | 135/18670 | 0.000763767 | 0.008303261 | 0.005832361 | ACVR2B/EPHA2/ETS2/FOXC1/KDM6B/KLF4/NR4A3/TRIM15/WNT5A | | 9 |
|  | BP | GO:0097191 | extrinsic apoptotic signaling pathway | 12/336 | 224/18670 | 0.000780656 | 0.008464325 | 0.005945495 | ATF3/BCL2A1/ICAM1/IL1B/ITPRIP/MCL1/SERPINE1/THBS1/TNFAIP3/TNFRSF12A/TNFRSF1A/TNFSF10 | | 12 |
|  | BP | GO:0050701 | interleukin-1 secretion | 6/336 | 61/18670 | 0.000784586 | 0.008464325 | 0.005945495 | CCL3/PANX1/SAA1/TNFAIP3/WNT5A/ZC3H12A | | 6 |
|  | BP | GO:0071384 | cellular response to corticosteroid stimulus | 6/336 | 61/18670 | 0.000784586 | 0.008464325 | 0.005945495 | EDN1/FAM107A/FOXO1/ICAM1/STC1/ZFP36 | | 6 |
|  | BP | GO:0043154 | negative regulation of cysteine-type endopeptidase activity involved in apoptotic process | 7/336 | 84/18670 | 0.000794544 | 0.008474051 | 0.005952327 | BIRC3/CRYAB/KLF4/PLAUR/PTGS2/SERPINB9/THBS1 | | 7 |
|  | BP | GO:0046889 | positive regulation of lipid biosynthetic process | 7/336 | 84/18670 | 0.000794544 | 0.008474051 | 0.005952327 | AVPR1A/FPR2/IL1B/LDLR/PTGS2/SIRT4/TNFRSF1A | | 7 |
|  | BP | GO:0050818 | regulation of coagulation | 7/336 | 84/18670 | 0.000794544 | 0.008474051 | 0.005952327 | CD9/EDN1/F3/PLAU/PLAUR/SERPINE1/THBS1 | | 7 |
|  | BP | GO:0051480 | regulation of cytosolic calcium ion concentration | 16/336 | 357/18670 | 0.00079509 | 0.008474051 | 0.005952327 | ANK2/AVPR1A/C5AR1/CALCA/CCL3/CCR1/CXCR1/EDN1/FPR1/FPR2/GJA1/LPAR1/PROK2/PTGER2/SAA1/WNT5A | | 16 |
|  | BP | GO:0014897 | striated muscle hypertrophy | 8/336 | 109/18670 | 0.000795507 | 0.008474051 | 0.005952327 | EDN1/FOXO1/GATA4/MIR21/NR4A3/PARP1/RGS2/TNFRSF1A | | 8 |
|  | BP | GO:0019432 | triglyceride biosynthetic process | 5/336 | 41/18670 | 0.000806861 | 0.008530541 | 0.005992006 | LDLR/LPIN2/MOGAT3/PNPLA3/SIK1 | | 5 |
|  | BP | GO:0150077 | regulation of neuroinflammatory response | 5/336 | 41/18670 | 0.000806861 | 0.008530541 | 0.005992006 | CCL3/IL1B/IL6/LDLR/PTGS2 | | 5 |
|  | BP | GO:1902893 | regulation of pri-miRNA transcription by RNA polymerase II | 5/336 | 41/18670 | 0.000806861 | 0.008530541 | 0.005992006 | BMP2/FOS/IL10/JUN/KLF5 | | 5 |
|  | BP | GO:0003012 | muscle system process | 19/336 | 465/18670 | 0.000814783 | 0.008592806 | 0.006035743 | ACTG2/ANK2/CRYAB/EDN1/FOXO1/GATA4/GJA1/IL1B/KCNE4/KLF4/MIR21/NR4A3/PARP1/PROK2/PTGS2/RGS2/STC1/TNFRSF1A/ZC3H12A | | 19 |
|  | BP | GO:0046426 | negative regulation of JAK-STAT cascade | 4/336 | 24/18670 | 0.000823578 | 0.008621065 | 0.006055592 | BCL3/SOCS1/SOCS2/SOCS3 | | 4 |
|  | BP | GO:0046639 | negative regulation of alpha-beta T cell differentiation | 4/336 | 24/18670 | 0.000823578 | 0.008621065 | 0.006055592 | BCL6/IL4R/SOCS1/ZC3H12A | | 4 |
|  | BP | GO:0046697 | decidualization | 4/336 | 24/18670 | 0.000823578 | 0.008621065 | 0.006055592 | GJA1/JUNB/PTGS2/STC1 | | 4 |
|  | BP | GO:0001666 | response to hypoxia | 16/336 | 359/18670 | 0.000843495 | 0.00880775 | 0.006186723 | APOLD1/BMP2/CRYAB/EDN1/EGLN1/EGR1/ICAM1/MIR21/MYC/NR4A2/PLAU/PTGS2/SIRT4/STC1/THBS1/TLR2 | | 16 |
|  | BP | GO:0032494 | response to peptidoglycan | 3/336 | 11/18670 | 0.000856242 | 0.008875113 | 0.00623404 | C5AR1/IL6/IRAK3 | | 3 |
|  | BP | GO:0034115 | negative regulation of heterotypic cell-cell adhesion | 3/336 | 11/18670 | 0.000856242 | 0.008875113 | 0.00623404 | IL10/IL1RN/KLF4 | | 3 |
|  | BP | GO:0070587 | regulation of cell-cell adhesion involved in gastrulation | 3/336 | 11/18670 | 0.000856242 | 0.008875113 | 0.00623404 | IL10/IL1RN/KLF4 | | 3 |
|  | BP | GO:0048608 | reproductive structure development | 18/336 | 431/18670 | 0.000866873 | 0.008963342 | 0.006296014 | ADAMTS1/ARID5B/FOSL1/FOXC1/FSTL3/GATA4/GJA1/ICAM1/IL10/INHBB/JUNB/PTGS2/SALL1/SOCS3/STC1/TIPARP/TNFSF10/WNT5A | | 18 |
|  | BP | GO:0003007 | heart morphogenesis | 13/336 | 259/18670 | 0.000875683 | 0.009032345 | 0.006344483 | ADAMTS1/ARL13B/BMP2/FOXC1/GATA4/GJA1/HAS2/JUN/MIR21/MSX1/PIM1/SEMA3C/WNT5A | | 13 |
|  | BP | GO:0002460 | adaptive immune response based on somatic recombination of immune receptors built from immunoglobulin superfamily domains | 16/336 | 361/18670 | 0.000894386 | 0.009178793 | 0.006447351 | BCL3/BCL6/ICAM1/IL10/IL18RAP/IL1B/IL1R1/IL1RL1/IL4R/IL6/MIR21/NFKBIZ/RELB/SLC11A1/TNFAIP3/ZC3H12A | | 16 |
|  | BP | GO:0014896 | muscle hypertrophy | 8/336 | 111/18670 | 0.000896392 | 0.009178793 | 0.006447351 | EDN1/FOXO1/GATA4/MIR21/NR4A3/PARP1/RGS2/TNFRSF1A | | 8 |
|  | BP | GO:1904019 | epithelial cell apoptotic process | 8/336 | 111/18670 | 0.000896392 | 0.009178793 | 0.006447351 | CCL2/ICAM1/IL10/IL6/SERPINE1/THBS1/TNFAIP3/ZFP36 | | 8 |
|  | BP | GO:0072001 | renal system development | 14/336 | 293/18670 | 0.000906472 | 0.009259587 | 0.006504102 | ACVR2B/ADAMTS1/ARID5B/BMP2/EGR1/FOXC1/FSTL3/HAS2/MYC/SALL1/SDC4/TACSTD2/TIPARP/WNT5A | | 14 |
|  | BP | GO:0061458 | reproductive system development | 18/336 | 434/18670 | 0.000938012 | 0.009558684 | 0.006714193 | ADAMTS1/ARID5B/FOSL1/FOXC1/FSTL3/GATA4/GJA1/ICAM1/IL10/INHBB/JUNB/PTGS2/SALL1/SOCS3/STC1/TIPARP/TNFSF10/WNT5A | | 18 |
|  | BP | GO:0002700 | regulation of production of molecular mediator of immune response | 9/336 | 139/18670 | 0.000940723 | 0.009563258 | 0.006717406 | BCL6/IL10/IL1B/IL1R1/IL4R/IL6/IRAK3/NR4A3/WNT5A | | 9 |
|  | BP | GO:0090025 | regulation of monocyte chemotaxis | 4/336 | 25/18670 | 0.00096666 | 0.009803365 | 0.006886061 | CCR1/FPR2/NBL1/SERPINE1 | | 4 |
|  | BP | GO:0001837 | epithelial to mesenchymal transition | 9/336 | 140/18670 | 0.000989794 | 0.010013972 | 0.007033995 | BMP2/FOXC1/GLIPR2/HAS2/IL1B/IL6/MIR21/MSX1/WNT5A | | 9 |
|  | BP | GO:0045619 | regulation of lymphocyte differentiation | 10/336 | 169/18670 | 0.000998229 | 0.010014797 | 0.007034575 | BCL6/CD83/DTX1/EGR3/IL4R/IRF1/MIR21/NFKBIZ/SOCS1/ZC3H12A | | 10 |
|  | BP | GO:0006692 | prostanoid metabolic process | 5/336 | 43/18670 | 0.001006453 | 0.010014797 | 0.007034575 | AVPR1A/EDN1/IL1B/PTGS2/TNFRSF1A | | 5 |
|  | BP | GO:0006693 | prostaglandin metabolic process | 5/336 | 43/18670 | 0.001006453 | 0.010014797 | 0.007034575 | AVPR1A/EDN1/IL1B/PTGS2/TNFRSF1A | | 5 |
|  | BP | GO:0045429 | positive regulation of nitric oxide biosynthetic process | 5/336 | 43/18670 | 0.001006453 | 0.010014797 | 0.007034575 | EDN1/ICAM1/IL1B/KLF4/PTGS2 | | 5 |
|  | BP | GO:0046460 | neutral lipid biosynthetic process | 5/336 | 43/18670 | 0.001006453 | 0.010014797 | 0.007034575 | LDLR/LPIN2/MOGAT3/PNPLA3/SIK1 | | 5 |
|  | BP | GO:0046463 | acylglycerol biosynthetic process | 5/336 | 43/18670 | 0.001006453 | 0.010014797 | 0.007034575 | LDLR/LPIN2/MOGAT3/PNPLA3/SIK1 | | 5 |
|  | BP | GO:1904646 | cellular response to amyloid-beta | 5/336 | 43/18670 | 0.001006453 | 0.010014797 | 0.007034575 | CASP4/FPR2/ICAM1/NAMPT/PARP1 | | 5 |
|  | BP | GO:0045600 | positive regulation of fat cell differentiation | 6/336 | 64/18670 | 0.001012756 | 0.010030319 | 0.007045478 | BMP2/KLF5/MIR21/PTGS2/ZC3H12A/ZFP36 | | 6 |
|  | BP | GO:0048247 | lymphocyte chemotaxis | 6/336 | 64/18670 | 0.001012756 | 0.010030319 | 0.007045478 | CCL2/CCL3/CCL8/CH25H/SAA1/WNT5A | | 6 |
|  | BP | GO:0071772 | response to BMP | 10/336 | 170/18670 | 0.001044085 | 0.010279954 | 0.007220826 | ACVR2B/BMP2/EGR1/FSTL3/GATA4/HIVEP1/MIR21/MSX1/NBL1/WNT5A | | 10 |
|  | BP | GO:0071773 | cellular response to BMP stimulus | 10/336 | 170/18670 | 0.001044085 | 0.010279954 | 0.007220826 | ACVR2B/BMP2/EGR1/FSTL3/GATA4/HIVEP1/MIR21/MSX1/NBL1/WNT5A | | 10 |
|  | BP | GO:1900407 | regulation of cellular response to oxidative stress | 7/336 | 88/18670 | 0.001047685 | 0.010279954 | 0.007220826 | GATA4/IL10/MCL1/MIR21/NCOA7/NR4A3/PARP1 | | 7 |
|  | BP | GO:1904035 | regulation of epithelial cell apoptotic process | 7/336 | 88/18670 | 0.001047685 | 0.010279954 | 0.007220826 | CCL2/ICAM1/IL6/SERPINE1/THBS1/TNFAIP3/ZFP36 | | 7 |
|  | BP | GO:0002758 | innate immune response-activating signal transduction | 14/336 | 298/18670 | 0.001066099 | 0.010394259 | 0.007301116 | BIRC3/CLEC4D/CLEC4E/GRAMD4/IRAK3/IRF1/PELI1/RELB/S100A8/S100A9/TLR2/TNFAIP3/TNIP3/TRIM15 | | 14 |
|  | BP | GO:0006690 | icosanoid metabolic process | 8/336 | 114/18670 | 0.001066708 | 0.010394259 | 0.007301116 | ALOX5AP/AVPR1A/EDN1/FADS1/IL1B/PTGS2/TLR2/TNFRSF1A | | 8 |
|  | BP | GO:0045446 | endothelial cell differentiation | 8/336 | 114/18670 | 0.001066708 | 0.010394259 | 0.007301116 | ACVR2B/APOLD1/ICAM1/IL1B/KDM6B/MIR21/STC1/TNFRSF1A | | 8 |
|  | BP | GO:0043901 | negative regulation of multi-organism process | 10/336 | 171/18670 | 0.00109165 | 0.010612845 | 0.007454655 | APOBEC3A/CCL3/CCL8/ISG20/JUN/PLSCR1/TNFAIP3/TRIM15/ZC3H12A/ZFP36 | | 10 |
|  | BP | GO:0032729 | positive regulation of interferon-gamma production | 6/336 | 65/18670 | 0.001099137 | 0.010636726 | 0.007471429 | BCL3/IL1B/IL1R1/IRF8/SLC11A1/WNT5A | | 6 |
|  | BP | GO:0072577 | endothelial cell apoptotic process | 6/336 | 65/18670 | 0.001099137 | 0.010636726 | 0.007471429 | CCL2/ICAM1/IL10/SERPINE1/THBS1/TNFAIP3 | | 6 |
|  | BP | GO:0010463 | mesenchymal cell proliferation | 5/336 | 44/18670 | 0.001118966 | 0.010681426 | 0.007502828 | BMP2/IRS2/MSX1/MYC/WNT5A | | 5 |
|  | BP | GO:1904407 | positive regulation of nitric oxide metabolic process | 5/336 | 44/18670 | 0.001118966 | 0.010681426 | 0.007502828 | EDN1/ICAM1/IL1B/KLF4/PTGS2 | | 5 |
|  | BP | GO:0051251 | positive regulation of lymphocyte activation | 15/336 | 334/18670 | 0.001125891 | 0.010681426 | 0.007502828 | BCL6/CCL2/CD83/EGR3/IGFBP2/IL1B/IL4R/IL6/IRS2/MAP3K8/MIR21/NFKBIZ/PELI1/SIRPA/SOCS1 | | 15 |
|  | BP | GO:0034114 | regulation of heterotypic cell-cell adhesion | 4/336 | 26/18670 | 0.001126357 | 0.010681426 | 0.007502828 | IL10/IL1B/IL1RN/KLF4 | | 4 |
|  | BP | GO:0072539 | T-helper 17 cell differentiation | 4/336 | 26/18670 | 0.001126357 | 0.010681426 | 0.007502828 | IL6/MIR21/NFKBIZ/ZC3H12A | | 4 |
|  | BP | GO:0031392 | regulation of prostaglandin biosynthetic process | 3/336 | 12/18670 | 0.001126488 | 0.010681426 | 0.007502828 | AVPR1A/IL1B/PTGS2 | | 3 |
|  | BP | GO:0045080 | positive regulation of chemokine biosynthetic process | 3/336 | 12/18670 | 0.001126488 | 0.010681426 | 0.007502828 | EGR1/IL1B/WNT5A | | 3 |
|  | BP | GO:0070586 | cell-cell adhesion involved in gastrulation | 3/336 | 12/18670 | 0.001126488 | 0.010681426 | 0.007502828 | IL10/IL1RN/KLF4 | | 3 |
|  | BP | GO:1900376 | regulation of secondary metabolite biosynthetic process | 3/336 | 12/18670 | 0.001126488 | 0.010681426 | 0.007502828 | ASIP/MIR21/WNT5A | | 3 |
|  | BP | GO:0071453 | cellular response to oxygen levels | 12/336 | 234/18670 | 0.001139499 | 0.01077048 | 0.007565381 | EDN1/EGLN1/EGR1/FOXO1/ICAM1/LPAR1/MIR21/MYC/NAMPT/PTGS2/SIRT4/STC1 | | 12 |
|  | BP | GO:0032874 | positive regulation of stress-activated MAPK cascade | 10/336 | 172/18670 | 0.001140973 | 0.01077048 | 0.007565381 | BMP2/EDAR/EDN1/ERN1/GADD45B/GADD45G/IL1B/IL1RN/WNT5A/ZC3H12A | | 10 |
|  | BP | GO:0006469 | negative regulation of protein kinase activity | 12/336 | 235/18670 | 0.001181896 | 0.011131934 | 0.007819272 | DUSP16/DUSP6/GADD45B/HEXIM2/IL1B/IRAK3/ITPRIP/RGS2/SOCS1/SOCS3/SPRY4/TNFAIP3 | | 12 |
|  | BP | GO:0001659 | temperature homeostasis | 10/336 | 173/18670 | 0.001192102 | 0.011140611 | 0.007825367 | ACVR2B/ADAMTS5/EGR1/FOXO1/GADD45G/GJA1/IL1B/IL4R/KDM6B/PTGS2 | | 10 |
|  | BP | GO:0070304 | positive regulation of stress-activated protein kinase signaling cascade | 10/336 | 173/18670 | 0.001192102 | 0.011140611 | 0.007825367 | BMP2/EDAR/EDN1/ERN1/GADD45B/GADD45G/IL1B/IL1RN/WNT5A/ZC3H12A | | 10 |
|  | BP | GO:0042475 | odontogenesis of dentin-containing tooth | 7/336 | 90/18670 | 0.001195989 | 0.011140611 | 0.007825367 | ACVR2B/BMP2/EDAR/FOXC1/FOXO1/MSX1/SERPINE1 | | 7 |
|  | BP | GO:0045778 | positive regulation of ossification | 7/336 | 90/18670 | 0.001195989 | 0.011140611 | 0.007825367 | ACVR2B/BMP2/CEBPD/GPM6B/IL6/MIR21/WNT5A | | 7 |
|  | BP | GO:1903035 | negative regulation of response to wounding | 7/336 | 90/18670 | 0.001195989 | 0.011140611 | 0.007825367 | CD9/EDN1/GJA1/PLAU/PLAUR/SERPINE1/THBS1 | | 7 |
|  | BP | GO:0015711 | organic anion transport | 19/336 | 482/18670 | 0.001240419 | 0.011529084 | 0.008098238 | AVPR1A/CA13/CA4/EDN1/GJA1/IL1B/IRS2/LDLR/PITPNM2/RGS2/SLC11A1/SLC16A13/SLC16A3/SLC1A3/SLC25A32/SLC7A1/SLCO4A1/THBS1/TNFAIP8L3 | | 19 |
|  | BP | GO:0002761 | regulation of myeloid leukocyte differentiation | 8/336 | 117/18670 | 0.001261948 | 0.011677854 | 0.008202736 | CCL3/CCR1/FOS/FSTL3/JUN/KLF10/MIR223/MYC | | 8 |
|  | BP | GO:1901222 | regulation of NIK/NF-kappaB signaling | 8/336 | 117/18670 | 0.001261948 | 0.011677854 | 0.008202736 | BCL3/EDAR/EDN1/IL1B/MIR21/MIR223/TLR2/ZC3H12A | | 8 |
|  | BP | GO:2000191 | regulation of fatty acid transport | 4/336 | 27/18670 | 0.00130367 | 0.012037603 | 0.008455431 | EDN1/IL1B/IRS2/THBS1 | | 4 |
|  | BP | GO:0048871 | multicellular organismal homeostasis | 19/336 | 485/18670 | 0.001332475 | 0.012276764 | 0.008623422 | ACVR2B/ADAMTS5/CALCA/EGR1/FOXC1/FOXO1/GADD45G/GJA1/GRHL1/HAS2/IL1B/IL4R/IL6/KDM6B/MAK/NR4A3/PTGS2/SLC11A1/TNFAIP3 | | 19 |
|  | BP | GO:2000117 | negative regulation of cysteine-type endopeptidase activity | 7/336 | 92/18670 | 0.001360258 | 0.012505504 | 0.008784093 | BIRC3/CRYAB/KLF4/PLAUR/PTGS2/SERPINB9/THBS1 | | 7 |
|  | BP | GO:0046651 | lymphocyte proliferation | 13/336 | 272/18670 | 0.001365332 | 0.012524922 | 0.008797732 | BCL6/GJA1/IGFBP2/IL10/IL1B/IL6/IRF1/IRS2/MIR21/MNDA/PELI1/SDC4/SLC11A1 | | 13 |
|  | BP | GO:0032480 | negative regulation of type I interferon production | 5/336 | 46/18670 | 0.001371584 | 0.012527927 | 0.008799843 | IL10/MIR21/RELB/SIRPA/TNFAIP3 | | 5 |
|  | BP | GO:0050706 | regulation of interleukin-1 beta secretion | 5/336 | 46/18670 | 0.001371584 | 0.012527927 | 0.008799843 | CCL3/PANX1/TNFAIP3/WNT5A/ZC3H12A | | 5 |
|  | BP | GO:0035914 | skeletal muscle cell differentiation | 6/336 | 68/18670 | 0.00139244 | 0.012663714 | 0.008895223 | ATF3/BTG2/EGR1/FOS/KLF5/MAFF | | 6 |
|  | BP | GO:0050918 | positive chemotaxis | 6/336 | 68/18670 | 0.00139244 | 0.012663714 | 0.008895223 | CCL3/F3/FPR2/SAA1/SAA2/WNT5A | | 6 |
|  | BP | GO:0001952 | regulation of cell-matrix adhesion | 8/336 | 119/18670 | 0.001407196 | 0.0127591 | 0.008962223 | BCL6/FAM107A/GPM6B/ONECUT1/PLAU/SDC4/SERPINE1/THBS1 | | 8 |
|  | BP | GO:0043542 | endothelial cell migration | 13/336 | 273/18670 | 0.001410927 | 0.0127591 | 0.008962223 | ANXA3/EDN1/EGR3/EPHA2/KLF4/MIR21/NR4A1/PTGS2/S100P/STC1/THBS1/WNT5A/ZC3H12A | | 13 |
|  | BP | GO:0060491 | regulation of cell projection assembly | 10/336 | 177/18670 | 0.001415676 | 0.0127591 | 0.008962223 | CDC42EP1/DPYSL3/EPHA2/FAM110C/FRMD7/ICAM1/KLF5/MAK/RAP1GAP/TACSTD2 | | 10 |
|  | BP | GO:0007623 | circadian rhythm | 11/336 | 208/18670 | 0.001418013 | 0.0127591 | 0.008962223 | EGR1/EGR3/JUN/KLF10/NAMPT/NFIL3/PROK2/RELB/SERPINE1/SIK1/TYMS | | 11 |
|  | BP | GO:0050670 | regulation of lymphocyte proliferation | 11/336 | 208/18670 | 0.001418013 | 0.0127591 | 0.008962223 | BCL6/IGFBP2/IL10/IL1B/IL6/IRF1/IRS2/MIR21/MNDA/PELI1/SDC4 | | 11 |
|  | BP | GO:0002430 | complement receptor mediated signaling pathway | 3/336 | 13/18670 | 0.001444995 | 0.012892159 | 0.009055686 | C5AR1/FPR1/FPR2 | | 3 |
|  | BP | GO:0045779 | negative regulation of bone resorption | 3/336 | 13/18670 | 0.001444995 | 0.012892159 | 0.009055686 | CALCA/IL6/TNFAIP3 | | 3 |
|  | BP | GO:0061042 | vascular wound healing | 3/336 | 13/18670 | 0.001444995 | 0.012892159 | 0.009055686 | MCAM/SERPINE1/TNFAIP3 | | 3 |
|  | BP | GO:1903960 | negative regulation of anion transmembrane transport | 3/336 | 13/18670 | 0.001444995 | 0.012892159 | 0.009055686 | IRS2/RGS2/THBS1 | | 3 |
|  | BP | GO:0036473 | cell death in response to oxidative stress | 7/336 | 93/18670 | 0.001448739 | 0.012898353 | 0.009060037 | GATA4/IL10/MCL1/MIR21/NCOA7/NR4A3/PARP1 | | 7 |
|  | BP | GO:0032943 | mononuclear cell proliferation | 13/336 | 274/18670 | 0.001457781 | 0.012951591 | 0.009097432 | BCL6/GJA1/IGFBP2/IL10/IL1B/IL6/IRF1/IRS2/MIR21/MNDA/PELI1/SDC4/SLC11A1 | | 13 |
|  | BP | GO:0051592 | response to calcium ion | 9/336 | 148/18670 | 0.001461814 | 0.012960196 | 0.009103477 | ALOX5AP/EDN1/FOS/FOSB/JUN/JUNB/SYT12/THBS1/WNT5A | | 9 |
|  | BP | GO:0032944 | regulation of mononuclear cell proliferation | 11/336 | 209/18670 | 0.001473343 | 0.013035078 | 0.009156075 | BCL6/IGFBP2/IL10/IL1B/IL6/IRF1/IRS2/MIR21/MNDA/PELI1/SDC4 | | 11 |
|  | BP | GO:0052547 | regulation of peptidase activity | 18/336 | 452/18670 | 0.001478474 | 0.013053169 | 0.009168783 | BIRC3/CASP4/CRYAB/CSTA/F3/GRAMD4/KLF4/MIR21/MYC/PLAUR/PTGS2/S100A8/S100A9/SERPINB9/SERPINE1/SOX7/THBS1/TNFSF10 | | 18 |
|  | BP | GO:0001516 | prostaglandin biosynthetic process | 4/336 | 28/18670 | 0.001499595 | 0.013129998 | 0.009222748 | AVPR1A/EDN1/IL1B/PTGS2 | | 4 |
|  | BP | GO:0001702 | gastrulation with mouth forming second | 4/336 | 28/18670 | 0.001499595 | 0.013129998 | 0.009222748 | ACVR2B/ETS2/NAT8B/WNT5A | | 4 |
|  | BP | GO:0042730 | fibrinolysis | 4/336 | 28/18670 | 0.001499595 | 0.013129998 | 0.009222748 | PLAU/PLAUR/SERPINE1/THBS1 | | 4 |
|  | BP | GO:0046457 | prostanoid biosynthetic process | 4/336 | 28/18670 | 0.001499595 | 0.013129998 | 0.009222748 | AVPR1A/EDN1/IL1B/PTGS2 | | 4 |
|  | BP | GO:0014911 | positive regulation of smooth muscle cell migration | 5/336 | 47/18670 | 0.001512568 | 0.013161834 | 0.009245111 | ADAMTS1/HAS2/LPAR1/MIR21/NR4A3 | | 5 |
|  | BP | GO:0046638 | positive regulation of alpha-beta T cell differentiation | 5/336 | 47/18670 | 0.001512568 | 0.013161834 | 0.009245111 | CD83/IL4R/MIR21/NFKBIZ/SOCS1 | | 5 |
|  | BP | GO:1900744 | regulation of p38MAPK cascade | 5/336 | 47/18670 | 0.001512568 | 0.013161834 | 0.009245111 | BMP2/GADD45B/GADD45G/IL1B/ZC3H12A | | 5 |
|  | BP | GO:2001235 | positive regulation of apoptotic signaling pathway | 10/336 | 179/18670 | 0.001539615 | 0.013332568 | 0.009365037 | ATF3/INHBB/MCL1/MSX1/PLAUR/S100A8/S100A9/THBS1/TNFRSF12A/TNFSF10 | | 10 |
|  | BP | GO:0045621 | positive regulation of lymphocyte differentiation | 7/336 | 94/18670 | 0.001541647 | 0.013332568 | 0.009365037 | BCL6/CD83/EGR3/IL4R/MIR21/NFKBIZ/SOCS1 | | 7 |
|  | BP | GO:1901216 | positive regulation of neuron death | 7/336 | 94/18670 | 0.001541647 | 0.013332568 | 0.009365037 | CCL3/EGR1/FOS/JUN/MCL1/PARP1/WNT5A | | 7 |
|  | BP | GO:1903531 | negative regulation of secretion by cell | 11/336 | 211/18670 | 0.001589317 | 0.013716777 | 0.009634913 | EDN1/IL10/IL1B/INHBB/MIDN/OSM/PIM3/SIRT4/TNFAIP3/TNFRSF1A/ZC3H12A | | 11 |
|  | BP | GO:0050777 | negative regulation of immune response | 9/336 | 150/18670 | 0.001604354 | 0.013818355 | 0.009706263 | BCL6/IL10/IL1RL1/IL4R/IRAK3/MIR21/SERPINB9/TNFAIP3/ZC3H12A | | 9 |
|  | BP | GO:0032720 | negative regulation of tumor necrosis factor production | 6/336 | 70/18670 | 0.001618804 | 0.013858138 | 0.009734208 | BCL3/IL10/IRAK3/SIRPA/TNFAIP3/ZC3H12A | | 6 |
|  | BP | GO:0050766 | positive regulation of phagocytosis | 6/336 | 70/18670 | 0.001618804 | 0.013858138 | 0.009734208 | CCL2/FPR2/IL1B/IL2RB/SIRPA/SLC11A1 | | 6 |
|  | BP | GO:0060395 | SMAD protein signal transduction | 6/336 | 70/18670 | 0.001618804 | 0.013858138 | 0.009734208 | BMP2/FOS/INHBB/INHBE/JUN/PARP1 | | 6 |
|  | BP | GO:0051896 | regulation of protein kinase B signaling | 12/336 | 244/18670 | 0.001625734 | 0.013889351 | 0.009756132 | CCL3/EPHA2/F3/FAM110C/GATA4/HBEGF/IRS2/KLF4/MIR21/OSM/THBS1/TNFAIP8L3 | | 12 |
|  | BP | GO:0044070 | regulation of anion transport | 7/336 | 95/18670 | 0.001639132 | 0.013975583 | 0.009816703 | AVPR1A/EDN1/IL1B/IRS2/RGS2/STC1/THBS1 | | 7 |
|  | BP | GO:0001822 | kidney development | 13/336 | 278/18670 | 0.001658326 | 0.014110786 | 0.009911672 | ACVR2B/ADAMTS1/ARID5B/BMP2/EGR1/FOXC1/FSTL3/HAS2/MYC/SALL1/SDC4/TACSTD2/TIPARP | | 13 |
|  | BP | GO:0045933 | positive regulation of muscle contraction | 5/336 | 48/18670 | 0.001663928 | 0.014130024 | 0.009925185 | EDN1/MIR21/PROK2/PTGS2/RGS2 | | 5 |
|  | BP | GO:1901214 | regulation of neuron death | 14/336 | 313/18670 | 0.001693224 | 0.014349991 | 0.010079694 | BTG2/C5AR1/CCL2/CCL3/EGR1/FOS/IL10/JUN/MCL1/NCOA7/NR4A2/NR4A3/PARP1/WNT5A | | 14 |
|  | BP | GO:0002697 | regulation of immune effector process | 18/336 | 458/18670 | 0.001709197 | 0.014427529 | 0.010134158 | BCL6/BIRC3/C5AR1/IL10/IL18RAP/IL1B/IL1R1/IL4R/IL6/IRAK3/MIR21/NFKBIZ/NR4A3/SERPINB9/TNFAIP3/TRIM15/WNT5A/ZC3H12A | | 18 |
|  | BP | GO:0006874 | cellular calcium ion homeostasis | 18/336 | 458/18670 | 0.001709197 | 0.014427529 | 0.010134158 | ANK2/AVPR1A/C5AR1/CALCA/CCL3/CCL8/CCR1/CXCR1/EDN1/FPR1/FPR2/GJA1/LPAR1/PROK2/PTGER2/SAA1/STC1/WNT5A | | 18 |
|  | BP | GO:0072538 | T-helper 17 type immune response | 4/336 | 29/18670 | 0.001715123 | 0.014448718 | 0.010149042 | IL6/MIR21/NFKBIZ/ZC3H12A | | 4 |
|  | BP | GO:1990823 | response to leukemia inhibitory factor | 7/336 | 96/18670 | 0.001741346 | 0.014587218 | 0.010246327 | ARID5B/DTX1/ICAM1/KLF4/KLF5/SBNO2/SOCS3 | | 7 |
|  | BP | GO:1990830 | cellular response to leukemia inhibitory factor | 7/336 | 96/18670 | 0.001741346 | 0.014587218 | 0.010246327 | ARID5B/DTX1/ICAM1/KLF4/KLF5/SBNO2/SOCS3 | | 7 |
|  | BP | GO:0061138 | morphogenesis of a branching epithelium | 10/336 | 182/18670 | 0.001741912 | 0.014587218 | 0.010246327 | BMP2/EDN1/EPHA2/IL10/MYC/SALL1/SEMA3C/SOCS3/TACSTD2/WNT5A | | 10 |
|  | BP | GO:0062012 | regulation of small molecule metabolic process | 18/336 | 459/18670 | 0.001750464 | 0.014629866 | 0.010276283 | AVPR1A/BMP2/EGR1/FOXO1/GCK/HAS2/IL1B/IRS2/LDLR/ME1/MIDN/MIR21/NR4A3/PARP1/PFKFB3/PTGS2/SIK1/SIRT4 | | 18 |
|  | BP | GO:0009896 | positive regulation of catabolic process | 17/336 | 423/18670 | 0.001802506 | 0.01501828 | 0.010549112 | BTG2/FOXO1/GJA1/IL1B/IL6/IRS2/LDLR/PFKFB3/PIM2/RNF152/RNF180/SUPV3L1/TIPARP/TNFAIP3/WNT5A/ZC3H12A/ZFP36 | | 17 |
|  | BP | GO:0034356 | NAD biosynthesis via nicotinamide riboside salvage pathway | 3/336 | 14/18670 | 0.001814694 | 0.01501828 | 0.010549112 | NAMPT/NNMT/PTGS2 | | 3 |
|  | BP | GO:0043455 | regulation of secondary metabolic process | 3/336 | 14/18670 | 0.001814694 | 0.01501828 | 0.010549112 | ASIP/MIR21/WNT5A | | 3 |
|  | BP | GO:0045064 | T-helper 2 cell differentiation | 3/336 | 14/18670 | 0.001814694 | 0.01501828 | 0.010549112 | BCL3/BCL6/IL4R | | 3 |
|  | BP | GO:2001279 | regulation of unsaturated fatty acid biosynthetic process | 3/336 | 14/18670 | 0.001814694 | 0.01501828 | 0.010549112 | AVPR1A/IL1B/PTGS2 | | 3 |
|  | BP | GO:0051017 | actin filament bundle assembly | 9/336 | 153/18670 | 0.001838871 | 0.015159034 | 0.010647981 | DPYSL3/FAM107A/FRMD7/LPAR1/MIR21/RND1/SDC4/SHROOM1/TACSTD2 | | 9 |
|  | BP | GO:0062013 | positive regulation of small molecule metabolic process | 9/336 | 153/18670 | 0.001838871 | 0.015159034 | 0.010647981 | AVPR1A/FOXO1/GCK/HAS2/IL1B/IRS2/NR4A3/PFKFB3/PTGS2 | | 9 |
|  | BP | GO:1902882 | regulation of response to oxidative stress | 7/336 | 97/18670 | 0.001848443 | 0.015208297 | 0.010682584 | GATA4/IL10/MCL1/MIR21/NCOA7/NR4A3/PARP1 | | 7 |
|  | BP | GO:1903556 | negative regulation of tumor necrosis factor superfamily cytokine production | 6/336 | 72/18670 | 0.001872096 | 0.015372995 | 0.010798271 | BCL3/IL10/IRAK3/SIRPA/TNFAIP3/ZC3H12A | | 6 |
|  | BP | GO:0032970 | regulation of actin filament-based process | 16/336 | 388/18670 | 0.001880927 | 0.015415581 | 0.010828184 | ANK2/CDC42EP1/CIT/EDN1/FAM107A/FRMD6/FRMD7/GATA4/GPM6B/ICAM1/LPAR1/MIR21/RND1/SDC4/STC1/TACSTD2 | | 16 |
|  | BP | GO:0042098 | T cell proliferation | 10/336 | 184/18670 | 0.001888348 | 0.015446467 | 0.010849879 | GJA1/IGFBP2/IL10/IL1B/IL6/IRF1/MIR21/PELI1/SDC4/SLC11A1 | | 10 |
|  | BP | GO:0038111 | interleukin-7-mediated signaling pathway | 4/336 | 30/18670 | 0.001951238 | 0.015838363 | 0.011125153 | CISH/IRS2/SOCS1/SOCS2 | | 4 |
|  | BP | GO:0050869 | negative regulation of B cell activation | 4/336 | 30/18670 | 0.001951238 | 0.015838363 | 0.011125153 | BCL6/IL10/MNDA/TNFAIP3 | | 4 |
|  | BP | GO:0061082 | myeloid leukocyte cytokine production | 4/336 | 30/18670 | 0.001951238 | 0.015838363 | 0.011125153 | BCL6/IRAK3/NR4A3/WNT5A | | 4 |
|  | BP | GO:1904893 | negative regulation of STAT cascade | 4/336 | 30/18670 | 0.001951238 | 0.015838363 | 0.011125153 | BCL3/SOCS1/SOCS2/SOCS3 | | 4 |
|  | BP | GO:0050764 | regulation of phagocytosis | 7/336 | 98/18670 | 0.001960579 | 0.015883694 | 0.011156995 | CCL2/FPR2/IL1B/IL2RB/SIRPA/SLC11A1/TLR2 | | 7 |
|  | BP | GO:0030072 | peptide hormone secretion | 12/336 | 250/18670 | 0.001991762 | 0.01610547 | 0.011312774 | ACVR2B/EDN1/GCK/GJA1/IL1B/IL1RN/IL6/INHBB/IRS2/MIDN/PIM3/SIRT4 | | 12 |
|  | BP | GO:0031100 | animal organ regeneration | 6/336 | 73/18670 | 0.002009462 | 0.016217582 | 0.011391524 | ANXA3/C5AR1/IL10/NNMT/NR4A3/TYMS | | 6 |
|  | BP | GO:0032479 | regulation of type I interferon production | 8/336 | 126/18670 | 0.00202307 | 0.01629631 | 0.011446824 | IL10/IRF1/MIR21/RELB/SIRPA/TLR2/TNFAIP3/TRIM15 | | 8 |
|  | BP | GO:0010001 | glial cell differentiation | 11/336 | 218/18670 | 0.002055516 | 0.016490451 | 0.011583192 | BMP2/C5AR1/CD9/DTX1/FPR2/IL1B/IL6/LDLR/LPAR1/S100A8/TLR2 | | 11 |
|  | BP | GO:0021537 | telencephalon development | 12/336 | 251/18670 | 0.002058869 | 0.016490451 | 0.011583192 | ALDH1A3/ANXA3/ARL13B/AVPR1A/BCL2A1/BMP2/BTG2/KDM6B/LPAR1/NR4A3/SALL1/WNT5A | | 12 |
|  | BP | GO:0045637 | regulation of myeloid cell differentiation | 12/336 | 251/18670 | 0.002058869 | 0.016490451 | 0.011583192 | CCL3/CCR1/FOS/FSTL3/JUN/KLF10/MIR223/MYC/NFE2/NR4A3/THBS1/ZFP36 | | 12 |
|  | BP | GO:0007259 | JAK-STAT cascade | 9/336 | 156/18670 | 0.002100041 | 0.016756743 | 0.01177024 | BCL3/CCL2/IL10/IL6/OSM/SOCS1/SOCS2/SOCS3/TNFRSF1A | | 9 |
|  | BP | GO:0030856 | regulation of epithelial cell differentiation | 9/336 | 156/18670 | 0.002100041 | 0.016756743 | 0.01177024 | APOLD1/FOXC1/GRHL1/IL1B/MAFF/MIR21/SERPINE1/TNFRSF1A/ZFP36 | | 9 |
|  | BP | GO:0007584 | response to nutrient | 11/336 | 219/18670 | 0.002130364 | 0.016966685 | 0.011917707 | DHODH/GATA4/IGFBP2/IL1B/OGG1/P2RY11/PANX1/PIM1/PTGS2/STC1/TYMS | | 11 |
|  | BP | GO:0070373 | negative regulation of ERK1 and ERK2 cascade | 6/336 | 74/18670 | 0.002154311 | 0.017125153 | 0.012029018 | ATF3/DUSP6/KLF4/MIR21/SIRPA/SPRY4 | | 6 |
|  | BP | GO:0007566 | embryo implantation | 5/336 | 51/18670 | 0.002184769 | 0.017298045 | 0.012150461 | CALCA/IL1B/MIR21/PTGS2/STC1 | | 5 |
|  | BP | GO:0097366 | response to bronchodilator | 5/336 | 51/18670 | 0.002184769 | 0.017298045 | 0.012150461 | EGLN1/FOXO1/ICAM1/NR4A2/RGS2 | | 5 |
|  | BP | GO:0043271 | negative regulation of ion transport | 9/336 | 157/18670 | 0.002193382 | 0.017298045 | 0.012150461 | GEM/GPM6B/ICAM1/IRS2/KCNE4/PTGS2/RGS2/STC1/THBS1 | | 9 |
|  | BP | GO:0061572 | actin filament bundle organization | 9/336 | 157/18670 | 0.002193382 | 0.017298045 | 0.012150461 | DPYSL3/FAM107A/FRMD7/LPAR1/MIR21/RND1/SDC4/SHROOM1/TACSTD2 | | 9 |
|  | BP | GO:0009914 | hormone transport | 14/336 | 322/18670 | 0.002199078 | 0.017298045 | 0.012150461 | ACVR2B/EDN1/GCK/GJA1/IL1B/IL1RN/IL6/INHBB/IRS2/MIDN/OSM/PIM3/SIRT4/SLCO4A1 | | 14 |
|  | BP | GO:0007200 | phospholipase C-activating G protein-coupled receptor signaling pathway | 7/336 | 100/18670 | 0.002200603 | 0.017298045 | 0.012150461 | C5AR1/CALCA/EDN1/FPR1/FPR2/LPAR1/P2RY11 | | 7 |
|  | BP | GO:0033198 | response to ATP | 4/336 | 31/18670 | 0.002208916 | 0.017331177 | 0.012173733 | IL1B/P2RY11/PANX1/PTGS2 | | 4 |
|  | BP | GO:0032606 | type I interferon production | 8/336 | 128/18670 | 0.002233181 | 0.017400398 | 0.012222355 | IL10/IRF1/MIR21/RELB/SIRPA/TLR2/TNFAIP3/TRIM15 | | 8 |
|  | BP | GO:0002467 | germinal center formation | 3/336 | 15/18670 | 0.002238311 | 0.017400398 | 0.012222355 | BCL3/BCL6/TNFAIP3 | | 3 |
|  | BP | GO:0017014 | protein nitrosylation | 3/336 | 15/18670 | 0.002238311 | 0.017400398 | 0.012222355 | NCOA7/S100A8/S100A9 | | 3 |
|  | BP | GO:0018119 | peptidyl-cysteine S-nitrosylation | 3/336 | 15/18670 | 0.002238311 | 0.017400398 | 0.012222355 | NCOA7/S100A8/S100A9 | | 3 |
|  | BP | GO:0046851 | negative regulation of bone remodeling | 3/336 | 15/18670 | 0.002238311 | 0.017400398 | 0.012222355 | CALCA/IL6/TNFAIP3 | | 3 |
|  | BP | GO:0055074 | calcium ion homeostasis | 18/336 | 471/18670 | 0.002315047 | 0.017963914 | 0.012618179 | ANK2/AVPR1A/C5AR1/CALCA/CCL3/CCL8/CCR1/CXCR1/EDN1/FPR1/FPR2/GJA1/LPAR1/PROK2/PTGER2/SAA1/STC1/WNT5A | | 18 |
|  | BP | GO:0032731 | positive regulation of interleukin-1 beta production | 5/336 | 52/18670 | 0.002382152 | 0.01845077 | 0.012960156 | CCL3/EGR1/MNDA/PANX1/WNT5A | | 5 |
|  | BP | GO:0042063 | gliogenesis | 13/336 | 290/18670 | 0.002401489 | 0.018566538 | 0.013041473 | BMP2/C5AR1/CCL2/CCL3/CD9/DTX1/FPR2/IL1B/IL6/LDLR/LPAR1/S100A8/TLR2 | | 13 |
|  | BP | GO:0030500 | regulation of bone mineralization | 6/336 | 76/18670 | 0.002467487 | 0.019007293 | 0.013351067 | ACVR2B/BMP2/CCL3/CCR1/GJA1/GPM6B | | 6 |
|  | BP | GO:0042310 | vasoconstriction | 6/336 | 76/18670 | 0.002467487 | 0.019007293 | 0.013351067 | AVPR1A/EDN1/GJA1/ICAM1/MIR21/PTGS2 | | 6 |
|  | BP | GO:0045589 | regulation of regulatory T cell differentiation | 4/336 | 32/18670 | 0.00248912 | 0.019035239 | 0.013370697 | BCL6/IRF1/MIR21/SOCS1 | | 4 |
|  | BP | GO:0045987 | positive regulation of smooth muscle contraction | 4/336 | 32/18670 | 0.00248912 | 0.019035239 | 0.013370697 | EDN1/MIR21/PROK2/PTGS2 | | 4 |
|  | BP | GO:0060317 | cardiac epithelial to mesenchymal transition | 4/336 | 32/18670 | 0.00248912 | 0.019035239 | 0.013370697 | BMP2/HAS2/MIR21/MSX1 | | 4 |
|  | BP | GO:0060325 | face morphogenesis | 4/336 | 32/18670 | 0.00248912 | 0.019035239 | 0.013370697 | ARID5B/CRISPLD2/MSX1/TIPARP | | 4 |
|  | BP | GO:0009410 | response to xenobiotic stimulus | 13/336 | 292/18670 | 0.002548522 | 0.019454332 | 0.013665076 | APOBEC3A/DHODH/EDN1/EGR1/FMO1/FOSB/FOXO1/ICAM1/NR4A2/RGS2/S100A12/TIPARP/UGT2B11 | | 13 |
|  | BP | GO:0032715 | negative regulation of interleukin-6 production | 5/336 | 53/18670 | 0.002592182 | 0.019716432 | 0.01384918 | IL10/IRAK3/SIRPA/TNFAIP3/ZC3H12A | | 5 |
|  | BP | GO:0050702 | interleukin-1 beta secretion | 5/336 | 53/18670 | 0.002592182 | 0.019716432 | 0.01384918 | CCL3/PANX1/TNFAIP3/WNT5A/ZC3H12A | | 5 |
|  | BP | GO:0043280 | positive regulation of cysteine-type endopeptidase activity involved in apoptotic process | 8/336 | 132/18670 | 0.002704607 | 0.020491989 | 0.014393945 | CASP4/F3/GRAMD4/MYC/S100A8/S100A9/SOX7/TNFSF10 | | 8 |
|  | BP | GO:0001946 | lymphangiogenesis | 3/336 | 16/18670 | 0.002718374 | 0.020491989 | 0.014393945 | ACVR2B/EPHA2/FOXC1 | | 3 |
|  | BP | GO:0009130 | pyrimidine nucleoside monophosphate biosynthetic process | 3/336 | 16/18670 | 0.002718374 | 0.020491989 | 0.014393945 | DHODH/TYMS/UCK1 | | 3 |
|  | BP | GO:0032695 | negative regulation of interleukin-12 production | 3/336 | 16/18670 | 0.002718374 | 0.020491989 | 0.014393945 | IL10/IRAK3/THBS1 | | 3 |
|  | BP | GO:0045623 | negative regulation of T-helper cell differentiation | 3/336 | 16/18670 | 0.002718374 | 0.020491989 | 0.014393945 | BCL6/IL4R/ZC3H12A | | 3 |
|  | BP | GO:0001655 | urogenital system development | 14/336 | 330/18670 | 0.002748091 | 0.020679144 | 0.014525406 | ACVR2B/ADAMTS1/ARID5B/BMP2/EGR1/FOXC1/FSTL3/HAS2/MYC/SALL1/SDC4/TACSTD2/TIPARP/WNT5A | | 14 |
|  | BP | GO:0048511 | rhythmic process | 13/336 | 295/18670 | 0.002782845 | 0.020903469 | 0.014682976 | ADAMTS1/EGR1/EGR3/HAS2/JUN/KLF10/NAMPT/NFIL3/PROK2/RELB/SERPINE1/SIK1/TYMS | | 13 |
|  | BP | GO:2001024 | negative regulation of response to drug | 4/336 | 33/18670 | 0.002792803 | 0.020941069 | 0.014709387 | IL10/MIR21/NR4A3/RGS2 | | 4 |
|  | BP | GO:0043407 | negative regulation of MAP kinase activity | 6/336 | 78/18670 | 0.002813698 | 0.02096123 | 0.014723548 | DUSP16/DUSP6/IL1B/IRAK3/RGS2/SPRY4 | | 6 |
|  | BP | GO:0070664 | negative regulation of leukocyte proliferation | 6/336 | 78/18670 | 0.002813698 | 0.02096123 | 0.014723548 | CCL8/IL10/MNDA/PELI1/SDC4/TNFAIP3 | | 6 |
|  | BP | GO:0002720 | positive regulation of cytokine production involved in immune response | 5/336 | 54/18670 | 0.002815318 | 0.02096123 | 0.014723548 | IL1B/IL1R1/IL6/NR4A3/WNT5A | | 5 |
|  | BP | GO:1904645 | response to amyloid-beta | 5/336 | 54/18670 | 0.002815318 | 0.02096123 | 0.014723548 | CASP4/FPR2/ICAM1/NAMPT/PARP1 | | 5 |
|  | BP | GO:0030198 | extracellular matrix organization | 15/336 | 368/18670 | 0.002878935 | 0.021397215 | 0.015029792 | ADAMTS4/ADAMTS5/B4GALT1/BCL3/CRISPLD2/ELF3/FOXC1/GPM6B/HAS2/ICAM1/IL6/P4HA1/SERPINE1/THBS1/TNFRSF1A | | 15 |
|  | BP | GO:0032355 | response to estradiol | 8/336 | 134/18670 | 0.002967745 | 0.022018588 | 0.015466256 | CRYAB/GJA1/IGFBP2/IL10/OGG1/PTGS2/SOCS2/STRN3 | | 8 |
|  | BP | GO:0001763 | morphogenesis of a branching structure | 10/336 | 196/18670 | 0.002989383 | 0.022140281 | 0.015551735 | BMP2/EDN1/EPHA2/IL10/MYC/SALL1/SEMA3C/SOCS3/TACSTD2/WNT5A | | 10 |
|  | BP | GO:0071260 | cellular response to mechanical stimulus | 6/336 | 79/18670 | 0.002999848 | 0.022178949 | 0.015578896 | GJA1/IL1B/IRF1/MAP3K14/PTGS2/TNFRSF1A | | 6 |
|  | BP | GO:2001242 | regulation of intrinsic apoptotic signaling pathway | 9/336 | 165/18670 | 0.003064889 | 0.022620274 | 0.015888891 | BCL2A1/MCL1/MIR21/MSX1/PARP1/PLAUR/PTGS2/S100A8/S100A9 | | 9 |
|  | BP | GO:0009749 | response to glucose | 10/336 | 197/18670 | 0.003100364 | 0.02284223 | 0.016044797 | ACVR2B/EGR1/ERN1/GATA4/GCK/GJA1/ICAM1/IRS2/PIM3/THBS1 | | 10 |
|  | BP | GO:0042533 | tumor necrosis factor biosynthetic process | 4/336 | 34/18670 | 0.003120903 | 0.022874004 | 0.016067115 | AKAP12/BCL3/IL10/THBS1 | | 4 |
|  | BP | GO:0042534 | regulation of tumor necrosis factor biosynthetic process | 4/336 | 34/18670 | 0.003120903 | 0.022874004 | 0.016067115 | AKAP12/BCL3/IL10/THBS1 | | 4 |
|  | BP | GO:0045066 | regulatory T cell differentiation | 4/336 | 34/18670 | 0.003120903 | 0.022874004 | 0.016067115 | BCL6/IRF1/MIR21/SOCS1 | | 4 |
|  | BP | GO:0097696 | STAT cascade | 9/336 | 166/18670 | 0.003190685 | 0.023344992 | 0.016397946 | BCL3/CCL2/IL10/IL6/OSM/SOCS1/SOCS2/SOCS3/TNFRSF1A | | 9 |
|  | BP | GO:0046890 | regulation of lipid biosynthetic process | 10/336 | 198/18670 | 0.003214606 | 0.023479394 | 0.016492352 | AVPR1A/BMP2/EGR1/FPR2/IL1B/LDLR/PTGS2/SIK1/SIRT4/TNFRSF1A | | 10 |
|  | BP | GO:0007596 | blood coagulation | 14/336 | 336/18670 | 0.003229982 | 0.023551026 | 0.016542668 | CD9/EDN1/F3/GATA4/IL6/IRF1/MAFF/NFE2/PLAU/PLAUR/PLSCR1/SAA1/SERPINE1/THBS1 | | 14 |
|  | BP | GO:0009129 | pyrimidine nucleoside monophosphate metabolic process | 3/336 | 17/18670 | 0.003257225 | 0.023668046 | 0.016624865 | DHODH/TYMS/UCK1 | | 3 |
|  | BP | GO:1905331 | negative regulation of morphogenesis of an epithelium | 3/336 | 17/18670 | 0.003257225 | 0.023668046 | 0.016624865 | MIR21/TACSTD2/WNT5A | | 3 |
|  | BP | GO:0001701 | in utero embryonic development | 15/336 | 373/18670 | 0.003266565 | 0.023695204 | 0.016643941 | BMP2/EDN1/ELF3/ELL/FOSL1/FOXC1/GJA1/IL10/JUNB/MAFF/MSX1/POLG2/SLC25A34/SOCS3/ZFP14 | | 15 |
|  | BP | GO:1903202 | negative regulation of oxidative stress-induced cell death | 5/336 | 56/18670 | 0.003302748 | 0.023916643 | 0.016799484 | GATA4/IL10/MIR21/NCOA7/NR4A3 | | 5 |
|  | BP | GO:0046425 | regulation of JAK-STAT cascade | 8/336 | 137/18670 | 0.003399369 | 0.024533732 | 0.017232938 | BCL3/IL10/IL6/OSM/SOCS1/SOCS2/SOCS3/TNFRSF1A | | 8 |
|  | BP | GO:0110110 | positive regulation of animal organ morphogenesis | 6/336 | 81/18670 | 0.003399567 | 0.024533732 | 0.017232938 | BMP2/EDN1/MYC/PIM1/SEMA3C/TNFAIP3 | | 6 |
|  | BP | GO:0001893 | maternal placenta development | 4/336 | 35/18670 | 0.003474345 | 0.024988102 | 0.017552096 | GJA1/JUNB/PTGS2/STC1 | | 4 |
|  | BP | GO:0098801 | regulation of renal system process | 4/336 | 35/18670 | 0.003474345 | 0.024988102 | 0.017552096 | AVPR1A/EDN1/GJA1/STC1 | | 4 |
|  | BP | GO:0060760 | positive regulation of response to cytokine stimulus | 5/336 | 57/18670 | 0.003567961 | 0.025531144 | 0.017933538 | CASP4/EDN1/IL1R1/TLR2/WNT5A | | 5 |
|  | BP | GO:1900408 | negative regulation of cellular response to oxidative stress | 5/336 | 57/18670 | 0.003567961 | 0.025531144 | 0.017933538 | GATA4/IL10/MIR21/NCOA7/NR4A3 | | 5 |
|  | BP | GO:1903428 | positive regulation of reactive oxygen species biosynthetic process | 5/336 | 57/18670 | 0.003567961 | 0.025531144 | 0.017933538 | EDN1/ICAM1/IL1B/KLF4/PTGS2 | | 5 |
|  | BP | GO:0007599 | hemostasis | 14/336 | 341/18670 | 0.003682553 | 0.026306614 | 0.018478243 | CD9/EDN1/F3/GATA4/IL6/IRF1/MAFF/NFE2/PLAU/PLAUR/PLSCR1/SAA1/SERPINE1/THBS1 | | 14 |
|  | BP | GO:0007548 | sex differentiation | 12/336 | 270/18670 | 0.00372863 | 0.026590851 | 0.018677896 | ADAMTS1/ARID5B/FOXC1/FSTL3/GATA4/GJA1/ICAM1/INHBB/SALL1/TIPARP/TNFSF10/WNT5A | | 12 |
|  | BP | GO:0043433 | negative regulation of DNA-binding transcription factor activity | 9/336 | 170/18670 | 0.003735094 | 0.026592107 | 0.018678778 | EGLN1/IL10/IRAK3/KLF4/PELI1/PIM1/SIK1/TNFAIP3/ZC3H12A | | 9 |
|  | BP | GO:0033559 | unsaturated fatty acid metabolic process | 7/336 | 110/18670 | 0.003758546 | 0.026714104 | 0.018764472 | ALOX5AP/AVPR1A/EDN1/FADS1/IL1B/PTGS2/TNFRSF1A | | 7 |
|  | BP | GO:0050817 | coagulation | 14/336 | 342/18670 | 0.003778996 | 0.026814385 | 0.01883491 | CD9/EDN1/F3/GATA4/IL6/IRF1/MAFF/NFE2/PLAU/PLAUR/PLSCR1/SAA1/SERPINE1/THBS1 | | 14 |
|  | BP | GO:0030278 | regulation of ossification | 10/336 | 203/18670 | 0.003837101 | 0.026960881 | 0.018937812 | ACVR2B/BMP2/CCL3/CCR1/CEBPD/GJA1/GPM6B/IL6/MIR21/WNT5A | | 10 |
|  | BP | GO:0031663 | lipopolysaccharide-mediated signaling pathway | 5/336 | 58/18670 | 0.003848118 | 0.026960881 | 0.018937812 | CCL2/CCL3/IL1B/TLR2/TNFAIP3 | | 5 |
|  | BP | GO:1902622 | regulation of neutrophil migration | 4/336 | 36/18670 | 0.003854034 | 0.026960881 | 0.018937812 | C5AR1/EDN1/IL1R1/MIR223 | | 4 |
|  | BP | GO:0002902 | regulation of B cell apoptotic process | 3/336 | 18/18670 | 0.003857019 | 0.026960881 | 0.018937812 | BCL6/IL10/IRS2 | | 3 |
|  | BP | GO:0035994 | response to muscle stretch | 3/336 | 18/18670 | 0.003857019 | 0.026960881 | 0.018937812 | EDN1/FOS/JUN | | 3 |
|  | BP | GO:0044320 | cellular response to leptin stimulus | 3/336 | 18/18670 | 0.003857019 | 0.026960881 | 0.018937812 | GCK/INHBB/NR4A3 | | 3 |
|  | BP | GO:0090026 | positive regulation of monocyte chemotaxis | 3/336 | 18/18670 | 0.003857019 | 0.026960881 | 0.018937812 | CCR1/FPR2/SERPINE1 | | 3 |
|  | BP | GO:0150078 | positive regulation of neuroinflammatory response | 3/336 | 18/18670 | 0.003857019 | 0.026960881 | 0.018937812 | CCL3/IL1B/IL6 | | 3 |
|  | BP | GO:2000319 | regulation of T-helper 17 cell differentiation | 3/336 | 18/18670 | 0.003857019 | 0.026960881 | 0.018937812 | MIR21/NFKBIZ/ZC3H12A | | 3 |
|  | BP | GO:0034620 | cellular response to unfolded protein | 8/336 | 140/18670 | 0.003878181 | 0.027001273 | 0.018966184 | ATF3/CCL2/CREB3L3/ERN1/HSPA2/IGFBP1/PPP1R15A/RNF175 | | 8 |
|  | BP | GO:0046330 | positive regulation of JNK cascade | 8/336 | 140/18670 | 0.003878181 | 0.027001273 | 0.018966184 | EDAR/EDN1/ERN1/GADD45B/GADD45G/IL1B/IL1RN/WNT5A | | 8 |
|  | BP | GO:0006937 | regulation of muscle contraction | 9/336 | 171/18670 | 0.003881952 | 0.027001273 | 0.018966184 | ANK2/EDN1/GATA4/MIR21/PROK2/PTGS2/RGS2/STC1/ZC3H12A | | 9 |
|  | BP | GO:0072676 | lymphocyte migration | 7/336 | 111/18670 | 0.003951191 | 0.027437747 | 0.019272771 | CCL2/CCL3/CCL8/CH25H/ICAM1/SAA1/WNT5A | | 7 |
|  | BP | GO:0051048 | negative regulation of secretion | 11/336 | 238/18670 | 0.00402697 | 0.027918122 | 0.019610195 | EDN1/IL10/IL1B/INHBB/MIDN/OSM/PIM3/SIRT4/TNFAIP3/TNFRSF1A/ZC3H12A | | 11 |
|  | BP | GO:0008286 | insulin receptor signaling pathway | 8/336 | 141/18670 | 0.004048844 | 0.027978039 | 0.019652282 | CISH/FOXO1/IGFBP1/IL1B/IRS2/SOCS1/SOCS2/SOCS3 | | 8 |
|  | BP | GO:0050709 | negative regulation of protein secretion | 8/336 | 141/18670 | 0.004048844 | 0.027978039 | 0.019652282 | IL10/IL1B/INHBB/MIDN/PIM3/SIRT4/TNFAIP3/ZC3H12A | | 8 |
|  | BP | GO:0097756 | negative regulation of blood vessel diameter | 6/336 | 84/18670 | 0.004071711 | 0.028090159 | 0.019731037 | AVPR1A/EDN1/GJA1/ICAM1/MIR21/PTGS2 | | 6 |
|  | BP | GO:0010656 | negative regulation of muscle cell apoptotic process | 5/336 | 59/18670 | 0.004143676 | 0.028401303 | 0.01994959 | EDN1/GATA4/MIR21/SIRT4/ZC3H12A | | 5 |
|  | BP | GO:0045071 | negative regulation of viral genome replication | 5/336 | 59/18670 | 0.004143676 | 0.028401303 | 0.01994959 | APOBEC3A/CCL8/ISG20/PLSCR1/ZC3H12A | | 5 |
|  | BP | GO:1902883 | negative regulation of response to oxidative stress | 5/336 | 59/18670 | 0.004143676 | 0.028401303 | 0.01994959 | GATA4/IL10/MIR21/NCOA7/NR4A3 | | 5 |
|  | BP | GO:2000351 | regulation of endothelial cell apoptotic process | 5/336 | 59/18670 | 0.004143676 | 0.028401303 | 0.01994959 | CCL2/ICAM1/SERPINE1/THBS1/TNFAIP3 | | 5 |
|  | BP | GO:0060419 | heart growth | 7/336 | 112/18670 | 0.004151234 | 0.028407071 | 0.019953642 | DUSP6/EDN1/FOXC1/GATA4/GJA1/PIM1/RGS2 | | 7 |
|  | BP | GO:0010614 | negative regulation of cardiac muscle hypertrophy | 4/336 | 37/18670 | 0.004260861 | 0.028876892 | 0.020283653 | FOXO1/MIR21/RGS2/TNFRSF1A | | 4 |
|  | BP | GO:0030212 | hyaluronan metabolic process | 4/336 | 37/18670 | 0.004260861 | 0.028876892 | 0.020283653 | HAS2/HMMR/IL1B/PIM1 | | 4 |
|  | BP | GO:0034405 | response to fluid shear stress | 4/336 | 37/18670 | 0.004260861 | 0.028876892 | 0.020283653 | GJA1/HAS2/KLF4/PTGS2 | | 4 |
|  | BP | GO:0060323 | head morphogenesis | 4/336 | 37/18670 | 0.004260861 | 0.028876892 | 0.020283653 | ARID5B/CRISPLD2/MSX1/TIPARP | | 4 |
|  | BP | GO:0071276 | cellular response to cadmium ion | 4/336 | 37/18670 | 0.004260861 | 0.028876892 | 0.020283653 | FOS/JUN/MT1A/OGG1 | | 4 |
|  | BP | GO:1903427 | negative regulation of reactive oxygen species biosynthetic process | 4/336 | 37/18670 | 0.004260861 | 0.028876892 | 0.020283653 | IL10/MIR21/SIRPA/ZC3H12A | | 4 |
|  | BP | GO:0043062 | extracellular structure organization | 16/336 | 422/18670 | 0.004286399 | 0.029003492 | 0.020372579 | ADAMTS4/ADAMTS5/B4GALT1/BCL3/CRISPLD2/ELF3/FOXC1/GPM6B/HAS2/ICAM1/IL6/P4HA1/SDC4/SERPINE1/THBS1/TNFRSF1A | | 16 |
|  | BP | GO:1903901 | negative regulation of viral life cycle | 6/336 | 85/18670 | 0.004316191 | 0.02915842 | 0.020481403 | APOBEC3A/CCL8/ISG20/PLSCR1/TRIM15/ZC3H12A | | 6 |
|  | BP | GO:0030073 | insulin secretion | 10/336 | 207/18670 | 0.00440081 | 0.029539349 | 0.020748974 | ACVR2B/GCK/GJA1/IL1B/IL1RN/INHBB/IRS2/MIDN/PIM3/SIRT4 | | 10 |
|  | BP | GO:0030879 | mammary gland development | 8/336 | 143/18670 | 0.004407503 | 0.029539349 | 0.020748974 | DHODH/ELF3/EPHA2/GJA1/IRS2/MSX1/SOCS2/WNT5A | | 8 |
|  | BP | GO:0035296 | regulation of tube diameter | 8/336 | 143/18670 | 0.004407503 | 0.029539349 | 0.020748974 | AVPR1A/EDN1/FOXC1/GJA1/ICAM1/MIR21/PTGS2/RGS2 | | 8 |
|  | BP | GO:0050880 | regulation of blood vessel size | 8/336 | 143/18670 | 0.004407503 | 0.029539349 | 0.020748974 | AVPR1A/EDN1/FOXC1/GJA1/ICAM1/MIR21/PTGS2/RGS2 | | 8 |
|  | BP | GO:0097746 | regulation of blood vessel diameter | 8/336 | 143/18670 | 0.004407503 | 0.029539349 | 0.020748974 | AVPR1A/EDN1/FOXC1/GJA1/ICAM1/MIR21/PTGS2/RGS2 | | 8 |
|  | BP | GO:0046879 | hormone secretion | 13/336 | 312/18670 | 0.004466313 | 0.029886136 | 0.020992564 | ACVR2B/EDN1/GCK/GJA1/IL1B/IL1RN/IL6/INHBB/IRS2/MIDN/OSM/PIM3/SIRT4 | | 13 |
|  | BP | GO:0120032 | regulation of plasma membrane bounded cell projection assembly | 9/336 | 175/18670 | 0.004515154 | 0.029959222 | 0.021043901 | CDC42EP1/DPYSL3/EPHA2/FRMD7/ICAM1/KLF5/MAK/RAP1GAP/TACSTD2 | | 9 |
|  | BP | GO:0002689 | negative regulation of leukocyte chemotaxis | 3/336 | 19/18670 | 0.004519741 | 0.029959222 | 0.021043901 | CCL2/MIR223/NBL1 | | 3 |
|  | BP | GO:0002726 | positive regulation of T cell cytokine production | 3/336 | 19/18670 | 0.004519741 | 0.029959222 | 0.021043901 | IL1B/IL1R1/IL6 | | 3 |
|  | BP | GO:0032288 | myelin assembly | 3/336 | 19/18670 | 0.004519741 | 0.029959222 | 0.021043901 | ANK2/CD9/TLR2 | | 3 |
|  | BP | GO:0036303 | lymph vessel morphogenesis | 3/336 | 19/18670 | 0.004519741 | 0.029959222 | 0.021043901 | ACVR2B/EPHA2/FOXC1 | | 3 |
|  | BP | GO:0060004 | reflex | 3/336 | 19/18670 | 0.004519741 | 0.029959222 | 0.021043901 | ALDH1A3/GJA1/NR4A3 | | 3 |
|  | BP | GO:0050792 | regulation of viral process | 10/336 | 208/18670 | 0.004551456 | 0.030122233 | 0.021158403 | APOBEC3A/CCL3/CCL8/ISG20/JUN/PLSCR1/TMPRSS2/TRIM15/ZC3H12A/ZFP36 | | 10 |
|  | BP | GO:0070542 | response to fatty acid | 6/336 | 86/18670 | 0.004571338 | 0.030206544 | 0.021217624 | EDN1/LDLR/PTGER2/PTGS2/TLR2/ZC3H12A | | 6 |
|  | BP | GO:0035150 | regulation of tube size | 8/336 | 144/18670 | 0.004595751 | 0.030320488 | 0.021297661 | AVPR1A/EDN1/FOXC1/GJA1/ICAM1/MIR21/PTGS2/RGS2 | | 8 |
|  | BP | GO:0006986 | response to unfolded protein | 9/336 | 176/18670 | 0.004685363 | 0.030740088 | 0.021592395 | ATF3/CCL2/CREB3L3/ERN1/HSPA2/IGFBP1/PPP1R15A/RNF175/THBS1 | | 9 |
|  | BP | GO:0051897 | positive regulation of protein kinase B signaling | 9/336 | 176/18670 | 0.004685363 | 0.030740088 | 0.021592395 | CCL3/F3/FAM110C/HBEGF/IRS2/MIR21/OSM/THBS1/TNFAIP8L3 | | 9 |
|  | BP | GO:0046636 | negative regulation of alpha-beta T cell activation | 4/336 | 38/18670 | 0.004695696 | 0.030740088 | 0.021592395 | BCL6/IL4R/SOCS1/ZC3H12A | | 4 |
|  | BP | GO:0046676 | negative regulation of insulin secretion | 4/336 | 38/18670 | 0.004695696 | 0.030740088 | 0.021592395 | INHBB/MIDN/PIM3/SIRT4 | | 4 |
|  | BP | GO:0050716 | positive regulation of interleukin-1 secretion | 4/336 | 38/18670 | 0.004695696 | 0.030740088 | 0.021592395 | CCL3/PANX1/SAA1/WNT5A | | 4 |
|  | BP | GO:0030888 | regulation of B cell proliferation | 5/336 | 61/18670 | 0.004782813 | 0.031165228 | 0.021891022 | BCL6/IL10/IRS2/MNDA/PELI1 | | 5 |
|  | BP | GO:0034113 | heterotypic cell-cell adhesion | 5/336 | 61/18670 | 0.004782813 | 0.031165228 | 0.021891022 | IL10/IL1B/IL1RN/KLF4/SIRPA | | 5 |
|  | BP | GO:0045123 | cellular extravasation | 5/336 | 61/18670 | 0.004782813 | 0.031165228 | 0.021891022 | CCL2/ICAM1/IL1R1/SELL/SIRPA | | 5 |
|  | BP | GO:0071901 | negative regulation of protein serine/threonine kinase activity | 8/336 | 145/18670 | 0.004790115 | 0.031165228 | 0.021891022 | DUSP16/DUSP6/HEXIM2/IL1B/IRAK3/RGS2/SPRY4/TNFAIP3 | | 8 |
|  | BP | GO:0046660 | female sex differentiation | 7/336 | 115/18670 | 0.004797536 | 0.03116556 | 0.021891255 | ADAMTS1/ARID5B/FOXC1/ICAM1/INHBB/TIPARP/WNT5A | | 7 |
|  | BP | GO:1904892 | regulation of STAT cascade | 8/336 | 146/18670 | 0.004990723 | 0.032370807 | 0.022737842 | BCL3/IL10/IL6/OSM/SOCS1/SOCS2/SOCS3/TNFRSF1A | | 8 |
|  | BP | GO:0010950 | positive regulation of endopeptidase activity | 9/336 | 178/18670 | 0.005040735 | 0.032645126 | 0.022930529 | CASP4/F3/GRAMD4/MIR21/MYC/S100A8/S100A9/SOX7/TNFSF10 | | 9 |
|  | BP | GO:0009791 | post-embryonic development | 6/336 | 88/18670 | 0.005114722 | 0.0329534 | 0.023147066 | ACVR2B/ARID5B/KLF4/NR4A2/SEMA3C/TIPARP | | 6 |
|  | BP | GO:0032088 | negative regulation of NF-kappaB transcription factor activity | 6/336 | 88/18670 | 0.005114722 | 0.0329534 | 0.023147066 | IL10/IRAK3/KLF4/PELI1/TNFAIP3/ZC3H12A | | 6 |
|  | BP | GO:1901888 | regulation of cell junction assembly | 6/336 | 88/18670 | 0.005114722 | 0.0329534 | 0.023147066 | EPHA2/FAM107A/GJA1/GPM6B/SDC4/THBS1 | | 6 |
|  | BP | GO:0032623 | interleukin-2 production | 5/336 | 62/18670 | 0.005127297 | 0.0329534 | 0.023147066 | CD83/IL1B/SLC11A1/TNFAIP3/ZFP36 | | 5 |
|  | BP | GO:0046635 | positive regulation of alpha-beta T cell activation | 5/336 | 62/18670 | 0.005127297 | 0.0329534 | 0.023147066 | CD83/IL4R/MIR21/NFKBIZ/SOCS1 | | 5 |
|  | BP | GO:0010761 | fibroblast migration | 4/336 | 39/18670 | 0.00515939 | 0.032959303 | 0.023151212 | AKAP12/ARID5B/SDC4/THBS1 | | 4 |
|  | BP | GO:0014741 | negative regulation of muscle hypertrophy | 4/336 | 39/18670 | 0.00515939 | 0.032959303 | 0.023151212 | FOXO1/MIR21/RGS2/TNFRSF1A | | 4 |
|  | BP | GO:1905521 | regulation of macrophage migration | 4/336 | 39/18670 | 0.00515939 | 0.032959303 | 0.023151212 | C5AR1/CCL3/CD9/THBS1 | | 4 |
|  | BP | GO:2000826 | regulation of heart morphogenesis | 4/336 | 39/18670 | 0.00515939 | 0.032959303 | 0.023151212 | BMP2/FOXC1/PIM1/SEMA3C | | 4 |
|  | BP | GO:0048771 | tissue remodeling | 9/336 | 179/18670 | 0.005226088 | 0.03297241 | 0.023160419 | ACVR2B/CALCA/ELF3/EPHA2/FOXC1/GJA1/IL6/SEMA3C/TNFAIP3 | | 9 |
|  | BP | GO:0002374 | cytokine secretion involved in immune response | 3/336 | 20/18670 | 0.005247205 | 0.03297241 | 0.023160419 | IL10/TLR2/WNT5A | | 3 |
|  | BP | GO:0002827 | positive regulation of T-helper 1 type immune response | 3/336 | 20/18670 | 0.005247205 | 0.03297241 | 0.023160419 | IL1B/IL1R1/SLC11A1 | | 3 |
|  | BP | GO:0006525 | arginine metabolic process | 3/336 | 20/18670 | 0.005247205 | 0.03297241 | 0.023160419 | MIR21/NAGS/PADI4 | | 3 |
|  | BP | GO:0034104 | negative regulation of tissue remodeling | 3/336 | 20/18670 | 0.005247205 | 0.03297241 | 0.023160419 | CALCA/IL6/TNFAIP3 | | 3 |
|  | BP | GO:0043371 | negative regulation of CD4-positive, alpha-beta T cell differentiation | 3/336 | 20/18670 | 0.005247205 | 0.03297241 | 0.023160419 | BCL6/IL4R/ZC3H12A | | 3 |
|  | BP | GO:0045019 | negative regulation of nitric oxide biosynthetic process | 3/336 | 20/18670 | 0.005247205 | 0.03297241 | 0.023160419 | IL10/SIRPA/ZC3H12A | | 3 |
|  | BP | GO:1903204 | negative regulation of oxidative stress-induced neuron death | 3/336 | 20/18670 | 0.005247205 | 0.03297241 | 0.023160419 | IL10/NCOA7/NR4A3 | | 3 |
|  | BP | GO:1903978 | regulation of microglial cell activation | 3/336 | 20/18670 | 0.005247205 | 0.03297241 | 0.023160419 | CCL3/IL6/LDLR | | 3 |
|  | BP | GO:1904406 | negative regulation of nitric oxide metabolic process | 3/336 | 20/18670 | 0.005247205 | 0.03297241 | 0.023160419 | IL10/SIRPA/ZC3H12A | | 3 |
|  | BP | GO:1905523 | positive regulation of macrophage migration | 3/336 | 20/18670 | 0.005247205 | 0.03297241 | 0.023160419 | C5AR1/CCL3/THBS1 | | 3 |
|  | BP | GO:0007193 | adenylate cyclase-inhibiting G protein-coupled receptor signaling pathway | 6/336 | 89/18670 | 0.005403504 | 0.033851897 | 0.023778187 | AKAP12/EDN1/FPR2/LPAR1/RGS1/RGS2 | | 6 |
|  | BP | GO:0046427 | positive regulation of JAK-STAT cascade | 6/336 | 89/18670 | 0.005403504 | 0.033851897 | 0.023778187 | IL10/IL6/OSM/SOCS1/SOCS3/TNFRSF1A | | 6 |
|  | BP | GO:0002792 | negative regulation of peptide secretion | 8/336 | 148/18670 | 0.005411181 | 0.033851897 | 0.023778187 | IL10/IL1B/INHBB/MIDN/PIM3/SIRT4/TNFAIP3/ZC3H12A | | 8 |
|  | BP | GO:0042471 | ear morphogenesis | 7/336 | 118/18670 | 0.005516874 | 0.034462127 | 0.024206823 | ALDH1A3/EDN1/LRIG1/MSX1/NR4A3/SALL1/WNT5A | | 7 |
|  | BP | GO:0010565 | regulation of cellular ketone metabolic process | 9/336 | 181/18670 | 0.005612613 | 0.034949676 | 0.024549286 | AVPR1A/BMP2/EGR1/IL1B/IRS2/MIR21/NR4A3/PTGS2/SIRT4 | | 9 |
|  | BP | GO:0045926 | negative regulation of growth | 11/336 | 249/18670 | 0.005627853 | 0.034949676 | 0.024549286 | BCL6/CRYAB/GJA1/MSX1/MT1A/OSGIN2/RGS2/SEMA3C/SEMA4B/SOCS2/WNT5A | | 11 |
|  | BP | GO:0071560 | cellular response to transforming growth factor beta stimulus | 11/336 | 249/18670 | 0.005627853 | 0.034949676 | 0.024549286 | ACVR2B/EDN1/FOS/JUN/LRG1/MIR21/ONECUT1/PARP1/PEG10/THBS1/WNT5A | | 11 |
|  | BP | GO:2001056 | positive regulation of cysteine-type endopeptidase activity | 8/336 | 149/18670 | 0.00563129 | 0.034949676 | 0.024549286 | CASP4/F3/GRAMD4/MYC/S100A8/S100A9/SOX7/TNFSF10 | | 8 |
|  | BP | GO:0002823 | negative regulation of adaptive immune response based on somatic recombination of immune receptors built from immunoglobulin superfamily domains | 4/336 | 40/18670 | 0.005652773 | 0.034949676 | 0.024549286 | BCL6/IL1RL1/IL4R/ZC3H12A | | 4 |
|  | BP | GO:0098760 | response to interleukin-7 | 4/336 | 40/18670 | 0.005652773 | 0.034949676 | 0.024549286 | CISH/IRS2/SOCS1/SOCS2 | | 4 |
|  | BP | GO:0098761 | cellular response to interleukin-7 | 4/336 | 40/18670 | 0.005652773 | 0.034949676 | 0.024549286 | CISH/IRS2/SOCS1/SOCS2 | | 4 |
|  | BP | GO:0010717 | regulation of epithelial to mesenchymal transition | 6/336 | 90/18670 | 0.005704041 | 0.035215165 | 0.024735771 | BMP2/FOXC1/GLIPR2/IL1B/IL6/MIR21 | | 6 |
|  | BP | GO:0010810 | regulation of cell-substrate adhesion | 10/336 | 215/18670 | 0.005723043 | 0.035280973 | 0.024781996 | BCL6/FAM107A/GPM6B/HAS2/ONECUT1/PLAU/SDC4/SERPINE1/TACSTD2/THBS1 | | 10 |
|  | BP | GO:0071383 | cellular response to steroid hormone stimulus | 11/336 | 250/18670 | 0.005795205 | 0.03567383 | 0.025057945 | EDN1/FAM107A/FOXO1/ICAM1/NR4A1/NR4A2/NR4A3/PARP1/STC1/STRN3/ZFP36 | | 11 |
|  | BP | GO:0002440 | production of molecular mediator of immune response | 12/336 | 286/18670 | 0.005860334 | 0.03586295 | 0.025190787 | BCL6/IL10/IL18RAP/IL1B/IL1R1/IL4R/IL6/IRAK3/NR4A3/SLC11A1/TLR2/WNT5A | | 12 |
|  | BP | GO:0045669 | positive regulation of osteoblast differentiation | 5/336 | 64/18670 | 0.005868329 | 0.03586295 | 0.025190787 | ACVR2B/BMP2/CEBPD/IL6/MIR21 | | 5 |
|  | BP | GO:0045670 | regulation of osteoclast differentiation | 5/336 | 64/18670 | 0.005868329 | 0.03586295 | 0.025190787 | CCL3/CCR1/FOS/FSTL3/KLF10 | | 5 |
|  | BP | GO:0060135 | maternal process involved in female pregnancy | 5/336 | 64/18670 | 0.005868329 | 0.03586295 | 0.025190787 | GJA1/JUNB/PTGS2/RGS2/STC1 | | 5 |
|  | BP | GO:2000378 | negative regulation of reactive oxygen species metabolic process | 5/336 | 64/18670 | 0.005868329 | 0.03586295 | 0.025190787 | CRYAB/IL10/MIR21/SIRPA/ZC3H12A | | 5 |
|  | BP | GO:0002699 | positive regulation of immune effector process | 10/336 | 216/18670 | 0.005908033 | 0.036053497 | 0.02532463 | IL18RAP/IL1B/IL1R1/IL4R/IL6/MIR21/NFKBIZ/NR4A3/TRIM15/WNT5A | | 10 |
|  | BP | GO:0008016 | regulation of heart contraction | 11/336 | 251/18670 | 0.005966453 | 0.036357539 | 0.025538195 | ANK2/AVPR1A/EDN1/GATA4/GJA1/HBEGF/KCNE4/KCNK1/RGS2/STC1/ZC3H12A | | 11 |
|  | BP | GO:0031331 | positive regulation of cellular catabolic process | 14/336 | 361/18670 | 0.006038345 | 0.036444612 | 0.025599357 | BTG2/FOXO1/IL1B/IL6/IRS2/LDLR/PFKFB3/PIM2/RNF152/RNF180/SUPV3L1/TNFAIP3/ZC3H12A/ZFP36 | | 14 |
|  | BP | GO:0032891 | negative regulation of organic acid transport | 3/336 | 21/18670 | 0.006041067 | 0.036444612 | 0.025599357 | IRS2/RGS2/THBS1 | | 3 |
|  | BP | GO:0048245 | eosinophil chemotaxis | 3/336 | 21/18670 | 0.006041067 | 0.036444612 | 0.025599357 | CCL2/CCL3/CCL8 | | 3 |
|  | BP | GO:0071498 | cellular response to fluid shear stress | 3/336 | 21/18670 | 0.006041067 | 0.036444612 | 0.025599357 | HAS2/KLF4/PTGS2 | | 3 |
|  | BP | GO:0072111 | cell proliferation involved in kidney development | 3/336 | 21/18670 | 0.006041067 | 0.036444612 | 0.025599357 | BMP2/EGR1/MYC | | 3 |
|  | BP | GO:0090280 | positive regulation of calcium ion import | 3/336 | 21/18670 | 0.006041067 | 0.036444612 | 0.025599357 | CCL2/CCL3/STC1 | | 3 |
|  | BP | GO:2000316 | regulation of T-helper 17 type immune response | 3/336 | 21/18670 | 0.006041067 | 0.036444612 | 0.025599357 | MIR21/NFKBIZ/ZC3H12A | | 3 |
|  | BP | GO:0036294 | cellular response to decreased oxygen levels | 10/336 | 217/18670 | 0.006097657 | 0.036733607 | 0.025802352 | EDN1/EGLN1/EGR1/ICAM1/MIR21/MYC/NAMPT/PTGS2/SIRT4/STC1 | | 10 |
|  | BP | GO:0010955 | negative regulation of protein processing | 4/336 | 41/18670 | 0.006176656 | 0.037103802 | 0.026062384 | BIRC3/SERPINE1/SIRT4/THBS1 | | 4 |
|  | BP | GO:1903318 | negative regulation of protein maturation | 4/336 | 41/18670 | 0.006176656 | 0.037103802 | 0.026062384 | BIRC3/SERPINE1/SIRT4/THBS1 | | 4 |
|  | BP | GO:0006940 | regulation of smooth muscle contraction | 5/336 | 65/18670 | 0.006265761 | 0.03758568 | 0.026400863 | EDN1/MIR21/PROK2/PTGS2/RGS2 | | 5 |
|  | BP | GO:0030968 | endoplasmic reticulum unfolded protein response | 7/336 | 121/18670 | 0.006314145 | 0.037822262 | 0.026567043 | ATF3/CCL2/CREB3L3/ERN1/IGFBP1/PPP1R15A/RNF175 | | 7 |
|  | BP | GO:1904894 | positive regulation of STAT cascade | 6/336 | 92/18670 | 0.006341466 | 0.03793219 | 0.026644258 | IL10/IL6/OSM/SOCS1/SOCS3/TNFRSF1A | | 6 |
|  | BP | GO:0046328 | regulation of JNK cascade | 9/336 | 185/18670 | 0.006451668 | 0.038536871 | 0.027068997 | EDAR/EDN1/ERN1/GADD45B/GADD45G/IL1B/IL1RN/SIRPA/WNT5A | | 9 |
|  | BP | GO:0043583 | ear development | 10/336 | 219/18670 | 0.006491112 | 0.038717788 | 0.027196077 | ALDH1A3/BMP2/CEBPD/EDN1/LRIG1/MSX1/NR4A3/SALL1/SDC4/WNT5A | | 10 |
|  | BP | GO:0051924 | regulation of calcium ion transport | 11/336 | 254/18670 | 0.0065042 | 0.038741211 | 0.02721253 | ANK2/CCL2/CCL3/CCR1/GEM/GJA1/HSPA2/ICAM1/MIR21/PTGS2/STC1 | | 11 |
|  | BP | GO:0045807 | positive regulation of endocytosis | 8/336 | 153/18670 | 0.006580638 | 0.039141375 | 0.027493612 | CCL2/FPR2/IL1B/IL2RB/SERPINE1/SIRPA/SLC11A1/WNT5A | | 8 |
|  | BP | GO:0030308 | negative regulation of cell growth | 9/336 | 186/18670 | 0.006675758 | 0.039520276 | 0.027759758 | BCL6/CRYAB/GJA1/MSX1/OSGIN2/RGS2/SEMA3C/SEMA4B/WNT5A | | 9 |
|  | BP | GO:0033273 | response to vitamin | 6/336 | 93/18670 | 0.006678895 | 0.039520276 | 0.027759758 | GATA4/OGG1/PIM1/PTGS2/STC1/TYMS | | 6 |
|  | BP | GO:0046626 | regulation of insulin receptor signaling pathway | 5/336 | 66/18670 | 0.006681721 | 0.039520276 | 0.027759758 | CISH/IL1B/SOCS1/SOCS2/SOCS3 | | 5 |
|  | BP | GO:1905207 | regulation of cardiocyte differentiation | 5/336 | 66/18670 | 0.006681721 | 0.039520276 | 0.027759758 | BMP2/EDN1/GATA4/RGS2/SEMA3C | | 5 |
|  | BP | GO:0071559 | response to transforming growth factor beta | 11/336 | 255/18670 | 0.006691655 | 0.039523758 | 0.027762204 | ACVR2B/EDN1/FOS/JUN/LRG1/MIR21/ONECUT1/PARP1/PEG10/THBS1/WNT5A | | 11 |
|  | BP | GO:0010613 | positive regulation of cardiac muscle hypertrophy | 4/336 | 42/18670 | 0.006731824 | 0.039650253 | 0.027851057 | EDN1/MIR21/NR4A3/PARP1 | | 4 |
|  | BP | GO:0021983 | pituitary gland development | 4/336 | 42/18670 | 0.006731824 | 0.039650253 | 0.027851057 | BMP2/MSX1/SALL1/WNT5A | | 4 |
|  | BP | GO:0007187 | G protein-coupled receptor signaling pathway, coupled to cyclic nucleotide second messenger | 11/336 | 256/18670 | 0.006883319 | 0.040154127 | 0.028204987 | AKAP12/CALCA/CCL2/CCR1/EDN1/FPR1/FPR2/LPAR1/PTGER2/RGS1/RGS2 | | 11 |
|  | BP | GO:0043900 | regulation of multi-organism process | 15/336 | 405/18670 | 0.006887917 | 0.040154127 | 0.028204987 | APOBEC3A/B4GALT1/BIRC3/CCL3/CCL8/IL1B/INHBB/ISG20/JUN/PLSCR1/TMPRSS2/TNFAIP3/TRIM15/ZC3H12A/ZFP36 | | 15 |
|  | BP | GO:0010226 | response to lithium ion | 3/336 | 22/18670 | 0.006902826 | 0.040154127 | 0.028204987 | ARL13B/IGFBP2/PTGS2 | | 3 |
|  | BP | GO:0040037 | negative regulation of fibroblast growth factor receptor signaling pathway | 3/336 | 22/18670 | 0.006902826 | 0.040154127 | 0.028204987 | SPRY4/THBS1/WNT5A | | 3 |
|  | BP | GO:0045624 | positive regulation of T-helper cell differentiation | 3/336 | 22/18670 | 0.006902826 | 0.040154127 | 0.028204987 | IL4R/MIR21/NFKBIZ | | 3 |
|  | BP | GO:0045723 | positive regulation of fatty acid biosynthetic process | 3/336 | 22/18670 | 0.006902826 | 0.040154127 | 0.028204987 | AVPR1A/IL1B/PTGS2 | | 3 |
|  | BP | GO:0060547 | negative regulation of necrotic cell death | 3/336 | 22/18670 | 0.006902826 | 0.040154127 | 0.028204987 | BIRC3/MIR223/PELI1 | | 3 |
|  | BP | GO:0060575 | intestinal epithelial cell differentiation | 3/336 | 22/18670 | 0.006902826 | 0.040154127 | 0.028204987 | GATA4/KLF5/TYMS | | 3 |
|  | BP | GO:2000773 | negative regulation of cellular senescence | 3/336 | 22/18670 | 0.006902826 | 0.040154127 | 0.028204987 | BCL6/NAMPT/ZKSCAN3 | | 3 |
|  | BP | GO:0070167 | regulation of biomineral tissue development | 6/336 | 94/18670 | 0.007029162 | 0.040832865 | 0.028681745 | ACVR2B/BMP2/CCL3/CCR1/GJA1/GPM6B | | 6 |
|  | BP | GO:0043903 | regulation of symbiosis, encompassing mutualism through parasitism | 10/336 | 222/18670 | 0.007117976 | 0.04129207 | 0.029004299 | APOBEC3A/CCL3/CCL8/ISG20/JUN/PLSCR1/TMPRSS2/TRIM15/ZC3H12A/ZFP36 | | 10 |
|  | BP | GO:0051101 | regulation of DNA binding | 7/336 | 124/18670 | 0.007194273 | 0.041677505 | 0.029275036 | BCL3/FOXC1/JUN/KLF4/MSX1/PARP1/PLAUR | | 7 |
|  | BP | GO:0071214 | cellular response to abiotic stimulus | 13/336 | 331/18670 | 0.007232392 | 0.041783863 | 0.029349743 | AVPR1A/CRYAB/EGR1/GJA1/IL1B/IRF1/MAP3K14/MYC/NAMPT/PARP1/PTGS2/RELB/TNFRSF1A | | 13 |
|  | BP | GO:0104004 | cellular response to environmental stimulus | 13/336 | 331/18670 | 0.007232392 | 0.041783863 | 0.029349743 | AVPR1A/CRYAB/EGR1/GJA1/IL1B/IRF1/MAP3K14/MYC/NAMPT/PARP1/PTGS2/RELB/TNFRSF1A | | 13 |
|  | BP | GO:0001953 | negative regulation of cell-matrix adhesion | 4/336 | 43/18670 | 0.007319041 | 0.042169244 | 0.029620442 | BCL6/FAM107A/SERPINE1/THBS1 | | 4 |
|  | BP | GO:0014742 | positive regulation of muscle hypertrophy | 4/336 | 43/18670 | 0.007319041 | 0.042169244 | 0.029620442 | EDN1/MIR21/NR4A3/PARP1 | | 4 |
|  | BP | GO:0042129 | regulation of T cell proliferation | 8/336 | 156/18670 | 0.007368683 | 0.042397495 | 0.029780769 | IGFBP2/IL10/IL1B/IL6/IRF1/MIR21/PELI1/SDC4 | | 8 |
|  | BP | GO:0002702 | positive regulation of production of molecular mediator of immune response | 6/336 | 95/18670 | 0.007392535 | 0.042419308 | 0.029796091 | IL1B/IL1R1/IL4R/IL6/NR4A3/WNT5A | | 6 |
|  | BP | GO:0110020 | regulation of actomyosin structure organization | 6/336 | 95/18670 | 0.007392535 | 0.042419308 | 0.029796091 | EDN1/FRMD7/LPAR1/MIR21/SDC4/TACSTD2 | | 6 |
|  | BP | GO:0010812 | negative regulation of cell-substrate adhesion | 5/336 | 68/18670 | 0.00757094 | 0.043384151 | 0.030473815 | BCL6/FAM107A/SERPINE1/TACSTD2/THBS1 | | 5 |
|  | BP | GO:0008585 | female gonad development | 6/336 | 96/18670 | 0.007769281 | 0.044400389 | 0.031187639 | ADAMTS1/ARID5B/FOXC1/ICAM1/INHBB/TIPARP | | 6 |
|  | BP | GO:0019217 | regulation of fatty acid metabolic process | 6/336 | 96/18670 | 0.007769281 | 0.044400389 | 0.031187639 | AVPR1A/IL1B/IRS2/NR4A3/PTGS2/SIRT4 | | 6 |
|  | BP | GO:0032717 | negative regulation of interleukin-8 production | 3/336 | 23/18670 | 0.007833833 | 0.044468832 | 0.031235714 | BCL3/IL10/KLF4 | | 3 |
|  | BP | GO:0089718 | amino acid import across plasma membrane | 3/336 | 23/18670 | 0.007833833 | 0.044468832 | 0.031235714 | RGS2/SLC1A3/SLC7A1 | | 3 |
|  | BP | GO:1901032 | negative regulation of response to reactive oxygen species | 3/336 | 23/18670 | 0.007833833 | 0.044468832 | 0.031235714 | IL10/MIR21/NR4A3 | | 3 |
|  | BP | GO:1903206 | negative regulation of hydrogen peroxide-induced cell death | 3/336 | 23/18670 | 0.007833833 | 0.044468832 | 0.031235714 | IL10/MIR21/NR4A3 | | 3 |
|  | BP | GO:2001039 | negative regulation of cellular response to drug | 3/336 | 23/18670 | 0.007833833 | 0.044468832 | 0.031235714 | IL10/MIR21/NR4A3 | | 3 |
|  | BP | GO:0045601 | regulation of endothelial cell differentiation | 4/336 | 44/18670 | 0.007939049 | 0.044885343 | 0.031528279 | APOLD1/IL1B/MIR21/TNFRSF1A | | 4 |
|  | BP | GO:0061756 | leukocyte adhesion to vascular endothelial cell | 4/336 | 44/18670 | 0.007939049 | 0.044885343 | 0.031528279 | ICAM1/KLF4/MIR21/SELL | | 4 |
|  | BP | GO:0071622 | regulation of granulocyte chemotaxis | 4/336 | 44/18670 | 0.007939049 | 0.044885343 | 0.031528279 | C5AR1/EDN1/MIR223/THBS1 | | 4 |
|  | BP | GO:0086003 | cardiac muscle cell contraction | 5/336 | 69/18670 | 0.008045046 | 0.045423898 | 0.03190657 | ANK2/GATA4/GJA1/KCNE4/STC1 | | 5 |
|  | BP | GO:0001657 | ureteric bud development | 6/336 | 97/18670 | 0.008159666 | 0.04594837 | 0.032274969 | BMP2/FOXC1/MYC/SALL1/SDC4/TACSTD2 | | 6 |
|  | BP | GO:0051591 | response to cAMP | 6/336 | 97/18670 | 0.008159666 | 0.04594837 | 0.032274969 | FOS/FOSB/FOSL1/JUN/JUNB/STC1 | | 6 |
|  | BP | GO:0002673 | regulation of acute inflammatory response | 8/336 | 159/18670 | 0.008225864 | 0.046259546 | 0.032493544 | C2CD4A/C2CD4B/C5AR1/CREB3L3/IL1B/IL6/OSM/PTGS2 | | 8 |
|  | BP | GO:0010595 | positive regulation of endothelial cell migration | 7/336 | 128/18670 | 0.008505193 | 0.04776688 | 0.033552323 | ANXA3/EDN1/MIR21/PTGS2/THBS1/WNT5A/ZC3H12A | | 7 |
|  | BP | GO:0072163 | mesonephric epithelium development | 6/336 | 98/18670 | 0.008563954 | 0.047939244 | 0.033673395 | BMP2/FOXC1/MYC/SALL1/SDC4/TACSTD2 | | 6 |
|  | BP | GO:0072164 | mesonephric tubule development | 6/336 | 98/18670 | 0.008563954 | 0.047939244 | 0.033673395 | BMP2/FOXC1/MYC/SALL1/SDC4/TACSTD2 | | 6 |
|  | BP | GO:0002820 | negative regulation of adaptive immune response | 4/336 | 45/18670 | 0.008592563 | 0.047939244 | 0.033673395 | BCL6/IL1RL1/IL4R/ZC3H12A | | 4 |
|  | BP | GO:0031018 | endocrine pancreas development | 4/336 | 45/18670 | 0.008592563 | 0.047939244 | 0.033673395 | FOXO1/IL6/ONECUT1/WNT5A | | 4 |
|  | BP | GO:0090278 | negative regulation of peptide hormone secretion | 4/336 | 45/18670 | 0.008592563 | 0.047939244 | 0.033673395 | INHBB/MIDN/PIM3/SIRT4 | | 4 |
|  | BP | GO:0010594 | regulation of endothelial cell migration | 10/336 | 229/18670 | 0.008762844 | 0.04882486 | 0.034295467 | ANXA3/EDN1/EPHA2/KLF4/MIR21/PTGS2/STC1/THBS1/WNT5A/ZC3H12A | | 10 |
|  | BP | GO:0001783 | B cell apoptotic process | 3/336 | 24/18670 | 0.008835298 | 0.048918551 | 0.034361278 | BCL6/IL10/IRS2 | | 3 |
|  | BP | GO:0032897 | negative regulation of viral transcription | 3/336 | 24/18670 | 0.008835298 | 0.048918551 | 0.034361278 | CCL3/JUN/ZFP36 | | 3 |
|  | BP | GO:0044062 | regulation of excretion | 3/336 | 24/18670 | 0.008835298 | 0.048918551 | 0.034361278 | AVPR1A/EDN1/STC1 | | 3 |
|  | BP | GO:0071677 | positive regulation of mononuclear cell migration | 3/336 | 24/18670 | 0.008835298 | 0.048918551 | 0.034361278 | CCR1/FPR2/SERPINE1 | | 3 |
|  | BP | GO:0035967 | cellular response to topologically incorrect protein | 8/336 | 161/18670 | 0.008837497 | 0.048918551 | 0.034361278 | ATF3/CCL2/CREB3L3/ERN1/HSPA2/IGFBP1/PPP1R15A/RNF175 | | 8 |
|  | BP | GO:0072527 | pyrimidine-containing compound metabolic process | 6/336 | 99/18670 | 0.008982408 | 0.049590867 | 0.034833525 | APOBEC3A/DHODH/OGG1/TK1/TYMS/UCK1 | | 6 |
|  | BP | GO:1901570 | fatty acid derivative biosynthetic process | 6/336 | 99/18670 | 0.008982408 | 0.049590867 | 0.034833525 | ALOX5AP/AVPR1A/EDN1/FADS1/IL1B/PTGS2 | | 6 |
|  | CC | GO:0030667 | secretory granule membrane | 18/343 | 298/19717 | 5.27E-06 | 0.001797125 | 0.001714189 | B4GALT1/C5AR1/CA4/CD9/CLEC4D/CXCR1/FCAR/FPR1/FPR2/PLAU/PLAUR/SELL/SIRPA/SLC11A1/SLC2A3/STX3/TLR2/TMEM63A | | 18 |
|  | CC | GO:0070820 | tertiary granule | 12/343 | 164/19717 | 3.12E-05 | 0.004416218 | 0.004212413 | CLEC4D/CXCL1/FCAR/FPR1/FPR2/LRG1/PLAU/SIRPA/SLC11A1/SLC2A3/TMEM63A/TNFAIP6 | | 12 |
|  | CC | GO:0070821 | tertiary granule membrane | 8/343 | 73/19717 | 3.89E-05 | 0.004416218 | 0.004212413 | CLEC4D/FCAR/FPR2/PLAU/SIRPA/SLC11A1/SLC2A3/TMEM63A | | 8 |
|  | CC | GO:0101003 | ficolin-1-rich granule membrane | 7/343 | 61/19717 | 8.83E-05 | 0.007530598 | 0.007183068 | CLEC4D/FCAR/FPR1/FPR2/SIRPA/SLC11A1/SLC2A3 | | 7 |
|  | CC | GO:0042581 | specific granule | 11/343 | 160/19717 | 0.000116389 | 0.007937761 | 0.00757144 | ANXA3/CLEC4D/CXCL1/FCAR/FPR2/LRG1/PLAU/PLAUR/SLC2A3/STX3/TMEM63A | | 11 |
|  | CC | GO:0101002 | ficolin-1-rich granule | 11/343 | 185/19717 | 0.000411046 | 0.021156774 | 0.020180408 | CLEC4D/CRISPLD2/FCAR/FPR1/FPR2/LRG1/MNDA/SIRPA/SLC11A1/SLC2A3/TNFAIP6 | | 11 |
|  | CC | GO:0045177 | apical part of cell | 17/343 | 384/19717 | 0.000434303 | 0.021156774 | 0.020180408 | ANK2/C5AR1/CA4/CD9/EDAR/GJA1/IGFBP2/KCNE4/KCNK1/LDLR/MAL2/PTPRH/SHROOM1/SLC12A1/SLC22A4/STC1/STX3 | | 17 |
|  | CC | GO:0035579 | specific granule membrane | 7/343 | 91/19717 | 0.001051262 | 0.043609776 | 0.041597224 | CLEC4D/FCAR/FPR2/PLAU/PLAUR/SLC2A3/TMEM63A | | 7 |
|  | CC | GO:0005942 | phosphatidylinositol 3-kinase complex | 4/343 | 27/19717 | 0.001150991 | 0.043609776 | 0.041597224 | CISH/SOCS1/SOCS2/SOCS3 | | 4 |
|  | CC | GO:0016324 | apical plasma membrane | 14/343 | 318/19717 | 0.001438604 | 0.049056408 | 0.046792499 | ANK2/CA4/CD9/GJA1/IGFBP2/KCNE4/KCNK1/MAL2/PTPRH/SHROOM1/SLC12A1/SLC22A4/STC1/STX3 | | 14 |
|  | MF | GO:0001228 | DNA-binding transcription activator activity, RNA polymerase II-specific | 31/336 | 439/17697 | 3.49E-10 | 2.08E-07 | 1.90E-07 | ATF3/CEBPD/CREB3L3/CSRNP3/EGR1/ELF3/FOS/FOSB/FOSL1/FOSL2/FOXC1/GATA4/GRHL1/IRF1/JUN/JUNB/KLF10/KLF4/KLF5/KLF6/MAFF/MSX1/MYC/NKRF/NR4A1/NR4A2/NR4A3/ONECUT1/PARP1/PLSCR1/TBX10 | | 31 |
|  | MF | GO:0050786 | RAGE receptor binding | 5/336 | 11/17697 | 1.01E-06 | 0.000216792 | 0.000197903 | FPR1/S100A12/S100A8/S100A9/S100P | | 5 |
|  | MF | GO:0005125 | cytokine activity | 17/336 | 220/17697 | 1.09E-06 | 0.000216792 | 0.000197903 | BMP2/CCL2/CCL3/CCL8/CMTM2/CXCL1/EDN1/IL10/IL1B/IL1RN/IL6/INHBB/INHBE/NAMPT/OSM/TNFSF10/WNT5A | | 17 |
|  | MF | GO:0048018 | receptor ligand activity | 26/336 | 482/17697 | 1.90E-06 | 0.000282179 | 0.000257593 | BMP2/CALCA/CCL2/CCL3/CCL8/CMTM2/CXCL1/EDN1/HBEGF/IL10/IL1B/IL1RN/IL6/INHBB/INHBE/NAMPT/NBL1/OSGIN2/OSM/SAA1/SAA2/SEMA3C/SEMA4B/STC1/TNFSF10/WNT5A | | 26 |
|  | MF | GO:0035014 | phosphatidylinositol 3-kinase regulator activity | 5/336 | 17/17697 | 1.23E-05 | 0.001461675 | 0.00133432 | CISH/KLF4/SOCS1/SOCS2/SOCS3 | | 5 |
|  | MF | GO:0036041 | long-chain fatty acid binding | 4/336 | 14/17697 | 0.000109912 | 0.009017396 | 0.008231714 | ALOX5AP/S100A8/S100A9/STX3 | | 4 |
|  | MF | GO:0046935 | 1-phosphatidylinositol-3-kinase regulator activity | 4/336 | 14/17697 | 0.000109912 | 0.009017396 | 0.008231714 | CISH/SOCS1/SOCS2/SOCS3 | | 4 |
|  | MF | GO:0005126 | cytokine receptor binding | 16/336 | 286/17697 | 0.0001221 | 0.009017396 | 0.008231714 | ACVR2B/BMP2/CCL2/CCL3/CCL8/CXCL1/IL10/IL1B/IL1RN/IL6/INHBB/INHBE/LRG1/OSM/SOCS2/TNFSF10 | | 16 |
|  | MF | GO:1901567 | fatty acid derivative binding | 5/336 | 28/17697 | 0.000164319 | 0.009017396 | 0.008231714 | ALOX5AP/PNPLA3/S100A8/S100A9/STX3 | | 5 |
|  | MF | GO:0002020 | protease binding | 10/336 | 128/17697 | 0.000166708 | 0.009017396 | 0.008231714 | ADAMTS4/CSTA/F3/IL1R1/LDLR/PANX1/SELL/SERPINB9/SERPINE1/TNFAIP3 | | 10 |
|  | MF | GO:0019955 | cytokine binding | 10/336 | 128/17697 | 0.000166708 | 0.009017396 | 0.008231714 | CCR1/CXCR1/IL1R1/IL1RL1/IL1RN/IL2RB/NBL1/THBS1/TNFRSF1A/ZFP36 | | 10 |
|  | MF | GO:0035259 | glucocorticoid receptor binding | 4/336 | 16/17697 | 0.000193923 | 0.009615329 | 0.008777549 | ETS2/NR4A1/NR4A2/NR4A3 | | 4 |
|  | MF | GO:0001227 | DNA-binding transcription repressor activity, RNA polymerase II-specific | 14/336 | 242/17697 | 0.000227978 | 0.010434396 | 0.009525252 | ARID5B/ATF3/BACH2/BCL6/BTG2/ETS2/FOXO1/HIVEP1/IRF8/MSX1/MYC/NFIL3/SALL1/ZKSCAN3 | | 14 |
|  | MF | GO:0031406 | carboxylic acid binding | 12/336 | 193/17697 | 0.000330172 | 0.014032291 | 0.012809663 | ALOX5AP/EGLN1/HMMR/NAGS/P4HA1/PADI4/S100A8/S100A9/SLC1A3/STX3/TNFAIP6/TYMS | | 12 |
|  | MF | GO:0004896 | cytokine receptor activity | 8/336 | 96/17697 | 0.000483129 | 0.019164098 | 0.017494338 | CCR1/CXCR1/F3/IL18RAP/IL1R1/IL1RL1/IL2RB/IL4R | | 8 |
|  | MF | GO:0043177 | organic acid binding | 12/336 | 205/17697 | 0.000568248 | 0.021131721 | 0.019290523 | ALOX5AP/EGLN1/HMMR/NAGS/P4HA1/PADI4/S100A8/S100A9/SLC1A3/STX3/TNFAIP6/TYMS | | 12 |
|  | MF | GO:0035325 | Toll-like receptor binding | 3/336 | 12/17697 | 0.001313922 | 0.045987279 | 0.041980426 | S100A8/S100A9/TLR2 | | 3 |
|  | MF | GO:0005539 | glycosaminoglycan binding | 12/336 | 229/17697 | 0.001485485 | 0.049103518 | 0.044825149 | ADAMTS1/ADAMTS5/CCL8/CRISPLD2/DPYSL3/HBEGF/HMMR/SAA1/SELL/THBS1/TLR2/TNFAIP6 | | 12 |
| *Yellow* | BP | GO:0006066 | alcohol metabolic process | 19/301 | 364/18670 | 7.78E-06 | 0.025201767 | 0.023488716 | ACSS1/ADH5/APOA5/CEBPA/CYP27B1/CYP7A1/DGAT1/GPAM/GPD1/LRAT/LSS/MOGAT1/MOGAT2/MVK/PLCD1/SEC14L2/SPHK1/SPHK2/TM7SF2 | | 19 |
|  | BP | GO:0045017 | glycerolipid biosynthetic process | 15/301 | 251/18670 | 1.49E-05 | 0.025201767 | 0.023488716 | DGAT1/ETNK2/GPAM/GPD1/HTR2B/MOGAT1/MOGAT2/MPPE1/MTMR4/PCTP/PIGQ/PIK3C2G/PLD1/PLD6/STARD10 | | 15 |
|  | BP | GO:0046890 | regulation of lipid biosynthetic process | 13/301 | 198/18670 | 2.07E-05 | 0.025201767 | 0.023488716 | APOA5/BRCA1/CYP27B1/CYP7A1/ENHO/GPAM/HTR2B/LSS/MVK/SEC14L2/SPHK1/SPHK2/TM7SF2 | | 13 |
|  | BP | GO:0016126 | sterol biosynthetic process | 8/301 | 81/18670 | 4.82E-05 | 0.032373748 | 0.030173192 | APOA5/CYB5R1/CYP7A1/GPAM/LSS/MVK/SEC14L2/TM7SF2 | | 8 |
|  | BP | GO:0051260 | protein homooligomerization | 17/301 | 351/18670 | 6.00E-05 | 0.032373748 | 0.030173192 | AQP11/CD247/CD3G/CDA/CRYAA/GBP1/KCTD14/KCTD18/LCN2/LGI1/MLKL/PEX14/PFKP/PKD2/STEAP4/TGM2/TIFA | | 17 |
|  | BP | GO:0044706 | multi-multicellular organism process | 13/301 | 222/18670 | 6.78E-05 | 0.032373748 | 0.030173192 | BYSL/CD38/CYP27B1/DDO/DSG1/LIF/MMP9/SLC38A3/SPHK2/STC2/TGFB3/THBD/UMPS | | 13 |
|  | BP | GO:0007565 | female pregnancy | 12/301 | 192/18670 | 6.92E-05 | 0.032373748 | 0.030173192 | BYSL/CD38/CYP27B1/DSG1/LIF/MMP9/SLC38A3/SPHK2/STC2/TGFB3/THBD/UMPS | | 12 |
|  | BP | GO:0048608 | reproductive structure development | 19/301 | 431/18670 | 7.88E-05 | 0.032373748 | 0.030173192 | AFP/ASPM/BOK/CEBPA/CYP27B1/DMRTA1/EOMES/ETNK2/ITGB8/LIF/MAMLD1/MMP19/NOS3/PKD2/PLCD1/PTX3/STC2/TBX3/TLR3 | | 19 |
|  | BP | GO:0061458 | reproductive system development | 19/301 | 434/18670 | 8.63E-05 | 0.032373748 | 0.030173192 | AFP/ASPM/BOK/CEBPA/CYP27B1/DMRTA1/EOMES/ETNK2/ITGB8/LIF/MAMLD1/MMP19/NOS3/PKD2/PLCD1/PTX3/STC2/TBX3/TLR3 | | 19 |
|  | BP | GO:0008654 | phospholipid biosynthetic process | 14/301 | 260/18670 | 8.85E-05 | 0.032373748 | 0.030173192 | ETNK2/GPAM/GPD1/HTR2B/MPPE1/MTMR4/MVK/PCTP/PIGQ/PIK3C2G/PLD1/PLD6/SPHK2/STARD10 | | 14 |
|  | BP | GO:0046165 | alcohol biosynthetic process | 11/301 | 175/18670 | 0.000131511 | 0.03819497 | 0.035598725 | APOA5/CYP27B1/CYP7A1/GPAM/LSS/MVK/PLCD1/SEC14L2/SPHK1/SPHK2/TM7SF2 | | 11 |
|  | BP | GO:1901617 | organic hydroxy compound biosynthetic process | 14/301 | 271/18670 | 0.000137051 | 0.03819497 | 0.035598725 | AMACR/APOA5/CYB5R1/CYP27B1/CYP7A1/GPAM/HDC/LSS/MVK/PLCD1/SEC14L2/SPHK1/SPHK2/TM7SF2 | | 14 |
|  | BP | GO:0045540 | regulation of cholesterol biosynthetic process | 6/301 | 50/18670 | 0.000146221 | 0.03819497 | 0.035598725 | CYP7A1/GPAM/LSS/MVK/SEC14L2/TM7SF2 | | 6 |
|  | BP | GO:0106118 | regulation of sterol biosynthetic process | 6/301 | 50/18670 | 0.000146221 | 0.03819497 | 0.035598725 | CYP7A1/GPAM/LSS/MVK/SEC14L2/TM7SF2 | | 6 |
|  | BP | GO:0006695 | cholesterol biosynthetic process | 7/301 | 75/18670 | 0.000205278 | 0.046625995 | 0.043456665 | APOA5/CYP7A1/GPAM/LSS/MVK/SEC14L2/TM7SF2 | | 7 |
|  | BP | GO:0046474 | glycerophospholipid biosynthetic process | 12/301 | 217/18670 | 0.000219418 | 0.046625995 | 0.043456665 | ETNK2/GPAM/GPD1/HTR2B/MPPE1/MTMR4/PCTP/PIGQ/PIK3C2G/PLD1/PLD6/STARD10 | | 12 |
|  | BP | GO:1902653 | secondary alcohol biosynthetic process | 7/301 | 76/18670 | 0.000223028 | 0.046625995 | 0.043456665 | APOA5/CYP7A1/GPAM/LSS/MVK/SEC14L2/TM7SF2 | | 7 |
|  | BP | GO:0006644 | phospholipid metabolic process | 18/301 | 430/18670 | 0.000229496 | 0.046625995 | 0.043456665 | APOA5/DBI/ETNK2/GPAM/GPD1/HTR2B/MPPE1/MTMR4/MVK/NOD2/PCTP/PIGQ/PIK3C2G/PLCD1/PLD1/PLD6/SPHK2/STARD10 | | 18 |
