# Supplementary material for: Identification of polyunsaturated fatty acids related key modules and genes in metabolic dysfunction-associated fatty liver disease using WGCNA analysis
Source: Front Genet. 2022 Nov 8;13:951224. doi: 10.3389/fgene.2022.951224 (PMC9679514; doi:10.3389/fgene.2022.951224)
Supplement: Supplementary file 8 [file Table4.DOCX]

| **Table S4.RT-qPCR primer pairs sequence** | | |
| --- | --- | --- |
| Gene | Primer | Sequence |
| adamts1 | Forward | TGTGACACTCTCGGAATGGC |
|  | Reverse | ATCGTGCGGCATGTTAAACAC |
| tgfβ3 | Forward | GGACTTCGGCCACATCAAGAA |
|  | Reverse | TAGGGGACGTGGGTCATCAC |
| socs3 | Forward | TGCGCCTCAAGACCTTCAG |
|  | Reverse | GCTCCAGTAGAATCCGCTCTC |
| epha2 | Forward | GCACAGGGAAAGGAAGTTGTT |
|  | Reverse | CATGTAGATAGGCATGTCGTCC |
| zc3h12a | Forward | ACGAAGCCTGTCCAAGAATCC |
|  | Reverse | TAGGGGCCTCTTTAGCCACA |
| gper | Forward | ATGGATGCGACTACTCCAGC |
|  | Reverse | GGAAGAGGGCAATCACGTACT |
| rmnd1 | Forward | GACGGCAAAGAACACTGGC |
|  | Reverse | GGGTCCGGGATGCTTTCAG |
